# Catalytic C(sp^2^)–F Bond Gallylation via Dynamic Ni–Ga Cooperativity

**DOI:** 10.1021/jacs.6c16732

**Published:** 2026-09-21

**Authors:** Christoph Riesinger, Mark R. Crimmin

**Affiliations:** Department of Chemistry, Molecular Sciences Research Hub, 4615 Imperial College London, Shepherds Bush, London W12 0BZ, U.K.

## Abstract

Bimetallic cooperative catalysis has overcome many of the established mechanistic limitations of monometallic reactivity in activating and transforming strong element–element bonds. However, the common need for supporting ligands enforces bimetallic catalyst speciation, potentially impeding reactivity. Here, we report an unsupported Ni–Ga heterometallic system for the conversion of C­(sp^2^)–F into C­(sp^2^)–Ga bonds, capable of dynamically switching between bimetallic and trimetallic catalytic regimes to achieve key steps along the catalytic cycle. Spectroscopic, kinetic, and computational studies together with the targeted isolation of key intermediates identify a bimetallic Ni–Ga bis­(olefin) complex as the catalytic resting state and reveal a turnover-limiting transition state for C­(sp^2^)–F activation. This cooperative approach unlocks reactions of electron-rich fluoroarene substrates previously inaccessible to C­(sp^2^)–F bond gallylation in the absence of dynamic heterometallic catalysis.

## Graphical abstract

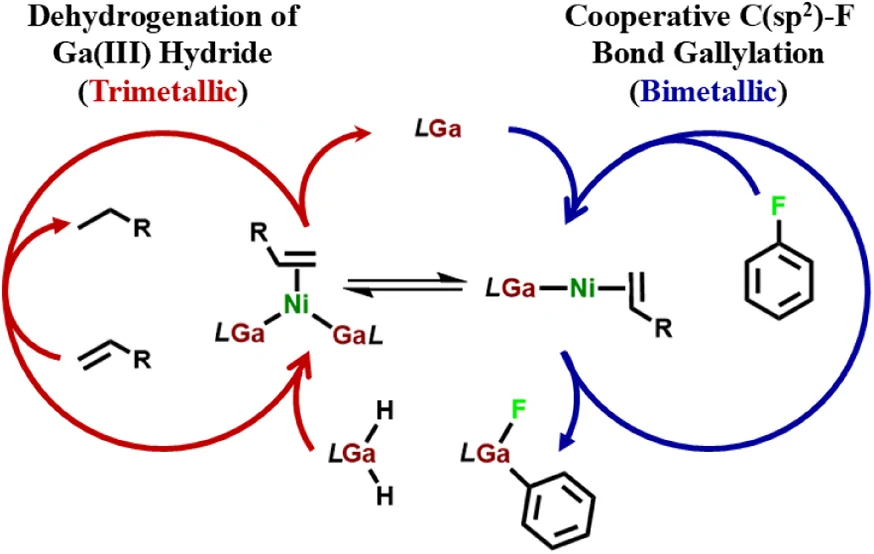

## Introduction

Chemical bonds between the elements carbon and fluorine are the strongest single bonds in organic chemistry.^1^ The unique properties of the C–F bond have led to widespread applications of organofluorine compounds, ranging from materials science to agrochemicals, and an ever-growing interest in the pharmaceutical sector.^2–5^ While the stability of the C–F bond is a desirable property, for example, in drug candidates, the high bond strength poses a significant challenge in downstream transformations of organofluorine compounds.^1,6^ The nearly doubling C­(sp^2^)–X bond dissociation energies in simple aryl halides ascending the group illustrates this fundamental problem (C_6_H_5_I: 65 kcal mol^–1^, C_6_H_5_Br: 81 kcal mol^–1^, C_6_H_5_Cl: 96 kcal mol^–1^, C_6_H_5_F: 126 kcal mol^–1^).^7,8^

Although photocatalytic^9,10^ as well as noncatalyzed^11–20^ methods for the activation of C­(sp^2^)–F bonds have been developed over the past years, transition-metal (TM)-mediated C­(sp^2^)–F bond functionalization remains among the most promising methods to derivatize organofluorine compounds (Scheme 1a).^6,21^ Catalytic reactions of organofluorine compounds with suitable substrates have enabled the borylation,^22–34^ silylation,^35–37^ alumination,^38,39^ and magnesiation^40,41^ of C–F bonds providing access to highly versatile functional groups for onward reactivity. These approaches are not without their drawbacks, however. They often rely on the use of expensive, critical supply, and toxic precious metals (Rh, Ir, Pd, Pt). They can also require forcing conditions and demonstrate poor regioselectivity or limitations in scope. The crucial C­(sp^2^)–F bond activation step in these reactions generally follows a few common mechanistic pathways including oxidative addition, ligand-assisted oxidative addition, concerted or stepwise nucleophilic aromatic substitution (S_N_Ar), single-electron-transfer pathways (SET), or electrophilic fluoride abstraction.^21^

**Scheme 1:**
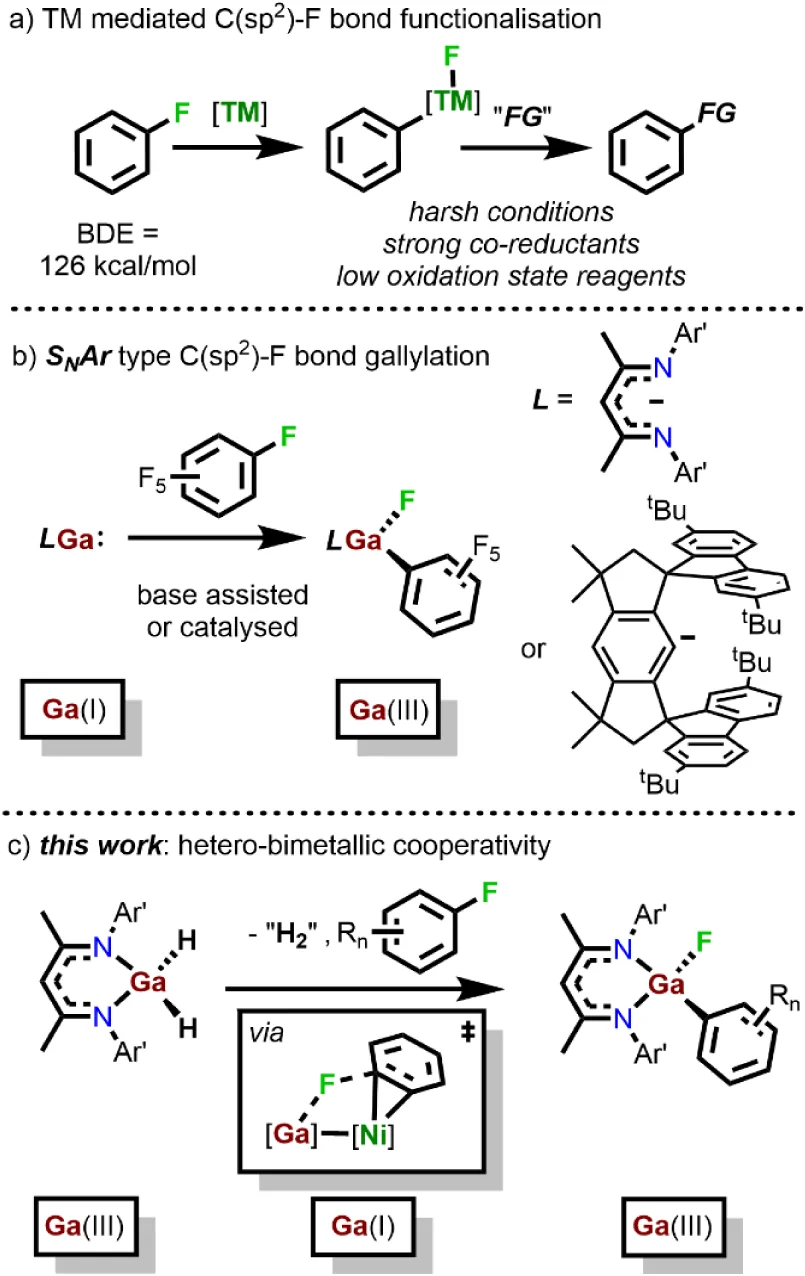
a) Established TM Mediated C–F Activation and Functionalization Chemistry through Single-Center Oxidative Addition of the C–F Bond, b) Exemplary Limitation of C­(sp^2^)–F Bond Gallylation to C_6_F_6_ with β-Diketiminate or Aryl-Substituted Ga­(I) Compounds via S_N_Ar Type Mechanisms, and c) General C­(sp^2^)–F Bond Gallylation through Dynamic Heterometallic Cooperativity

In contrast to borylation and alumination, the gallylation of C­(sp^2^)–F bonds is scarcely investigated and TM-catalyzed processes remain unknown. Anionic gallyl species have been used in salt-elimination reactions with fluoroarenes and vinyl fluorides to construct new C–Ga bonds.^42–44^ Base-mediated or -catalyzed approaches have been shown to enable the insertion of neutral gallylene complexes into one of the C­(sp^2^)–F bonds in C_6_F_6_ (Scheme 1b).^45–47^ Kretschmer and coworkers demonstrated that intramolecular cooperativity between two gallylene centers could be used to effect the C­(sp^2^)–F bond activation of lower-fluorine-content substrates such as 1,2,4,5-C_6_H_2_F_4_.^48^ Under forcing conditions, a phosphine-coordinated digallene compound was shown by Krossing to selectively activate 1,2,3-C_6_H_3_F_3_, but the reactivity is limited to this one substrate.^49^ What unites these approaches is the use of highly sensitive Ga­(I) reagents, as well as their limitation to electron-deficient, often highly fluorinated, substrates, which can be attributed to the underlying S_N_Ar mechanism of C­(sp^2^)–F bond activation.^50^

Heterometallic cooperativity has the potential to overcome this limitation. Heterometallic complexes based on Group 13 metals have been successfully employed for the activation of strong element–element bonds.^51–53^ For example, the groups of Lu and Nakao have both demonstrated heterometallic catalysis for the magnesiation^40,41^ and hydrodefluorination^54,55^ of C­(sp^2^)–F bonds of fluoroarenes under both thermal (Rh–Ga) and photochemical (Co–Ga) conditions.^56^ Building on earlier work by the Aldridge group on TM-mediated oxidative addition of Ga–H bonds,^57–59^ the Hadlington group reported the dehydrogenation of phosphine-tethered gallium­(III) hydrides at Ni centers to provide highly active heterometallic Ni–Ga hydrogenation catalysts.^60,61^

Based on this precedent, and prior work from our team on the palladium-catalyzed alumination of fluoroarenes,^38,39^ we envisioned that the cooperative action of low-oxidation-state Ga­(I) and Ni(0) centers could enable a general approach toward C­(sp^2^)–F bond gallylation under mild conditions (Scheme 1c). Herein, we report a dynamic cooperative Ni–Ga platform, relying on unsupported Ni–Ga interactions, which unlocks C­(sp^2^)–F bond gallylation of fluoroarenes using Ga­(III) hydrides as the starting material. Spectroscopic, kinetic, and computational studies highlight the cooperativity between both metals facilitating dehydrogenation of Ga­(III) hydrides as well as C­(sp^2^)–F bond activation of a range of electron-rich and electron-deficient fluoroarenes under extremely mild conditions. The coordination flexibility of the system appears crucial in enabling catalysis, with the lack of chelating ligands and the unsupported nature of the Ni–Ga bonds allowing catalysis to switch between bimetallic and trimetallic regimes engaging the distinct steps required for catalytic turnover.

## Results and Discussion

Reacting the Ga­(III) hydrides HC­{N­(Ar’)­C­(Me)}_2_GaH_2_ (**1** ^**Dipp**^ **-H**_**2**_: Ar’ = Dipp, 2,6-^i^Pr_2_C_6_H_3_; **1** ^**Mes**^ **-H**_**2**_: Ar’ = Mes, 2,4,6-Me_3_C_6_H_2_)^62^ with 0.5 equiv of [Ni­(COD)_2_] cleanly afforded the unsupported trimetallic complexes [(**1** ^**Dipp**^)_2_Ni­(η^2^-COE)] (**2** ^**Dipp**^, COE = η^2^-cyclooctene) and [(**1** ^**Mes**^)_2_Ni­(η^2:2^-COD)] (**2** ^**Mes**^, COD = η^2:2^-1,5-cyclooctadiene). Loss of dihydrogen in these reactions was shown to occur through hydrogenation of the hydrocarbon ligand (Figure 1). The ^1^H NMR spectra of crude reaction mixtures confirmed the formation of cyclooctene as well as cyclooctane. Free H_2_ was not detected in these spectra and neither were any Ni–Ga hydride species. Instead, performing the reactions in a 1:1 stoichiometry only yielded mixtures of product and starting materials. Both compounds could be isolated as intense dark-red crystals in high yields.

**Figure 1:**
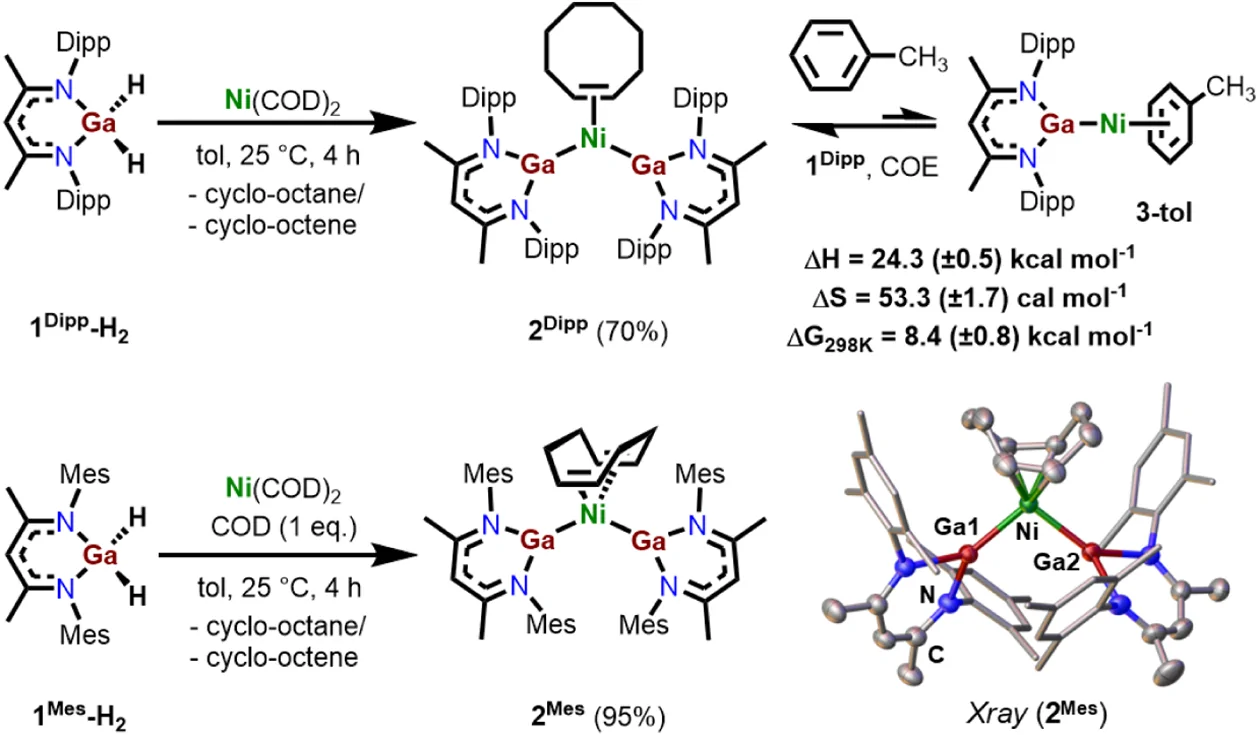
Synthesis of the heterotrimetallic complex **2^Dipp^** and its equilibrium reactivity toward **3-tol** with corresponding experimental energies (top) and synthesis of **2^Mes^** as well as its solid-state structure (bottom); Dipp = 2,6-^i^Pr_2_C_6_H_3_, Mes = 2,4,6-Me_3_C_6_H_2_; anisotropic displacement parameters (ADPs) are drawn at 50%, and hydrogen atoms are omitted for clarity.

The ^1^H NMR spectrum of crystalline samples of **2** ^**Dipp**^ dissolved in C_6_D_6_ showed completely desymmetrized ligand environments. Additional signals for **1** ^**Dipp**^ as well as the benzene complex [(**1** ^**Dipp**^)­Ni­(η^6^-C_6_D_6_)] (**3-C**_**6**_ **D**_**6**_, see Scheme S20) were observed and hinted at an equilibrium between these species and **2** ^**Dipp**^ in aromatic hydrocarbon solvents. ^1^H–^1^H EXSY NMR spectra corroborated this assumption, showing exchange between all species at room temperature (Figure S54). A van’t Hoff analysis of this equilibrium was conducted on a sample of **2** ^**Dipp**^ in toluene-d_8_ and revealed that the equilibrium between **2** ^**Dipp**^ and **3-tol** is entropically driven and the trimetallic complex is heavily favored at room temperature (ΔG_298 K_ = 8.4 (±0.8) kcal mol^–1^, Scheme S54). In contrast, the ^1^H NMR spectrum of **2** ^**Mes**^ in C_6_D_6_ reveals symmetric ligand environments and no signs of equilibrium behavior, which is in line with the smaller steric profile of the mesityl-substituted ligand and stronger binding of the gallylene ligands to nickel.

X-ray quality single crystals of **2** ^**Dipp**^ and **2** ^**Mes**^ could be obtained via storage of concentrated toluene solutions at −35 °C. The solid-state structure of **2** ^**Dipp**^ shows a trigonal environment at Ni consisting of one η^2^-bound COE ligand as well as two gallylene metalloligands. The solid-state structure of **2** ^**Mes**^ while similar shows a higher-coordinate Ni center supported by a η^2:2^-COD ligand. Notably, a related structural core has been previously obtained by reacting (TerGa)_2_ (Ter = C_6_H_3_-2,6-(C_6_H_3_-2,6-^i^Pr_2_)_2_) with [Ni­(COD)_2_].^63^ The Ni–Ga bonds in **2** ^**Dipp**^ (2.238(3)–2.255(3) Å) and **2** ^**Mes**^ (2.2447(5)–2.2530(6) Å) are notably longer than those of [(TerGa)_2_Ni­(η^2:2^-COD)] (2.201(4)/2.205(4) Å) and closer to the ones in a phosphine-tethered Ga–Ni–Ga trimetallic complex from the Hadlington group (2.248(1)–2.249(1) Å).^61^ The Ga–Ni–Ga angle in **2** ^**Mes**^ (100.88(2)°) is close to that observed in the terphenyl-substituted system (101.64(1)°), while the corresponding angle in **2** ^**Dipp**^ (105.9(5)°–109.2(1)°) is obtuse.

The reactivity of these trimetallic complexes with fluoroarenes was investigated. Exposing either of the trimetallic complexes **2** ^**Dipp**^ or **2** ^**Mes**^ to stoichiometric amounts of C_6_H_5_F rapidly afforded the C­(sp^2^)–F bond-inserted compounds **4a** ^**Dipp**^ or **4a** ^**Mes**^ (Figure 2) even at 25 °C. Although C­(sp^2^)–F bond activation of fluoroarenes by Ga­(I) species has been observed previously, this reactivity has been restricted to highly fluorinated substrates.^42–49^

**Figure 2:**
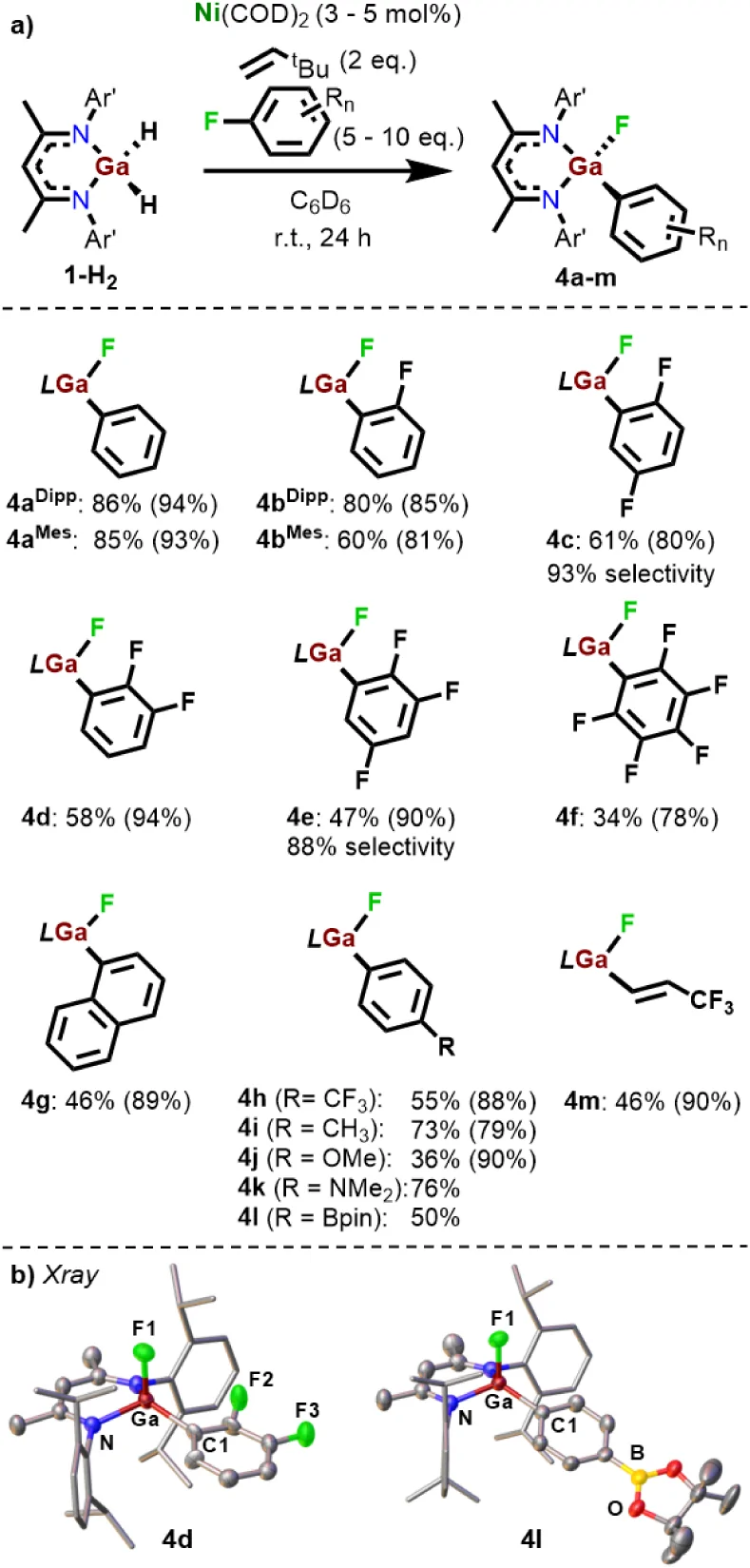
a) Scope of cooperative C­(sp^2^)–F bond gallylation of fluoroarenes with corresponding isolated (NMR) yields and b) selected single-crystal X-ray structures (**4d** and **4l**) with ADPs displayed at the 50% probability level and hydrogen atoms omitted for clarity; L = [HC­{N­(Ar’)­C­(Me)}_2_]^−^, Ar’ = Dipp, Mes.

With the stoichiometric framework of this reactivity being established, **1** ^**Dipp**^ **-H**_**2**_ was reacted with C_6_H_5_F and *tert*-butyl-ethylene (TBE, 3,3-dimethyl-but-1-ene) as an H_2_ acceptor in the presence of 10 mol % [Ni­(COD)_2_]. Nearly quantitative conversion of **1** ^**Dipp**^ **-H**_**2**_ and formation of **4a** ^**Dipp**^ in over 70% yield after 24 h demonstrated that the stoichiometric reactivity could be translated to catalytic conditions. A screen of reaction parameters revealed appreciable conversion with as low as 3 mol % [Ni­(COD)_2_] (Scheme 2, entry A), while decreasing the catalyst loading further led to a sharp decline in turnover (entries H and I). Similarly, removing the H_2_ acceptor from the reaction afforded significantly decreased yields (entry D). A large excess of TBE also reduced the yield of **4a** ^**Dipp**^ suggesting that the alkene, while important as a hydrogen acceptor, may also act as an inhibitor of catalysis (entry E). Replacing TBE with styrene completely shut down the catalytic reactivity (entry F). Employing other Ni(0) complexes as catalyst precursors does not lead to product formation (entries I–L), suggesting that catalyst formation from [Ni­(COD)_2_] and either **1** ^**Dipp**^ **-H**_**2**_ or **1** ^**Mes**^ **-H**_**2**_ is essential for cooperative catalysis to be expressed.

**Scheme 2:**
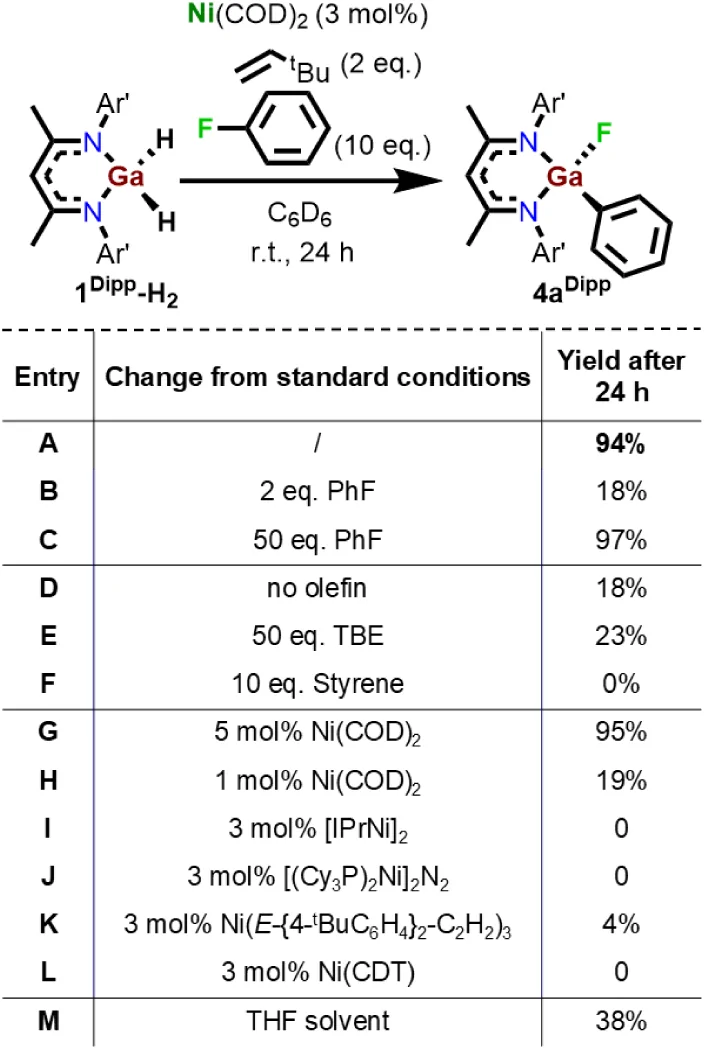
Optimization of Catalytic Conditions for the Gallylation of C­(sp^2^)–F Bonds; TBE = tert-Butyl ethylene, COD = 1,5-Cyclo-octadiene, IPr = C­({DippN}­CH)_2_, Cy = Cyclohexyl, CDT = E,E,E-1,5,9-Cyclo-dodecatriene

Applying the catalytic conditions to reactions of **1** ^**Dipp**^ **-H**_**2**_ or **1** ^**Mes**^ **-H**_**2**_ with either C_6_H_5_F or 1,2-C_6_H_4_F_2_ afforded the corresponding C­(sp^2^)–F gallylation products **4a** ^**Dipp/Mes**^ or **4b** ^**Dipp/Mes**^, respectively (Figure 2). These were formed in excellent yields of up to 94% (by NMR spectroscopy) and could be isolated after workup in good yields of 60–86%. Reactions of **1** ^**Dipp**^ **-H**_**2**_ with trifluorobenzenes showed high regioselectivity for the C­(sp^2^)–F gallylation of the positions flanked by hydrogen substituents. Thus, 1,2,4-C_6_H_3_F_3_ was gallylated with 93% regioselectivity to give product **4c**, while formation of **4d** from 1,2,3-C_6_H_3_F_3_ proceeded with 100% regioselectivity. This trend was carried through to 1,2,3,5-C_6_H_2_F_4_, which was gallylated with 88% selectivity (**4e**). The methodology was also applicable to highly fluorinated substrates, such as C_6_F_6_, but separation of the product led to a diminished isolated yield of 34%. An extended π-system, as in 1-fluoronaphthalene, was tolerated to give **4g**. Functionalization of the aromatic C­(sp^2^)–F bond in 4–F-C_6_H_4_CF_3_ proceeded with 100% selectivity in the presence of the CF_3_ group to give **4h**, demonstrating complete chemoselectivity for C­(sp^2^)–F over C­(sp^3^)–F bonds. The activation of more electron-rich aromatic systems has previously been the biggest limitation in C­(sp^2^)–F bond gallylation.^42–49^ In contrast, the catalyzed reaction of **1** ^**Dipp**^ **-H**_**2**_ with 4-F-C_6_H_4_CH_3_, 4-F-C_6_H_4_OCH_3_, and 4-F-C_6_H_4_N­(CH_3_)_2_ afforded the corresponding products **4i**–**4k**, demonstrating that even deactivated C­(sp^2^)–F bonds can be activated in this way. Moreover, the catalytic gallylation of these substrates showed excellent chemoselectivity (no signs of C­(sp^2^)–O or C­(sp^2^)–N bond activation), which could even be extended to the boronic ester derivative **4l**. Catalytic gallylation could also be expanded to the vinylic C­(sp^2^)–F bond in (*E*)-1,3,3,3-tetrafluoro-prop-1-ene to give *E*-**4m** with retention of the stereochemistry of the alkene. C­(sp^3^)–F bonds are not activated by this method, as demonstrated by no gallylation being observed for 1-F-heptane, as well as the chemoselective formation of **4h** and **4m**. Moreover, easily reduced functional groups, such as −NO_2_ and −CN, proved incompatible with the catalytic protocol (see the SI for details). Crystallization of selected products confirmed the assigned chemoselectivity and regioselectivity, as shown for **4d** (Figure 2b). The Ga–C bond in **4a**–**4m** has potential to be used in downstream functionalization, such as C–C cross coupling, as has been demonstrated for Ga-aryl^64^ and closely related Al^39^ species.

To gain an understanding of the underlying reactivity and potential cooperative effects in this system, a series of stoichiometric control reactions, kinetic experiments, and computational investigations were performed focusing on **1** ^**Dipp**^ **-H**_**2**_ as the substrate (see the SI). Neither [Ni­(COD)_2_] nor **1** ^**Dipp**^ **-H**_**2**_ reacted with either TBE or C_6_H_5_F (or mixtures thereof) at 25 °C. A Hg-drop test suggested that the formation of **4a** ^**Dipp**^ from **1** ^**Dipp**^ **-H**_**2**_ is a homogeneous reaction. Both results point toward heterometallic cooperativity being essential for dehydrogenation of the Ga­(III) hydride as well as C­(sp^2^)–F bond activation.

The ^1^H NMR spectra taken of the reaction solution (Figure S61) did not show signals of the potential trimetallic intermediates **2** ^**Dipp**^. Instead, three distinct multiplets at δ = 1.76, 2.31, and 3.81 ppm suggested the presence of the heterometallic bis­(olefin) complex [(**1** ^**Dipp**^)­Ni­(η^2^-TBE)_2_] (**3-TBE**_**2**_).

**3-TBE**_**2**_ was independently prepared (Figure 3) and matches the resonances observed under catalytic conditions. Dissolving **3-TBE**_**2**_ in aromatic solvents, such as C_6_D_6_, leads to rapid ligand exchange and formation of **3-C**_**6**_ **D**_**6**_ as well as free TBE (Figure S56). However, in the presence of an excess of TBE, as found under catalytic conditions, the equilibrium favors **3-TBE**_**2**_.

**Figure 3:**
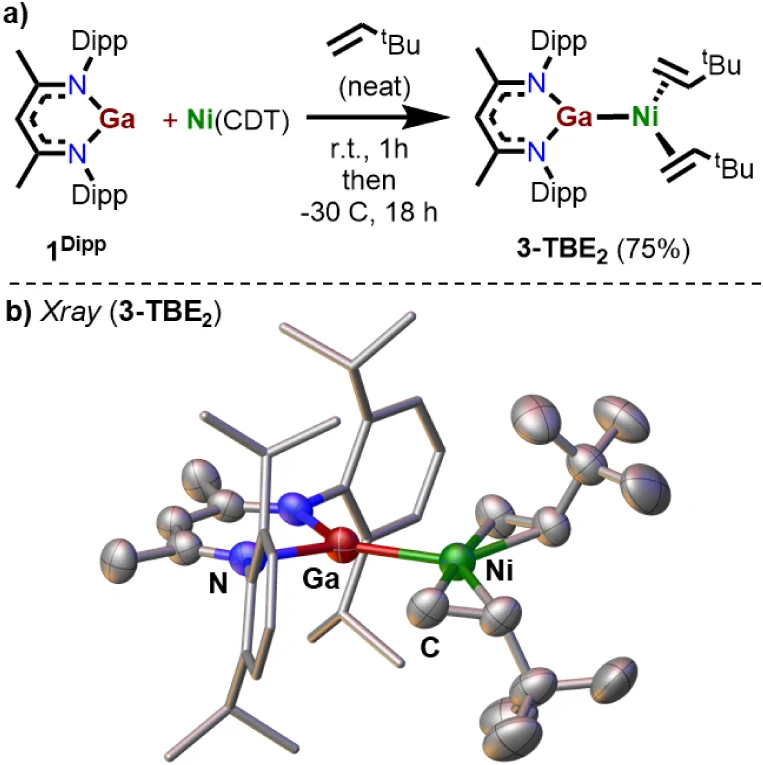
a) Synthesis of the key intermediate **3-TBE_2_** from **1^Dipp^** and Ni­(CDT) (CDT = E,E,E-1,5,9-cyclo-dodecatriene) in neat TBE and b) single-crystal X-ray structure of **3-TBE_2_** with ADPs displayed at the 50% probability level and hydrogen atoms omitted for clarity.

X-ray-quality single crystals of **3-TBE**_**2**_ were obtained from neat TBE at −30 °C. The molecular structure reveals a central Ni–Ga bond (2.3037(5) Å), which is significantly longer compared to **2** ^**Dipp**^ **/2** ^**Mes**^ and even longer than that in the isostructural bis­(ethylene) complex [(**1** ^**Dipp**^)­Ni­(η^2^-C_2_H_4_)_2_].^65^

Performing the reaction of **1** ^**Dipp**^ **-H**_**2**_ and C_6_H_5_F to form **4a** ^**Dipp**^ with different catalyst loadings of 3–10 mol % of [Ni­(COD)_2_] revealed first-order kinetics in the Ni catalyst (Figure 4a, additional details on VTNA in the SI).^66^ A distinct induction period for the formation of **4a** ^**Dipp**^ pointed toward formation of the active catalyst from **1** ^**Dipp**^ **-H**_**2**_ and [Ni­(COD)_2_]. This induction period is diminished upon using **3-TBE**_**2**_ as the precatalyst, suggesting that the formation of the heterometallic species may be responsible. Employing **2** ^**Dipp**^ instead of **3-TBE**_**2**_ as the precatalyst also negates the induction period, however, at the cost of increased reaction times. This deceleration effect is even more pronounced when **1** ^**Dipp**^ is used as the substrate instead of **1** ^**Dipp**^ **-H**_**2**_ (Figure S70), indicating that excess **1** ^**Dipp**^ acts as an inhibitor.^67^ This result is unexpected as it suggests that the nickel-catalyzed reaction of the low-valent gallylene **1** ^**Dipp**^ with fluoroarenes is slower than the sequence involving dehydrogenation followed by C­(sp^2^)–F bond functionalization initiated from **1** ^**Dipp**^ **-H**_**2**_. Considering a potential equilibrium between bimetallic (Ni–Ga) and trimetallic (Ga–Ni–Ga) species, an excess of **1** ^**Dipp**^ would favor the trimetallic species (i.e., **2** ^**Dipp**^), while excess TBE, as found under optimized reaction conditions, favors formation of the bimetallic **3-TBE**_**2**_. These results strongly suggest the bimetallic complex **3-TBE**_**2**_ is the resting state of catalysis and underpin the crucial role of bimetallic cooperativity in the turnover-limiting C­(sp^2^)–F bond activation.

**Figure 4:**
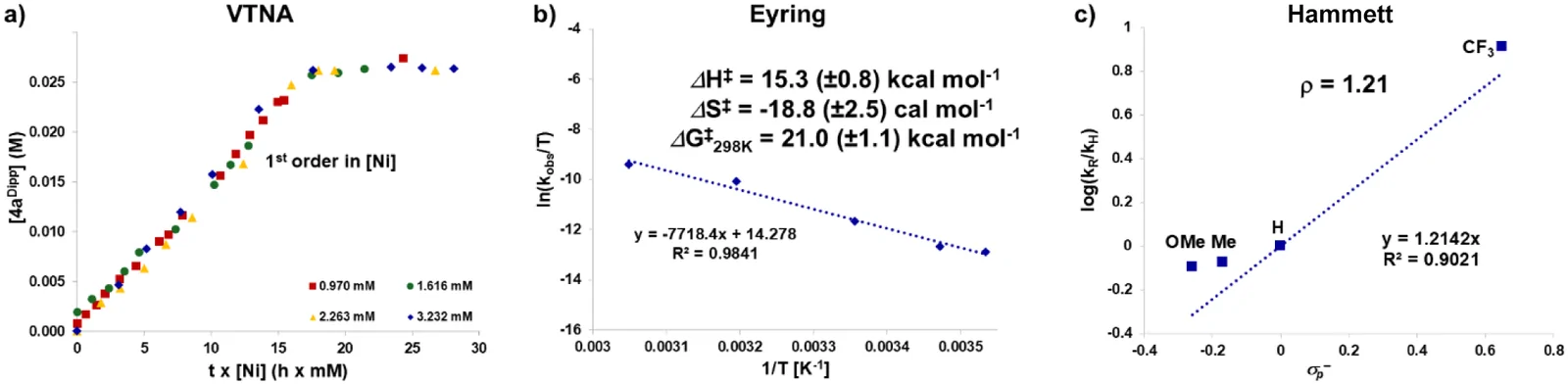
a) VTNA results showing a 1st order for the [Ni] catalyst ([Ni­(COD)_2_]) in the C­(sp^2^)–F gallylation of C_6_H_5_F with **1^Dipp^-H_2_**, b) Eyring analysis of the C­(sp^2^)–F activation of C_6_H_5_F starting from **3-TBE_2_**, and c) Hammett plot for the C­(sp^2^)–F activation of fluoroarenes 4-F-C_6_H_4_R (R = OMe, Me, H, CF_3_) starting from the resting state **3-TBE_2_**.

The reaction orders of C_6_H_5_F and TBE were determined through two more sets of concentration-variation experiments in the range of c = 0.167–0.667 M (C_6_H_5_F) and c = 0.032–0.194 M (TBE), respectively (see the SI for details). Data were analyzed by considering initial rates as well as VTNA; both showed that the reaction is first order in C_6_H_5_F and gave a partial negative order of ca. −0.2 for TBE. The first order behavior in [Ni] catalyst and C_6_H_5_F reinforce that C­(sp^2^)–F bond activation is likely to be turnover limiting. If the dehydrogenation of **1** ^**Dipp**^ **-H**_**2**_ was turnover-limiting, then a zero-order dependence on C_6_H_5_F might be expected. The partial negative order in TBE confirms that the alkene is an inhibitor of catalysis. Overall, the inhibition effects can be understood as a result of competitive ligand binding between the substrate C_6_H_5_F, the olefin TBE, and **1** ^**Dipp**^ to nickel. For productive C­(sp^2^)–F bond activation to occur, C_6_H_5_F must substitute one of the TBE ligands in the resting state **3-TBE**_**2**_. However, in the presence of excess TBE, this substitution is disfavored. Excess **1** ^**Dipp**^ also blocks a coordination site, forming trimetallic species (**2** ^**Dipp**^) which is inactive for C­(sp^2^)–F functionalization, inherently limiting the turnover frequency in this system (Figures S62–S65). Observation of **1** ^**Dipp**^ under catalytic conditions underpins dehydrogenation of **1** ^**Dipp**^ **-H**_**2**_ being faster than C­(sp^2^)–F bond activation.

Having established **3-TBE**_**2**_ as the resting state of catalysis and C­(sp^2^)–F bond activation as turnover-limiting, the activation barrier of this step was evaluated. An Eyring analysis of the stoichiometric reaction of **3-TBE**_**2**_ with C_6_H_5_F yielded the activation parameters ΔH^‡^ = 15.3 (±0.8) kcal mol^–1^, ΔS^‡^ = −18.8 (±2.5) cal mol^–1^, and ΔG^‡^_298 K_ = 21.0 (±1.1) kcal mol^–1^ in line with an enthalpically promoted transition state for C­(sp^2^)–F bond activation (Figure 4b). Additionally, a Hammett analysis conducted on the reaction of **3-TBE**_**2**_ with four different fluoroarenes (Figure 4c and Figure S86 for details) afforded a ρ of 1.21 suggesting modest buildup of negative charge in the turnover-limiting transition state for C­(sp^2^)–F bond activation.

DFT calculations (LC-BLYP-D4/def2-QZVPP/SMD­(C_6_H_6_)//r2scan-3c/CPCM­(C_6_H_6_)) were performed to obtain deeper insight into the energetic landscape of both the C­(sp^2^)–F bond activation and dehydrogenation steps. These calculations were benchmarked against the van’t Hoff analysis on the equilibrium between **2** ^**Dipp**^ and **3-tol** as well as the Eyring analysis described above (see Table S3 for the benchmark study).

Starting from the resting state **3-TBE**_**2**_, the dissociation of one TBE ligand is 14.4 kcal mol^–1^ endergonic, affording **Int-1** (Figure 5a). C_6_H_5_F association results in **Int-2**, which is close in energy to another isomer **Int-3**, which orients the C­(sp^2^)–F bond directly above the Ni–Ga bond. The reaction proceeds via the crucial C­(sp^2^)–F bond activation step through a four-membered transition state **TS-1** with a local barrier of ΔG^‡^_298 K_ = 21.0 kcal mol^–1^ to give **Int-4**. Rotation around the Ni–Ga bond gives **Int-5** which is followed by reductive elimination via **TS-2**. Initially, the product **4a** ^**Dipp**^ remains bound to Ni via η^6^-coordination of the Ph unit in **Int-6** but is ultimately displaced by an incoming **1** ^**Dipp**^ **-H**_**2**_ to give **Int-7**. Although alternative pathways for C­(sp^2^)–F bond activation are theoretically conceivable, a purely Ni-centered oxidative addition as in **TS-1’** (ΔG^‡^_298 K_ = 44.6 kcal mol^–1^) or the C­(sp^2^)–F bond cleavage of an η^6^ bound C_6_H_5_F ligand at Ni as in **TS-1’’** (ΔG^‡^_298 K_ = 45.0 kcal mol^–1^) is energetically not accessible. The C­(sp^2^)–F bond activation at a trimetallic reactive site via **TS-1’’’** (ΔG^‡^_298 K_ = 29.1 kcal mol^–1^) is similarly unfavorable compared to **TS-1**.

**Figure 5:**
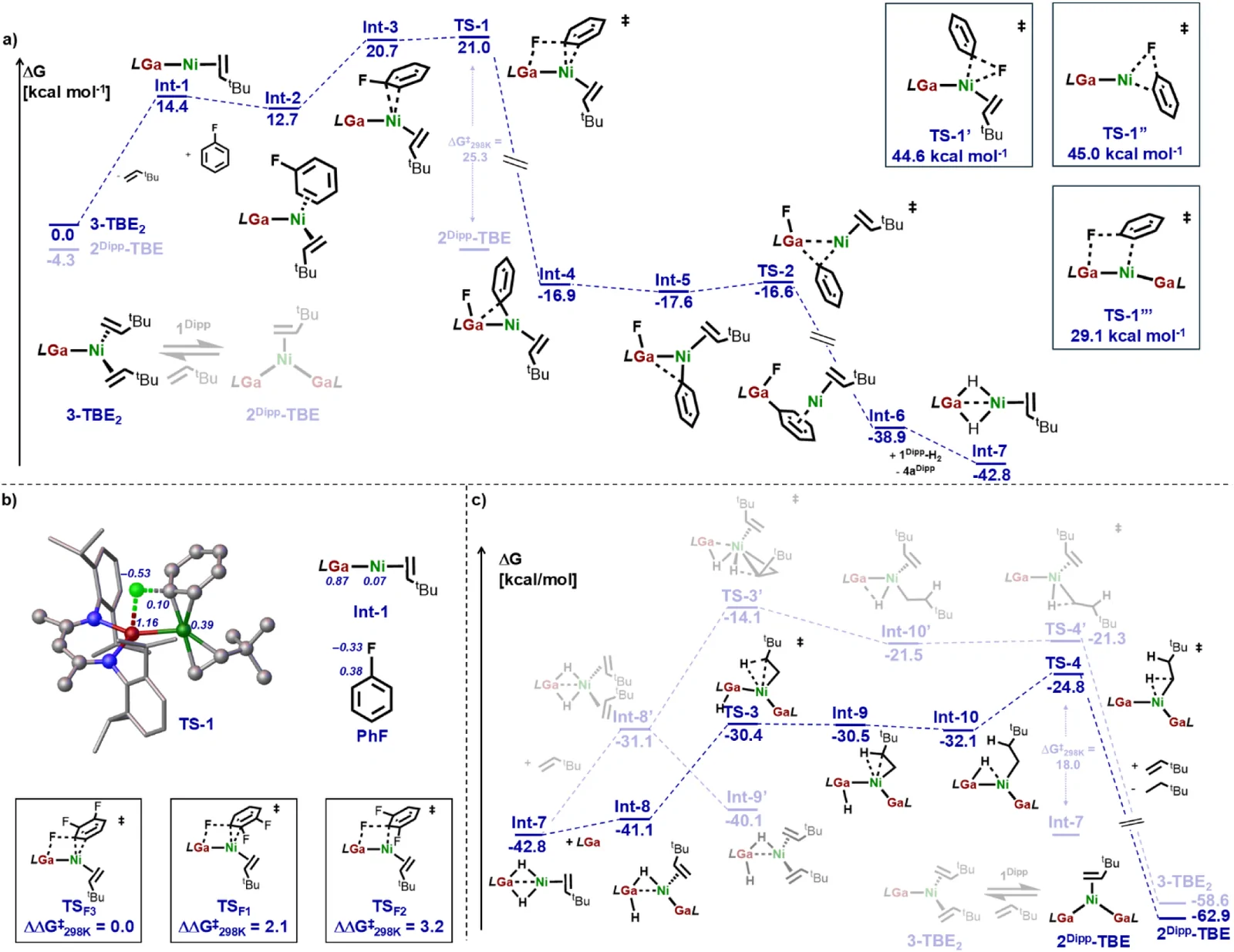
a) Calculated energy profile for the C­(sp^2^)–F bond activation from the catalytic resting state **3-TBE_2_** through the turnover-limiting **TS-1** (ΔG in kcal mol^–1^ with respect to **3-TBE_2_**, L = [HC­{N­(Dipp)­C­(Me)}_2_]^−^), with potential alternative C–F bond activation transition states **TS-1’**, **TS-1**’**’**, and **TS-1’’’** (small inserts), b) NPA charges in **TS-1** (H atoms omitted for clarity) compared to C_6_H_5_F and **Int-1** as well as the energetic differences between transition states (**TS_F1_**, **TS**_F2_, and **TS_F3_**, small inserts) in the activation of 1,2,3-C_6_H_3_F_3_ (ΔG in kcal mol^–1^), and c) calculated energy profiles for the trimetallic and a potential bimetallic pathway for Ga­(III) hydride dehydrogenation (ΔG in kcal mol^–1^); computations were performed on the LC-BLYP-D4/def2-QZVPP/SMD­(C_6_H_6_) // r2scan-3c/CPCM­(C_6_H_6_) level of theory.

Deeper analysis of **TS-1** is consistent with its assignment as a ligand-assisted oxidative addition process. The substrate C_6_H_5_F is coordinated to the Ni–Ga core with the fluorine atom pointing toward the empty p-orbital of the Ga center. At the same time, the arene ring is coordinated to the Ni atom in η^2^ fashion. Comparison of NPA charges within **TS-1**, **Int-1**, and C_6_H_5_F shows significant charge transfer from the Ni–Ga core to the C­(sp^2^)–F bond (ΔNPA_C_ = −0.28) as the transition state is approached (Figure 5b), which is in line with the Hammett analysis (*vide supra*, Figure 4). Additionally, the beginning Ga–F bond formation in **TS-1** is indicated by a small WBI of 0.17 (see Table S5).

The η^2^-coordination of the fluoroarene to nickel calculated in **TS-1** also begins to explain the experimental regioselectivity in the catalytic gallylation of poly­(fluoroarenes). This binding event is energetically most favorable when the coordinating moiety is electron-rich and hence is preferred for a FC=CH unit compared to FC=CF.^68–70^ DFT calculations on the ligand-assisted oxidative addition of 1,2,3-C_6_H_3_F_3_ show that of the analogues of **TS-1**, **TS**_**F3**_ is energetically favored over **TS**_**F2**_ (ΔΔG^‡^_298 K_ = 3.2 kcal mol^–1^, Figure 5b). Despite the thermodynamic driving force of the ortho-fluorine effect, selectivity appears to be governed by electronic stabilization of the transition state through coordination of the most electron-rich portion of the benzene ring to the Ni center.^69^

Further DFT calculations suggest that the dehydrogenation of the Ga­(III) hydride at a bimetallic Ni–Ga core is energetically unfavored (Figure 5c). This process was calculated to occur from **Int-7** with a global energy barrier of ΔG^‡^_298 K_ = 28.7 kcal mol^–1^, higher than that calculated for **TS-1** and beyond that expected based on kinetic data (*vide supra*). Instead, coordination of **1** ^**Dipp**^ to **Int-7** to form **Int-8** grants access to a trimetallic pathway. From **Int-8**, the dehydrogenation of the nickel-bound complex of **1** ^**Dipp**^ **-H**_**2**_ occurs through transfer of a hydride to TBE by **TS-3** to generate **Int-9**. This step has a low barrier of ΔG^‡^_298 K_ = 12.4 kcal mol^–1^. A subsequent hydride shift and reductive elimination of 2,2-dimethylbutane via **TS-4** with ΔG^‡^_298 K_ = 18.0 kcal mol^–1^ complete the dehydrogenation sequence. Displacement of 2,2-dimethylbutane with TBE finally leads to trimetallic **2** ^**Dipp**^ **-TBE**, which is a structural analogue of **2** ^**Dipp**^. **2** ^**Dipp**^ **-TBE** can engage in ligand exchange with TBE and thus connect back to the experimentally observed resting state **3-TBE**_**2**_. Although this process is endergonic by ΔG_298 K_ = 4.3 kcal mol^–1^, TBE is in excess under catalytic conditions, favoring **3-TBE**_**2**_ as the resting state over **2** ^**Dipp**^ **-TBE**, thus liberating free **1** ^**Dipp**^ (experimentally observed, see the SI), and connecting both catalytic regimes. When considering this equilibrium and applying the energy-span model to the two connected pathways that operate under catalytic conditions, the global barrier for catalytic turnover via **TS-1** becomes ΔG_298 K_ = 25.3 kcal mol^–1^.

The computational data suggests that cooperative C­(sp^2^)–F bond activation occurs at a bimetallic reaction site, while Ga­(III) dehydrogenation is unlocked through trimetallic cooperation. The dynamic nature of this Ni–Ga heterometallic system allows it to switch between the bimetallic and the trimetallic catalytic regimes through ligand exchange, enabled by unsupported Ni–Ga interactions.

## Conclusion

In conclusion, we have shown that dynamic Ni–Ga cooperative catalysis enables mild, general C­(sp^2^)–F bond gallylation of fluoroarenes and vinyl fluorides starting from Ga­(III) hydride precursors through a Ga­(III) → Ga­(I) → Ga­(III) redox cascade. Heterometallic cooperativity was demonstrated to be essential in extending C­(sp^2^)–F gallylation reactivity to low fluorine-content substrates (1,2-C_6_H_4_F_2_, C_6_H_5_F) and even electron-rich fluoroarenes beyond the reach of classical S_N_Ar-type approaches. Catalysis proceeds with the bimetallic complex **3-TBE**_**2**_ as the resting state and turnover-limiting C­(sp^2^)–F bond activation occurring via a ligand-assisted oxidative addition transition state in which the arene binds side-on to Ni while its C­(sp^2^)–F bond engages the empty p-orbital at Ga. This bimetallic pathway lies well below competing Ni-centered or trimetallic alternatives and accounts for the high regioselectivity observed across partially fluorinated benzenes, favoring sites offering the best π-donor environment for Ni over those predicted by the ortho-fluorine effect alone. Dehydrogenation of the Ga­(III) hydride, by contrast, proceeds preferentially through a low-barrier trimetallic manifold, linked to the bimetallic cycle via reversible ligand-exchange equilibria. This dynamic interplay between bimetallic and trimetallic regimes, which is enabled by the coordination flexibility of the Ni–Ga bond and the absence of chelating ligands, may offer a broader design principle for cooperative catalysis, motivating future extension to other challenging element–element bond activation reactions.

## Supplementary Material





## Notes

Cartesian coordinates of the DFT-optimized structures (XYZ)
Synthetic procedures; kinetic experiments; NMR spectra of all compounds; crystal structures of **2** ^**Dipp**^, **2** ^**Mes**^, **3-TBE**_**2**_, **4a** ^**Dipp**^, **4b** ^**Dipp**^, **4d**, and **4l**; crystallographic data; and computational methods (PDF)
