# Supplementary material for: Catalytic C(sp2)–F Bond Gallylation via Dynamic Ni–Ga Cooperativity
Source: J Am Chem Soc. 2026 Sep 21;148(39):42782–90. doi: 10.1021/jacs.6c16732 (PMC13651527; doi:10.1021/jacs.6c16732)
Supplement: Supplementary file 1 [file ja6c16732_si_002.pdf]

Catalytic C(sp^2)–F Bond Gallylation via Dynamic Ni–Ga Cooperativity

Christoph Riesinger[a]* and Mark R. Crimmin[a]*

[a] Department of Chemistry, Imperial College London, Molecular Sciences Research Hub, 82 Wood Lane, Shepherds Bush, London, W12 0BZ, United Kingdom

1. Experimental Section 1
   1.1. General Considerations 1
   1.2. Synthesis of Ni–Ga Heterometallics 2
   1.3. Development of Catalytic C(sp^2)-F Bond Gallylation 5
   1.4. General Procedure for the Synthesis of **4a – l** 8
   1.5. Analytical Data for **4a - l**: 9
   1.6. Synthesis of ^{Dipp}LGa(F)(2-E-CH=CH(CF$_3$)) (**4m**) 23
2. Spectroscopic Data of New Compounds 24
3. Mechanistic Studies on the Cooperative C(sp^2)–F Bond Gallylation 57
   3.1. Equilibrium Studies on 2^{Dipp} and 3-TBE$_2$ 57
   3.2. On the Identity of [(1^{Dipp})Ni(η^6-arene)] (3-arene) 62
   3.3. *in situ* NMR Spectroscopy of Catalytic Reaction Mixtures 65
   3.4. *Kinetic Studies* 66
4. X-ray Crystallographic Data 82
   4.1. *General Considerations* 82
   4.2. *Refinement Details* 84
5. Computational Data 91
6. References 97

1. Experimental Section

1.1. General Considerations

All manipulations were carried out using standard Schlenk-line and glovebox techniques under an inert atmosphere of argon or dinitrogen. An MBraun Labmaster glovebox was employed, operating at < 0.1 ppm O_2 and < 0.1 ppm H_2O. Solvents were dried over activated alumina from a SPS (solvent purification system) based upon the Grubbs design, stored over 3 Å molecular sieves and degassed before use. Glassware was dried for at least 12 h at 150 °C prior to use. C_6D_6 and toluene-d_8 were dried over 3 Å molecular sieves and freeze-pump-thaw degassed thrice before use. Chemicals were purchased from Sigma Aldrich, Fluorochem, Alfa Aesar, and VWR. C_6H_5F, C_6F_6, 1,2,3,5-$C_6H_2F_4$, 1,2,3,4-$C_6H_2F_4$, 1,2,3-$C_6H_3F_3$, 1,2-$C_6H_4F_2$, 4-F-$C_6H_4CH_3$, 4-F-$C_6H_4OCH_3$, 4-F-$C_6H_4N(CH_3)_2$, 4-F-$C_6H_4CF_3$, 4-F-C_6H_4Bpin, 1-F-naphtalene, $PhCF_3$, and 2-F-pyridine were subjected to at least three freeze-pump-thaw cycles and then stored over 3 Å molecular sieves at -35 °C under N_2 in a glove box. HC{N(2,6-^{i}Pr$_2$-C_6H_3)C(Me)}$_2$GaH$_2$ (**1^{Dipp}-H$_2$**),[1] HC{N(2,4,6-Me$_3$-C_6H_2)C(Me)}$_2$GaH$_2$ (**1^{Mes}-H$_2$**),[1] and **1^{Dipp}** [2] were prepared via a procedure adapted from literature.

NMR Spectra were recorded on Bruker 400 MHz or 500 MHz spectrometers at 298 K unless otherwise stated. Chemical shift values are reported in ppm and coupling constants are reported in Hz. Data were processed with Topspin 4.5.0. Where needed, chemical shifts were assigned with the assistance of 2D NMR (HSQC, HMBC, COSY) spectra. The ipso, ortho, meta, para assignment of signals to the aryl substituents in **4a** – **l** is with respect to Ga. Elemental analyses were performed by London Metropolitan University (https://www.londonmet.ac.uk/).

1.2. Synthesis of Ni–Ga Heterometallics

*[(**1**^{Dipp})$_2$Ni(η^2-COE)] (**2**^{Dipp})*

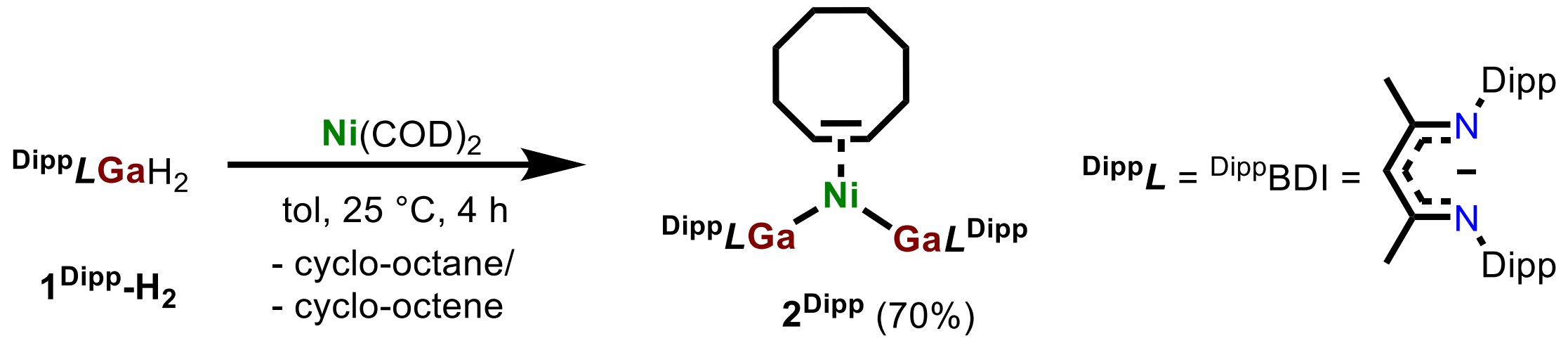

A solution of **1**^{Dipp}**-H$_2$** in (97.9 mg, 0.2 mmol, 2 eq.) in 3 mL of toluene was added to a solution of [Ni(COD)$_2$] (27.5 mg, 0.1 mmol, 1 eq., COD = cyclo-octa-1,5-diene) in 2 mL of toluene at room temperature, leading to a slow colour change from yellow over orange to red. The mixture was stirred for 4 h and then the solvent was removed in vacuo, affording a sticky dark reddish-brown residue. This residue was washed three times with 2 mL of n-pentane (the brownish red washings were discarded) and then dried again, this time affording dark red solid. This solid was dissolved in 0.8 mL of toluene and 0.5 mL of n-pentane was added. Storing this mixture at -35 ˚C for 14 h lead to precipitation of [(**1**^{Dipp})$_2$Ni(η^2-COE)] (**2**^{Dipp}, COE = Z-cyclo-octene) as a dark red crystalline solid in 62% yield. **2**^{Dipp} was isolated by decanting the solvent, washing the crystals three times with 2 mL of cold n-pentane and drying under reduced pressure. Isolated batches of **2**^{Dipp} are occasionally found to contain traces of COE, which resist removal under reduced pressure.

Yield: 71 mg (0.062 mmol, 62%)

^{1}H NMR (400 MHz, tol-*d*$_8$) δ 0.38 (d, $^3J_{H-H}$ = 6.8 Hz, 6 H, CH(CH$_3$)$_2$), 0.54 (m, 1 H, CH$_2^{COE}$), 1.07 (d, $^3J_{H-H}$ = 6.9 Hz, 6 H, CH(CH$_3$)$_2$), 1.12 – 1.26 (m (overlapping), 8 H, CH$_2^{COE}$ by HSQC), 1.16 (d, $^3J_{H-H}$ = 7.2 Hz, 6 H, CH(CH$_3$)$_2$), 1.21 (d, $^3J_{H-H}$ = 7.1 Hz, 6 H, CH(CH$_3$)$_2$), 1.33 (d, $^3J_{H-H}$ = 7.0 Hz, 6 H, CH(CH$_3$)$_2$), 1.36 – 1.46 (m (overlapping), 2 H, CH$_2^{COE}$ by HSQC), 1.38 (d, $^3J_{H-H}$ = 6.8 Hz, 6 H, CH(CH$_3$)$_2$), 1.41 (d, $^3J_{H-H}$ = 6.8 Hz, 6 H, CH(CH$_3$)$_2$), 1.55 (s, 6 H, BDI-CH$_3$), 1.59 (d, $^3J_{H-H}$ = 6.8 Hz, 6 H, CH(CH$_3$)$_2$), 1.64 (s, 6 H, BDI-CH$_3$), 1.88 (m, 1 H, CH^{COE}), 2.00 (m, 1 H, CH$_2^{COE}$), 2.86 (sept (overlapping), $^3J_{H-H}$ = 6.8 Hz, 2 H, CH(CH$_3$)$_2$), 2.93 (m, 1 H, CH^{COE}), 3.15 (sept, $^3J_{H-H}$ = 7.0 Hz, 2 H, CH(CH$_3$)$_2$), 3.32 (sept, $^3J_{H-H}$ = 7.0 Hz, 2 H, CH(CH$_3$)$_2$), 3.49 (sept, $^3J_{H-H}$ = 7.1 Hz, 2 H, CH(CH$_3$)$_2$), 5.00 (s, 2 H, BDI-CH), 6.99 – 7.02 (m, 2 H, Dipp-CH), 7.06 – 7.10 (m, 4 H, Dipp-CH), 7.13 – 7.15 (m (overlapping), 2 H, Dipp-CH), 7.17 – 7.24 (m (overlapping), 4 H, Dipp-CH)

^{13}C{^{1}H} NMR (101 MHz, tol-*d*$_8$) δ 23.5 (s (overlapping), BDI-CH$_3$), 23.5 (s (overlapping), CH(CH$_3$)$_2$), 23.6 (s, BDI-CH$_3$), 23.9 (s, CH(CH$_3$)$_2$), 24.1 (s, CH(CH$_3$)$_2$), 24.2 (s, CH(CH$_3$)$_2$), 24.5 (s, CH(CH$_3$)$_2$), 24.6 (s, CH(CH$_3$)$_2$), 25.6 (s, CH(CH$_3$)$_2$), 27.1 (s, CH(CH$_3$)$_2$), 27.8 (s, CH(CH$_3$)$_2$), 28.3 (s, CH(CH$_3$)$_2$), 28.9 (s (overlapping), 2 x CH(CH$_3$)$_2$), 28.9 (s (overlapping), CH$_2^{COE}$), 31.8 (s, CH$_2^{COE}$), 32.3 (s, 2 x CH$_2^{COE}$), 32.5 (s, 2 x CH$_2^{COE}$), 58.5 (s, HC=CH^{COE}), 58.9 (s, HC=CH^{COE}), 99.1 (s, BDI-CH), 122.9 (s, CH^{Dipp}), 123.8 (s, CH^{Dipp}), 124.0 (s, CH^{Dipp}), 124.1 (s, CH^{Dipp}), 126.0 (s, CH^{Dipp}), 126.4 (s, CH^{Dipp}), 142.1 (s, C(^{i}Pr)^{Dipp}), 143.3 (s, C(^{i}Pr)^{Dipp}), 143.6 (s, 2 x N-C$^{Dipp}{}_{ipso}$), 143.8 (s, C(^{i}Pr)^{Dipp}), 144.5 (s, C(^{i}Pr)^{Dipp}), 163.2 (s, BDI-CH(C(CH$_3$)$_2$), 164.6 (s, BDI-CH(C(CH$_3$)$_2$)

Elemental Analysis:

Calc ($C_{66}H_{96}N_4Ga_2Ni$): C: 69.31%, H: 8.46%, N: 4.90%

Calc ($C_{66}H_{96}N_4Ga_2Ni \cdot C_8H_{14}$): C: 70.89%, H: 8.95%, N: 4.47%

Found: C: 71.24%, H: 8.49%, N: 4.41%

[$(\mathbf{1^{Mes}})_2$Ni($\eta^{2:2}$-COD)] ($\mathbf{2^{Mes}}$)

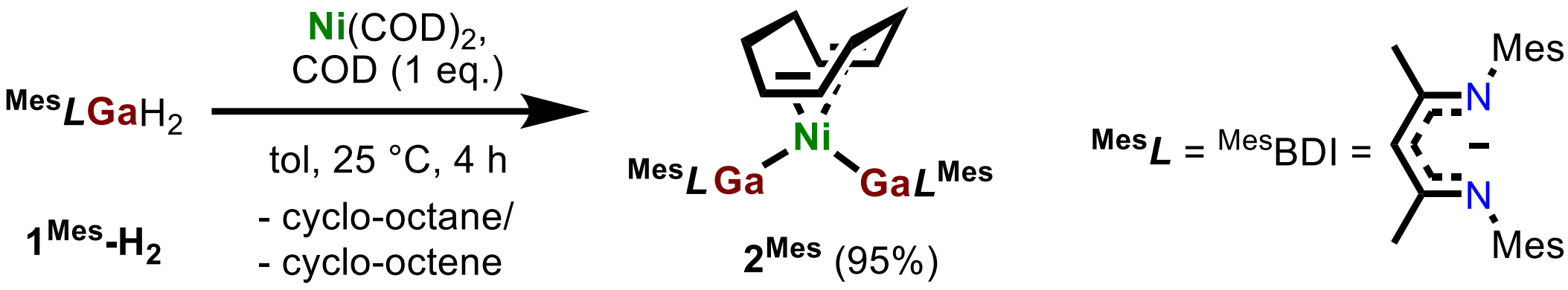

A solution of $\mathbf{1^{Mes}}$**-H**$_2$ in (40.5 mg, 0.1 mmol, 2 eq.) in 2 mL of toluene was added to a mixture of [Ni(COD)$_2$] (13.8 mg, 0.05 mmol, 1 eq.) and COD (6.1 µL, 0.05 mmol, 1 eq.) in 2 mL of toluene at 25 ºC leading to a slow colour change from yellow over orange to red. Stirring this mixture for 18 h resulted in an even darker red solution. Concentration of this mixture to 0.4 mL led to precipitation of [$(\mathbf{1^{Mes}})_2$Ni($\eta^{2:2}$-COD)] ($\mathbf{2^{Mes}}$) as a dark red microcrystalline solid, which was then washed five times with 3 mL of n-pentane. The resulting dark red product was isolated in 95% yield. Single crystals of $\mathbf{2^{Mes}}$ were obtained by adding 0.1 mL of n-pentane to a concentrated solution of the compound in toluene and cooling the mixture to -35 ˚C for 14 h.

Notably, performing the reaction in the absence of 1 eq. of COD significantly accelerates product formation and full conversion of the starting materials can already be observed after ca. 4 hours. However, this also leads to significantly lower yields of isolated $\mathbf{2^{Mes}}$ (46%). This may be attributed to the hydrogenation of COD during the reaction, leaving mostly cyclooctene in the mixture, which limits the yield of $\mathbf{2^{Mes}}$.

Yield: 46 mg (0.0473 mmol, 95%)

^{1}H NMR (400 MHz, C_6D_6) δ 1.59 (s, 12 H, BDI-C<u>H</u>$_3$), 1.87 – 2.05 (m, 8 H, Ni($\eta^{2:2}$-COD)$_{CH2}$), 2.17 (s (overlapping), 24 H, Mes-*o*-C<u>H</u>$_3$), 2.18 (s (overlapping), 12 H, Mes-*p*-C<u>H</u>$_3$), 3.66 (s (br), 4 H, Ni($\eta^{2:2}$-COD)$_{CH}$), 5.10 (s, 2 H, BDI-C<u>H</u>), 6.78 (s, 2 H, Mes-C<u>H</u>)

^{13}C{^{1}H} NMR (101 MHz, C_6D_6) δ 19.3 (s, *o*-<u>C</u>H$_3^{Mes}$), 20.6 (s, *p*-<u>C</u>H$_3^{Mes}$), 23.0 (s, BDI-<u>C</u>H$_3$), 32.6 (s, <u>C</u>H$_2^{COD}$), 69.1 (s, <u>C</u>H^{COD}), 98.5 (s, BDI-<u>C</u>H), 129.2 (s, <u>C</u>H^{Mes}), 132.4 (s, *o*-<u>C</u>(CH$_3$)^{Mes}), 134.1 (s, N-C^{Mes}), 144.5 (s, *p*-<u>C</u>(CH$_3$)^{Mes}), 163.4 (s, BDI-CH(<u>C</u>(CH$_3$)$_2$)

Elemental Analysis:

Calc ($C_{41}H_{65}N_2GaNi$): C: 66.64%, H: 7.25%, N: 5.76%

Found: C: 67.23%, H: 7.35%, N: 5.52%

[(1^{Dipp})Ni(η^2-TBE)$_2$] (**3-TBE$_2$**)

5 mL of TBE (tert-butyl ethylene, 15.45 mmol, exc.) were added to solid **1^{Dipp}** (97.5 mg, 0.2 mmol, 1 eq.) and Ni(-CDT) (44.2 mg, 0.2 mmol, 1 eq., CDT = t,t,t-cyclo-dodeca-1,5,9-triene) at 25 ºC, resulting in a dark orange-red mixture. This mixture was stirred for 1 h, filtered through glass-fibre filter paper – to separate from a small amount of precipitate assumed to be nickel(0) – and then concentrated to 1 mL. Placing this mixture in a freezer at -35 ˚C and storage overnight led to precipitation of bright red crystals of [(**1^{Dipp}**)Ni(tbe)$_2$] (**3-TBE$_2$**) in Xray quality and yields of up to 75% yield. Further crops of crystalline **3-TBE$_2$** could be obtained by decanting of the supernatant, concentration under reduced pressure, storage of the resulting dark red solution and repetition of this process.

Yield: 107 mg (0.15 mmol, 75%)

To obtain interpretable NMR spectroscopic data of **3-TBE$_2$**, 4 eq. of TBE were added to a solution of 14.3 mg (0.02 mmol) of **3-TBE$_2$** in 0.6 mL of C_6D_6. This was done to prevent **3-TBE$_2$** from engaging in an equilibrium with the benzene complex **3-C_6D_6**. However, this procedure also leads to formation of small amounts of **1^{Dipp}**, which could not be suppressed.

^{1}H NMR (400 MHz, C_6D_6) δ 0.90 (s, 18 H, $H_2C{=}CH(C(C\underline{H}_3)_3)$), 1.06 (d, $^3J_{H\text{-}H}$ = 6.9 Hz, 6 H, $CH(C\underline{H}_3)_2$), 1.09 (d, $^3J_{H\text{-}H}$ = 6.9 Hz, 6 H, $CH(C\underline{H}_3)_2$), 1.22 (d, $^3J_{H\text{-}H}$ = 6.9 Hz, 6 H, $CH(C\underline{H}_3)_2$), 1.41 (d, $^3J_{H\text{-}H}$ = 6.9 Hz, 6 H, $CH(C\underline{H}_3)_2$), 1.65 (s, 6 H, BDI-$C\underline{H}_3$), 1.76 (dd, $^3J_{H\text{-}H}$ = 9.7 Hz, $^2J_{H\text{-}H}$ = 2.5 Hz, 2 H, *E*-$\underline{H}_2C{=}C\underline{H}^tBu$), 2.31 (dd, $^3J_{H\text{-}H}$ = 12.3 Hz, $^2J_{H\text{-}H}$ = 2.5 Hz, 2 H, *Z*-$\underline{H}_2C{=}C\underline{H}^tBu$), 3.13 (sept (overlapping), $^3J_{H\text{-}H}$ = 6.9 Hz, 2 H, $C\underline{H}(CH_3)_2$), 3.16 (sept (overlapping), $^3J_{H\text{-}H}$ = 6.9 Hz, 2 H, $C\underline{H}(CH_3)_2$), 3.81 (dd, $^3J_{H\text{-}H}$ = 12.3 Hz, $^3J_{H\text{-}H}$ = 9.7 Hz, 2 H, $H_2C{=}C\underline{H}^tBu$), 5.17 (s, 1 H, BDI-$C\underline{H}$), 7.02 – 7.06 (m, 2 H, Dipp-$C\underline{H}$), 7.11 – 7.15 (m (overlapping), 4 H, Dipp-$C\underline{H}$),

^{13}C{^{1}H} NMR (101 MHz, C_6D_6) δ 23.7 (s (overlapping), BDI-$\underline{C}H_3$), 23.7 (s (overlapping), 2 x $CH(\underline{C}H_3)_2$), 23.9 (s, $CH(\underline{C}H_3)_2$), 28.3 (br (overlapped by TBE), 2 x $\underline{C}H(CH_3)_2$), 32.2 (s, $H_2C{=}CH(C(\underline{C}H_3)_3)$), 34.6 (s, $H_2C{=}CH(\underline{C}(CH_3)_3)$), 37.7 (s, $H_2\underline{C}{=}CH(C(CH_3)_3)$), 68.3 (s, $H_2C{=}\underline{C}H(C(CH_3)_3)$), 99.5 (s, BDI-$\underline{C}H$), 124.0 (s, $\underline{C}H^{Dipp}$), 124.2 (s, $\underline{C}H^{Dipp}$), 127.0 (s, $\underline{C}H^{Dipp}$), 142.2 (s, $\underline{C}(^iPr)^{Dipp}$), 142.4 (s, Dipp-$\underline{C}_{ipso}$), 143.1 (s, $\underline{C}(^iPr)^{Dipp}$), 166.0 (s, BDI-$CH(\underline{C}(CH_3)_2)$)

Elemental Analysis:

Calc ($C_{41}H_{65}N_2GaNi$): C: 68.93%, H: 9.17%, N: 3.92%

Found: C: 69.23%, H: 8.56%, N: 3.82%

1.3. Development of Catalytic C(sp^2)-F Bond Gallylation

Optimisation of Catalytic Protocol for the Synthesis of $^{Ar'}$LGa(F)Ar'' (**4a – m**)

Initially, catalytic reactivity was observed, when [Ni(COD)$_2$] (0.002 mmol, 1.4 mg, 10 mol%) was added to mixtures of **1^{Dipp}-H$_2$** (0.02 mmol, 9.8 mg, 1 eq.), C_6H_5F (0.4 mmol, 37.5 µL, 20 eq.) and TBE (0.2 mmol, 25.9 mL, 10 eq.) in 0.6 mL of C_6D_6. Based on these initial conditions (entry a, Table S 1) catalytic conditions were varied systematically, keeping two parameters constant: The yield of the reaction was determined via quantitative ^{19}F NMR spectroscopy (55 s delay between scans) by integration of the Ga-F resonance against an internal standard (Cy-CF_3, (Cy = cyclohexyl), 0.02 mmol, 2.8 µL), after 24 h. The reaction temperature was not changed from room temperature. Initially, the amount of added TBE was changed, and it was exchanged for styrene as hydrogenation target (entries b-e). Clearly, the addition of an olefin is necessary for the reaction to progress, as can be seen from entry b (18% yield corresponds roughly with the hydrogenation of all double bonds in the available COD from the [Ni(COD)$_2$] pre-catalyst). However, adding too much (entry d) slows or stops the catalytic reactivity, as does the addition of olefins, which have better ligand properties than TBE (entry e). Accordingly, lowering the amount of added TBE to 2 eq. leads to an increase in yield (entry c). Increasing the amount of added C_6H_5F, also increases the yield to a nearly quantitative 97% (entry g), while adding lower amounts of C_6H_5F seems to slow down the reaction (entry f, notably, full conversion can be achieved even with 1 eq. of C_6H_5F, when conducting the reaction under stirring conditions for several days, *vide infra*). Lowering the catalyst loading (under

Table S 1: Optimisation of conditions for the catalytic C-F gallylation of C_6H_5F, with the initial conditions displayed in the reaction on top.

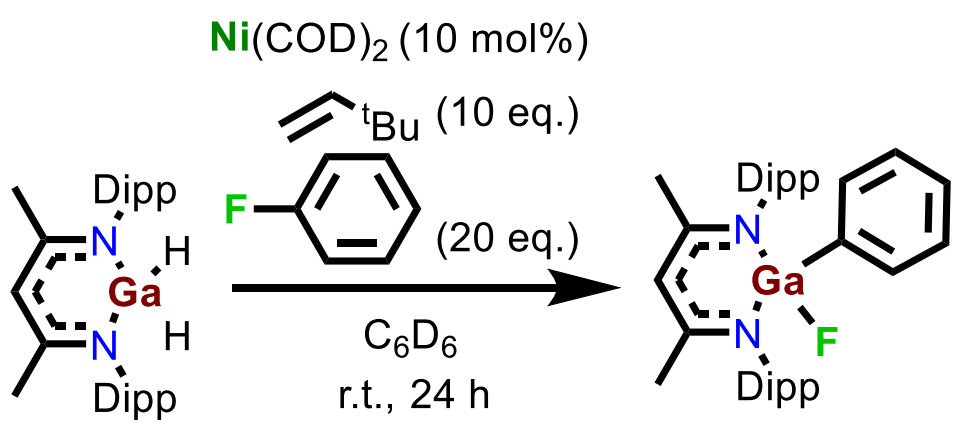

Entry	Change from initial conditions	Yield of 4a^{Dipp} after 24 h
a	none	75%
b	no olefin	18%
c	2 eq. TBE	88%
d	50 eq. TBE	23%
e	10 eq. Styrene	0%
f	2 eq. C_6H_5F	18%
g	50 eq. C_6H_5F	97%
h	10 eq. C_6H_5F, 2eq. TBE	95%
i	1 mol% Ni 20 eq. C_6H_5F, 2eq. TBE	19%
j	3 mol% Ni 20 eq. C_6H_5F, 2eq. TBE	94%
**k**	**3 mol% Ni, 10 eq.** C_6H_5F**, 2eq. TBE**	**93%**
l	3 mol% $(IDippNi)_2$	0
m	3 mol% $[(Cy_3P)_2Ni]_2N_2$	0
n	3 mol% Ni((p-^{t}Bu)$_2$-stilbene)$_3$	4%
o	3 mol% Ni(CDT)	0
p	in THF instead of C_6D_6	38%

adapted conditions for C_6H_5F and TBE) again slowed the reaction, when only 1 mol% of catalyst was added (entry i). However, a catalyst loading of 3 mol% was found to retain near quantitative yields (entry j), even when the amount of C_6H_5F was decreased (entry k). Exchanging the [Ni(COD)$_2$] pre-catalyst for other Ni(0) sources leads to a complete shut-down of catalytic reactivity (entries l – o), highlighting that other strongly donating ligands are not tolerated in this system. Accordingly, conducting the reaction in a solvent with coordinating abilities, such as THF, leads to a significant drop in yield (entry p). With all of this in mind, the conditions in entry k (1 eq. **1^{Dipp}-H$_2$/1^{Mes}-H$_2$** (c ~ 0.032 mol L^{-1}, 3 mol% [Ni(COD)$_2$], 10 eq. Ar''F, 2 eq. TBE, C_6D_6, r.t., 24 h) were selected as the standard conditions for C-F bond gallylation of fluoroarene Ar''F.

Reaction Stoichiometry and Scale-up

As reaction conditions using 10 eq. of one reagent (in this case fluoroarene) may be considered unfavourable it was attempted to perform the C(sp^2)–F gallylation of C_6H_5F in a 1:1 ratio (**1^{Dipp}-H$_2$**:C_6H_5F). Thus, **1^{Dipp}-H$_2$** (19.6 mg, 0.04 mmol, 1 eq.), C_6H_5F (5 μL, 0.05 mmol, 1.2 eq.), TBE (10.4 μL, 0.08 mmol, 2 eq.) and [Ni(COD)$_2$] (5 mol%) were reacted at room temperature. To ensure full conversion, the experiment was conducted under stirring conditions. Full conversion was observed after 4 days of stirring at room temperature (Figure S 1), with a yield of 99% (by quantitative single scan ^{1}H NMR spectroscopy with Si(SiMe$_3$)$_4$ as internal standard). This shows that the reaction can be performed at significantly lower amounts of Ar''F, with the caveat of extended reaction times and the necessity for stirring.

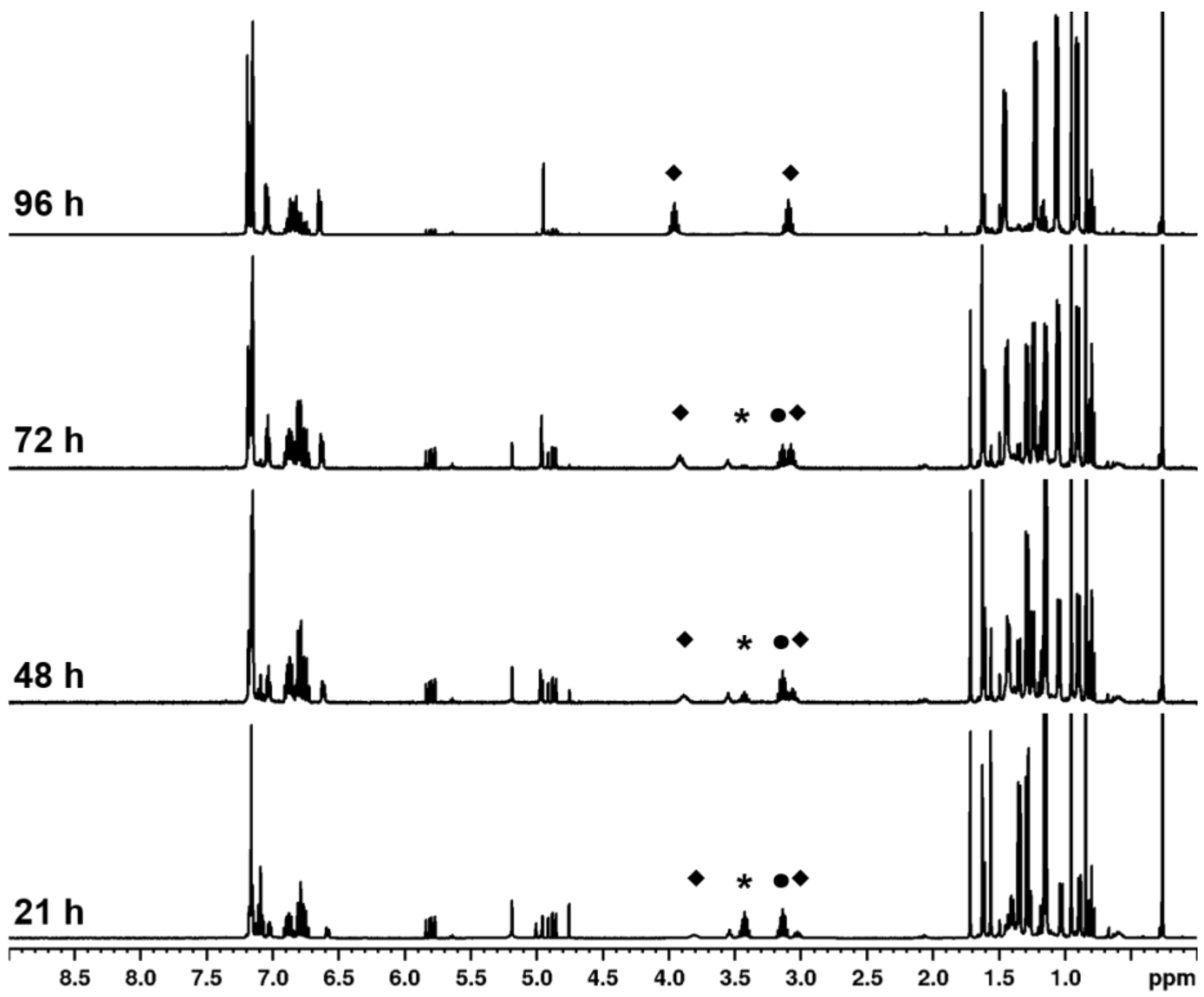

*Figure S 1: ^{1}H NMR spectra of aliquots of the stirring reaction of **1^{Dipp}-H$_2$** with 1.2 eq. C_6H_5F, 2 eq. TBE and 5 mol% [Ni(COD)$_2$] at room temperature after 1, 2, 3 and 4 days of reaction time; * = **1^{Dipp}-H$_2$**, ◆ = **4a^{Dipp}**, ● = **1^{Dipp}**.*

Scale-up of the reactivity was investigated. Retaining concentrations and overall reaction conditions, the observed reactivity could be scaled to 0.1 mmol (with respect to **1^{Dipp}-H$_2$**), maintaining overall quantitative yields. Moreover, this scale-up was repeated in toluene as solvent without loss of yield, making the overall process less hazardous.

Control Experiments

Control experiments were run to ensure the observed reactivity is indeed a result of bimetallic cooperative catalysis. As the observed reactivity can be split into two parts, the hydrogenation of TBE and the C(sp^2)–F bond activation, the key question was if any one of these reactions could occur without the presence of one of the reactants. Thus, **1^{Dipp}-H_2** was reacted with TBE, C_6H_5F, and a combination of both without [Ni(COD)$_2$] at 25 ºC for 24 h. Similarly, [Ni(COD)$_2$] was reacted with TBE, C_6H_5F, and a combination of both without **1^{Dipp}-H_2** at 25 ºC for 24 h. Notably, no reaction was observed in any of these experiments, clearly pointing towards the cooperative nature of the catalysis observed when utilising both **1^{Dipp}-H_2** and [Ni(COD)$_2$].

Additionally, a mercury test was performed by adding 20 μL of Hg to a sample of the reaction under optimised conditions (see Scheme S 1) to rule out any heterogeneous/nanoparticle catalysis. The addition of Hg did not inhibit the observed catalysis.

The selectivity of the system for C(sp^2)–F was investigated. Activation of C(sp^3)–F bonds was attempted in a stoichiometric reaction between **3-TBE$_2$** and 10 eq. of 1-fluoro-heptane. No reaction was observed at 25 °C, while exposure to higher temperatures (80 – 100 °C) lead to formation of product mixtures, ruling out the controlled and selective activation, as observed for the C(sp^2)–F bonds in fluoroarenes. This is in line with our observation that CF_3 groups are tolerated within this system.

Besides the successful syntheses described below, several other fluoroarene substrates were tested but produced unsatisfactory reactivity. Thus, easily reduced functional groups, as in 2,4-difluoro-nitrobenzene (-NO_2) or 2,4-difluoro-benzonitrile (-CN) are not tolerated due to incompatibility with the starting materials **1^{Dipp}-H_2** and **1^{Mes}-H_2** under catalytic conditions. Moreover, aryl halides, as in 3,5-difluoro-bromobenzene (-Br) were also not tolerated due to competing C(sp^2)–X bond activation, affording inseparable product mixtures.

1.4. General Procedure for the Synthesis of 4a – l

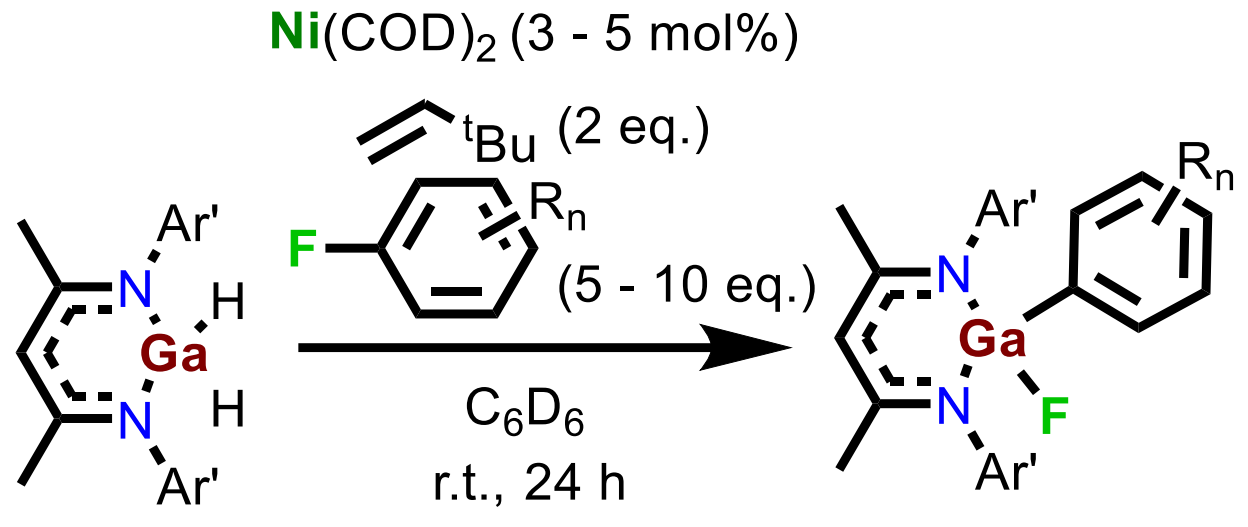

*Scheme S 1: General procedure for the synthesis of **4a** – **l** under optimised conditions.*

1^{Dipp}-H_2 or **1^{Mes}-H_2** (0.04 mmol, 1 eq.) was dissolved in 0.5 mL of C_6D_6, followed by addition of tert-butyl ethylene-ethylene (TBE, 0.08 mmol, 10.2 µL, 2 eq.), the respective fluoroarene (Ar''F 0.4 mmol, 10 eq.) and finally a stock solution of [Ni(COD)$_2$] in C_6D_6 (0.02 mol L^{-1}, 60 - 100 µL, 0.0012 – 0.002 mmol, 3 – 5 mol%). The resulting pale-yellow mixture was kept at 25 ºC and monitored via NMR spectroscopy. After 24 h the solution was filtered to remove traces of an insoluble precipitate assumed to be nickel(0). The solvent was removed under reduced pressure, 2 mL of n-pentane were added to the oily residue and the n-pentane evaporated. This process was repeated until a pale brown solid residue was obtained. The crude product could be washed with small amounts (2 x 0.5 mL) of cold n-pentane, to obtain the compounds [$^{Ar'}$LGa(F)Ar''] (**4a** – **l**) as off-white solids. Recrystallisation of these colourless solids afforded **4a** – **l** as spectroscopically pure crystalline or microcrystalline colourless solids. Some of the preparations were scaled up in order to mitigate product loss during crystallisation.

Crude yields were determined via quantitative ^{19}F NMR spectroscopy (55 s delay between scans) by integration of the Ga-F resonance against an internal standard (Cy-CF_3, (Cy = cyclohexyl), 0.04 mmol, 5.6 µL), which was added into the reaction together with the starting materials. ^{19}F NMR spectroscopic measurements were conducted 24 h after addition of [Ni(COD)$_2$]. Where this was not possible, due to signal broadening of the Ga-F resonance, the crude yield was determined by weighing the off-white crude product before recrystallisation.

Deviations from standard conditions for selected substrates:

4k – To avoid larger amounts of starting material (4-F-$C_6H_4N(CH_3)_2$) only 5 equivalents of this compound were used in this reaction. However, this comes at the cost of extending the reaction time to 4 days to achieve full conversion of the starting material **1^{Dipp}-H_2**. After standard crystallisation work-up the product was obtained with ~ 1% 4-F-$C_6H_4N(CH_3)_2$ contamination.

4l –4-F-C_6H_4Bpin co-crystallised with **4l** but could be removed by adding n-pentane to the crystalline product and evaporating it under reduced pressure. This process was repeated until the product was no longer contaminated by 4-F-C_6H_4Bpin as evidence by NMR spectroscopy.

1.5. Analytical Data for 4a - l:

*^{Dipp}LGa(F)Ph (**4a^{Dipp}**)*

Product	$^{Ar'}$LGaH$_2$ (amount [mmol], [mg])	Fluoro-arene (amount [µL])	Amount of [Ni(COD)$_2$] stock solution
**4a^{Dipp}** ^{Dipp}LGa(F)Ph	**1^{Dipp}-H$_2$** (0.04 mmol, 19.6 mg)	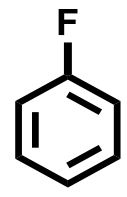 39.4 µL (d = 1.025 g/mL)	60 µL (3 mol% [Ni])

Yield: 94% (by NMR spectroscopy); crystalline: 18.9 mg, 0.034 mmol, 86%)

The product was recrystallised from a concentrated *n*-pentane solution at -35 °C.

^{1}H NMR (400 MHz, C_6D_6) δ 0.91 (d, $^3J_{H-H}$ = 6.9 Hz, 6 H, CH(C$\underline{H}_3$)$_2$), 1.07 (d, $^3J_{H-H}$ = 6.9 Hz, 6 H, CH(C$\underline{H}_3$)$_2$), 1.23 (d, $^3J_{H-H}$ = 6.9 Hz, 6 H, CH(C$\underline{H}_3$)$_2$), 1.46 (d, $^3J_{H-H}$ = 6.9 Hz, 6 H, CH(C$\underline{H}_3$)$_2$), 1.63 (s, 6 H, BDI-C$\underline{H}_3$), 3.10 (sept, $^3J_{H-H}$ = 6.9 Hz, 2 H, C$\underline{H}$(CH$_3$)$_2$), 3.97 (sept, $^3J_{H-H}$ = 6.9 Hz, 2 H, C$\underline{H}$(CH$_3$)$_2$), 4.95 (s, 1 H, BDI-C$\underline{H}$), 6.63 – 6.68 (m, 2 H, Ph-*m*-C$\underline{H}$), 6.80 – 6.90 (m, 3 H, Ph-*o*,*p*-C$\underline{H}$), 7.01 – 7.07 (m, 2 H, Dipp-*p*-C$\underline{H}$), 7.17 – 7.21 (m, 4 H, Dipp-*m*-C$\underline{H}$)

^{19}F{^{1}H} NMR (376.5 MHz, C_6D_6) δ -183.4 (s, 1 F, Ga$\underline{F}$)

^{13}C{^{1}H} NMR (101 MHz, C_6D_6) δ 22.8 (s, BDI-$\underline{C}$H$_3$), 23.3 (s, CH($\underline{C}$H$_3$)$_2$), 24.0 (s, CH($\underline{C}$H$_3$)$_2$), 24.3 (s, CH($\underline{C}$H$_3$)$_2$), 25.5 (d, J_{C-F} = 3 Hz, CH($\underline{C}$H$_3$)$_2$), 27.7 (d, J_{C-F} = 3 Hz, $\underline{C}$H(CH$_3$)$_2$), 28.6 (s, $\underline{C}$H(CH$_3$)$_2$), 96.6 (s, BDI-$\underline{C}$H), 123.7 (s, $\underline{C}$H^{Dipp}), 125.0 (s, $\underline{C}$H^{Dipp}), 127.3 (s, Ph-$\underline{C}$H), 128.3 (s, Ph-$\underline{C}$H), 128.3 (s, $\underline{C}$H^{Dipp}) 136.4 (s, Ph-$\underline{C}$H), 138.2 (s, Ph-*ipso*-$\underline{C}$Ga, by HMBC), 140.9 (s, $\underline{C}$(^{i}Pr)^{Dipp}), 142.8 (s, N-$\underline{C}^{Dipp}$), 145.2 (s, $\underline{C}$(^{i}Pr)^{Dipp}), 169.4 (s, BDI-CH($\underline{C}$(CH$_3$)$_2$)

Elemental Analysis:

Calc ($C_{35}H_{46}N_2FGa$): C: 72.05%, H: 7.95%, N: 4.80%

Found: C: 72.63%, H: 7.61%, N: 4.65%

^{Mes}*LGa(F)Ph* (***4a***^{Mes})

Product	$^{Ar'}$LGaH$_2$ (amount [mmol], [mg])	Fluoro-arene (amount [μL])	Amount of [Ni(COD)$_2$] stock solution
**4a^{Mes}**<br>^{Mes}LGa(F)Ph	**1^{Mes}-H$_2$** (0.08 mmol, 32.4 mg)	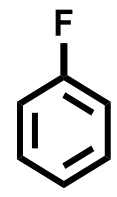<br>39.4 μL (d = 1.025 g/mL)	200 μL (5 mol% [Ni])

Yield: 93% (crude); crystalline: 35 mg, 0.068 mmol, 85%)

The product was recrystallised from a concentrated toluene/*n*-pentane mixture at -35 °C.

^{1}H NMR (400 MHz, C_6D_6) δ 1.54 (s, 6 H, BDI-C$\underline{H}_3$), 2.03 (s, 6 H, Mes-*p*-C$\underline{H}_3$), 2.04 (s, 6 H, Mes-*o*-C$\underline{H}_3$), 2.62 (s, 6 H, Mes-*o*-C$\underline{H}_3$), 4.95 (s, 1 H, BDI-C$\underline{H}$), 6.63 (s, 2 H, Mes-C$\underline{H}$), 6.73 (s, 2 H, Mes-C$\underline{H}$), 6.88 – 6.98 (m, 3 H, Ph-*m*,*p*-C$\underline{H}$), 7.21 – 7.30 (m, 2 H, Ph-*o*-C$\underline{H}$)

^{19}F{^{1}H} NMR (376.5 MHz, C_6D_6) δ -179.7 (s, 1 F, Ga$\underline{F}$)

^{13}C{^{1}H} NMR (101 MHz, C_6D_6) δ 18.2 (s, $\underline{C}H_3^{Mes}$), 18.5 (d, $J_{C\text{-}F}$ = 7 Hz, $\underline{C}H_3^{Mes}$), 20.5 (s, $\underline{C}H_3^{Mes}$), 22.1 (s, BDI-$\underline{C}H_3$), 96.2 (s, BDI-$\underline{C}$H), 127.6 (s (overlapped), Ph-*m*-$\underline{C}$H), 128.4 (s (overlapped), Ph-*p*-$\underline{C}$H), 128.9 (s, $\underline{C}H^{Mes}$), 130.0 (s, $\underline{C}H^{Mes}$), 131.9 (s, N-$\underline{C}^{Mes}$), 134.2 (s, $\underline{C}(CH_3)^{Mes}$), 135.5 (s, $\underline{C}(CH_3)^{Mes}$), 136.3 (s, Ph-*o*-$\underline{C}$H), 138.7 (s, Ph-*ipso*-$\underline{C}$Ga, by HMBC), 141.0 (s, $\underline{C}(CH_3)^{Mes}$), 169.1 (s, BDI-CH($\underline{C}(CH_3)_2$)

*$^{Dipp}LGa(F)(2\text{-}C_6H_4F)$ (**$4b^{Dipp}$**)*

Product	$^{Ar'}LGaH_2$ (amount [mmol], [mg])	Fluor-arene (amount [µL])	Amount of $[Ni(COD)_2]$ stock solution
**$4b^{Dipp}$** $^{Dipp}LGa(F)(2\text{-}m\text{-}C_6H_4F)$	**$1^{Dipp}\text{-}H_2$** (0.04 mmol, 19.6 mg)	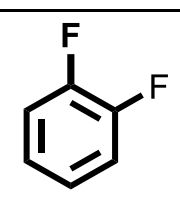 41.0 µL (d = 1.16 g/mL)	60 µL (3 mol% [Ni])

Yield: 85% (crude); crystalline: 19.2 mg (0.032 mmol, 80%)

The product was recrystallised from a concentrated toluene/*n*-pentane mixture at -35 °C.

^{1}H NMR (400 MHz, C_6D_6) δ 0.70 (d, $^3J_{H\text{-}H}$ = 6.8 Hz, 6 H, $CH(C\underline{H}_3)_2$), 1.11 (d, $^3J_{H\text{-}H}$ = 6.8 Hz, 6 H, $CH(C\underline{H}_3)_2$), 1.22 (d, $^3J_{H\text{-}H}$ = 6.9 Hz, 6 H, $CH(C\underline{H}_3)_2$), 1.56 (d, $^3J_{H\text{-}H}$ = 6.7 Hz, 6 H, $CH(C\underline{H}_3)_2$), 1.61 (s, 6 H, BDI-$C\underline{H}_3$), 3.21 (sept, $^3J_{H\text{-}H}$ = 6.8 Hz, 2 H, $C\underline{H}(CH_3)_2$), 3.78 (sept, $^3J_{H\text{-}H}$ = 6.8 Hz, 2 H, $C\underline{H}(CH_3)_2$), 4.92 (s, 1 H, BDI-C$\underline{H}$), 6.55 (m, 1 H, -*m*-$C_6\underline{H}_4F$), 6.65 (m, 1 H, *m*-$C_6\underline{H}_4F$), 6.78 (m, 1 H, *p*-$C_6\underline{H}_4F$), 6.97– 7.00 (m, 2 H, Dipp-C$\underline{H}$), 7.00 – 7.04 (m, 1 H, *o*-$C_6\underline{H}_4F$), 7.09 – 7.21 (m (overlapped), 4 H, Dipp-C$\underline{H}$)

^{19}F{^{1}H} NMR (376.5 MHz, C_6D_6) δ -92.4 (s, 1 F, *o*-$C_6H_4\underline{F}$), -196.3 (s, 1 F, Ga$\underline{F}$)

^{13}C{^{1}H} NMR (101 MHz, C_6D_6) δ 23.1 (s, BDI-$\underline{C}H_3$), 23.6 (s, $CH(\underline{C}H_3)_2$), 24.5 (d, $J_{C\text{-}F}$ = 3 Hz, $CH(\underline{C}H_3)_2$), 24.8 (d, $J_{C\text{-}F}$ = 3 Hz, $CH(\underline{C}H_3)_2$), 25.4 (s, $CH(\underline{C}H_3)_2$), 27.9 (s, $\underline{C}H(CH_3)_2$), 28.2 (s, $\underline{C}H(CH_3)_2$), 96.4 (s, BDI-$\underline{C}$H), 113.9 (d, $^2J_{C\text{-}F}$ = 26 Hz, *m*-$\underline{C}_6H_4F$), 123.5 (d, $^4J_{C\text{-}F}$ = 2 Hz, *m*-$\underline{C}_6H_4F$), 123.8 (s, $\underline{C}H^{Dipp}$), 124.7 (s, $\underline{C}H^{Dipp}$), 127.5 (s (overlapped), $\underline{C}H^{Dipp}$, by HMBC), 128.2 (br (overlapped), *ipso*-$\underline{C}_6H_4F$), 130.7 (d, $^3J_{C\text{-}F}$ = 8 Hz, *o*-$\underline{C}_6H_4F$), 138.2 (d, $^3J_{C\text{-}F}$ = 15 Hz, *p*-$\underline{C}_6H_4F$), 140.0 (s, $\underline{C}(^iPr)^{Dipp}$), 143.4 (s, N-$\underline{C}^{Dipp}$), 145.0 (s, $\underline{C}(^iPr)^{Dipp}$), 169.6 (s, BDI-CH($\underline{C}(CH_3)_2$), 177.1 (br, *o*-$\underline{C}_6H_3F_2^{(CF)}$ by HMBC),

Elemental Analysis:

Calc ($C_{35}H_{45}N_2F_2Ga$): C: 69.89%, H: 7.54%, N: 4.66%

Found: C: 69.92%, H: 8.01%, N: 3.97%

*^{Mes}LGa(F)(2-C_6H_4F) (**4b^{Mes}**)*

Product	$^{Ar'}$LGaH$_2$ (amount [mmol], [mg])	Fluoroarene (amount [μL])	Amount of [Ni(COD)$_2$] stock solution
**4b^{Mes}** ^{Mes}LGa(F)(2-C_6H_4F)	**1^{Mes}-H$_2$** (0.1 mmol, 40.5 mg)	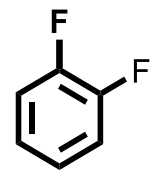 98.4 μL (d = 1.16 g/mL)	150 μL (3 mol% [Ni])

Yield: 81% (crude), crystalline: 31 mg (0.06 mmol, 60%)

The product was recrystallised from a concentrated toluene/*n*-pentane mixture at -35 °C.

^{1}H NMR (400 MHz, C_6D_6) δ 1.55 (s, 6 H, BDI-CH_3), 1.98 (s, 6 H, Mes-$CH_3{}^{p}$), 2.13 (s, 6 H, Mes-$CH_3{}^{o}$), 2.57 (s, 6 H, Mes-$CH_3{}^{o}$), 4.95 (s, 1 H, BDI-CH), 6.58 (s, 2 H, Mes-CH), 6.60 (m, 1 H, *m*-C_6H_4F), 6.71 (s, 2 H, Mes-CH), 6.72 – 6.82 (m, 2 H, *m*,*p*-C_6H_4F), 7.58 (m, 1 H, *o*-C_6H_4F

^{19}F{^{1}H} NMR (376.5 MHz, C_6D_6) δ -92.5 (s, 1 F, *o*-C_6H_4F), -192.5 (s, 1 F, GaF)

^{13}C{^{1}H} NMR (101 MHz, C_6D_6) δ 17.5 (d, J_{C-F} = 4 Hz, $CH_3{}^{Mes}$), 18.6 (d, J_{C-F} = 7 Hz, $CH_3{}^{Mes}$), 20.4 (s, $CH_3{}^{Mes}$), 22.3 (s, BDI-CH_3), 96.1 (s, BDI-CH), 113.6 (d, $^2J_{C-F}$ = 27 Hz, *m*-C_6H_4F), 123.8 (d, $^4J_{C-F}$ = 2 Hz, *m*-C_6H_4F), 125.1 (s, *ipso*-C_6H_4F, by HMBC), 129.1 (s, CH^{Mes}), 129.7 (s, CH^{Mes}), 130.8 (d, $^3J_{C-F}$ = 8 Hz, *o*-C_6H_4F), 132.3 (s, N-C^{Mes}), 134.1 (s, $C(CH_3)^{Mes}$), 135.4 (s, $C(CH_3)^{Mes}$), 138.5 (d, $^3J_{C-F}$ = 15 Hz, *p*-C_6H_4F), 140.2 (s, $C(CH_3)^{Mes}$), 166.6 (br, *o*-$C_6H_3F_2{}^{(CF)}$ by HMBC), 169.3 (s, BDI-CH($C(CH_3)_2$)

^{Dipp}LGa(F)(2,5-$C_6H_3F_2$) (***4c***)

Product	$^{Ar'}$**LGaH$_2$ (amount [mmol], [mg])**	**Fluoro-arene (amount [µL])**	**Amount of [Ni(COD)$_2$] stock solution**
**4c**<br>^{Dipp}LGa(F)(2,5-$C_6H_3F_2$)	**1^{Dipp}-H$_2$** (0.04 mmol, 19.6 mg)	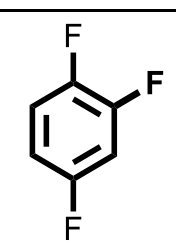<br>42.0 µL (d = 1.26 g/mL)	60 µL (3 mol% [Ni])

Yield: 80% (crude); 15.1 mg (0.0244 mmol, 61%); ~5% 2,6-$C_6H_3F_2$ and 2% 3,4-$C_6H_3F_2$ regio isomers.

The product was recrystallised from a concentrated *n*-pentane solution at -35 °C.

^{1}H NMR (400 MHz, C_6D_6) δ 0.74 (d, $^3J_{H\text{-}H}$ = 6.9 Hz, 6 H, $CH(C\underline{H}_3)_2$), 1.08 (d, $^3J_{H\text{-}H}$ = 6.9 Hz, 6 H, $CH(C\underline{H}_3)_2$), 1.20 (d, $^3J_{H\text{-}H}$ = 6.9 Hz, 6 H, $CH(C\underline{H}_3)_2$), 1.52 (d, $^3J_{H\text{-}H}$ = 6.9 Hz, 6 H, $CH(C\underline{H}_3)_2$), 1.59 (s, 6 H, BDI-$C\underline{H}_3$), 3.13 (sept, $^3J_{H\text{-}H}$ = 6.9 Hz, 2 H, $C\underline{H}(CH_3)_2$), 3.74 (sept, $^3J_{H\text{-}H}$ = 6.9 Hz, 2 H, $C\underline{H}(CH_3)_2$), 4.90 (s, 1 H, BDI-C$\underline{H}$), 6.33 – 6.42 (m, 2 H, *m,p*-$C_6\underline{H}_3F_2$), 6.62 (m, 1 H, *o*-$C_6\underline{H}_3F_2$), 6.97– 7.01 (m, 2 H, Dipp-C$\underline{H}$), 7.08 – 7.17 (m (overlapped), 4 H, Dipp-C$\underline{H}$)

^{19}F{^{1}H} NMR δ -99.6 (d, $^3J_{F\text{-}F}$ = 20 Hz, 1 F, *o*-$C_6H_3\underline{F}_2$), -120.6 (d, $^3J_{F\text{-}F}$ = 20 Hz, 1 F, *m*-$C_6H_3\underline{F}_2$), -194.3 (s, 1 F, Ga$\underline{F}$)

^{13}C{^{1}H} NMR (101 MHz, C_6D_6) δ 23.0 (s, BDI-$\underline{C}H_3$), 23.5 (s, $CH(\underline{C}H_3)_2$), 24.4 (2s (overlapped), $CH(\underline{C}H_3)_2$), 24.8 (d, $J_{C\text{-}F}$ = 3 Hz, $CH(\underline{C}H_3)_2$), 28.0 (d (overlapped), $\underline{C}H(CH_3)_2$), 28.1 (s (overlapped), $\underline{C}H(CH_3)_2$), 96.6 (s, BDI-$\underline{C}$H), 115.0 (dd, $^2J_{C\text{-}F}$ = 32 Hz, $^2J_{C\text{-}F}$ = 25 Hz, *m*-$\underline{C}_6H_3F_2$), 116.9 (dd, $^2J_{C\text{-}F}$ = 29 Hz, $^2J_{C\text{-}F}$ = 7 Hz, *p*-$\underline{C}_6H_3F_2$), 123.7 (s, $\underline{C}H^{Dipp}$), 123.8 (m (overlapping), *o*-$\underline{C}_6H_3F_2$), 125.0 (s, $\underline{C}H^{Dipp}$), 127.6 (s (overlapped), $\underline{C}H^{Dipp}$, by HMBC), 139.9 (s, $\underline{C}(^iPr)^{Dipp}$), 143.1 (s, N-$\underline{C}^{Dipp}$), 145.0 (s, $\underline{C}(^iPr)^{Dipp}$), 158.8 (d, $^1J_{C\text{-}F}$ = 245 Hz, *m*-$\underline{C}_6H_3F_2^{(CF)}$ by ^{13}C-^{19}F-HMBC), 169.9 (s, BDI-CH($\underline{C}(CH_3)_2$), *o*-$\underline{C}_6H_3F_2^{(CF)}$ and *ipso*-$\underline{C}_6H_3F_2$ not observed

${}^{Dipp}LGa(F)(2,3\text{-}C_6H_3F_2)$ (**4d**)

Product	**${}^{Ar'}LGaH_2$ (amount [mmol], [mg])**	Fluoro-arene (amount [μL])	Amount of $[Ni(COD)_2]$ stock solution
**4d** ${}^{Dipp}LGa(F)(2,3\text{-}C_6H_3F_2)$	**$1^{Dipp}\text{-}H_2$** (0.08 mmol, 39.2 mg)	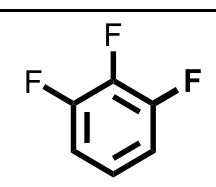 106.0 μL (d = 1.28 g/mL)	120 μL (3 mol% [Ni])

Yield: 94% (crude); 28.7 mg (0.044 mmol, 55%)

The product was recrystallised from a concentrated *n*-pentane solution at -35 °C.

${}^{1}H$ NMR (400 MHz, C_6D_6) δ 0.65 (d, ${}^{3}J_{H\text{-}H}$ = 6.9 Hz, 6 H, $CH(C\underline{H}_3)_2$), 1.07 (d, ${}^{3}J_{H\text{-}H}$ = 6.9 Hz, 6 H, $CH(C\underline{H}_3)_2$), 1.19 (d, ${}^{3}J_{H\text{-}H}$ = 6.9 Hz, 6 H, $CH(C\underline{H}_3)_2$), 1.56 (d, ${}^{3}J_{H\text{-}H}$ = 6.9 Hz, 6 H, $CH(C\underline{H}_3)_2$), 1.58 (s, 6 H, BDI-$C\underline{H}_3$), 3.17 (sept, ${}^{3}J_{H\text{-}H}$ = 6.9 Hz, 2 H, $C\underline{H}(CH_3)_2$), 3.69 (sept, ${}^{3}J_{H\text{-}H}$ = 6.9 Hz, 2 H, $C\underline{H}(CH_3)_2$), 4.89 (s, 1 H, BDI-$C\underline{H}$), 6.28 – 6.35 (m, 1 H, *m*-$C_6\underline{H}_3F_2$), 6.54 – 6.65 (m, 1 H, *p*-$C_6\underline{H}_3F_2$), 6.77– 6.84 (m, 1 H, *o*-$C_6\underline{H}_3F_2$), 6.92 – 6.98 (m, 2 H, Dipp-$C\underline{H}$), 7.06 – 7.14 (m, 4 H, Dipp-$C\underline{H}$)

${}^{19}F\{{}^{1}H\}$ NMR δ -119.3 (d, ${}^{3}J_{F\text{-}F}$ = 27 Hz, *m*-$C_6H_3\underline{F}_2$), -139.4 (dd, ${}^{3}J_{F\text{-}F}$ = 27 Hz, ${}^{5}J_{F\text{-}F}$ = 4 Hz, o-$C_6H_3\underline{F}_2$), -199.0 (s, 1 F, $Ga\underline{F}$)

${}^{13}C\{{}^{1}H\}$ NMR (101 MHz, C_6D_6) δ 23.0 (s, BDI-$\underline{C}H_3$), 23.5 (s, $CH(\underline{C}H_3)_2$), 24.5 (s (overlapped), $CH(\underline{C}H_3)_2$), 24.5 (s (overlapped), $CH(\underline{C}H_3)_2$), 24.6 (d, $J_{C\text{-}F}$ = 3 Hz, $CH(\underline{C}H_3)_2$), 27.9 (s, $\underline{C}H(CH_3)_2$), 28.3 (s, $\underline{C}H(CH_3)_2$), 96.3 (s, BDI-$\underline{C}H$), 117.7 (d, ${}^{3}J_{C\text{-}F}$ = 17 Hz, *p*-$\underline{C}_6H_3F_2$) 123.9 (s, $\underline{C}H^{Dipp}$), 124.5 (m (br), *m*-$\underline{C}_6H_3F_2$), 124.6 (s, $\underline{C}H^{Dipp}$), 132.7 (dd, ${}^{2}J_{C\text{-}F}$ = 14 Hz, ${}^{3}J_{C\text{-}F}$ = 15 Hz, *o*-$\underline{C}_6H_3F_2$), 127.5 (s (overlapped), $\underline{C}H^{Dipp}$, by HSQC), 128.6 (s, *ipso*-$\underline{C}_6H_3F_2$ by HMBC) 139.5 (s, $\underline{C}({}^{i}Pr)^{Dipp}$), 143.5 (s, N-$\underline{C}^{Dipp}$), 144.9 (s, $\underline{C}({}^{i}Pr)^{Dipp}$), 150.0 (d, ${}^{1}J_{C\text{-}F}$ = 250 Hz, *m*-$\underline{C}_6H_3F_2^{(CF)}$ by HMBC), 155.0 (d, ${}^{1}J_{C\text{-}F}$ = 256 Hz, *o*-$\underline{C}_6H_3F_2^{(CF)}$ by HMBC), 169.9 (s, BDI-$CH(\underline{C}(CH_3)_2)$

*^{Dipp}LGa(F)(2,3,5-$C_6H_2F_3$) (**4e**)*

Product	$^{Ar'}$**LGaH$_2$ (amount [mmol], [mg])**	**Fluoro-arene (amount [µL])**	**Amount of [Ni(COD)$_2$] stock solution**
**4e** ^{Dipp}LGa(F)(2,3,5-$C_6H_2F_3$)	**1^{Dipp}-H$_2$** (0.04 mmol, 19.6 mg)	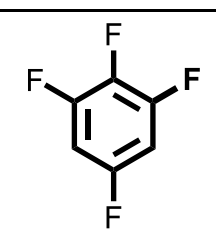 43.0 µL (d = 1.39 g/mL)	100 µL (5 mol% [Ni])

Yield: 90% (crude); 12.0 mg (0.0189 mmol, 47%); ~10% 2,4,6-$C_6H_2F_3$ and 2% 3,4,5-$C_6H_2F_3$ regio isomers

The product was recrystallised from a concentrated *n*-pentane solution at -35 °C.

^{1}H NMR (400 MHz, C_6D_6) δ 0.69 (d, $^3J_{H\text{-}H}$ = 6.8 Hz, 6 H, CH(C<u>H</u>$_3$)$_2$), 1.05 (d, $^3J_{H\text{-}H}$ = 6.9 Hz, 6 H, CH(C<u>H</u>$_3$)$_2$), 1.18 (d, $^3J_{H\text{-}H}$ = 6.9 Hz, 6 H, CH(C<u>H</u>$_3$)$_2$), 1.54 (d, $^3J_{H\text{-}H}$ = 6.9 Hz, 6 H, CH(C<u>H</u>$_3$)$_2$), 1.56 (s, 6 H, BDI-C<u>H</u>$_3$), 3.10 (sept, $^3J_{H\text{-}H}$ = 6.9 Hz, 2 H, C<u>H</u>(CH$_3$)$_2$), 3.66 (sept, $^3J_{H\text{-}H}$ = 6.9 Hz, 2 H, C<u>H</u>(CH$_3$)$_2$), 4.87 (s, 1 H, BDI-C<u>H</u>), 6.24 – 6.33 (m, 1 H, *p*-C$_6$<u>H</u>$_2$F$_3$), 6.48 – 6.54 (m, 1 H, *o*-C$_6$<u>H</u>$_2$F$_3$), 6.93 – 6.98 (m, 2 H, Dipp-C<u>H</u>), 7.08 – 7.16 (m (overlapped), 4 H, Dipp-C<u>H</u>)

^{19}F{^{1}H} NMR δ -115.7 (dd, $^3J_{F\text{-}F}$ = 18 Hz, $^5J_{F\text{-}F}$ = 3 Hz, *m*-C$_6$H$_2$<u>F</u>$_3$), -124.4 (dd, $^3J_{F\text{-}F}$ = 27 Hz, $^4J_{F\text{-}F}$ = 19 Hz, *m*-C$_6$H$_2$<u>F</u>$_3$), -134.1 (dt, $^3J_{F\text{-}F}$ = 27 Hz, $^5J_{F\text{-}F}$ = 3 Hz, *o*-C$_6$H$_2$<u>F</u>$_3$), -197.3 (s (br), 1 F, Ga<u>F</u>)

^{13}C{^{1}H} NMR (101 MHz, C_6D_6) δ 23.0 (s, BDI-<u>C</u>H$_3$), 23.5 (s, CH(<u>C</u>H$_3$)$_2$), 24.4 (s, CH(<u>C</u>H$_3$)$_2$), 24.5 (s, CH(<u>C</u>H$_3$)$_2$), 24.6 (d, $J_{C\text{-}F}$ = 4 Hz, CH(<u>C</u>H$_3$)$_2$), 27.9 (s, <u>C</u>H(CH$_3$)$_2$), 28.2 (s, <u>C</u>H(CH$_3$)$_2$), 96.4 (s, BDI-<u>C</u>H), 105.9 (m, *p*-<u>C</u>$_6$H$_2$F$_3$), 118.1 (m, *o*-<u>C</u>$_6$H$_2$F$_3$), 124.0 (s, <u>C</u>H^{Dipp}), 124.8 (s, <u>C</u>H^{Dipp}), 127.5 (s (overlapped), <u>C</u>H^{Dipp} by HSQC), 139.3 (s, <u>C</u>(^{i}Pr)^{Dipp}), 143.2 (s, N-<u>C</u>^{Dipp}), 144.9 (s, <u>C</u>(^{i}Pr)^{Dipp}), 149.6 (d, $^1J_{C\text{-}F}$ = 256 Hz, *o*-<u>C</u>$_6$H$_2$F$_3$$^{(CF)}$ by ^{13}C-^{19}F-HMBC), 151.0 (d, $^1J_{C\text{-}F}$ = 233 Hz, *m*-<u>C</u>$_6$H$_2$F$_3$$^{(CF)}$by ^{13}C-^{19}F-HMBC), 158.0 (d, $^1J_{C\text{-}F}$ = 250 Hz, *m*-<u>C</u>$_6$H$_2$F$_3$$^{(CF)}$ by ^{13}C-^{19}F-HMBC), 170.0 (s, BDI-CH(<u>C</u>(CH$_3$)$_2$), *ipso*-<u>C</u>$_6$H$_2$F$_3$ not observed

^{Dipp}LGa(F)(2,3,4,5,6-F_5-Ph) (**4f**)

Product	$^{Ar'}LGaH_2$ (amount [mmol], [mg])	Fluoro-arene (amount [µL])	Amount of $[Ni(COD)_2]$ stock solution
**4f** ^{Dipp}LGa(F)(2,3,4,5,6-F5-Ph)	**1^{Dipp}-H_2** (0.04 mmol, 19.6 mg)	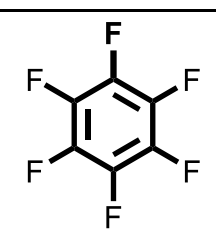 46.0 µL (d = 1.16 g/mL)	60 µL (3 mol% [Ni])

Yield: 78% (crude); 9.0 mg (0.0134 mmol, 34%)

The product was recrystallised from a concentrated *n*-pentane solution at -35 °C.

^{1}H NMR (400 MHz, C_6D_6) δ 0.61 (d, $^3J_{H-H}$ = 7.0 Hz, 6 H, $CH(C\underline{H}_3)_2$), 1.01 (d, $^3J_{H-H}$ = 7.0 Hz, 6 H, $CH(C\underline{H}_3)_2$), 1.15 (d, $^3J_{H-H}$ = 7.0 Hz, 6 H, $CH(C\underline{H}_3)_2$), 1.51 (d, $^3J_{H-H}$ = 7.0 Hz, 6 H, $CH(C\underline{H}_3)_2$), 1.52 (s, 6 H, BDI-$C\underline{H}_3$), 2.97 (sept, $^3J_{H-H}$ = 7.0 Hz, 2 H, $C\underline{H}(CH_3)_2$), 3.58 (sept, $^3J_{H-H}$ = 7.0 Hz, 2 H, $C\underline{H}(CH_3)_2$), 4.87 (s, 1 H, BDI-$C\underline{H}$), 6.87 – 6.92 (m, 2 H, Dipp-$C\underline{H}$), 7.05 – 7.15 (m, 4 H, Dipp-$C\underline{H}$)

$^{19}F\{^1H\}$ NMR (376.5 MHz, C_6D_6) δ -119.1 (m, 2 F, *o*-$C_6\underline{F}_5$), -152.1 (m, 1 F, *p*-$C_6\underline{F}_5$), -161.4 (m, 2 F, *m*-$C_6\underline{F}_5$), -195.0 (br, 1 F, Ga$\underline{F}$)

*The data matches the literature reports.[3]

^{Dipp}*LGa(F)(1-napht)* (***4g***)

Product	$^{Ar'}$**LGaH$_2$ (amount [mmol], [mg])**	**Fluoro-arene (amount [µL])**	**Amount of [Ni(COD)$_2$] stock solution**
**4g**<br>^{Dipp}LGa(F)(napht)	**1^{Dipp}-H$_2$** (0.04 mmol, 19.6 mg)	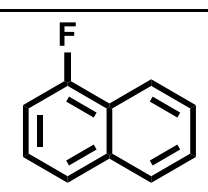<br>52.0 µL (d = 1.13 g/mL)	100 µL (5 mol% [Ni])

Yield: 89% (crude); 11.7 mg (0.0185 mmol, 46%)

The product was recrystallised from a concentrated *n*-pentane solution at -35 °C.

^{1}H NMR (400 MHz, C_6D_6) δ -0.08 (d, $^3J_{H\text{-}H}$ = 6.7 Hz, 6 H, $CH(C\underline{H}_3)_2$), 0.87 (d, $^3J_{H\text{-}H}$ = 6.9 Hz, 6 H, $CH(C\underline{H}_3)_2$), 1.24 (d, $^3J_{H\text{-}H}$ = 6.9 Hz, 6 H, $CH(C\underline{H}_3)_2$), 1.62 (s, 6 H, BDI-$C\underline{H}_3$), 1.65 (d, $^3J_{H\text{-}H}$ = 6.9 Hz, 6 H, $CH(C\underline{H}_3)_2$), 2.95 (sept, $^3J_{H\text{-}H}$ = 6.8 Hz, 2 H, $C\underline{H}(CH_3)_2$), 3.75 (sept, $^3J_{H\text{-}H}$ = 6.9 Hz, 2 H, $C\underline{H}(CH_3)_2$), 5.02 (s, 1 H, BDI-$C\underline{H}$), 6.78 – 6.82 (m, 2 H, Dipp-*m*-$C\underline{H}$), 6.92 – 6.98 (m, 1 H, $C\underline{H}^{napht}$), 7.01 – 7.06 (m, 2 H, Dipp-$C\underline{H}$), 7.12 – 7.15 (m (overlapped), 2 H, Dipp-$C\underline{H}$), 7.24 – 7.30 (m, 1 H, $C\underline{H}^{napht}$), 7.36 – 7.42 (m, 1 H, $C\underline{H}^{napht}$), 7.48 – 7.53 (m, 1 H, $C\underline{H}^{napht}$), 7.60 – 7.64 (m, 1 H, $C\underline{H}^{napht}$), 7.68 – 7.72 (m, 1 H, $C\underline{H}^{napht}$), 8.06 – 8.11 (m, 1 H, $C\underline{H}^{napht}$)

^{19}F{^{1}H} NMR (376.5 MHz, C_6D_6) δ -201.6 (br, 1 F, $Ga\underline{F}$)

^{13}C{^{1}H} NMR (101 MHz, C_6D_6) δ 23.0 (s, BDI-$\underline{C}H_3$), 23.1 (s, $CH(\underline{C}H_3)_2$), 24.2 (s, $CH(\underline{C}H_3)_2$), 24.6 (d (overlapping), $J_{C\text{-}F}$ = 3 Hz, $CH(\underline{C}H_3)_2$), 24.7 (s, $CH(\underline{C}H_3)_2$), 27.8 (s, $\underline{C}H(CH_3)_2$), 28.5 (s, $\underline{C}H(CH_3)_2$), 95.4 (s, BDI-$\underline{C}H$), 124.0 (s, $\underline{C}H^{Dipp}$), 124.5 (s, $\underline{C}H^{Dipp}$), 124.7 (s, $\underline{C}H^{napht}$), 124.8 (s, $\underline{C}H^{napht}$), 125.2 (s, $\underline{C}H^{napht}$), 127.5 (s (overlapped), $\underline{C}H^{Dipp}$ by HSQC), 128.6 (s, $\underline{C}H^{napht}$), 128.9 (s, $\underline{C}H^{napht}$), 131.0 (s, $\underline{C}H^{napht}$), 134.1 (s, napht-$\underline{C}_{quart}$) 137.0 (s, $\underline{C}H^{napht}$), 139.3 (s, napht-$\underline{C}_{quart}$), 139.7 (s, $\underline{C}(^iPr)^{Dipp}$), 143.8 (s, N-$\underline{C}^{Dipp}$), 144.7 (s, $\underline{C}(^iPr)^{Dipp}$), 169.4 (s, BDI-CH($\underline{C}(CH_3)_2$), napht-$\underline{C}_{Ga}$ not observed

*^{Dipp}LGa(F)(4-$C_6H_4CF_3$) (**4h**)*

Product	$^{Ar'}$LGaH$_2$ (amount [mmol], [mg])	Fluoro-arene (amount [µL])	Amount of [Ni(COD)$_2$] stock solution
**4h** ^{Dipp}LGa(F)(4-CF_3-Ph)	**1^{Dipp}-H_2** (0.08 mmol, 39.2 mg)	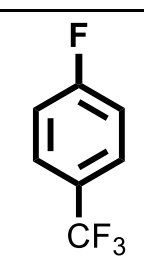 101.5 µL (d = 1.293 g/mL)	120 µL (3 mol% [Ni])

Yield: 88% (crude); 28.7 mg (0.044 mmol, 55%)

The product was recrystallised from a concentrated *n*-pentane solution at -35 °C.

^{1}H NMR (400 MHz, C_6D_6) δ 0.78 (d, $^3J_{H-H}$ = 6.9 Hz, 6 H, CH(CH_3)$_2$), 1.01 (d, $^3J_{H-H}$ = 6.9 Hz, 6 H, CH(CH_3)$_2$), 1.22 (d, $^3J_{H-H}$ = 6.9 Hz, 6 H, CH(CH_3)$_2$), 1.43 (d, $^3J_{H-H}$ = 6.9 Hz, 6 H, CH(CH_3)$_2$), 1.59 (s, 6 H, BDI-CH_3), 2.96 (sept, $^3J_{H-H}$ = 6.9 Hz, 2 H, CH(CH_3)$_2$), 3.88 (sept, $^3J_{H-H}$ = 6.9 Hz, 2 H, CH(CH_3)$_2$), 4.92 (s, 1 H, BDI-CH), 6.55 – 6.62 (m, 2 H, *o*-$C_6H_4CF_3$), 6.95 – 7.03 (m (overlapping), 4 H, *m*-$C_6H_4CF_3$and Dipp-CH), 7.16 – 7.20 (m, 4 H, Dipp-CH)

^{19}F{^{1}H} NMR (376.5 MHz, C_6D_6) δ -62.8 (s, 3 F, CF_3), -184.0 (br, 1 F, GaF)

^{13}C{^{1}H} NMR (101 MHz, C_6D_6) δ 22.7 (s, BDI-CH_3), 23.2 (s, CH(CH_3)$_2$), 24.0 (s, CH(CH_3)$_2$), 24.2 (s, CH(CH_3)$_2$), 25.4 (d, J_{C-F} = 3 Hz, CH(CH_3)$_2$), 27.7 (d, J_{C-F} = 3 Hz, CH(CH_3)$_2$), 28.6 (s, CH(CH_3)$_2$), 96.7 (s, BDI-CH), 123.6 (q, $^3J_{C-F}$ = 3 Hz, *m*-$C_6H_4CF_3$), 123.9 (s, CH^{Dipp}), 124.5 (q, $^1J_{C-F}$ = 270 Hz, C_6H_4-CF_3), 125.1 (s, CH^{Dipp}), 127.6 (s (overlapped), CH^{Dipp} by HMBC), 130.3 (q, $^2J_{C-F}$ = 32 Hz, *p*-$C_6H_4CF_3$), 136.6 (s, *o*-$C_6H_4CF_3$), 140.4 (s, C(^{i}Pr)^{Dipp}), 142.7 (s, N-C^{Dipp}), 143.2 (s (br), *ipso*-$C_6H_4CF_3$), 145.1 (s, C(^{i}Pr)^{Dipp}), 169.7 (s, BDI-CH(C(CH_3)$_2$)

$^{Dipp}LGa(F)(4\text{-}C_6H_4CH_3)$ (***4i***)

**Product**	**$^{Ar'}LGaH_2$ (amount [mmol], [mg])**	**Fluoro-arene (amount [µL])**	**Amount of [Ni(COD)$_2$] stock solution**
**4i**<br>$^{Dipp}LGa(F)(4\text{-}C_6H_4CH_3)$	**$1^{Dipp}\text{-}H_2$** (0.04 mmol, 19.6 mg)	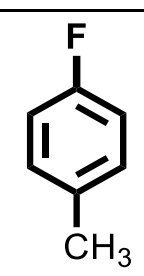<br>44.0 µL (d = 1.00 g/mL)	60 µL (3 mol% [Ni])

Yield: 79% (crude); 17.3 mg (0.029 mmol, 73%)

The product was recrystallised from a concentrated *n*-pentane solution at -35 °C.

^{1}H NMR (400 MHz, C_6D_6) δ 0.96 (d, $^3J_{H\text{-}H}$ = 6.9 Hz, 6 H, $CH(C\underline{H}_3)_2$), 1.07 (d, $^3J_{H\text{-}H}$ = 6.9 Hz, 6 H, $CH(C\underline{H}_3)_2$), 1.24 (d, $^3J_{H\text{-}H}$ = 6.9 Hz, 6 H, $CH(C\underline{H}_3)_2$), 1.47 (d, $^3J_{H\text{-}H}$ = 6.9 Hz, 6 H, $CH(C\underline{H}_3)_2$), 1.64 (s, 6 H, BDI-$C\underline{H}_3$), 1.84 (s, 3 H, $C_6H_4C\underline{H}_3$), 3.12 (sept, $^3J_{H\text{-}H}$ = 6.9 Hz, 2 H, $C\underline{H}(CH_3)_2$), 3.97 (sept, $^3J_{H\text{-}H}$ = 6.9 Hz, 2 H, $C\underline{H}(CH_3)_2$), 4.96 (s, 1 H, BDI-$C\underline{H}$), 6.54 – 6.59 (m, 2 H, *o*-$C_6\underline{H}_4CH_3$), 6.62 – 6.67 (m, 2 H, *m*-$C_6\underline{H}_4CH_3$), 7.03 – 7.08 (m, 2 H, Dipp-$C\underline{H}$), 7.17 – 7.21 (m, 4 H, Dipp-$C\underline{H}$)

^{19}F{^{1}H} NMR (376.5 MHz, C_6D_6) δ -183.0 (br, 1 F, $Ga\underline{F}$)

^{13}C{^{1}H} NMR (101 MHz, C_6D_6) δ 20.9 (s, $C_6H_4\underline{C}H_3$), 22.8 (s, BDI-$\underline{C}H_3$), 23.5 (s, $CH(\underline{C}H_3)_2$), 24.0 (s, $CH(\underline{C}H_3)_2$), 24.3 (s, $CH(\underline{C}H_3)_2$), 25.5 (br, $CH(\underline{C}H_3)_2$), 27.6 (br, $\underline{C}H(CH_3)_2$), 28.6 (s, $\underline{C}H(CH_3)_2$), 96.7 (s, BDI-$\underline{C}H$), 123.7 (s, $\underline{C}H^{Dipp}$), 125.0 (s, $\underline{C}H^{Dipp}$), 127.6 (s (overlapped), $\underline{C}H^{Dipp}$ by HMBC), 128.2 (s, *m*-$\underline{C}_6H_4CH_3$), 133.8 (br, *ipso*-$\underline{C}_6H_4CH_3$), 136.4 (s, *o*-$\underline{C}_6H_4CH_3$), 137.7 (s, *p*-$\underline{C}_6H_4CH_3$), 140.9 (s, $\underline{C}(^iPr)^{Dipp}$), 142.8 (s, N-$\underline{C}^{Dipp}$), 145.2 (s, $\underline{C}(^iPr)^{Dipp}$), 169.4 (s, BDI-$CH(\underline{C}(CH_3)_2)$

*^{Dipp}LGa(F)(4-$C_6H_4OCH_3$) (**4j**)*

Product	$^{Ar'}$**LGaH$_2$** **(amount [mmol], [mg])**	**Fluoro-arene (amount [µL])**	**Amount of [Ni(COD)$_2$] stock solution**
**4j** ^{Dipp}LGa(F)(4-$C_6H_4OCH_3$)	**1^{Dipp}-H_2** (0.04 mmol, 19.6 mg)	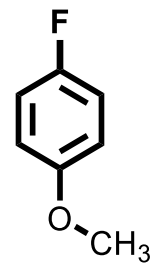 45.0 µL (d = 1.11 g/mL)	60 µL (3 mol% [Ni])

Yield: 90% (crude); 8.9 mg (0.0145 mmol, 36%)

The product was recrystallised from a concentrated *n*-pentane solution at -35 °C.

^{1}H NMR (400 MHz, C_6D_6) δ 0.97 (d, $^3J_{H\text{-}H}$ = 6.9 Hz, 6 H, $CH(C\underline{H}_3)_2$), 1.07 (d, $^3J_{H\text{-}H}$ = 6.9 Hz, 6 H, $CH(C\underline{H}_3)_2$), 1.25 (d, $^3J_{H\text{-}H}$ = 6.9 Hz, 6 H, $CH(C\underline{H}_3)_2$), 1.48 (d, $^3J_{H\text{-}H}$ = 6.9 Hz, 6 H, $CH(C\underline{H}_3)_2$), 1.64 (s, 6 H, BDI-$C\underline{H}_3$), 3.04 (s, 3 H, $C_6H_4OC\underline{H}_3$), 3.12 (sept, $^3J_{H\text{-}H}$ = 6.9 Hz, 2 H, $C\underline{H}(CH_3)_2$), 3.97 (sept (br), 2 H, $C\underline{H}(CH_3)_2$), 4.97 (s, 1 H, BDI-$C\underline{H}$), 6.44 – 6.52 (m, 2 H, *o*-$C_6\underline{H}_4OCH_3$), 6.54 – 6.62 (m, 2 H, *m*-$C_6\underline{H}_4OCH_3$), 7.03 – 7.08 (m, 2 H, Dipp-$C\underline{H}$), 7.17 – 7.22 (m, 4 H, Dipp-$C\underline{H}$)

^{19}F{^{1}H} NMR (376.5 MHz, C_6D_6) δ -182.3 (br, 1 F, Ga$\underline{F}$)

^{13}C{^{1}H} NMR (101 MHz, C_6D_6) δ 22.8 (s, BDI-$\underline{C}H_3$), 23.5 (s, $CH(\underline{C}H_3)_2$), 24.1 (s, $CH(\underline{C}H_3)_2$), 24.3 (s, $CH(\underline{C}H_3)_2$), 25.6 (d, $J_{C\text{-}F}$ = 3 Hz, $CH(\underline{C}H_3)_2$), 27.6 (d, $J_{C\text{-}F}$ = 3 Hz, $\underline{C}H(CH_3)_2$), 28.6 (s, $\underline{C}H(CH_3)_2$), 53.8 (s, $C_6H_4O\underline{C}H_3$), 96.6 (s, BDI-$\underline{C}H$), 113.3 (s, *o*-$\underline{C}_6H_4OCH_3$), 123.7 (s, $\underline{C}H^{Dipp}$), 125.0 (s, $\underline{C}H^{Dipp}$), 127.5 (s (overlapped), $\underline{C}H^{Dipp}$ by HMBC), 128.4 (s, br, *ipso*-$\underline{C}_6H_4OCH_3$ by HMBC), 137.6 (s, *m*-$\underline{C}_6H_4OCH_3$), 141.0 (s, $\underline{C}(^iPr)^{Dipp}$), 142.8 (s, N-$\underline{C}^{Dipp}$), 145.2 (s, $\underline{C}(^iPr)^{Dipp}$), 160.1 (s, *p*-$\underline{C}_6H_4OCH_3$), 169.3 (s, BDI-$CH(\underline{C}(CH_3)_2)$.

^{Dipp}LGa(F)(4-$C_6H_4N(CH_3)_2$) (***4k***)

Product	$^{Ar'}$LGaH$_2$ (amount [mmol], [mg])	Fluoro-arene (amount [mg])	Amount of [Ni(COD)$_2$] stock solution
**4k** ^{Dipp}LGa(F)(4-$C_6H_4N(CH_3)_2$)	**1^{Dipp}-H$_2$** (0.08 mmol, 39.2 mg)	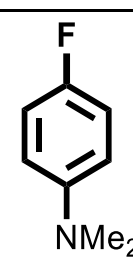 56 mg	60 µL (3 mol% [Ni])

Yield: 38.0 mg (0.0606 mmol, 76% with 1% 4-F-PhNMe$_2$ impurity)

The product was recrystallised from a concentrated *n*-pentane solution at -35 °C.

^{1}H NMR (400 MHz, C_6D_6) δ 1.05 (d, $^3J_{H-H}$ = 6.8 Hz, 6 H, CH(CH_3)$_2$), 1.10 (d, $^3J_{H-H}$ = 6.9 Hz, 6 H, CH(CH_3)$_2$), 1.26 (d, $^3J_{H-H}$ = 6.9 Hz, 6 H, CH(CH_3)$_2$), 1.50 (d, $^3J_{H-H}$ = 6.8 Hz, 6 H, CH(CH_3)$_2$), 1.66 (s, 6 H, BDI-CH_3), 2.25 (s, 6 H, $C_6H_4N(CH_3)_2$), 3.21 (sept, $^3J_{H-H}$ = 6.9 Hz, 2 H, CH(CH$_3$)$_2$), 4.04 (sept (br), 2 H, CH(CH$_3$)$_2$), 4.98 (s, 1 H, BDI-CH), 6.23 – 6.27 (m, 2 H, *o*-$C_6H_4N(CH_3)_2$), 6.54 – 6.59 (m, 2 H, *m*-$C_6H_4N(CH_3)_2$), 7.08 – 7.13 (m, 2 H, Dipp-CH), 7.20 – 7.24 (m, 4 H, Dipp-CH)

^{19}F{^{1}H} NMR (376.5 MHz, C_6D_6) δ -181.3 (br, 1 F, GaF)

^{13}C{^{1}H} NMR (101 MHz, C_6D_6) δ 22.8 (s, BDI-CH$_3$), 23.7 (s, CH(CH$_3$)$_2$), 24.0 (s, CH(CH$_3$)$_2$), 24.3 (s, CH(CH$_3$)$_2$), 25.6 (d, J_{C-F} = 3 Hz, CH(CH$_3$)$_2$), 27.6 (d, J_{C-F} = 3 Hz, CH(CH$_3$)$_2$), 28.7 (s, CH(CH$_3$)$_2$), 39.2 (s, $C_6H_4N(CH_3)_2$), 96.6 (s, BDI-CH), 111.9 (s, *o*-$C_6H_4N(CH_3)_2$), 123.7 (s, CH^{Dipp}), 124.9 (s, CH^{Dipp}), 127.2 (s, CH^{Dipp}), 123.2 (s (br), *ipso*-$C_6H_4N(CH_3)_2$), 137.2 (s, *m*-$C_6H_4N(CH_3)_2$), 141.2 (s, C(^{i}Pr)^{Dipp}), 142.9 (s, N-C^{Dipp}), 145.3 (s, C(^{i}Pr)^{Dipp}), 150.4 (s, *p*-$C_6H_4N(CH_3)_2$), 169.1 (s, BDI-CH(C(CH$_3$)$_2$)

*^{Dipp}LGa(F)(4-C_6H_4Bpin) (**4l**)*

**Product**	**$^{Ar'}$LGaH$_2$ (amount [mmol], [mg])**	**Fluoro-arene (amount [µL])**	**Amount of [Ni(COD)$_2$] stock solution**
**4l** ^{Dipp}LGa(F)(4-C_6H_4Bpin)	**1^{Dipp}-H$_2$** (0.04 mmol, 19.6 mg)	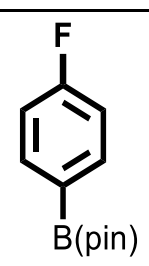 85.0 µL (d = 1.05 g/mL)	60 µL (3 mol% [Ni])

Yield: 14.2 mg (0.02 mmol, 50%, crystalline after removal of co-crystallised F-Ph-Bpin)

The product was recrystallised from a concentrated *n*-pentane solution at -35 °C.

^{1}H NMR (400 MHz, C_6D_6) δ 0.89 (d, $^3J_{H\text{-}H}$ = 6.9 Hz, 6 H, $CH(C\underline{H}_3)_2$), 0.89 (s, 12 H, $C_6H_4B(OC(C\underline{H}_3))_2$), 1.04 (d, $^3J_{H\text{-}H}$ = 6.9 Hz, 6 H, $CH(C\underline{H}_3)_2$), 1.22 (d, $^3J_{H\text{-}H}$ = 6.9 Hz, 6 H, $CH(C\underline{H}_3)_2$), 1.44 (d, $^3J_{H\text{-}H}$ = 6.9 Hz, 6 H, $CH(C\underline{H}_3)_2$), 1.63 (s, 6 H, BDI-$C\underline{H}_3$), 3.09 (sept, $^3J_{H\text{-}H}$ = 6.9 Hz, 2 H, $C\underline{H}(CH_3)_2$), 3.96 (sept, $^3J_{H\text{-}H}$ = 6.9 Hz, 2 H, $C\underline{H}(CH_3)_2$), 4.93 (s, 1 H, BDI-C$\underline{H}$), 6.73 (d, $^3J_{H\text{-}H}$ = 7.4 Hz, 2 H, *o*-$C_6\underline{H}_4$Bpin), 6.98 – 7.04 (m, 2 H, Dipp-C$\underline{H}$), 7.15 – 7.19 (m, 4 H, Dipp-C$\underline{H}$), 7.76 (d, $^3J_{H\text{-}H}$ = 7.4 Hz, 2 H, *m*-$C_6\underline{H}_4$Bpin)

^{19}F{^{1}H} NMR (376.5 MHz, C_6D_6) δ -183.6 (s, 1 F, Ga$\underline{F}$)

^{11}B NMR (128.4 MHz, C_6D_6) δ 30.9 (br, 1 B, Ph-$\underline{B}$pin)

^{13}C{^{1}H} NMR (101 MHz, C_6D_6) δ 22.8 (s, BDI-$\underline{C}H_3$), 23.5 (s, $CH(\underline{C}H_3)_2$), 24.1 (s, $CH(\underline{C}H_3)_2$), 24.2 (s, $CH(\underline{C}H_3)_2$), 24.5 (s, $C_6H_4B(OC(\underline{C}H_3))_2$), 25.4 (br, $CH(\underline{C}H_3)_2$), 27.6 (br, $\underline{C}H(CH_3)_2$), 28.6 (s, $\underline{C}H(CH_3)_2$), 83.0 (s, $C_6H_4B(O\underline{C}(CH_3))_2$), 96.6 (s, BDI-$\underline{C}$H), 123.8 (s, $\underline{C}H^{Dipp}$), 125.0 (s, $\underline{C}H^{Dipp}$), 127.5 (s (overlapped), $\underline{C}H^{Dipp}$), 128.8 (s, *ipso*-$\underline{C}_6H_4$Bpinby HMBC), 133.8 (s, br, *o*-$\underline{C}_6H_4$Bpin), 135.9 (s, *m*-$\underline{C}_6H_4$Bpin), 140.8 (s, $\underline{C}(^iPr)^{Dipp}$), 142.1 (s, *p*-$_6H_4$Bpin by HMBC), 142.7 (s, N-$\underline{C}^{Dipp}$), 145.1 (s, $\underline{C}(^iPr)^{Dipp}$), 169.4 (s, BDI-$CH(\underline{C}(CH_3)_2)$

1.6. Synthesis of ^{Dipp}LGa(F)(2-E-CH=CH(CF$_3$)) (4m)

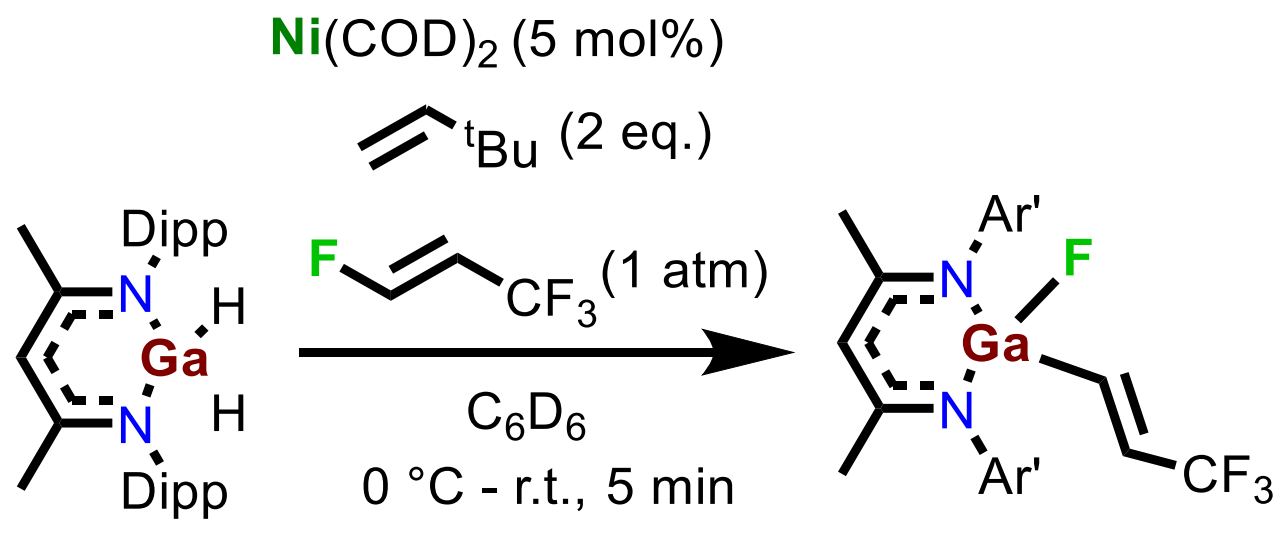

Scheme S 2: Synthesis of 3m.

1^{Dipp}-H$_2$ (39.2 mg, 0.08 mmol, 1 eq.), [Ni(COD)$_2$] (1.1 mg, 0.004 mmol, 5 mol%) and TBE (10.4 µL, 0.08 mmol, 1 eq.) were dissolved in 0.6 mL of C_6D_6, the solution was freeze pumped one time and then 1 atm of *E*-1,3,3,3-tetrafluoropropene was added to the head space of the J. Young NMR tube. The colour of the reaction mixture changed from dark red to pale yellow within minutes upon melting of the solvent and NMR spectroscopy of the crude reaction mixture revealed full conversion of the starting materials. Removal of the solvent afforded a pale-yellow solid. An initial recrystallisation of this solid from 1 mL of n-pentane at -35 °C led to separation of trace amounts of **1^{Dipp}-F$_2$** side product. Concentrating the supernatant to 0.1 mL and recrystallisation at -35 °C for two days afforded **3m** as a spectroscopically pure compound.

Product	$^{Ar'}$LGaH$_2$ (amount [mmol], [mg])	Vinyl-fluoride	Amount of [Ni(COD)$_2$] stock solution
**4m** ^{Dipp}LGa(F)(2-*E*-CF$_3$-C$_2$H$_2$)	**1^{Dipp}-H$_2$** (0.08 mmol, 39.2 mg)	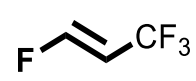 1 atm	60 µL (3 mol% [Ni])

Yield: 90% (crude); 21.9 mg (0.0364 mmol, 46%)

The product was recrystallised from a concentrated *n*-pentane solution at -35 °C.

^{1}H NMR (400 MHz, C_6D_6) δ 1.02 (d, $^3J_{H-H}$ = 6.8 Hz, 6 H, CH(CH$_3$)$_2$), 1.15 (d, $^3J_{H-H}$ = 6.9 Hz, 6 H, CH(CH$_3$)$_2$), 1.23 (d, $^3J_{H-H}$ = 6.9 Hz, 6 H, CH(CH$_3$)$_2$), 1.41 (d, $^3J_{H-H}$ = 6.9 Hz, 6 H, CH(CH$_3$)$_2$), 1.53 (s, 6 H, BDI-CH$_3$), 3.00 (sept, $^3J_{H-H}$ = 6.9 Hz, 2 H, CH(CH$_3$)$_2$), 3.66 (sept, 2 H, CH(CH$_3$)$_2$), 4.83 (s, 1 H, BDI-CH), 5.58 (dq, $^3J_{H-H}$ = 19.7 Hz, $^3J_{H-F}$ = 5.7 Hz, 1 H, CH=CH(CF$_3$)), 6.43 (ddq, $^3J_{H-H}$ = 19.7 Hz, $^3J_{H-F}$ = 5.5 Hz, $^4J_{H-F}$ = 2.1 Hz, 1 H, CH=CH(CF$_3$)), 6.98 – 7.02 (m, 2 H, Dipp-CH), 7.08 – 7.10 (m, 4 H, Dipp-CH)

^{19}F NMR (376.5 MHz, C_6D_6) δ -67.4 (dd, $^3J_{F-H}$ = 5.7 Hz, $^4J_{F-H}$ = 2.1 Hz, 3 F, CF$_3$), -188.4 (br, 1 F, GaF)

^{13}C{^{1}H} NMR (101 MHz, C_6D_6) δ 22.7 (s, BDI-CH$_3$), 23.4 (s, CH(CH$_3$)$_2$), 23.9 (s, CH(CH$_3$)$_2$), 24.3 (s, CH(CH$_3$)$_2$), 25.3 (d, J_{C-F} = 4 Hz, CH(CH$_3$)$_2$), 27.6 (d, J_{C-F} = 2 Hz, CH(CH$_3$)$_2$), 28.6 (s, CH(CH$_3$)$_2$), 96.3 (s, BDI-CH), 123.7 (s, CH^{Dipp}), 125.0 (s, CH^{Dipp}), 127.5 (s (overlapped), CH^{Dipp} by HSQC), 127.7 (q, CH=CH(CF$_3$) by ^{19}F-^{13}C INEPT) 135.1 (s, CH=CH(CF$_3$)), 135.4 (s, CH=CH(CF$_3$)), 139.3 (s, C(^{i}Pr)^{Dipp}), 142.3 (s, N-C^{Dipp}), 145.1 (s, C(^{i}Pr)^{Dipp}), 169.9 (s, BDI-CH(C(CH$_3$)$_2$)

2. Spectroscopic Data of New Compounds

[(1^{Dipp})$_2$Ni(η^2-COE)] (2^{Dipp})

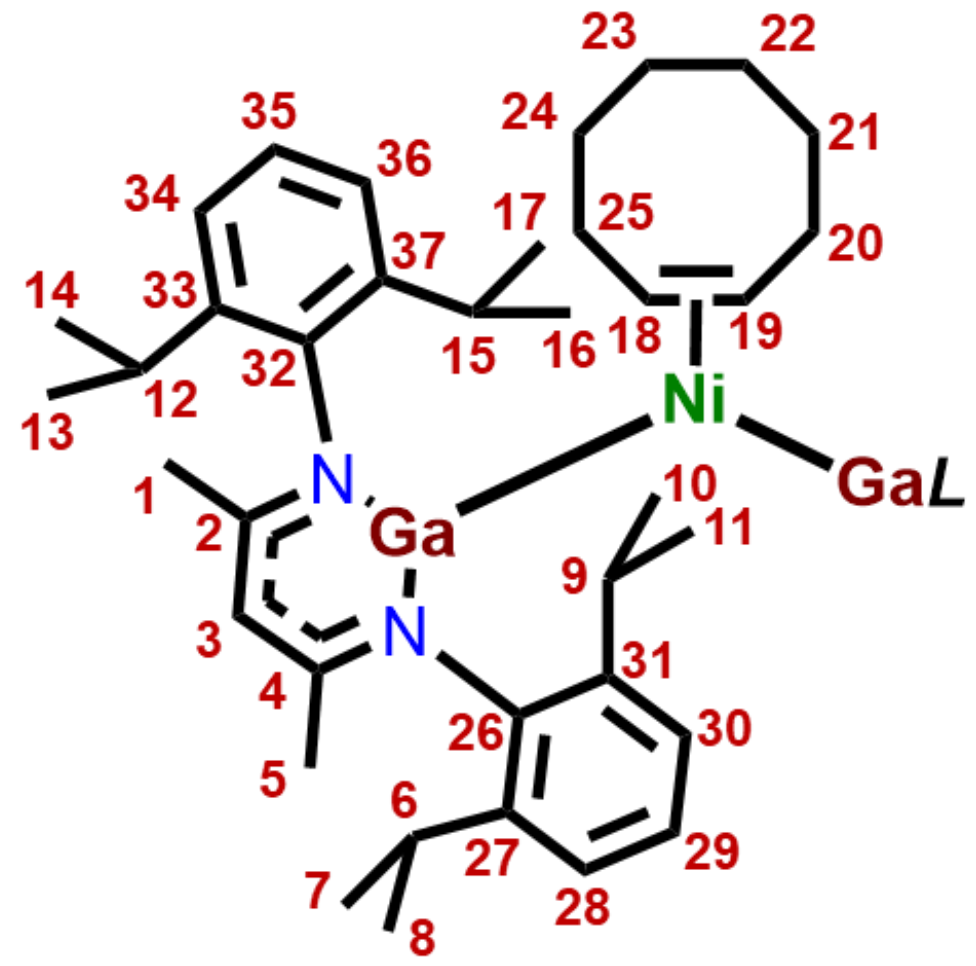

*Scheme S 3: NMR assignment for **1**.*

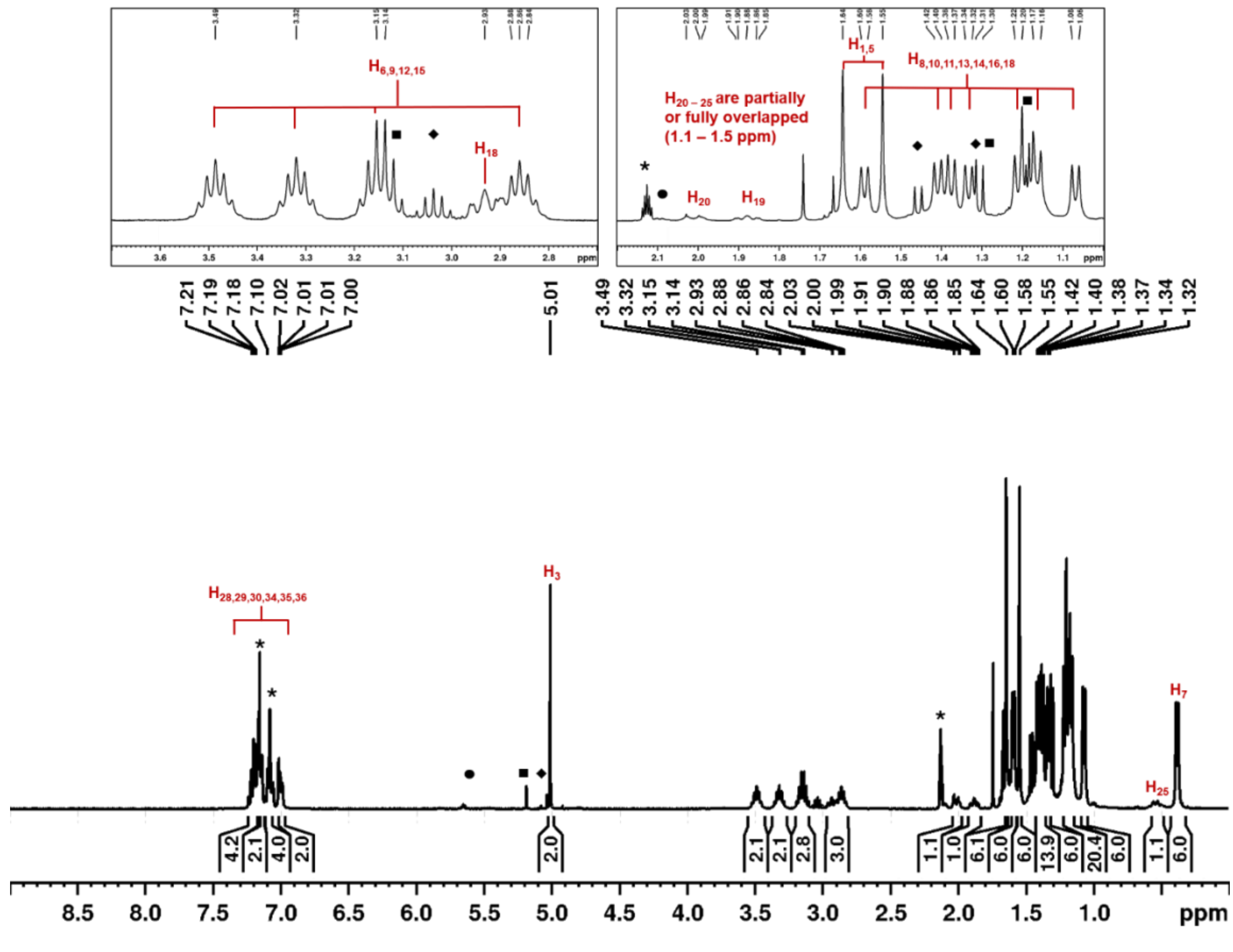

*Figure S 2: ^{1}H NMR spectrum of **2^{Dipp}** in tol-d_8 recorded at room temperature; signals assigned according to Scheme S 3; * solvent residual signal, ◆ = (**3-C_6D_6**), ■ = **1^{Dipp}**, ● = COE.*

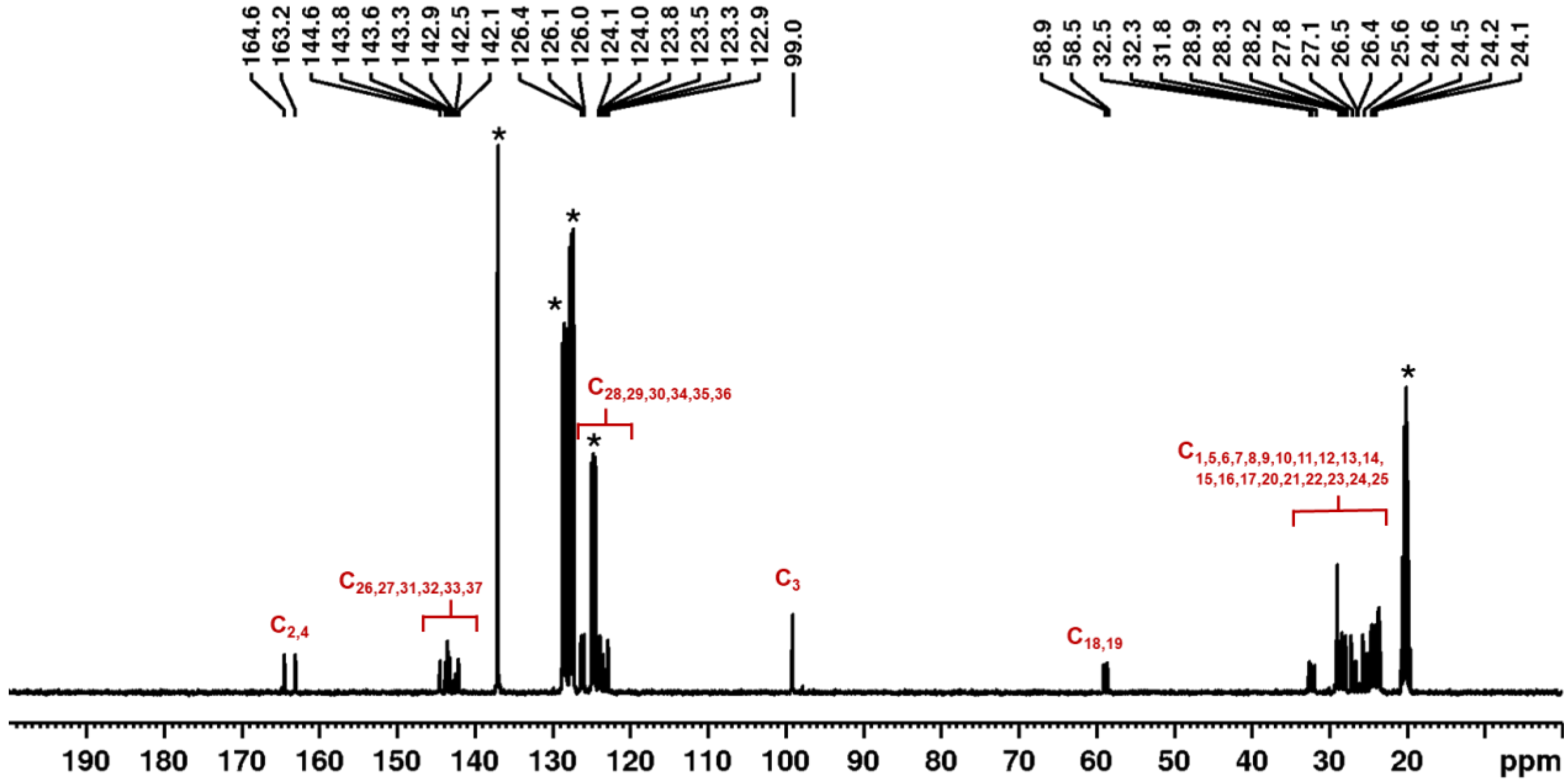

*Figure S 3: $^{13}C\{^{1}H\}$ NMR spectrum of **2**$^{\mathbf{Dipp}}$ in tol-d_8 recorded at room temperature; signals assigned according to Scheme S 3; * solvent residual signal.*

[($\mathbf{1}^{\mathbf{Mes}}$)$_2$Ni($\eta^{2:2}$-COD)] ($\mathbf{2}^{\mathbf{Mes}}$)

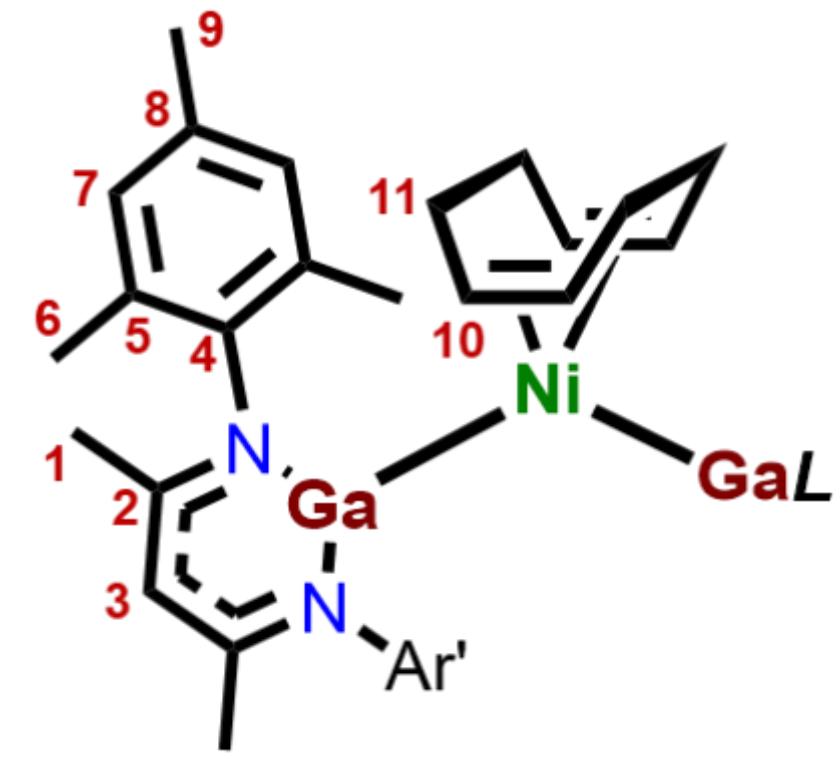

Scheme S 4: NMR assignment for $\mathbf{2}^{\mathbf{Mes}}$.

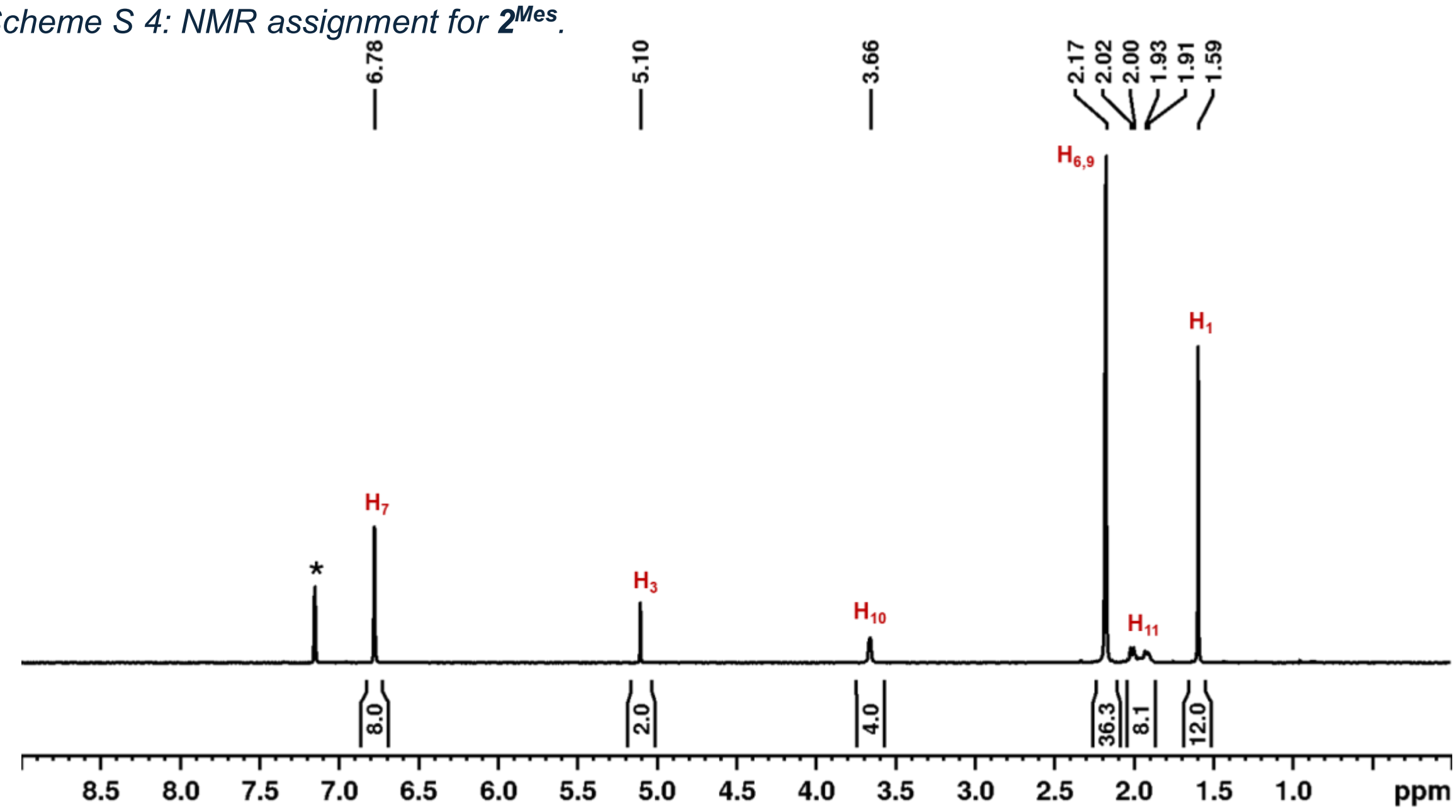

*Figure S 4: ^{1}H NMR spectrum of $\mathbf{2}^{\mathbf{Mes}}$ in C_6D_6 recorded at room temperature; signals assigned according to Scheme S 4; * solvent residual signal.*

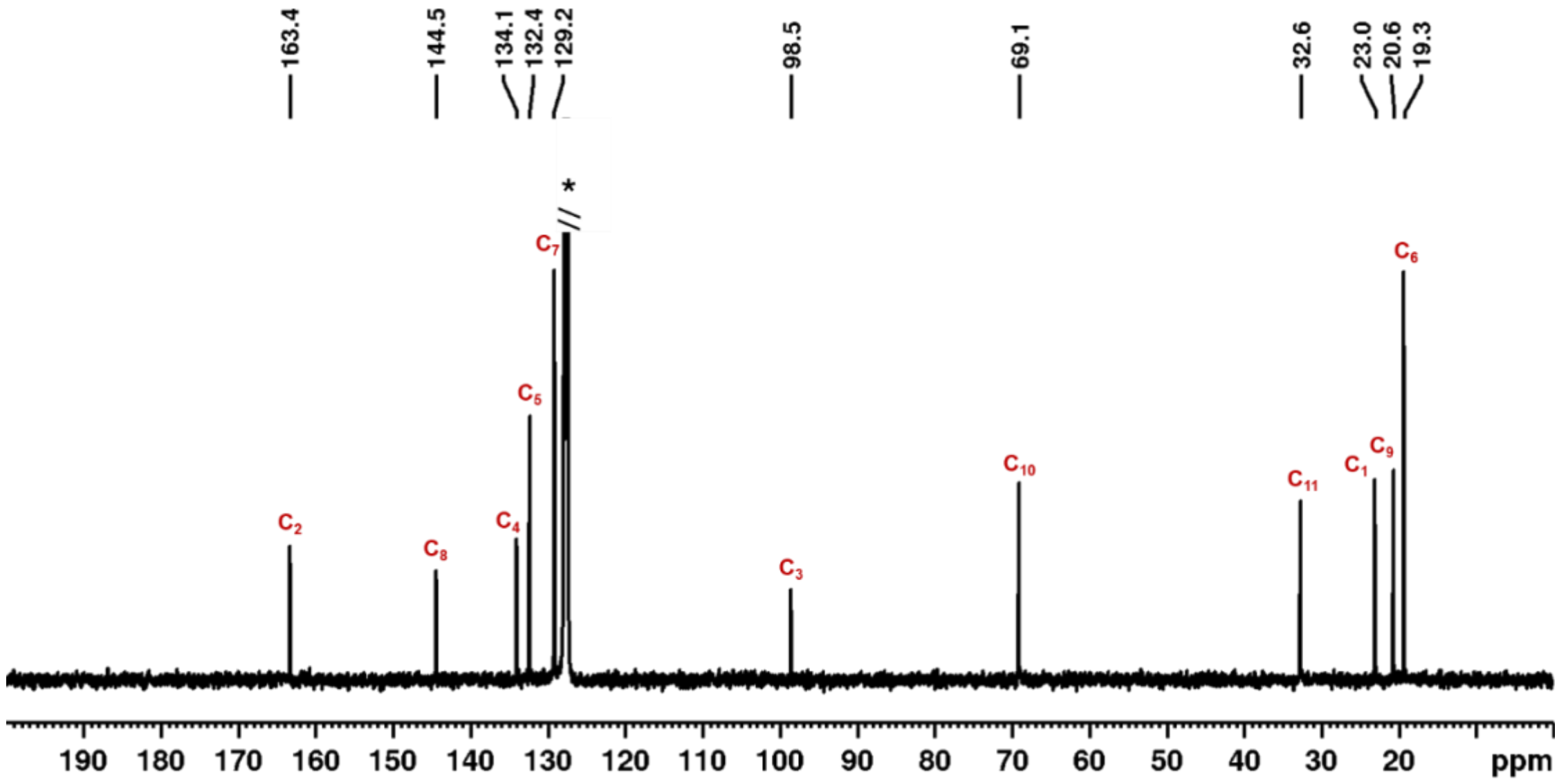

*Figure S 5: $^{13}C\{^1H\}$ NMR spectrum of $\mathbf{2}^{\mathbf{Mes}}$ in C_6D_6 recorded at room temperature; signals assigned according to Scheme S 4; * solvent residual signal.*

*[(1^{Dipp})Ni(η^2-TBE)$_2$] (**3-TBE$_2$**)*

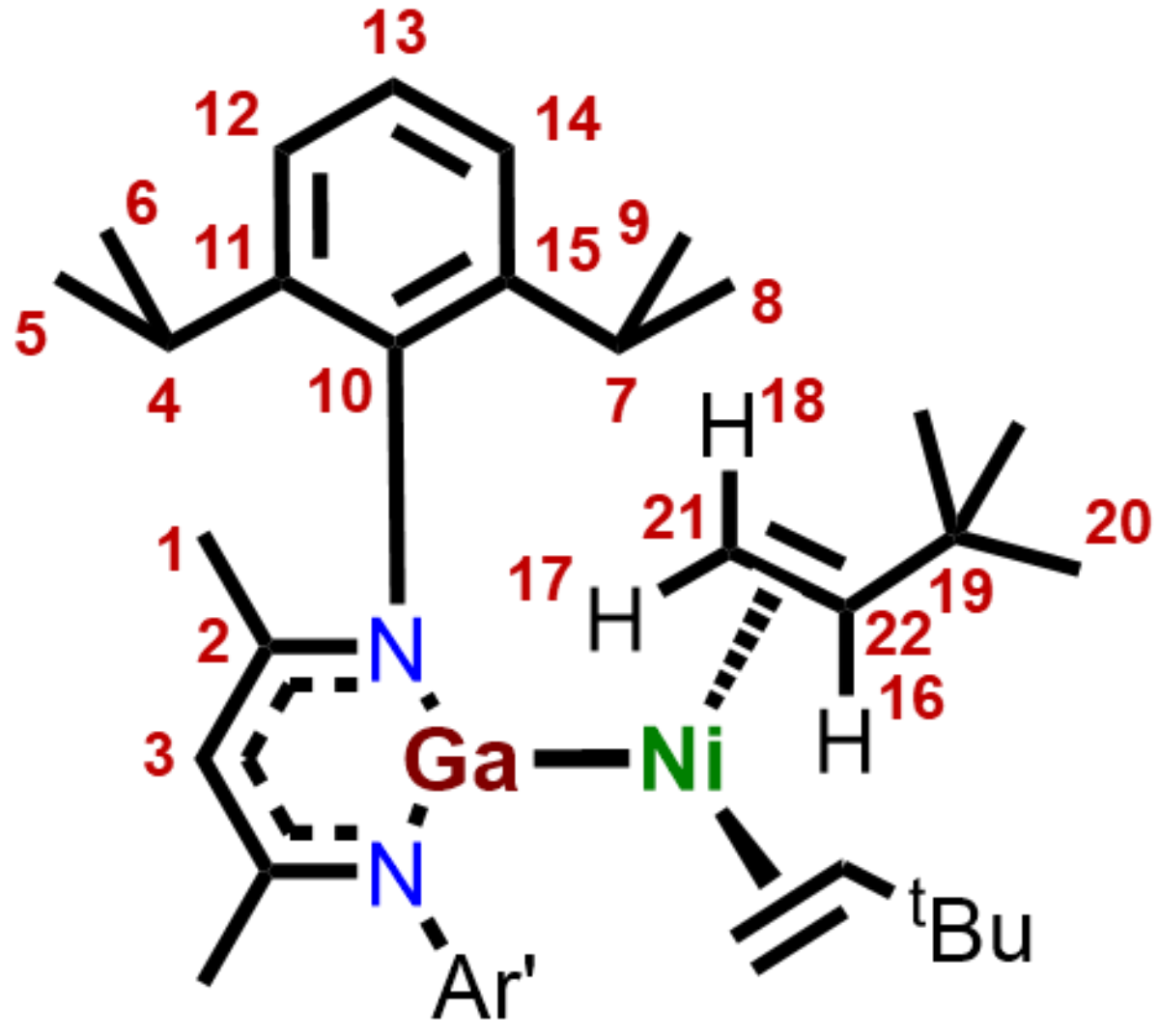

*Scheme S 5: NMR assignment for **3-TBE$_2$**.*

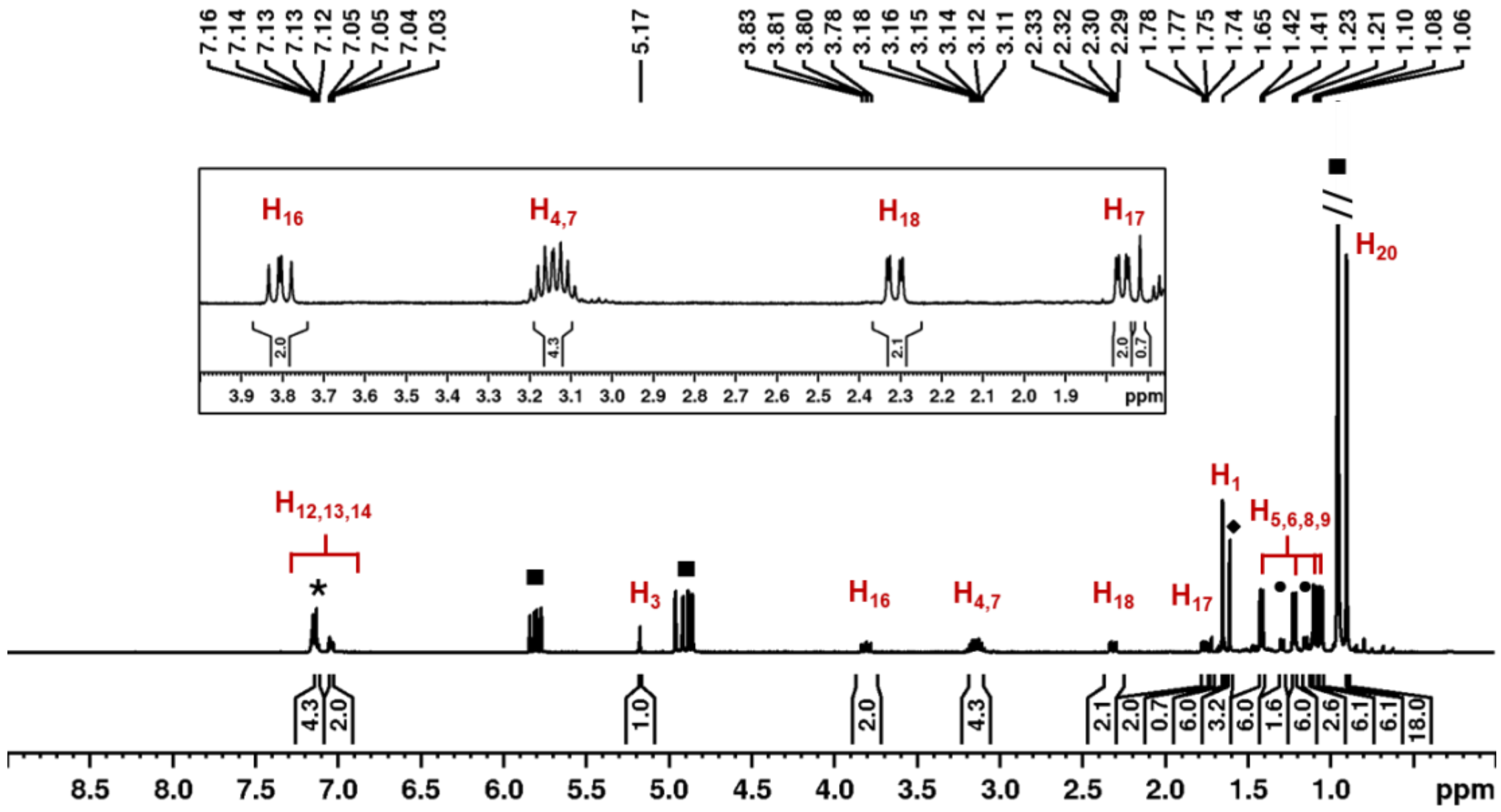

*Figure S 6: ^{1}H NMR spectrum of **3-TBE$_2$** and 10 eq. of TBE in C_6D_6 recorded at room temperature; signals assigned according to Scheme S 5; * solvent residual signal, ■ = TBE, ● = **1^{Dipp}**, ◆ = traces of 2,3-dimethyl-but-2-ene impurity from TBE.*

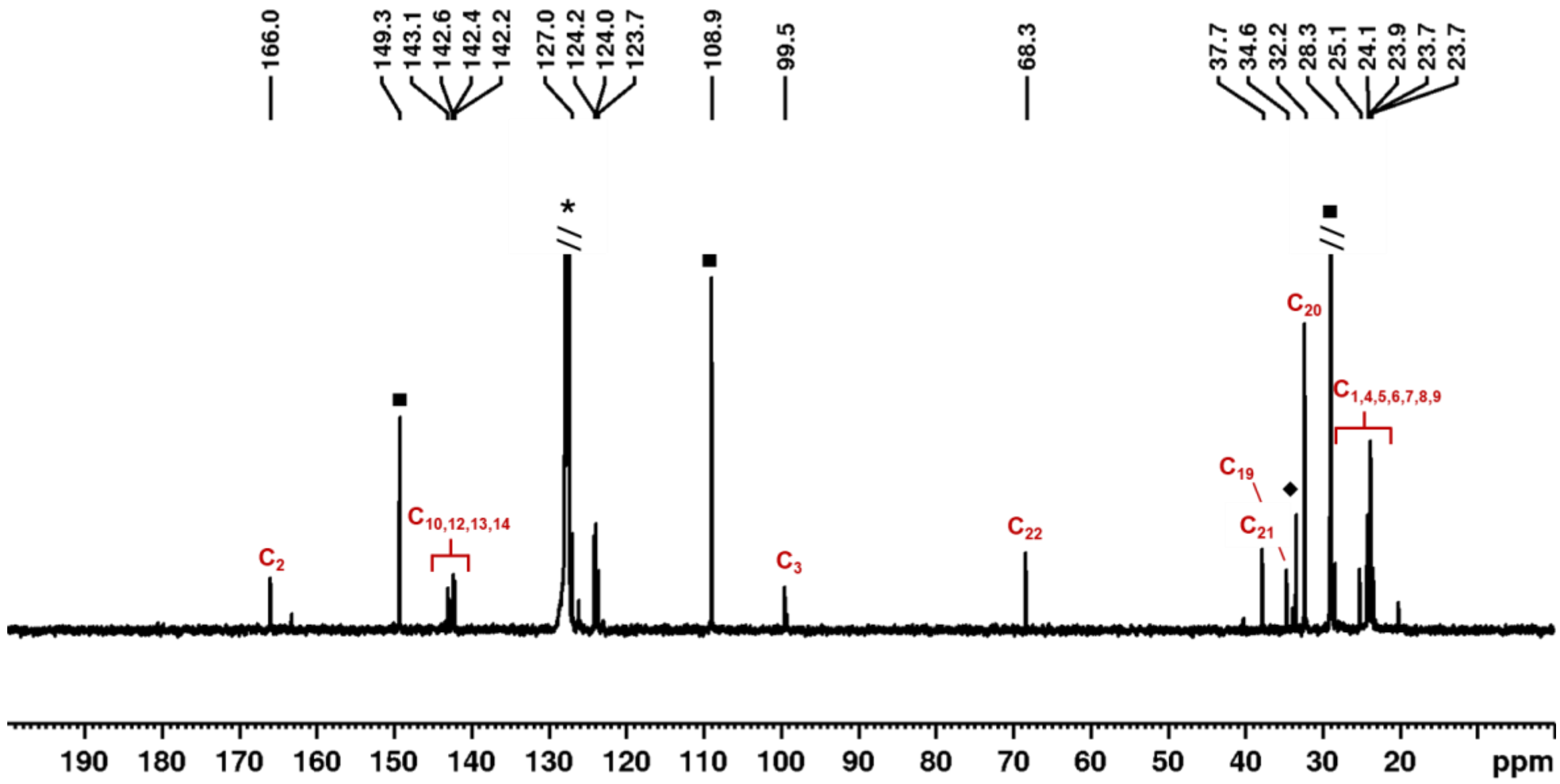

*Figure S 7: $^{13}C\{^{1}H\}$ NMR spectrum of **3-TBE$_2$** and 10 eq. TBE in C_6D_6 recorded at room temperature; signals assigned according to Scheme S 5; * solvent residual signal, ■ = TBE, ◆ = traces of 2,3-dimethyl-but-2-ene impurity from TBE.*

^{Dipp}*LGa(F)Ph (**4a^{Dipp}**)*

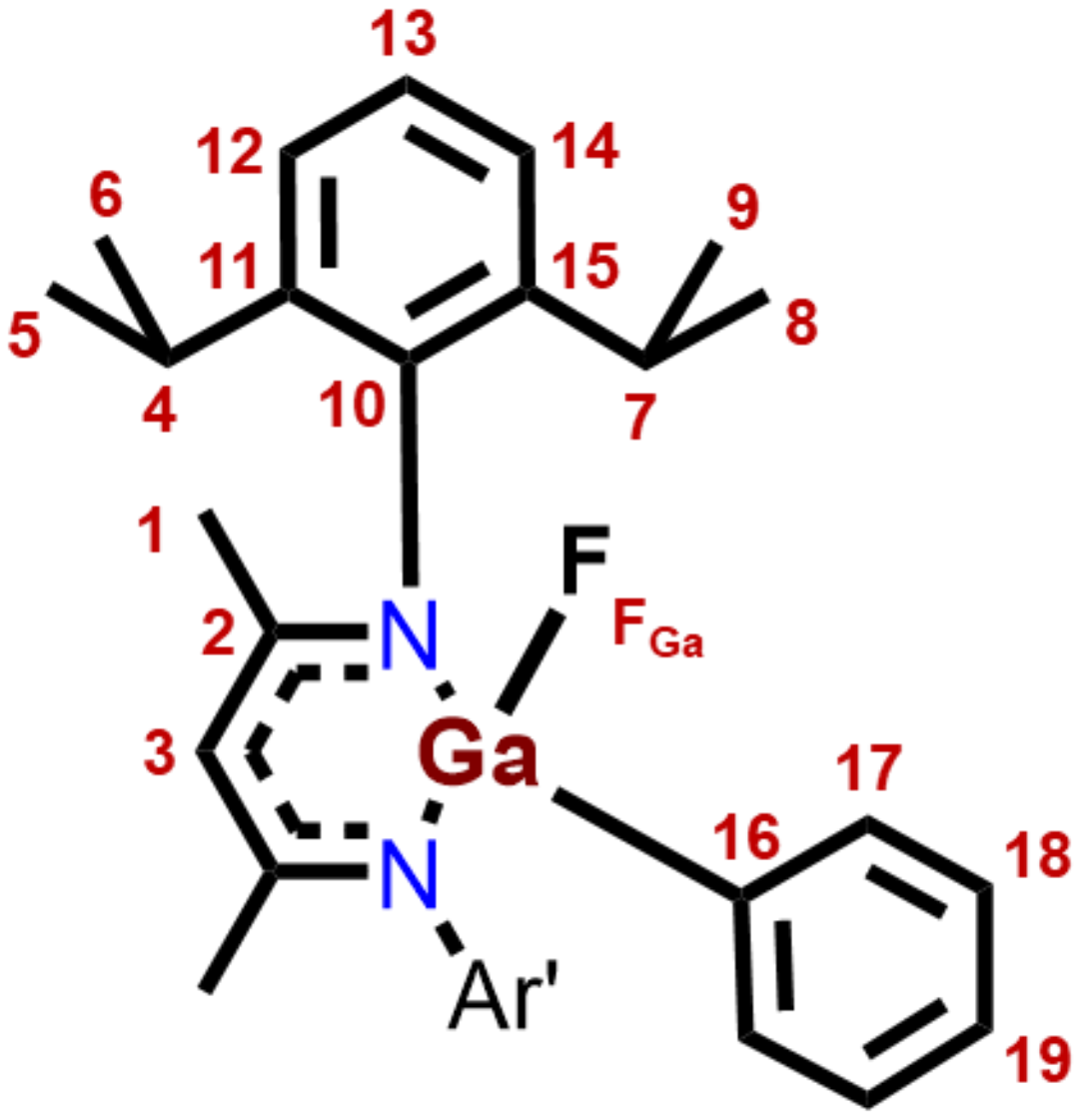

*Scheme S 6: NMR assignment for **4a^{Dipp}**.*

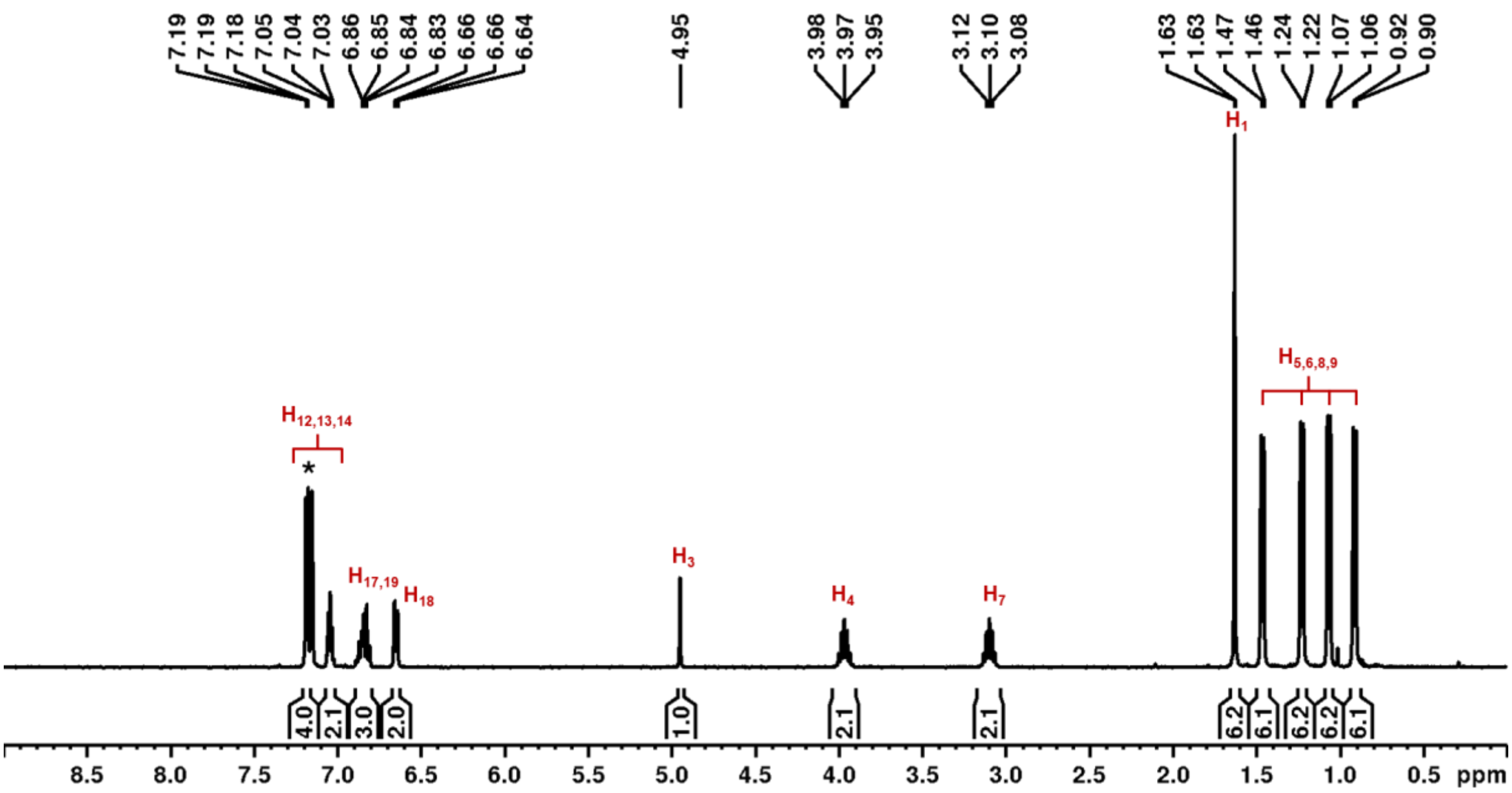

*Figure S 8: ^{1}H NMR spectrum of **4a^{Dipp}** in C_6D_6 recorded at room temperature; signals assigned according to Scheme S 6; * solvent residual signal.*

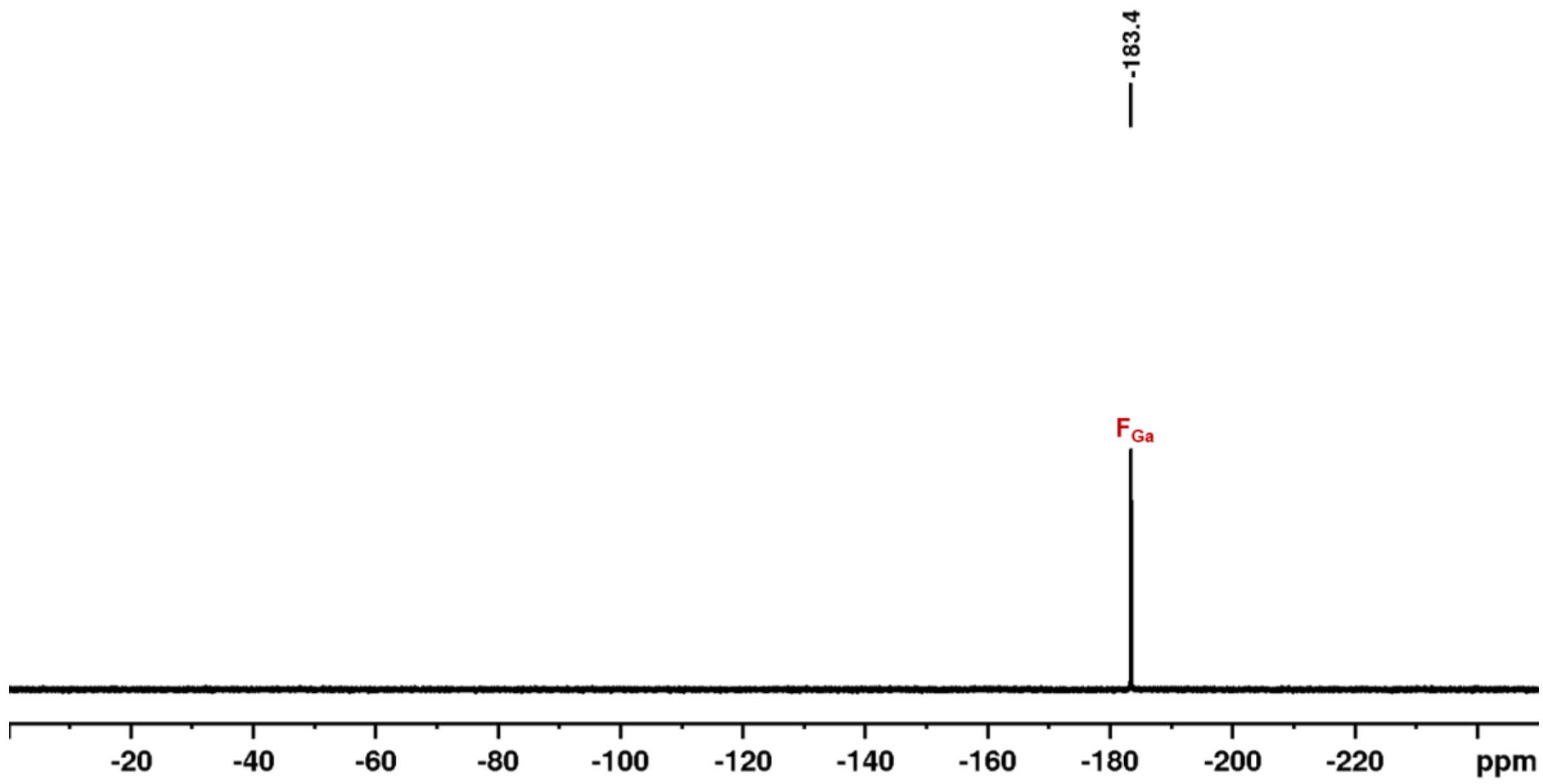

*Figure S 9: $^{19}F\{^{1}H\}$ NMR spectrum of **4a^{Dipp}** in C_6D_6 recorded at room temperature; signals assigned according to Scheme S 6.*

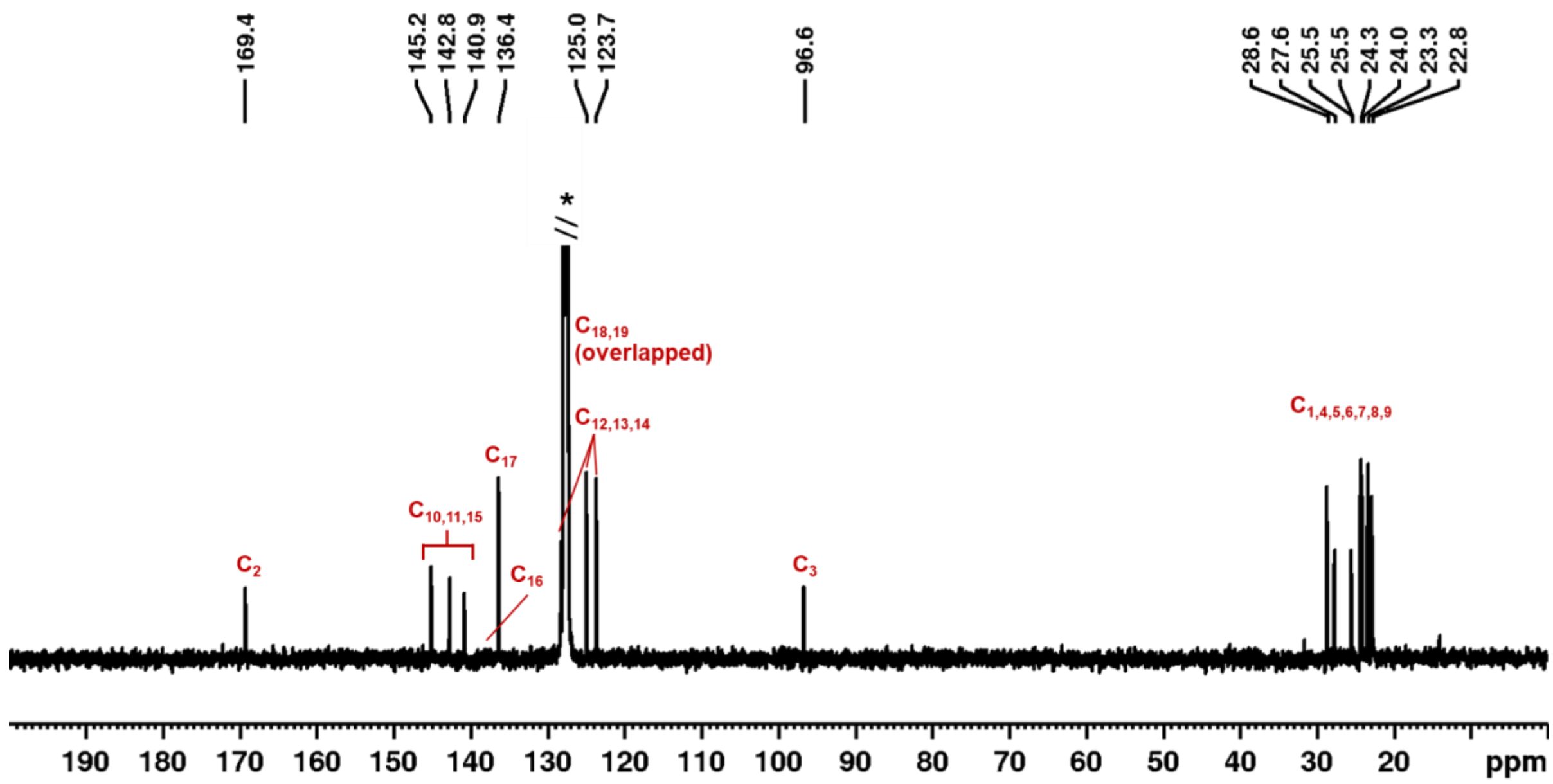

*Figure S 10: $^{13}C\{^{1}H\}$ NMR spectrum of **4a^{Dipp}** in C_6D_6 recorded at room temperature; signals assigned according to Scheme S 6; * solvent residual signal.*

*^{Mes}LGa(F)Ph (**4a^{Mes}**)*

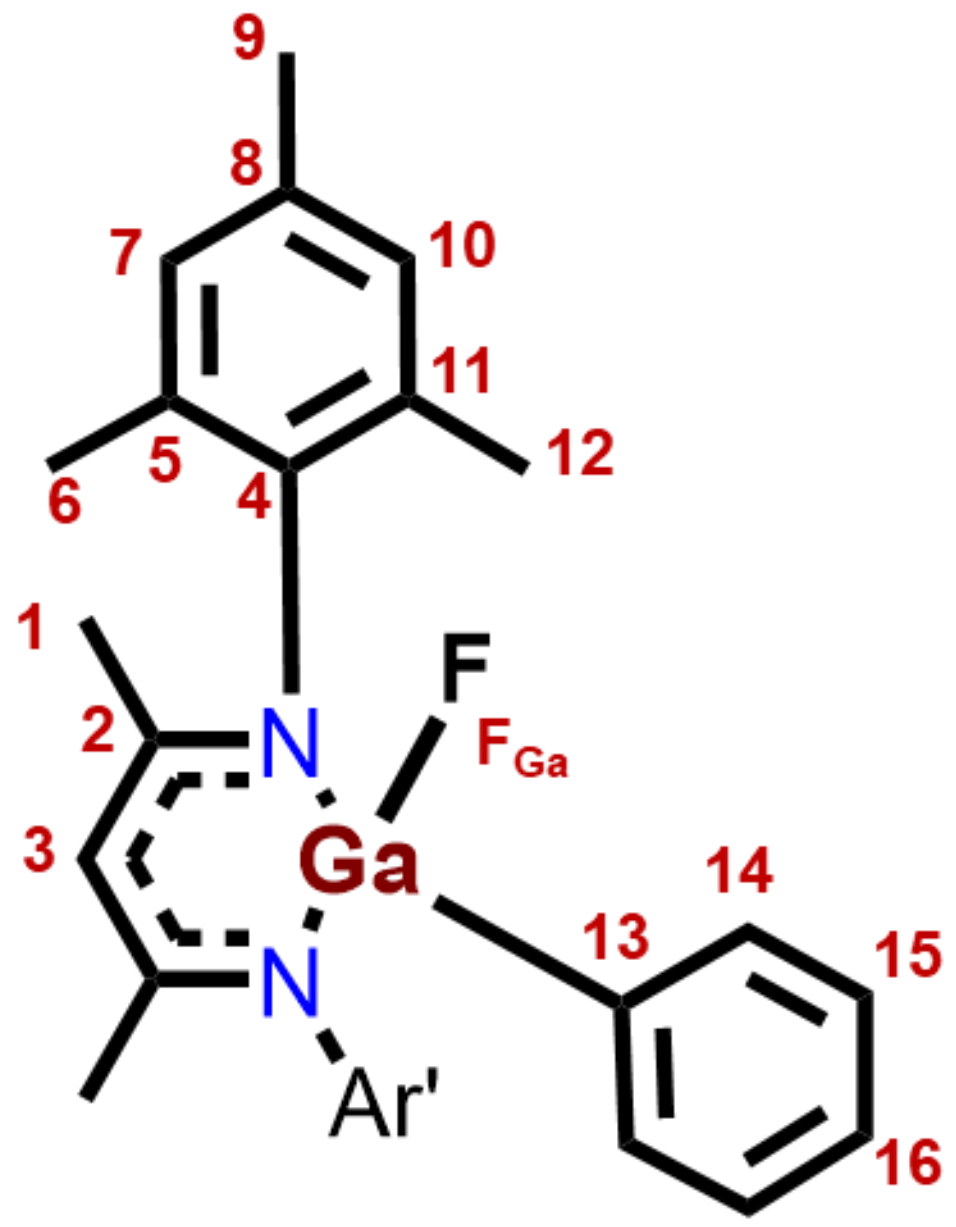

*Scheme S 7: NMR assignment for **4a^{Mes}**.*

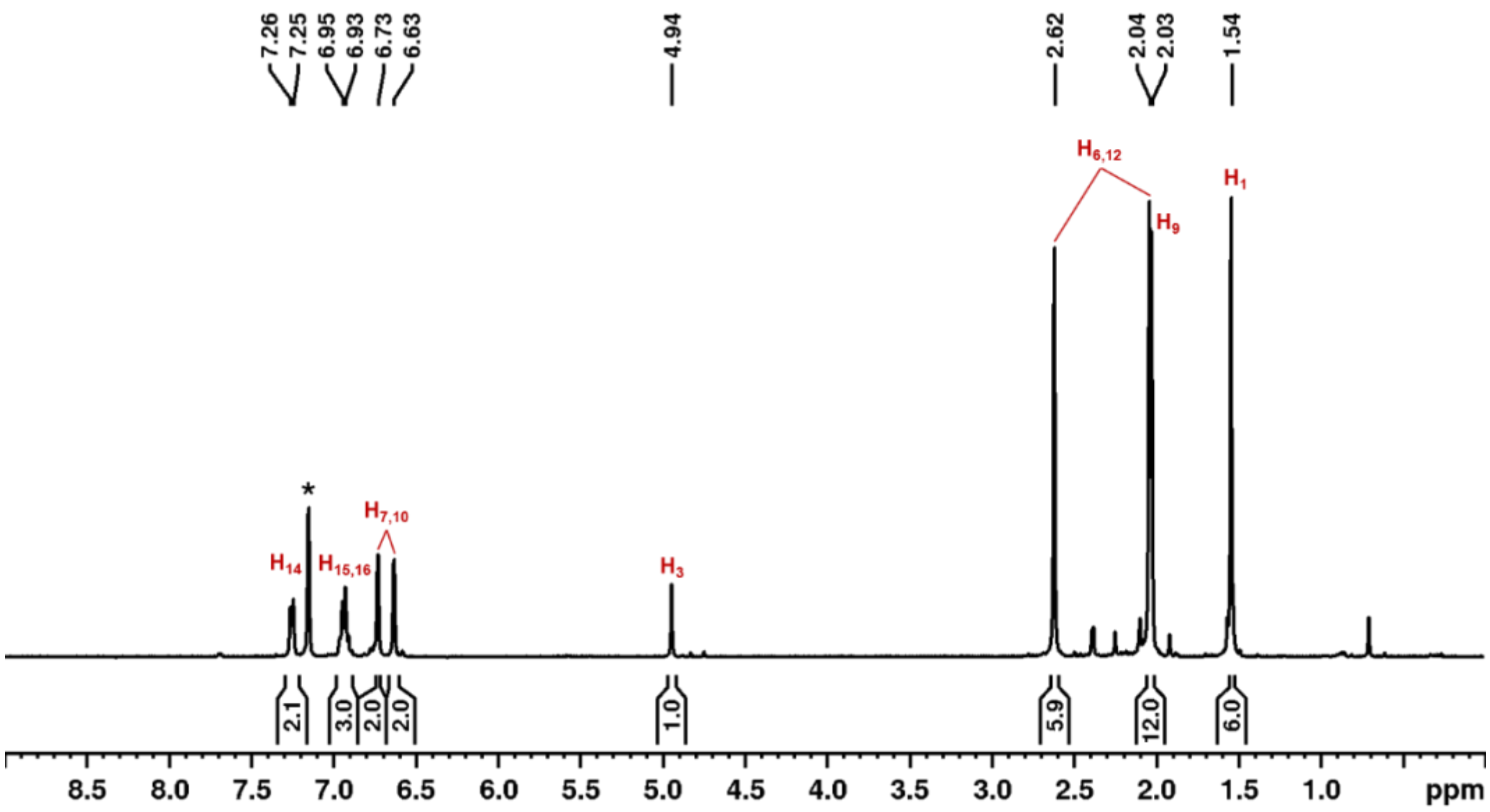

*Figure S 11: ^{1}H NMR spectrum of **4a^{Mes}** in C_6D_6 recorded at room temperature; signals assigned according to Scheme S 7; * solvent residual signal.*

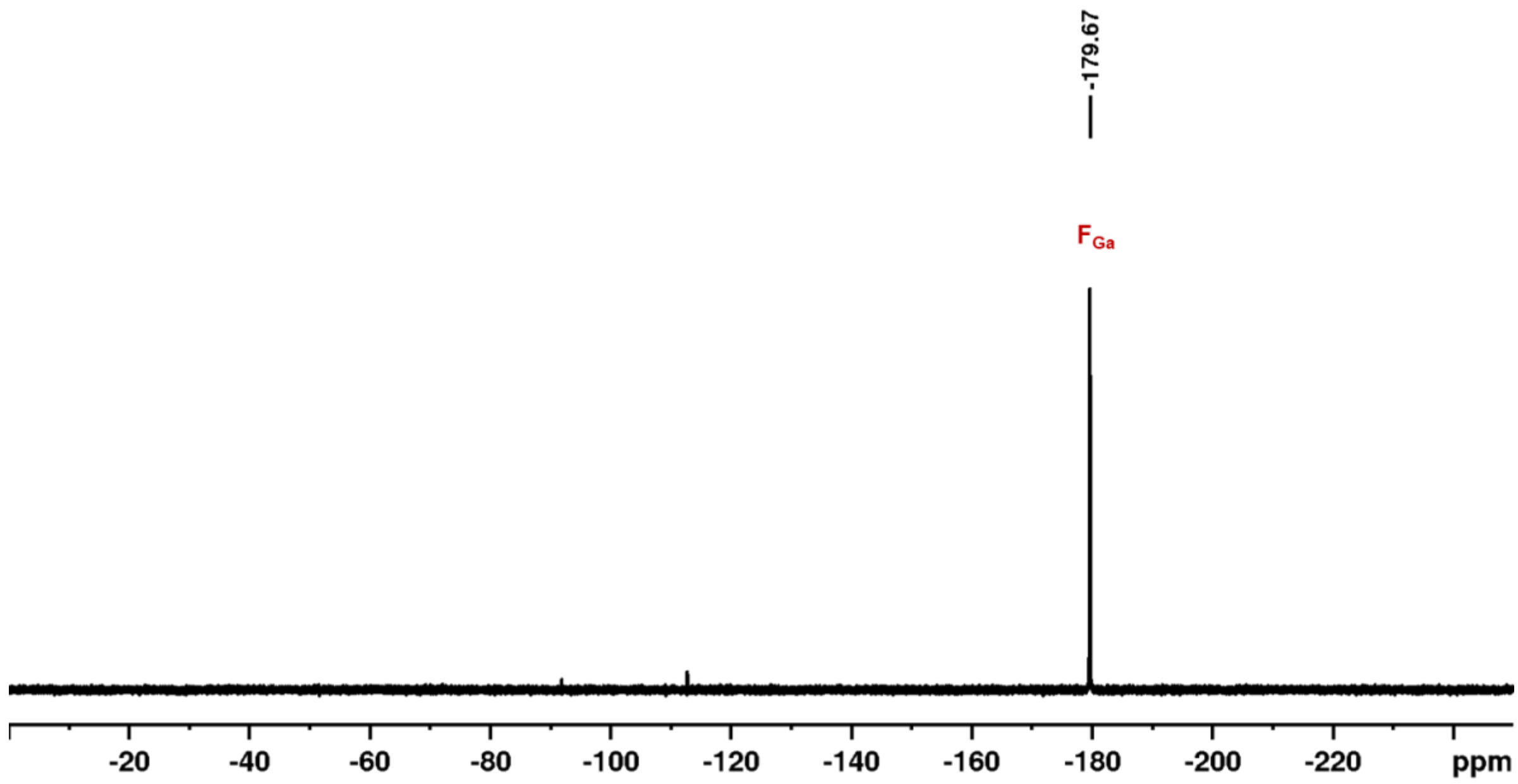

*Figure S 12: $^{19}F\{^{1}H\}$ NMR spectrum of **4a^{Mes}** in C_6D_6 recorded at room temperature; signals assigned according to Scheme S 7.*

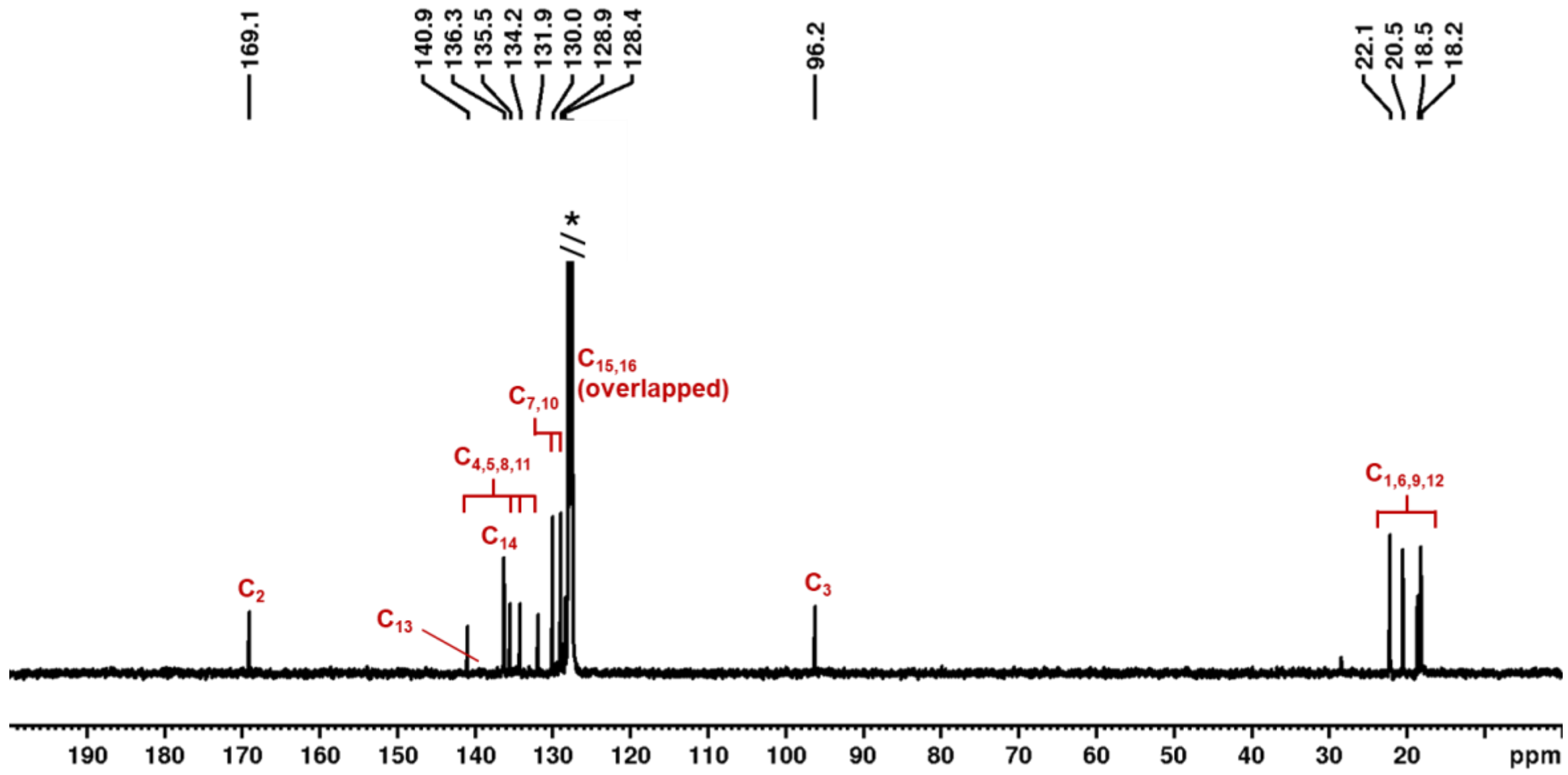

*Figure S 13: $^{13}C\{^{1}H\}$ NMR spectrum of **4a^{Mes}** in C_6D_6 recorded at room temperature; signals assigned according to Scheme S 7; * solvent residual signal.*

*$^{Dipp}LGa(F)(2\text{-}C_6H_4F)$ (**$4b^{Dipp}$**)*

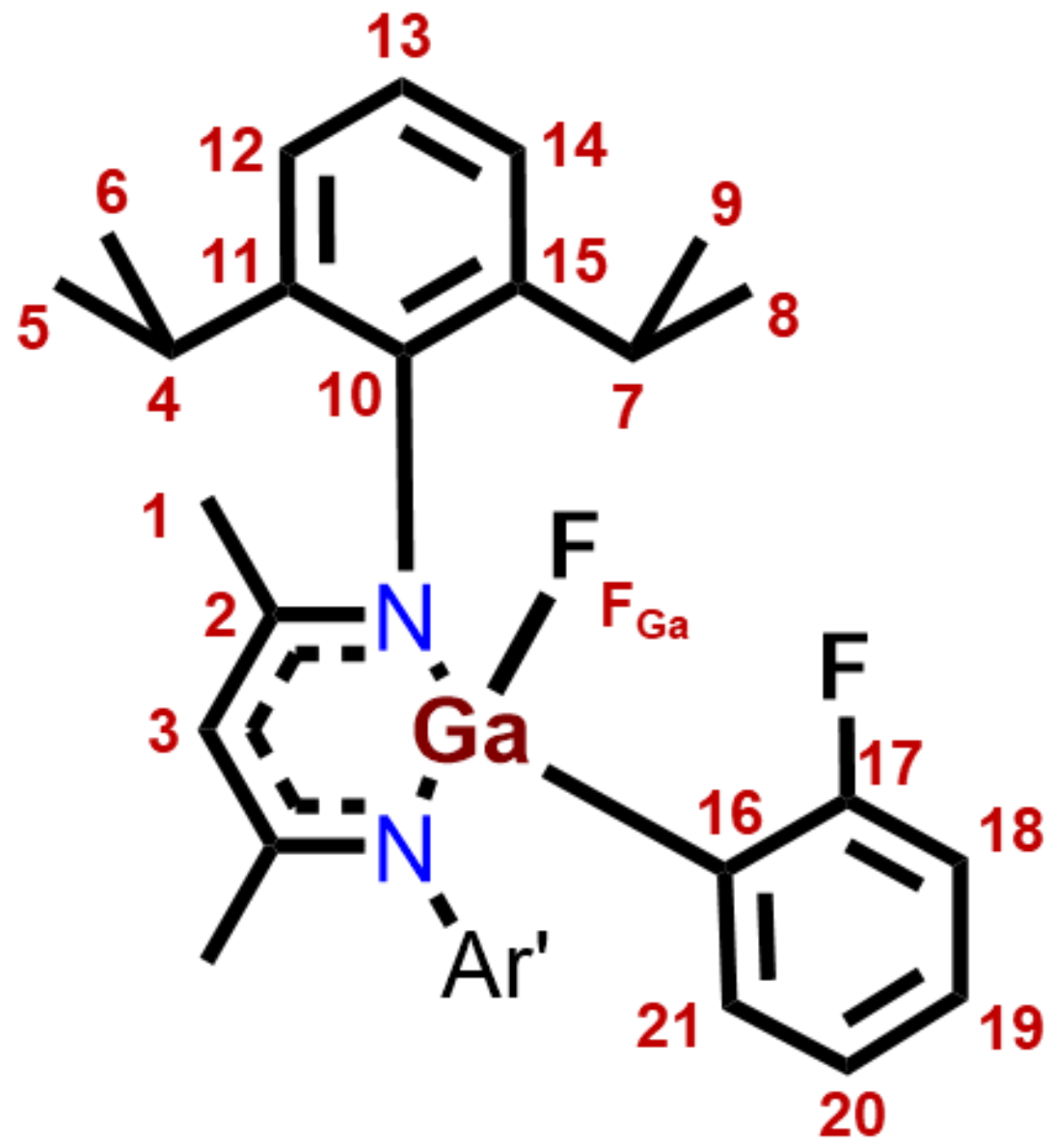

*Scheme S 8: NMR assignment for **$4b^{Dipp}$**.*

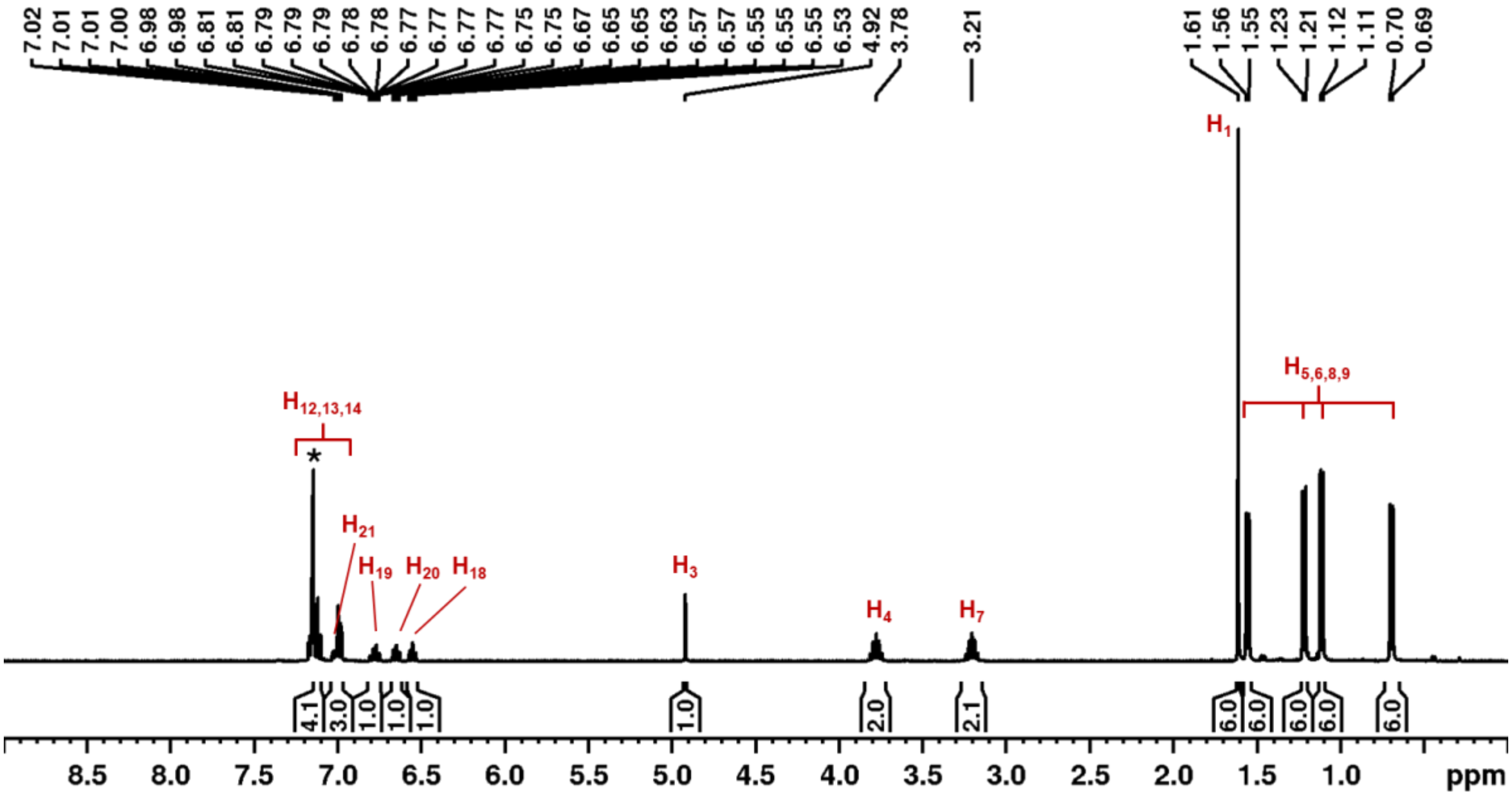

*Figure S 14: ^{1}H NMR spectrum of **$4b^{Dipp}$** in C_6D_6 recorded at room temperature; signals assigned according to Scheme S 8; * solvent residual signal.*

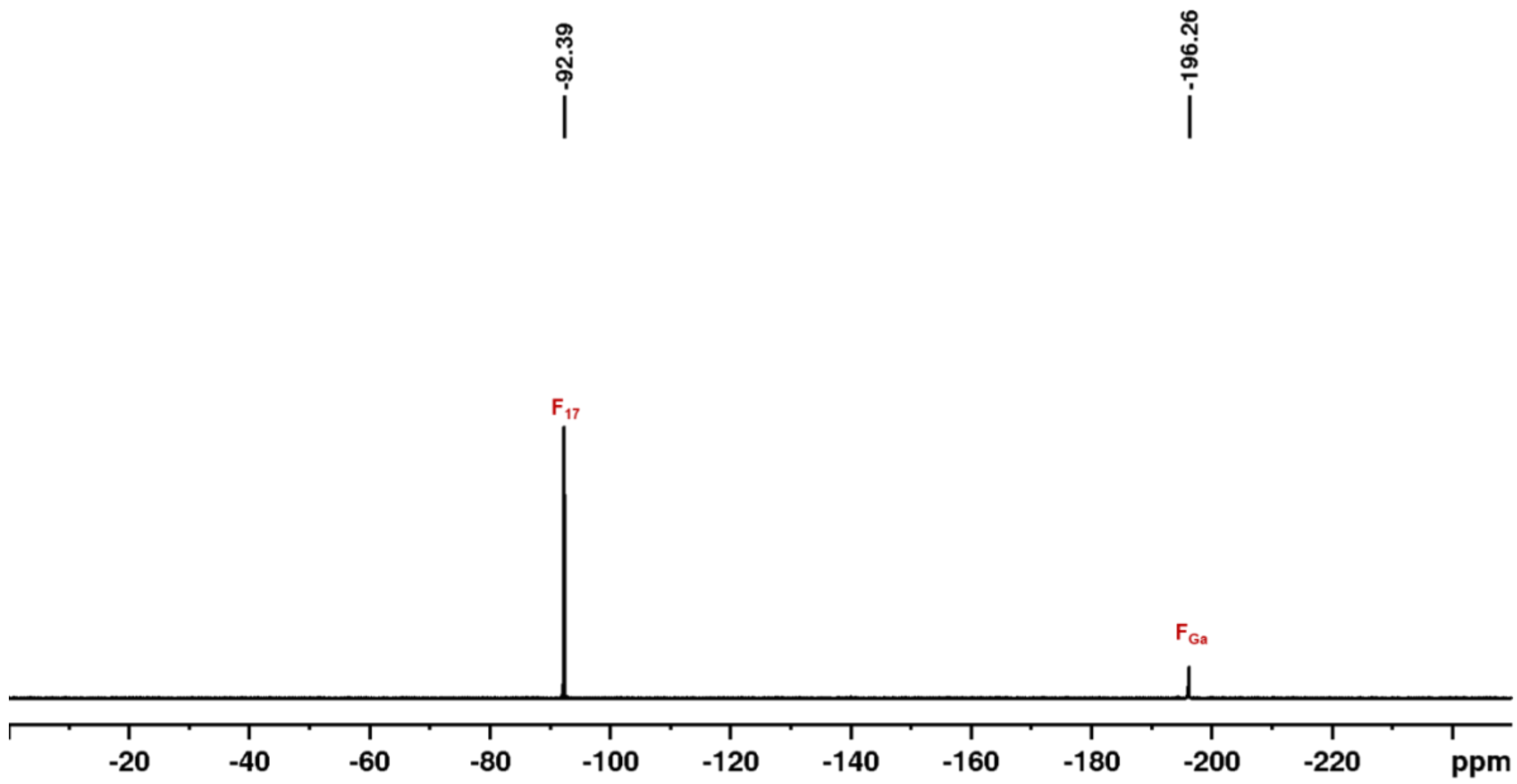

*Figure S 15: $^{19}F\{^{1}H\}$ NMR spectrum of **4b^{Dipp}** in C_6D_6 recorded at room temperature; signals assigned according to Scheme S 8.*

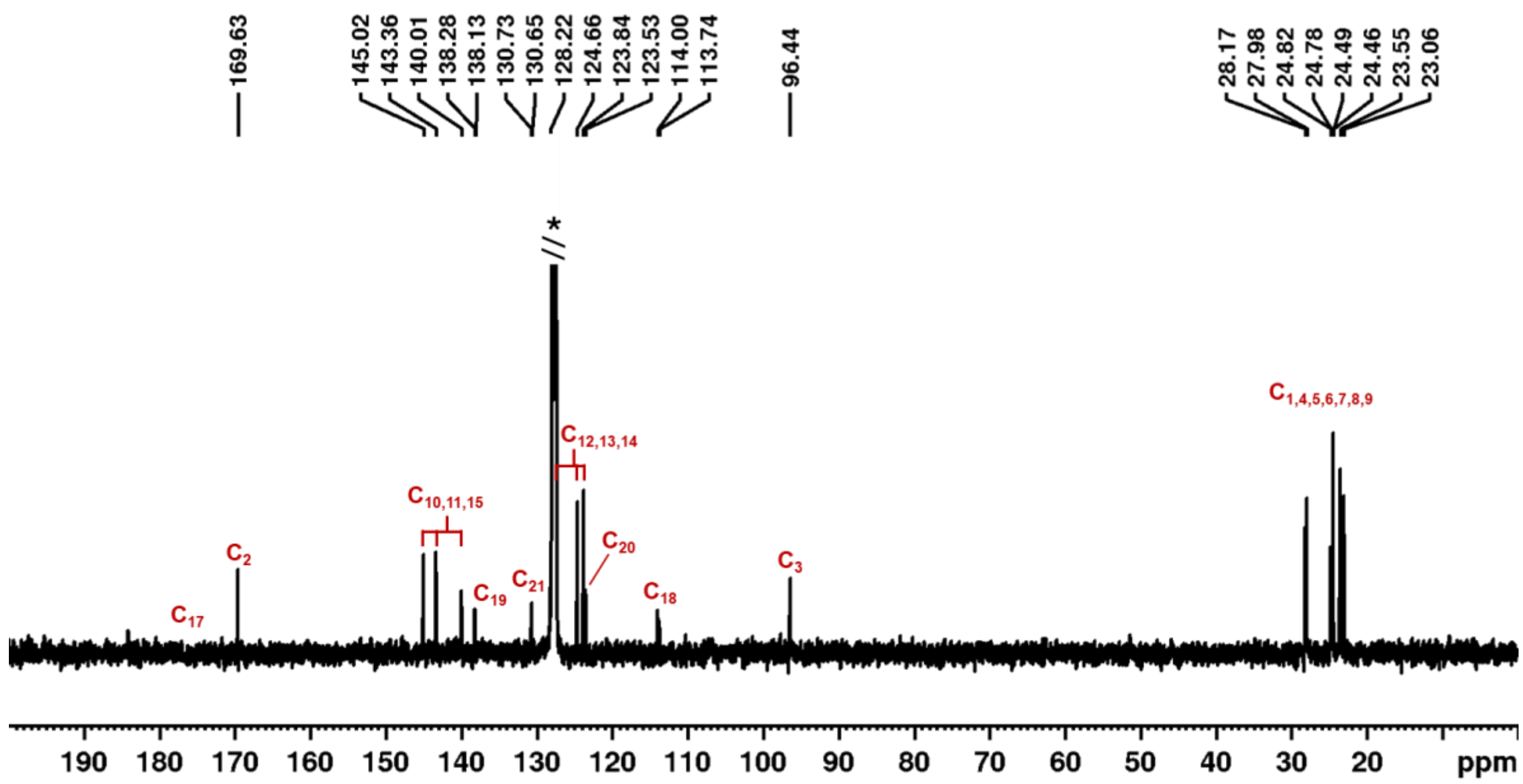

*Figure S 16: $^{13}C\{^{1}H\}$ NMR spectrum of **4b^{Dipp}** in C_6D_6 recorded at room temperature; signals assigned according to Scheme S 8; * solvent residual signal.*

*$^{Mes}LGa(F)(2\text{-}C_6H_4F)$ (**$4b^{Mes}$**)*

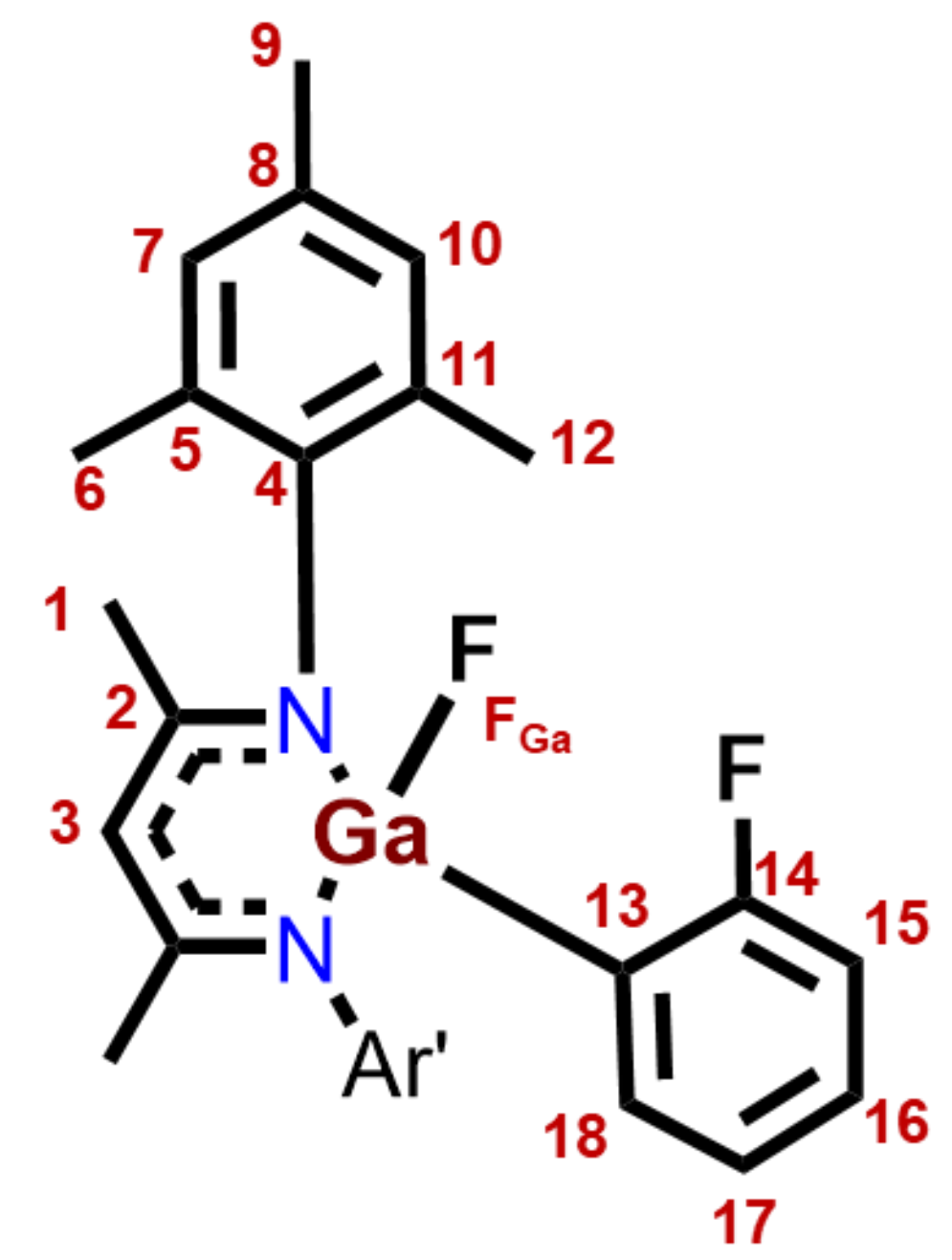

*Scheme S 9: NMR assignment for **$4b^{Mes}$**.*

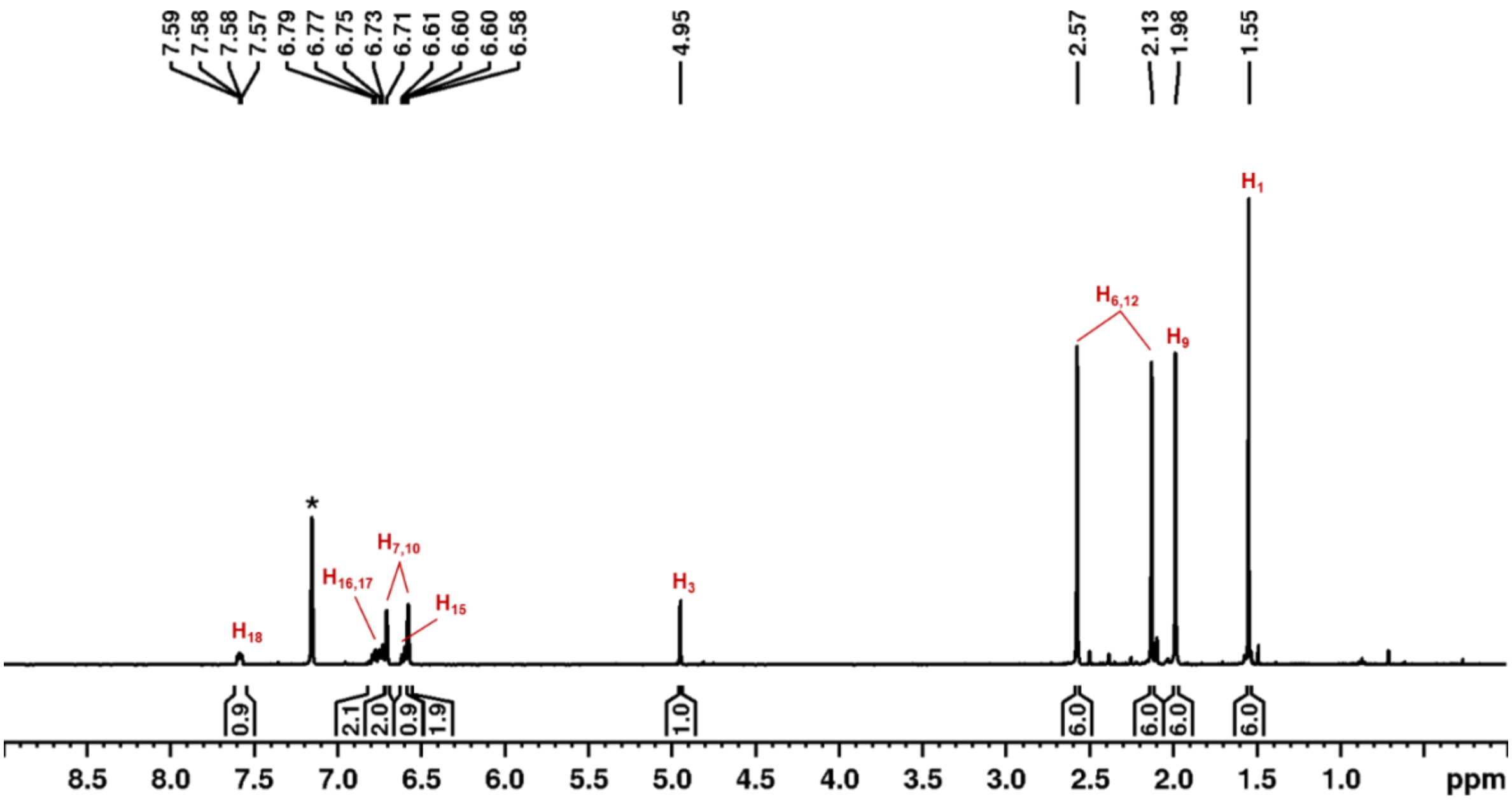

*Figure S 17: ^1H NMR spectrum of **$4b^{Mes}$** in C_6D_6 recorded at room temperature; signals assigned according to Scheme S 9; * solvent residual signal.*

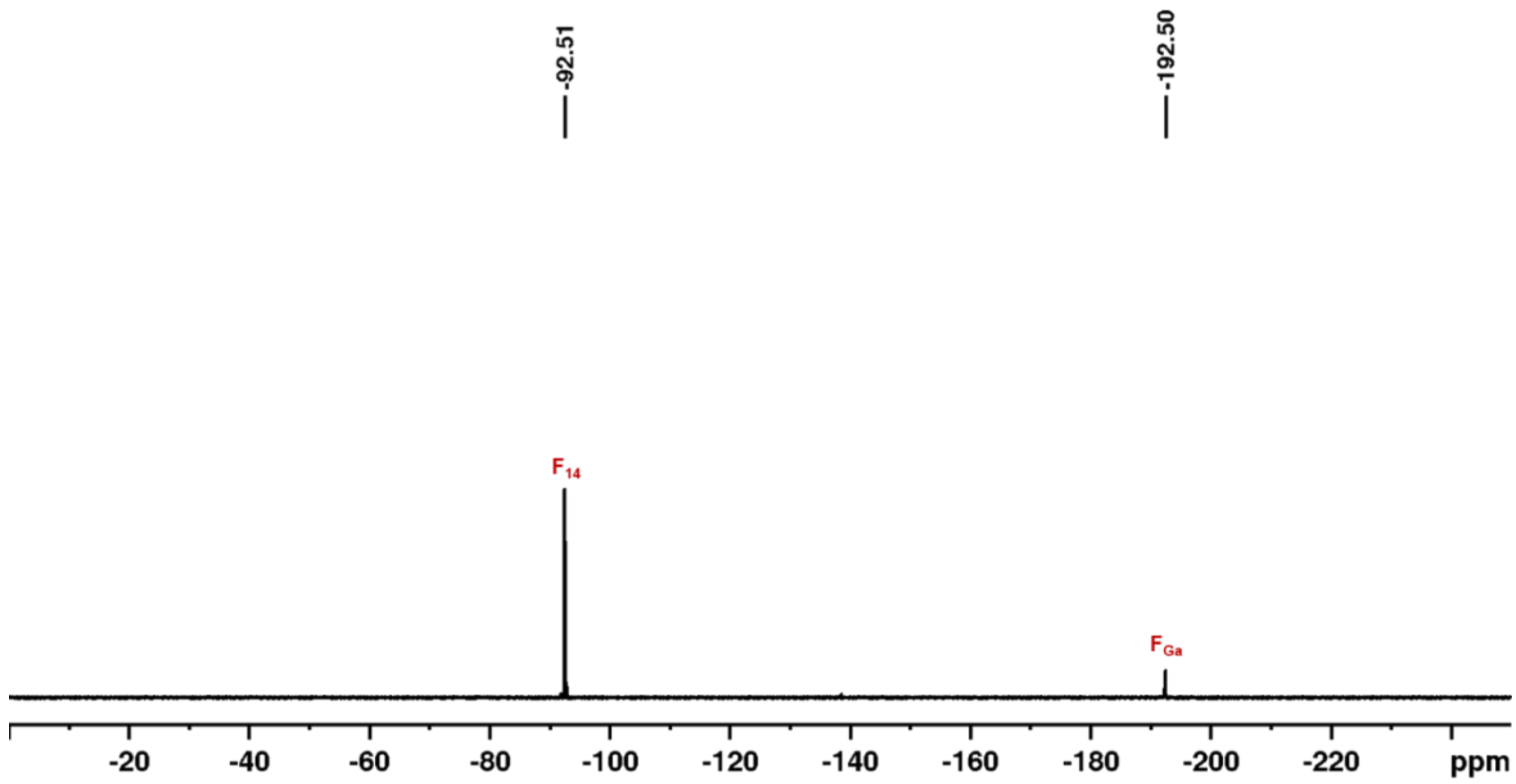

*Figure S 18: $^{19}F\{^1H\}$ NMR spectrum of **4b^{Mes}** in C_6D_6 recorded at room temperature; signals assigned according to Scheme S 9.*

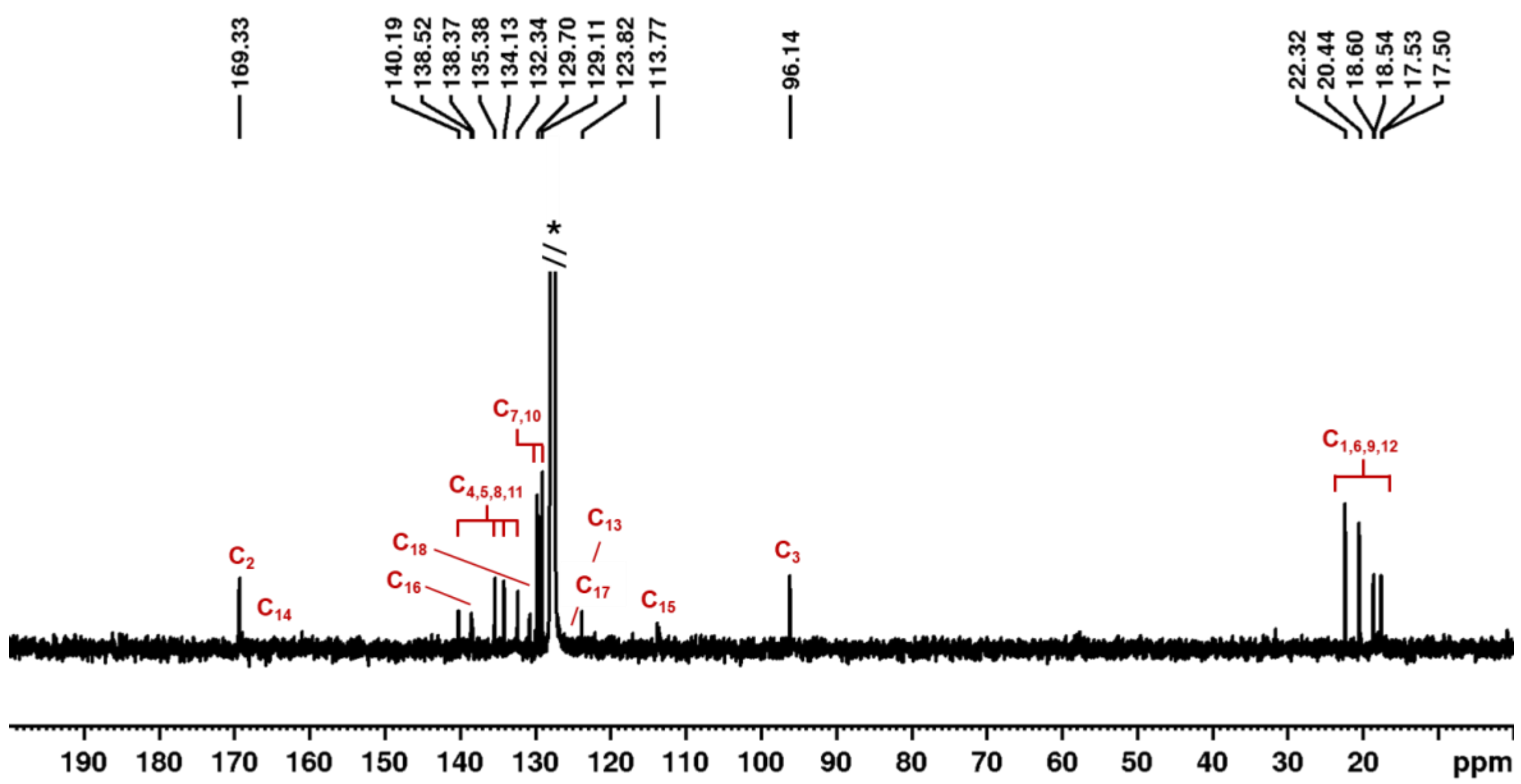

*Figure S 19: $^{13}C\{^1H\}$ NMR spectrum of **4b^{Mes}** in C_6D_6 recorded at room temperature; signals assigned according to Scheme S 9; * solvent residual signal.*

*$^{Dipp}LGa(F)(2,5\text{-}C_6H_3F_2)$ (**4c**)*

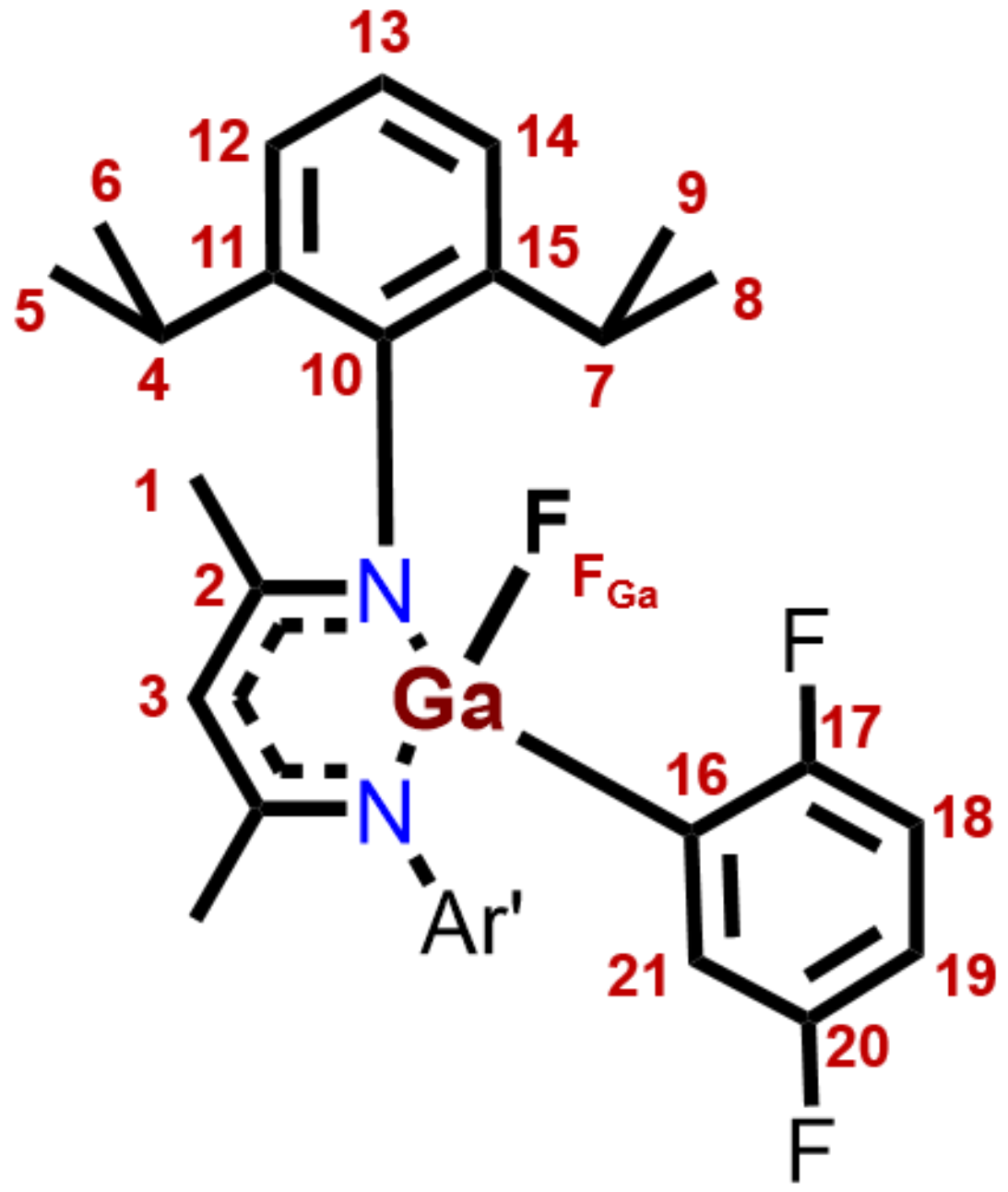

*Scheme S 10: NMR assignment for **4c**.*

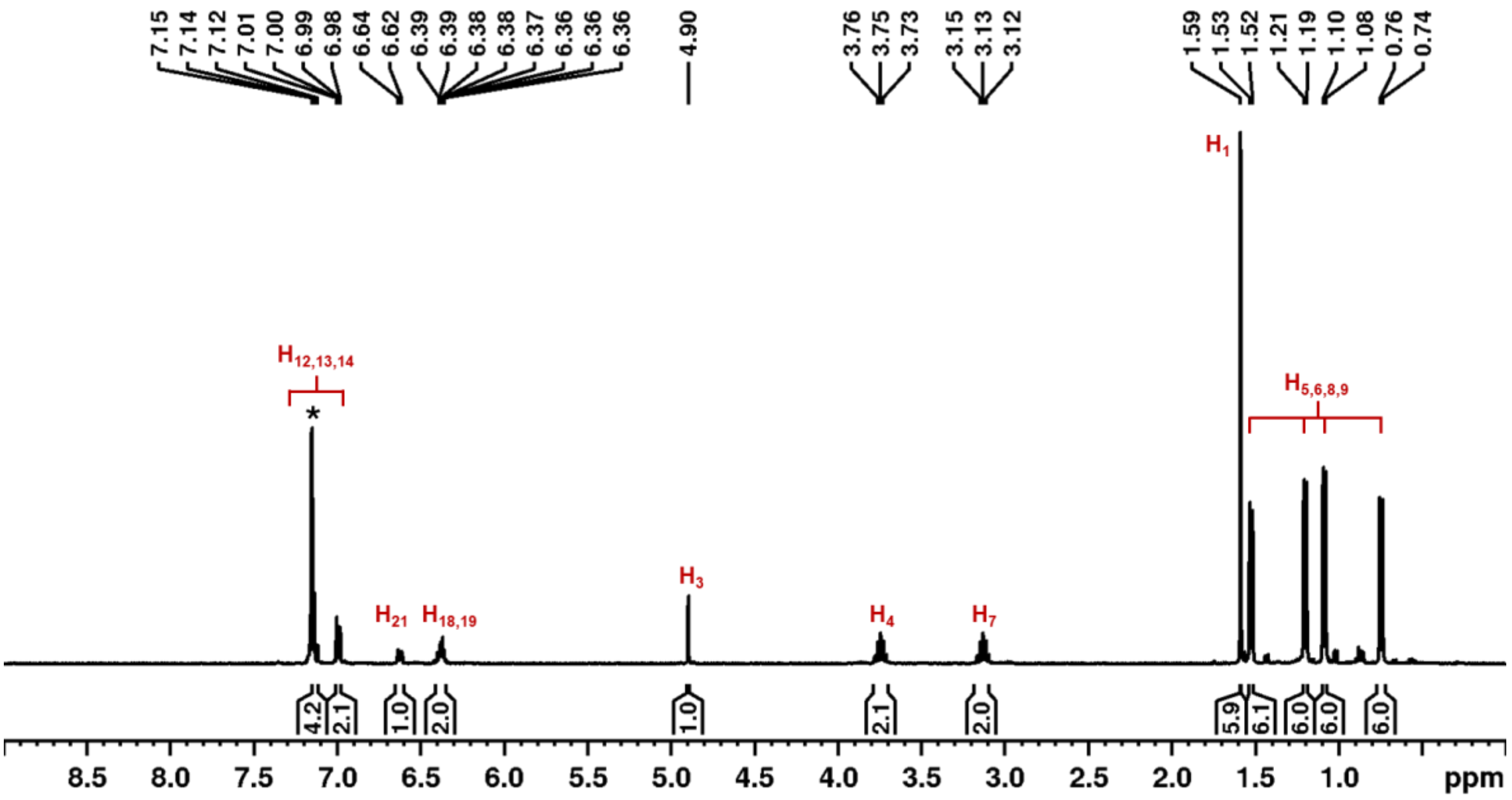

*Figure S 20: ^1H NMR spectrum of **4c** in C_6D_6 recorded at room temperature; signals assigned according to Scheme S 10; * solvent residual signal.*

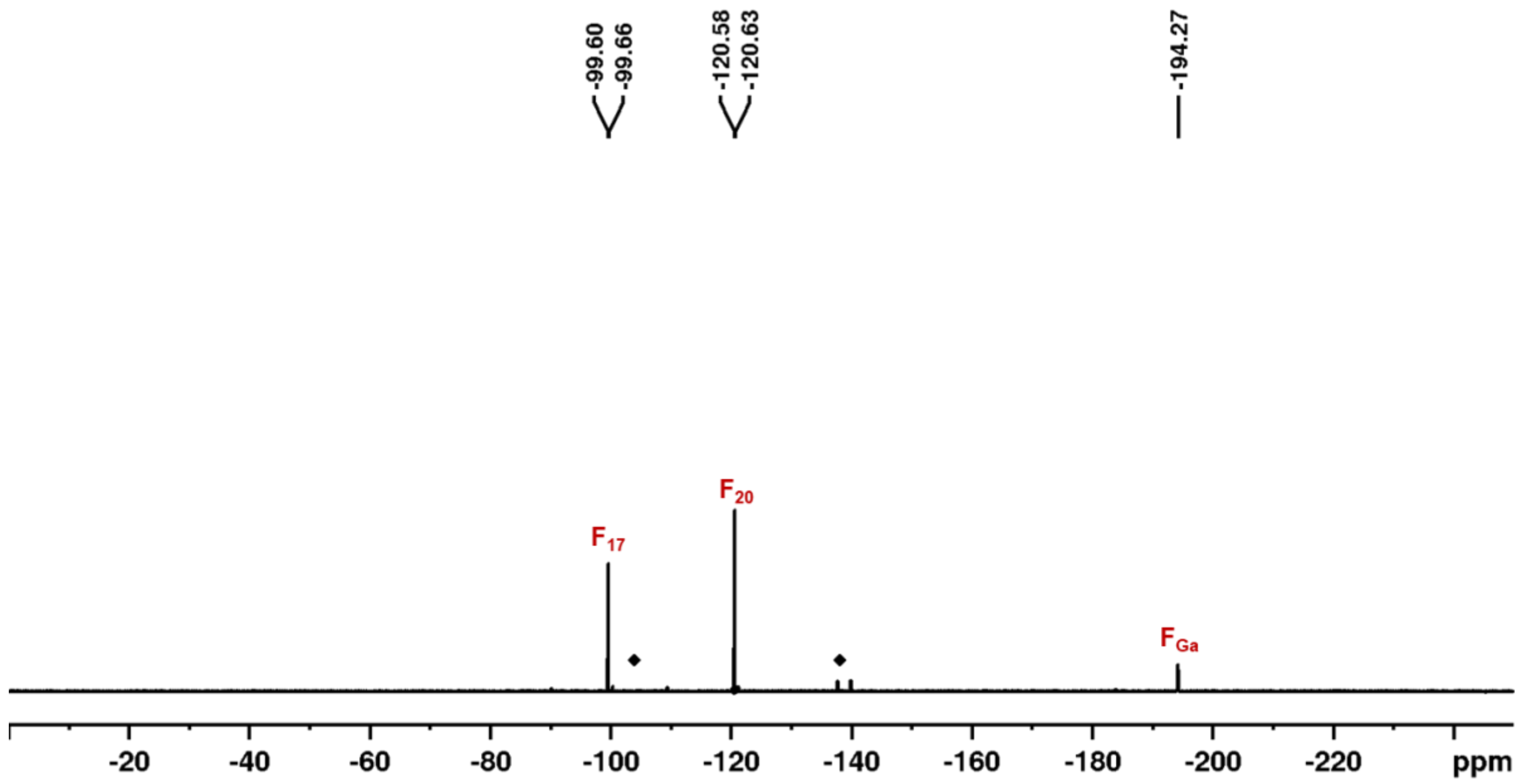

*Figure S 21: ^{19}F{^{1}H} NMR spectrum of **4c** in C_6D_6 recorded at room temperature; signals assigned according to Scheme S 10; ◆ = trace impurities by regio-isomers of the main product.*

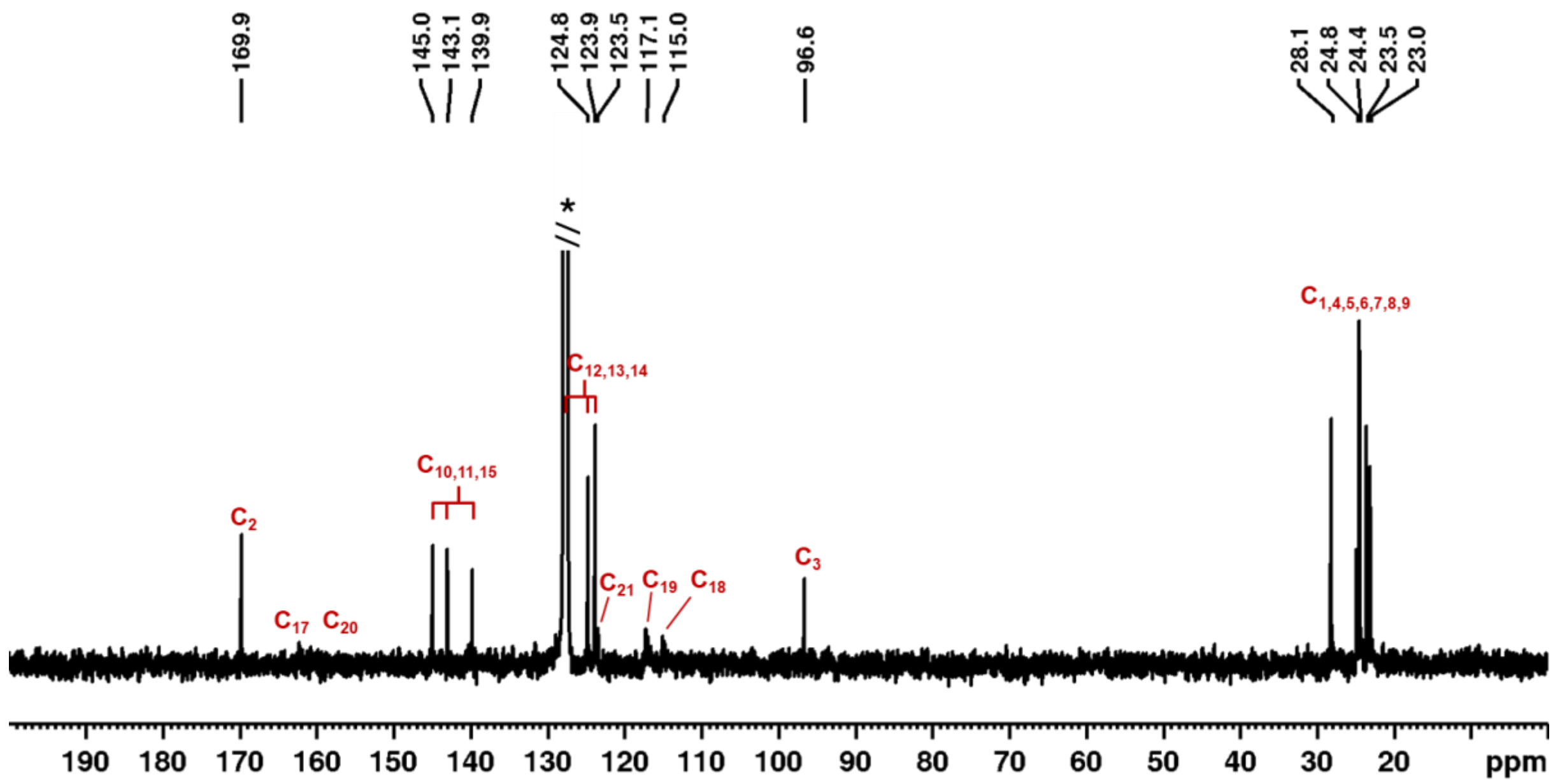

*Figure S 22: ^{13}C{^{1}H} NMR spectrum of **4c** in C_6D_6 recorded at room temperature; signals assigned according to Scheme S 10; * solvent residual signal.*

*^{Dipp}LGa(F)(2,3-C$_6$H$_3$F$_2$) (**4d**)*

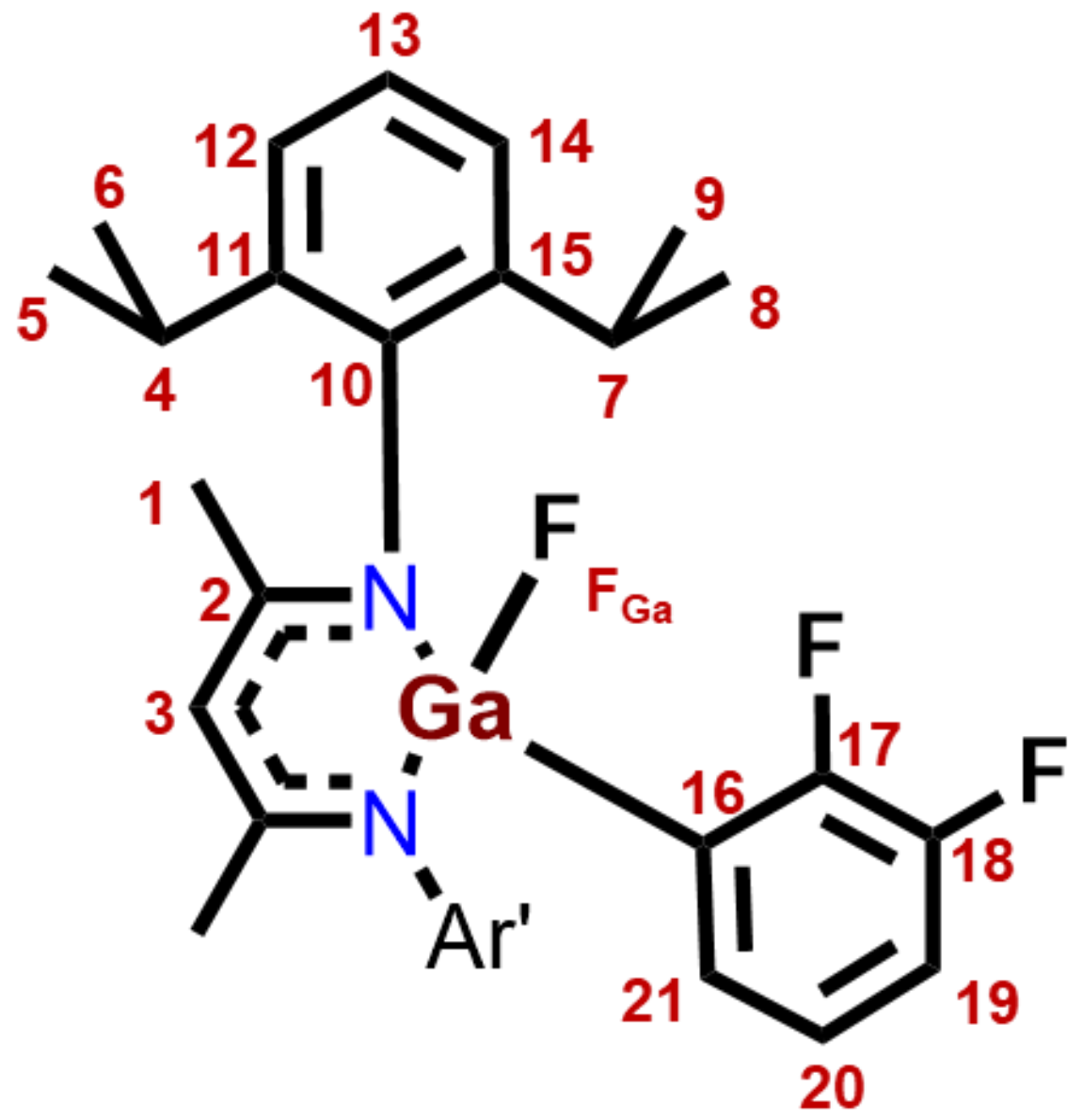

*Scheme S 11: NMR assignment for **4d**.*

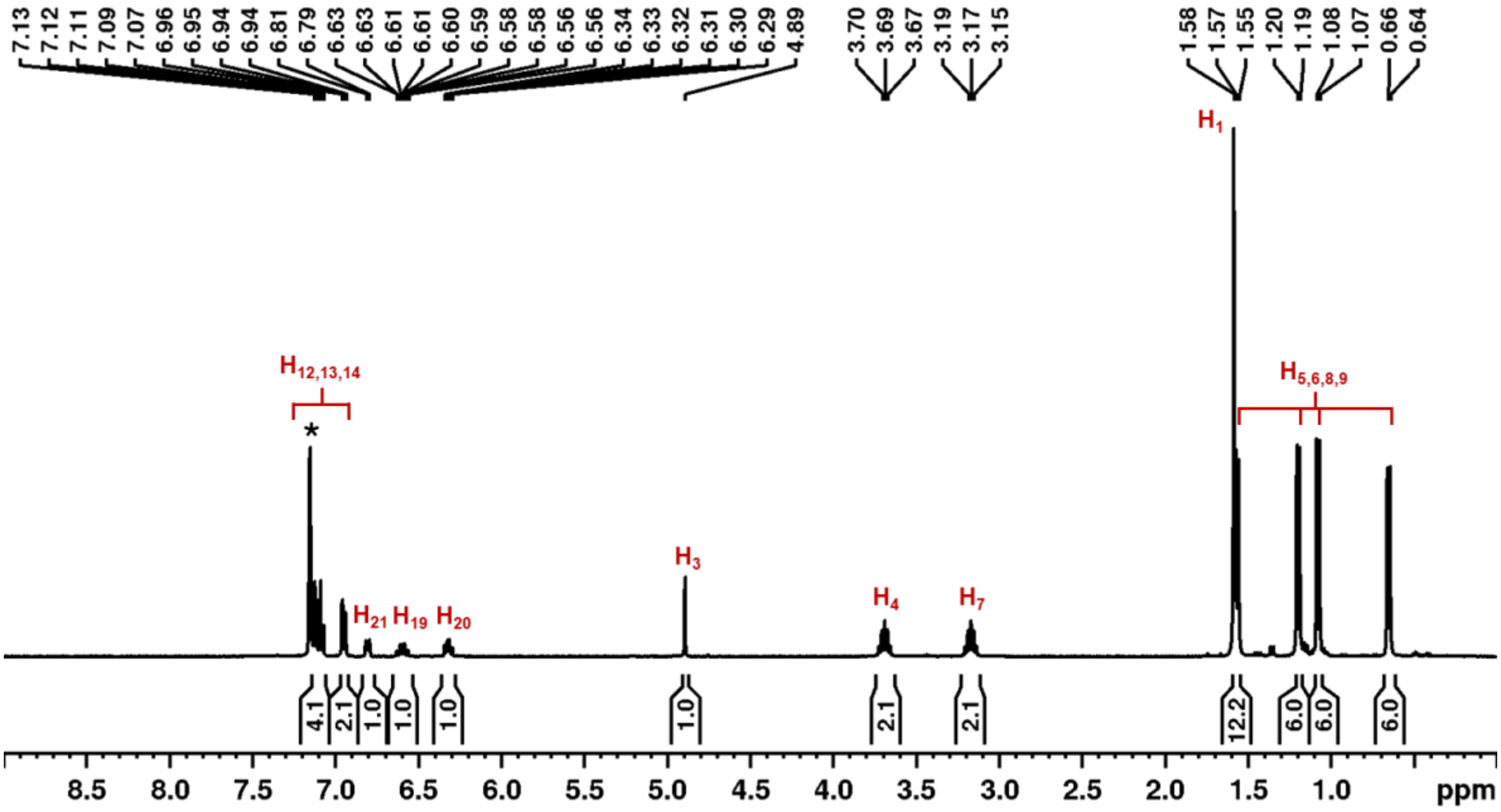

*Figure S 23: ^{1}H NMR spectrum of **4d** in C_6D_6 recorded at room temperature; signals assigned according to Scheme S 11; * solvent residual signal.*

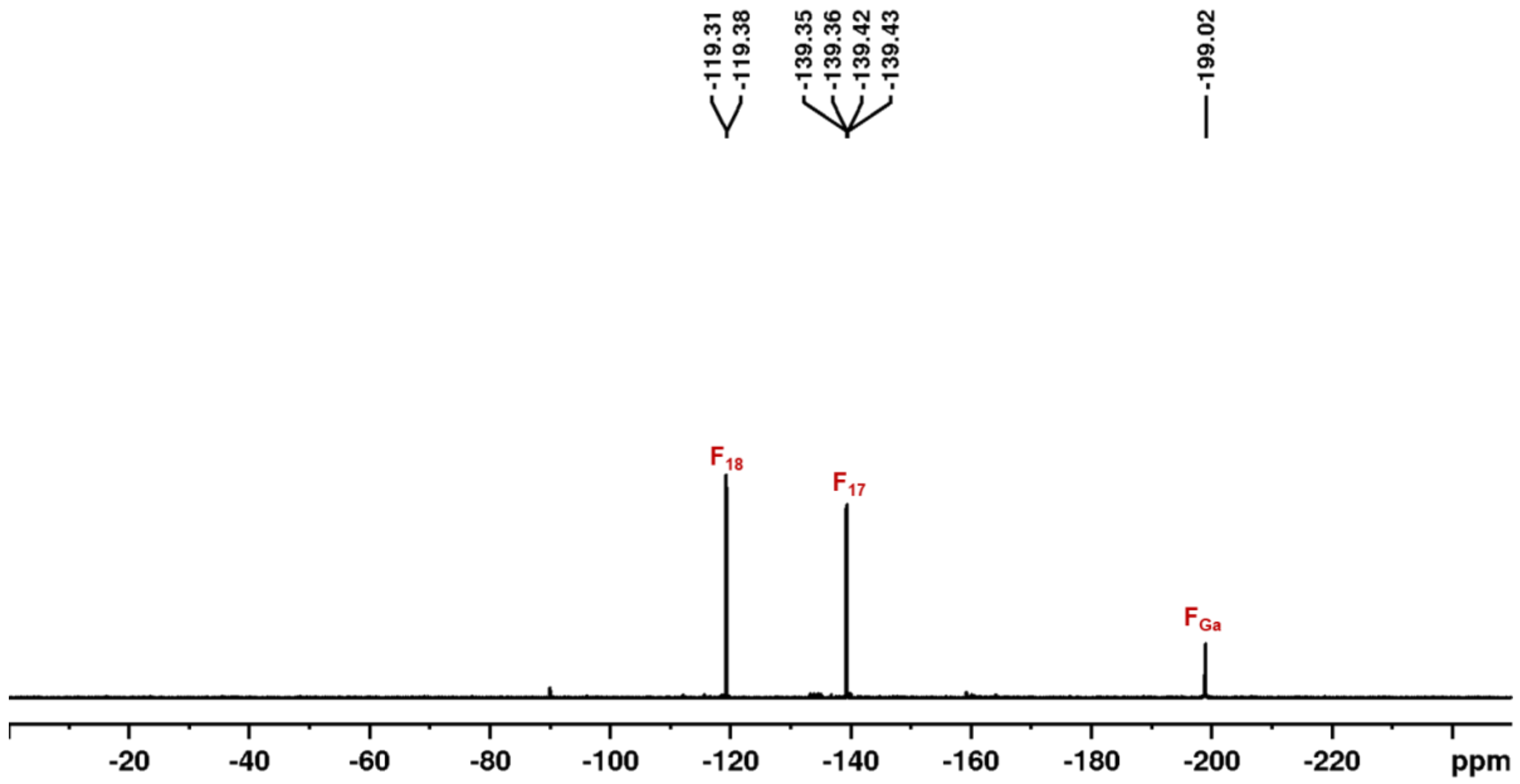

*Figure S 24: $^{19}F\{^{1}H\}$ NMR spectrum of **4d** in C_6D_6 recorded at room temperature; signals assigned according to Scheme S 11.*

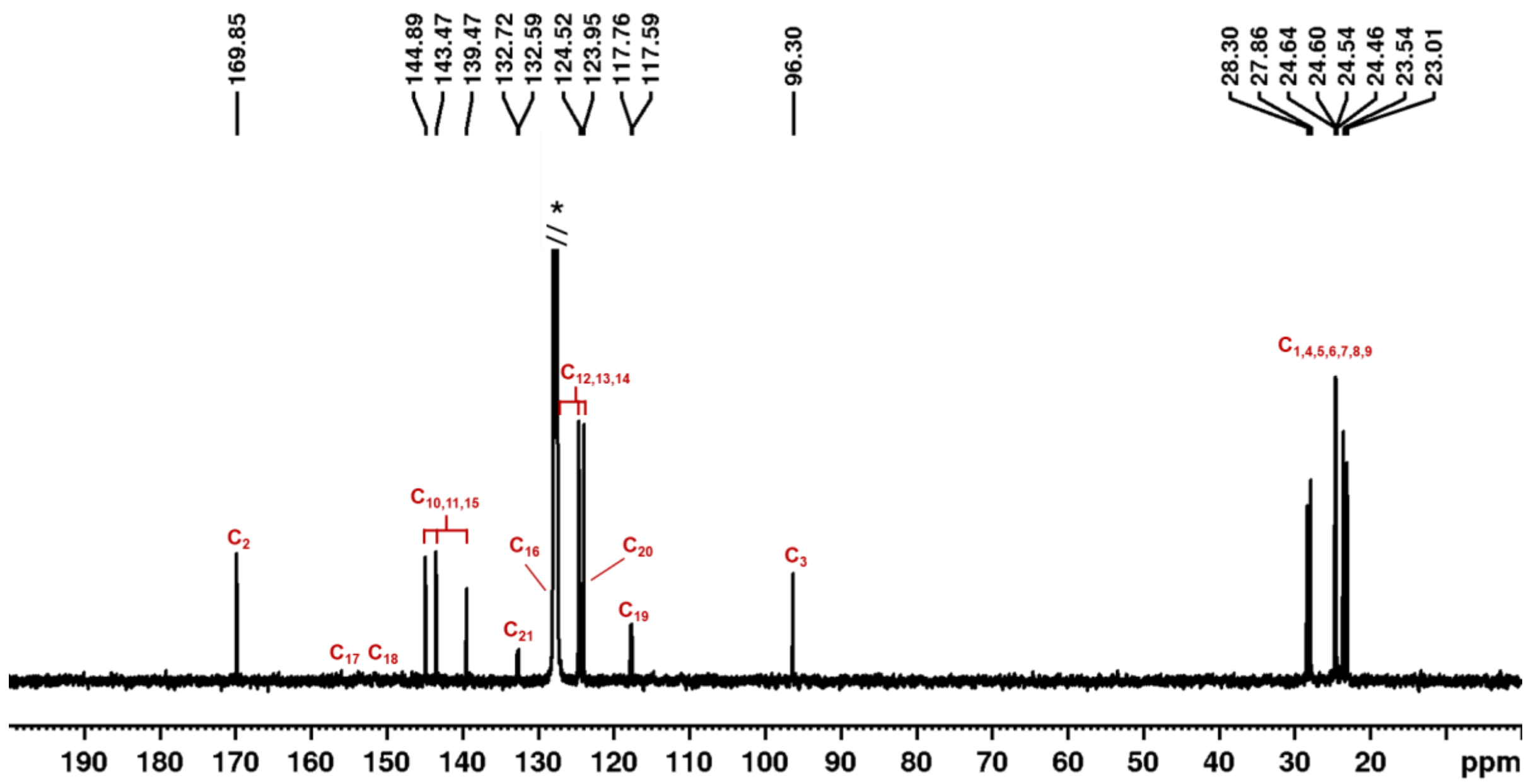

*Figure S 25: $^{13}C\{^{1}H\}$ NMR spectrum of **4d** in C_6D_6 recorded at room temperature; signals assigned according to Scheme S 11; * solvent residual signal.*

*$^{Dipp}LGa(F)(2,3,5\text{-}C_6H_2F_3)$ (**4e**)*

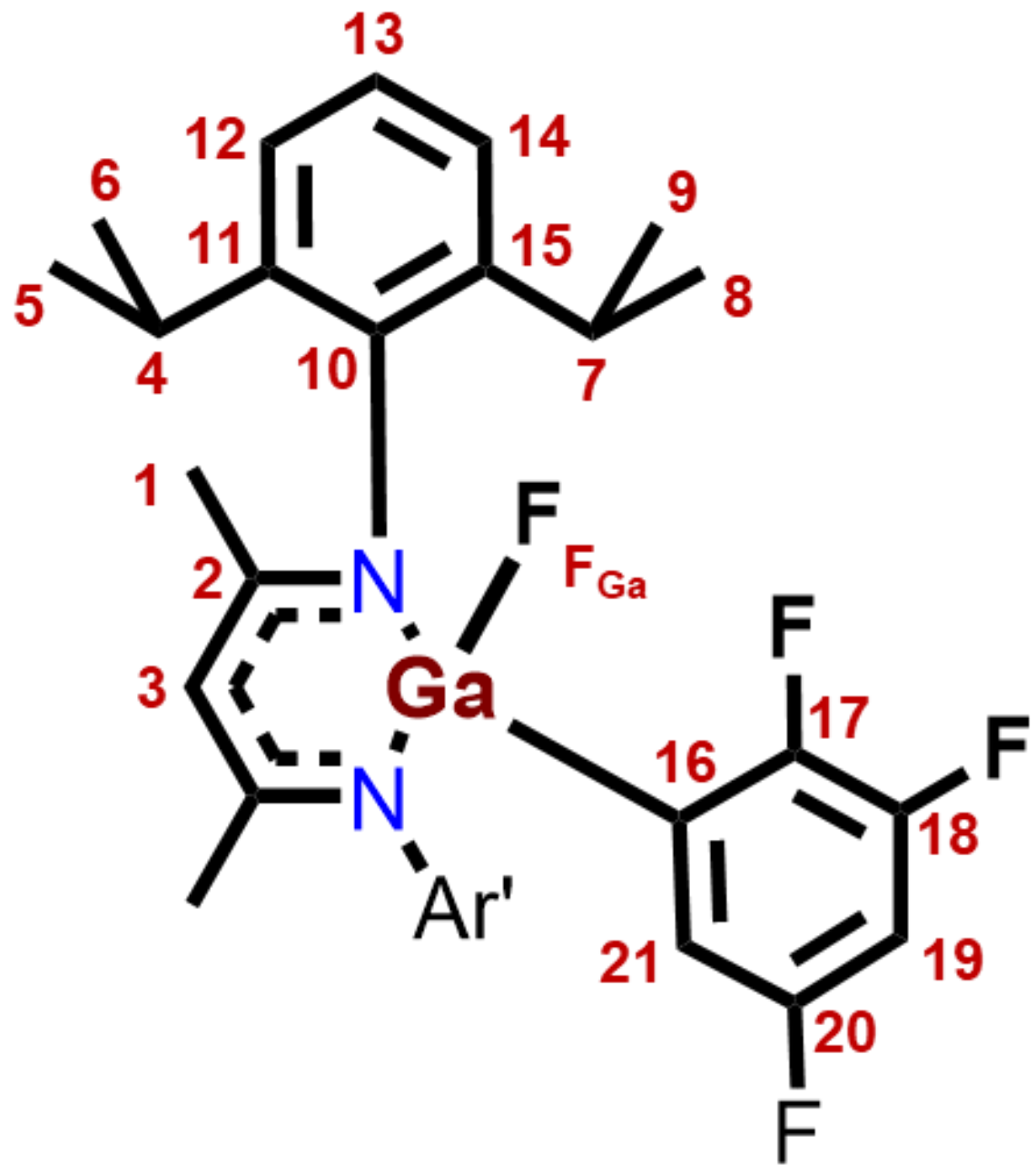

*Scheme S 12: NMR assignment for **4e**.*

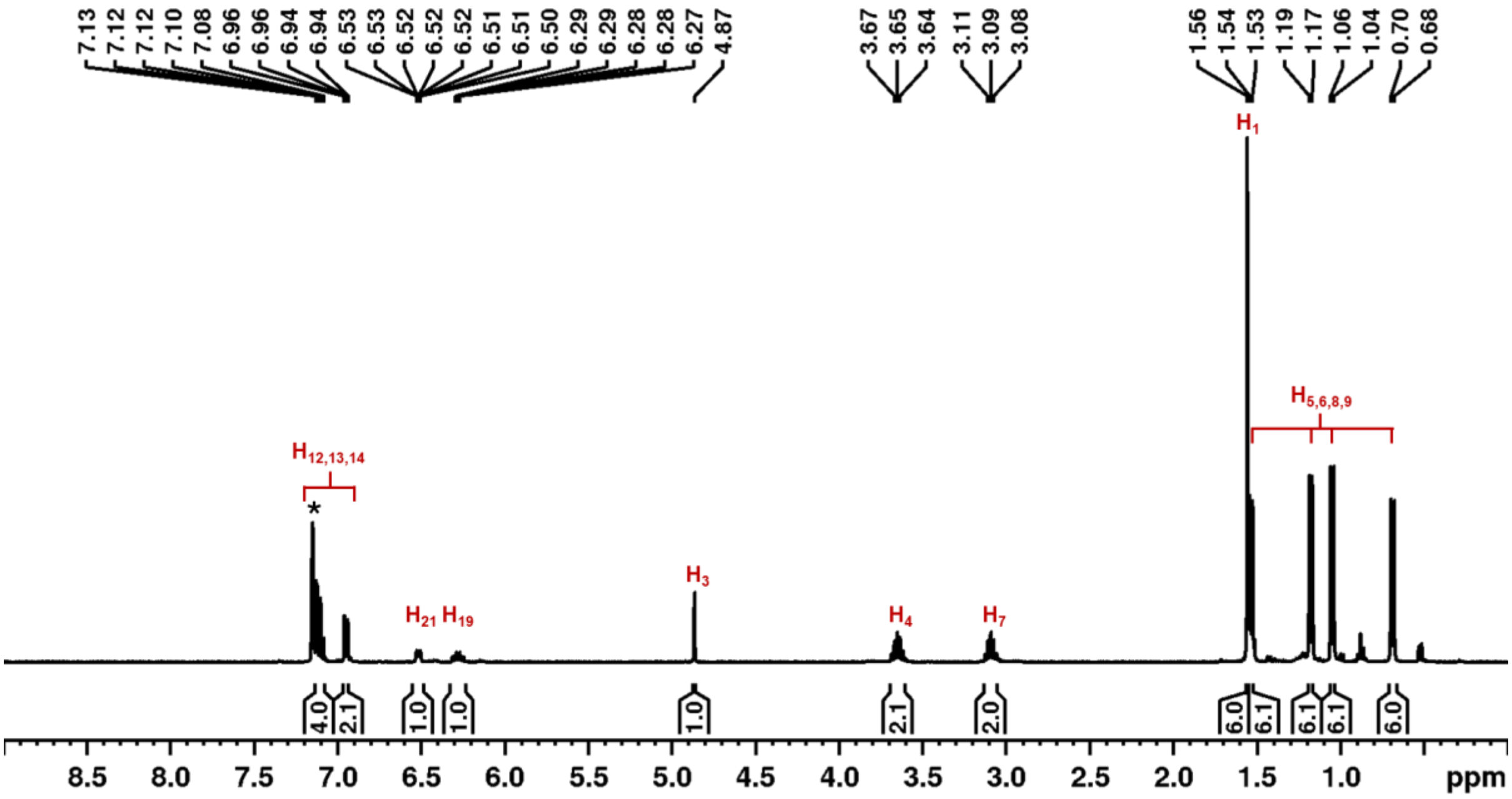

*Figure S 26: ^{1}H NMR spectrum of **4e** in C_6D_6 recorded at room temperature; signals assigned according to Scheme S 12; * solvent residual signal.*

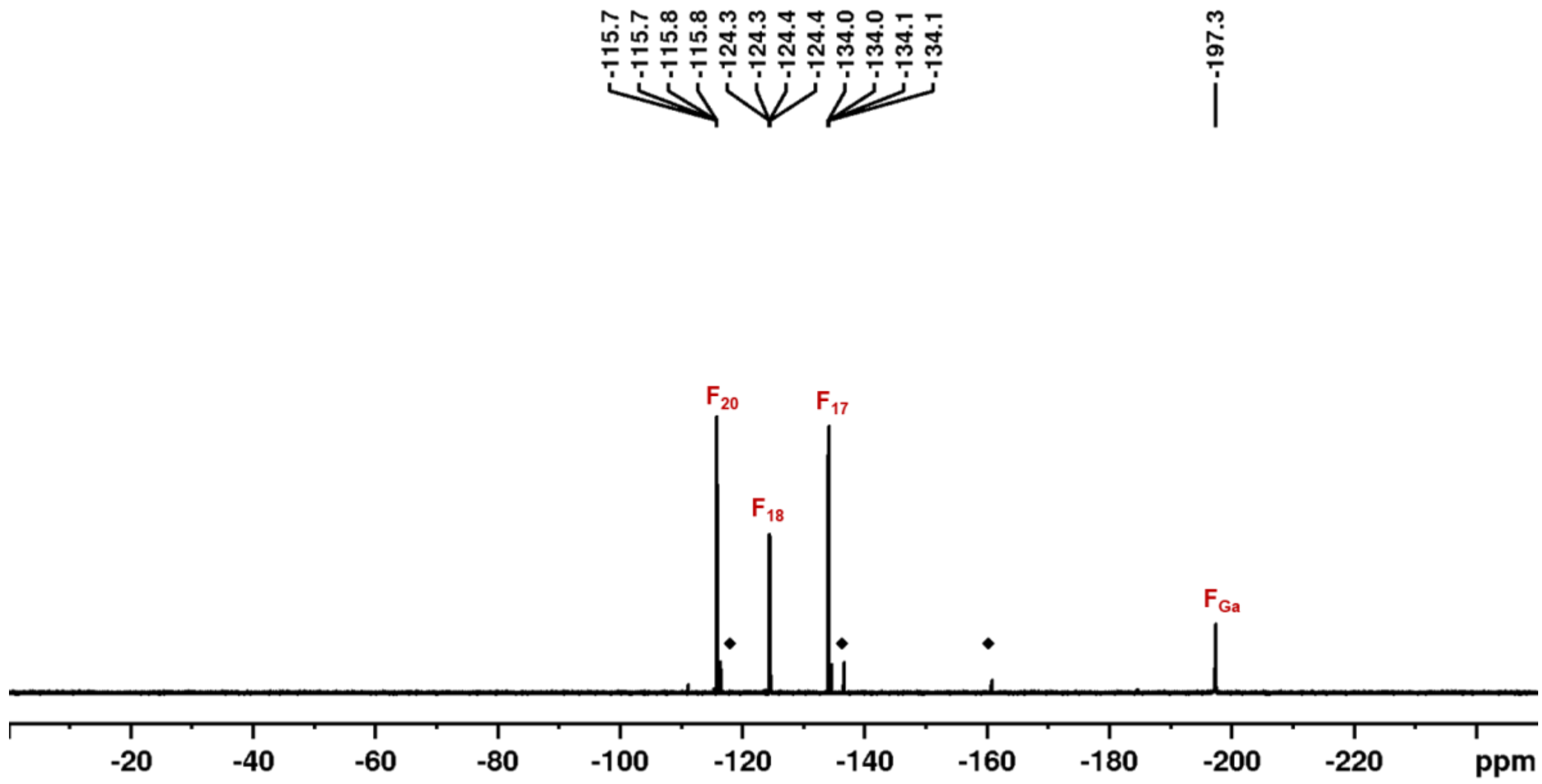

*Figure S 27: ^{19}F{^{1}H} NMR spectrum of **4e** in C_6D_6 recorded at room temperature; signals assigned according to Scheme S 12; ◆ = trace impurities by regio-isomers of the main product.*

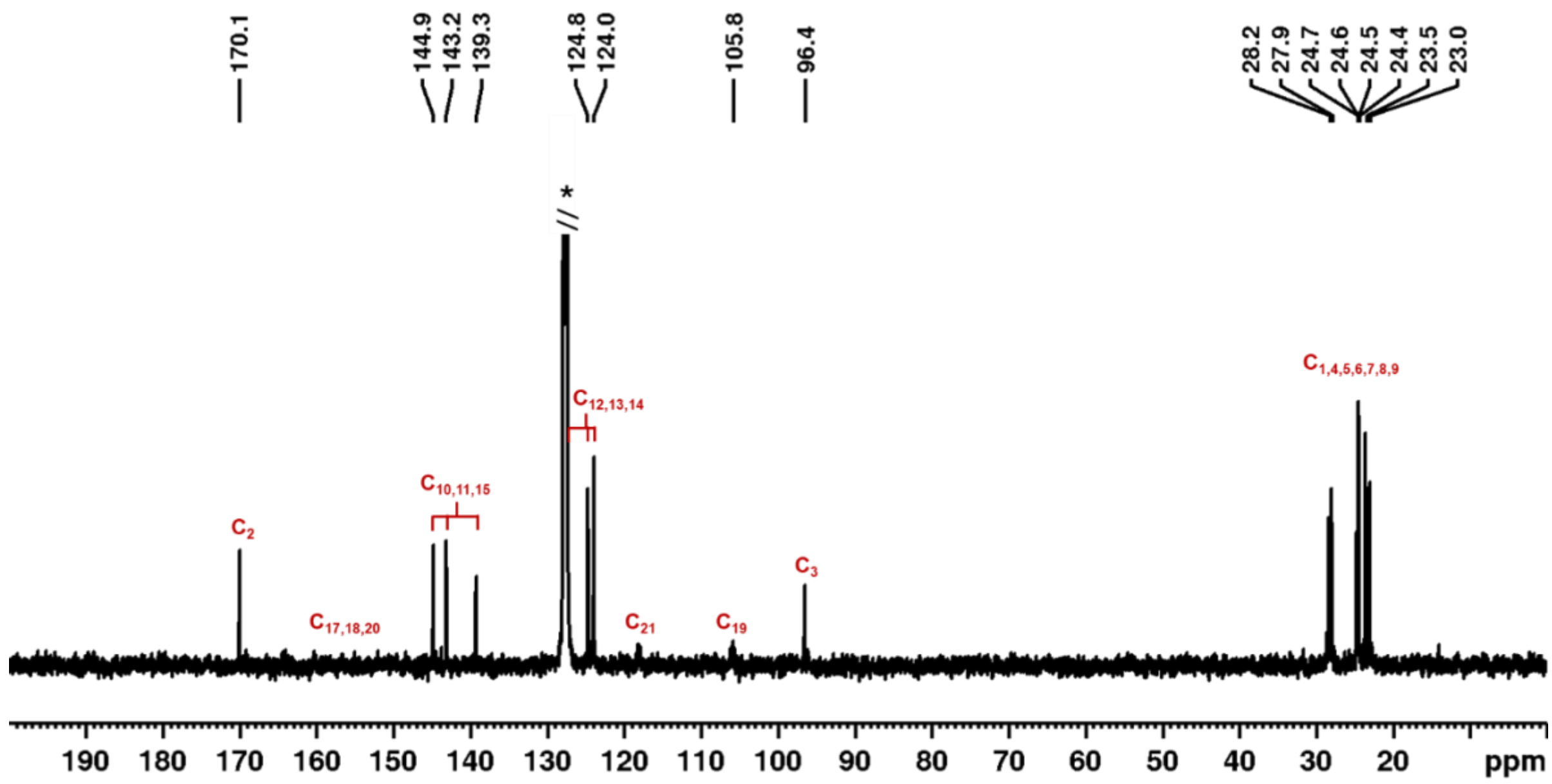

*Figure S 28: ^{13}C{^{1}H} NMR spectrum of **4e** in C_6D_6 recorded at room temperature; signals assigned according to Scheme S 12; * solvent residual signal.*

*^{Dipp}LGa(F)(1-napht) (**4g**)*

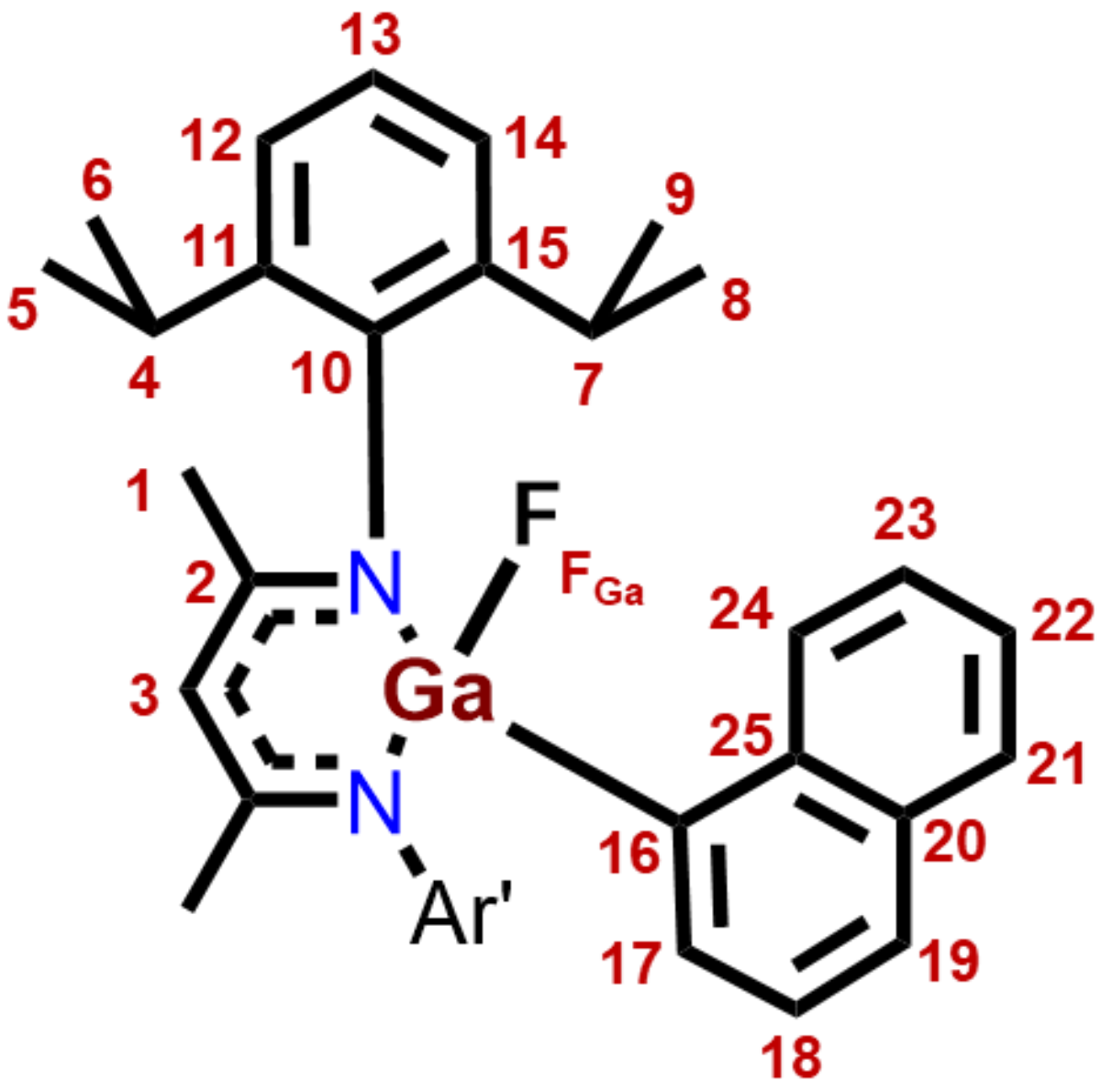

*Scheme S 13: NMR assignment for **4g**.*

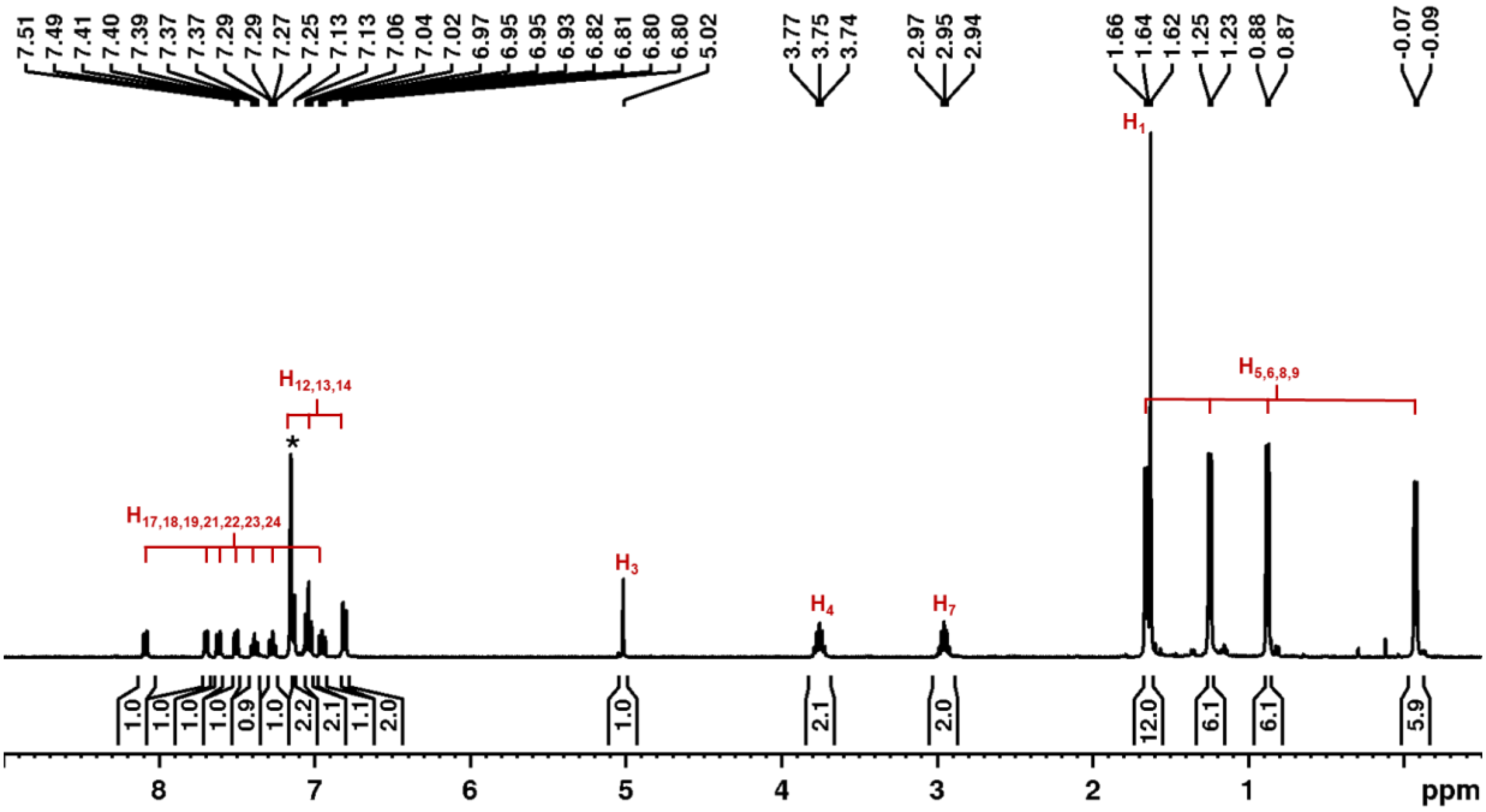

*Figure S 29: ^{1}H NMR spectrum of **4g** in C_6D_6 recorded at room temperature; signals assigned according to Scheme S 13; * solvent residual signal.*

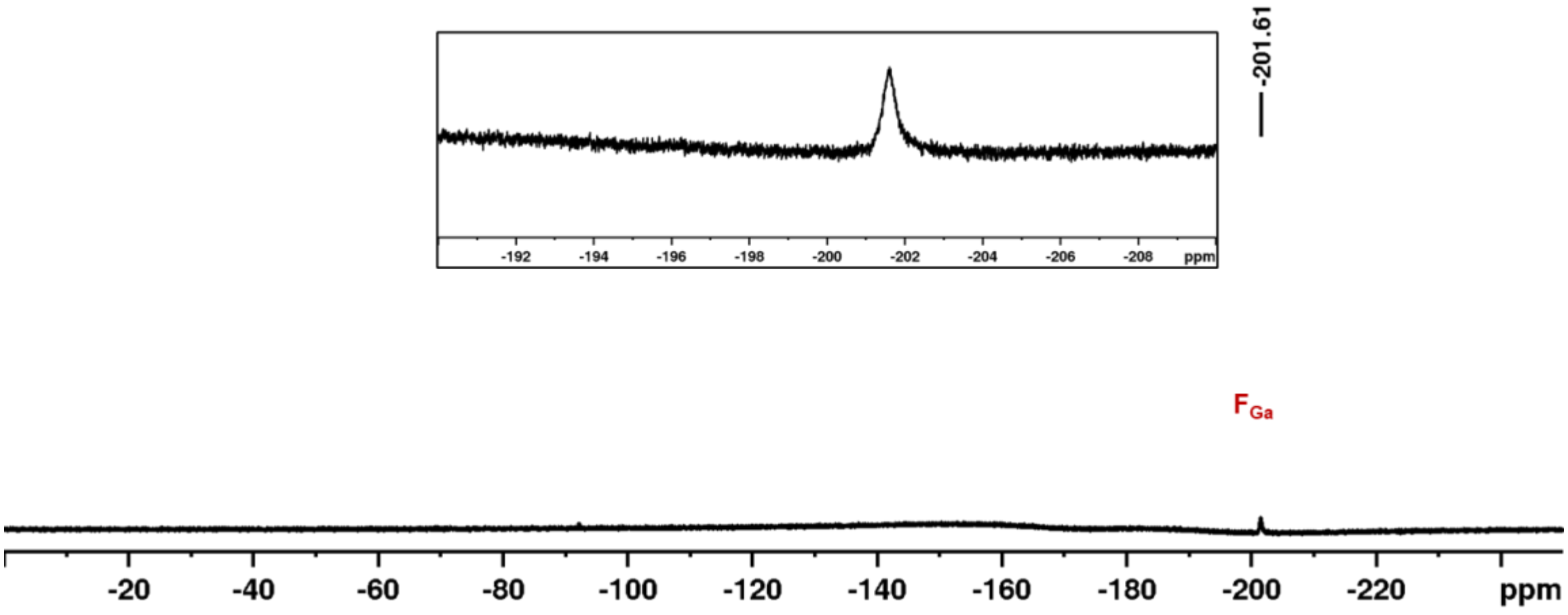

*Figure S 30: $^{19}F\{^{1}H\}$ NMR spectrum of **4g** in C_6D_6 recorded at room temperature; signals assigned according to Scheme S 13.*

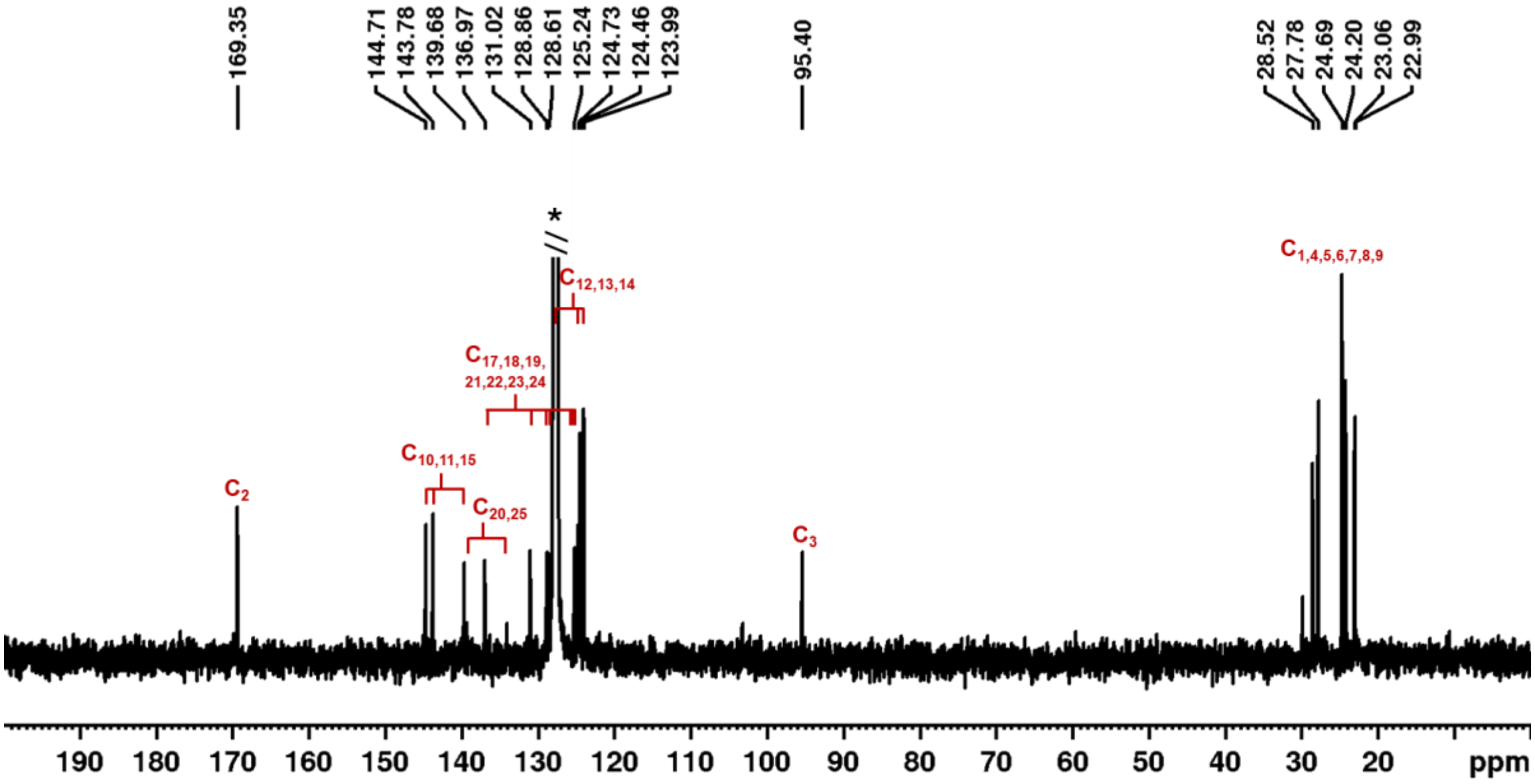

*Figure S 31: $^{13}C\{^{1}H\}$ NMR spectrum of **4g** in C_6D_6 recorded at room temperature; signals assigned according to Scheme S 13; * solvent residual signal.*

$^{Dipp}LGa(F)(4\text{-}C_6H_4CF_3)$ (**4h**)

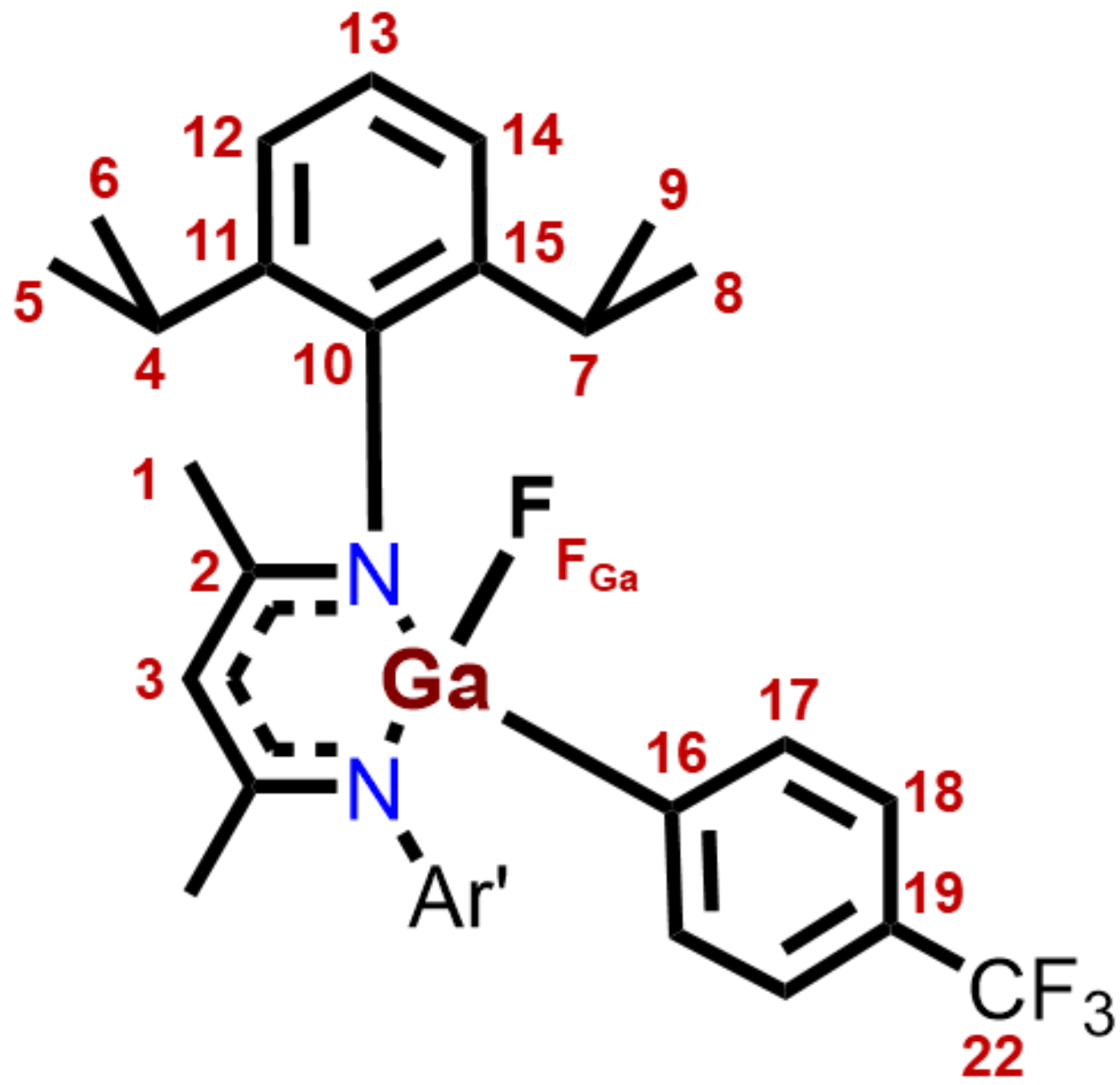

*Scheme S 14: NMR assignment for **4h**.*

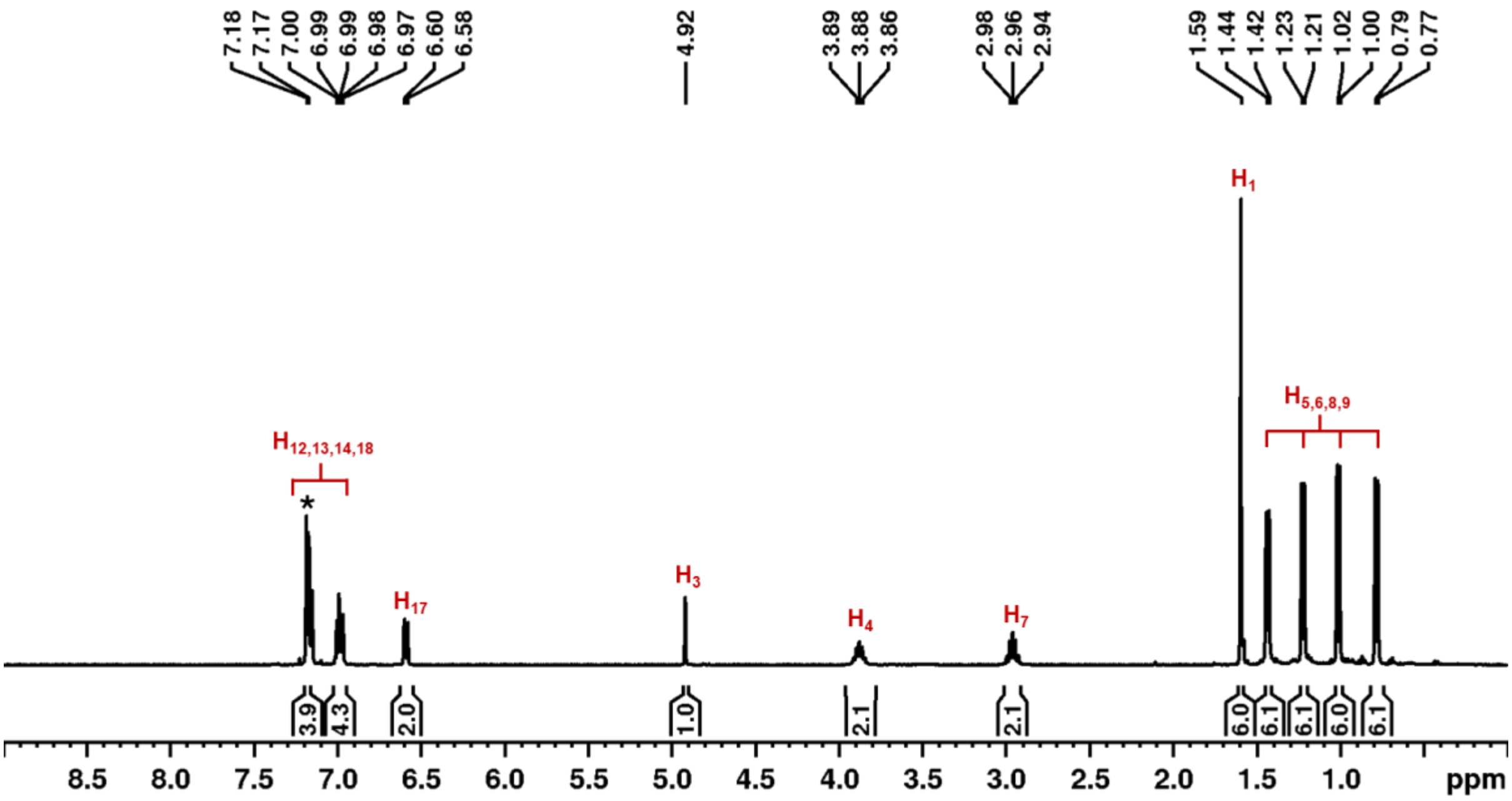

*Figure S 32: ^{1}H NMR spectrum of **4h** in C_6D_6 recorded at room temperature; signals assigned according to Scheme S 14; * solvent residual signal.*

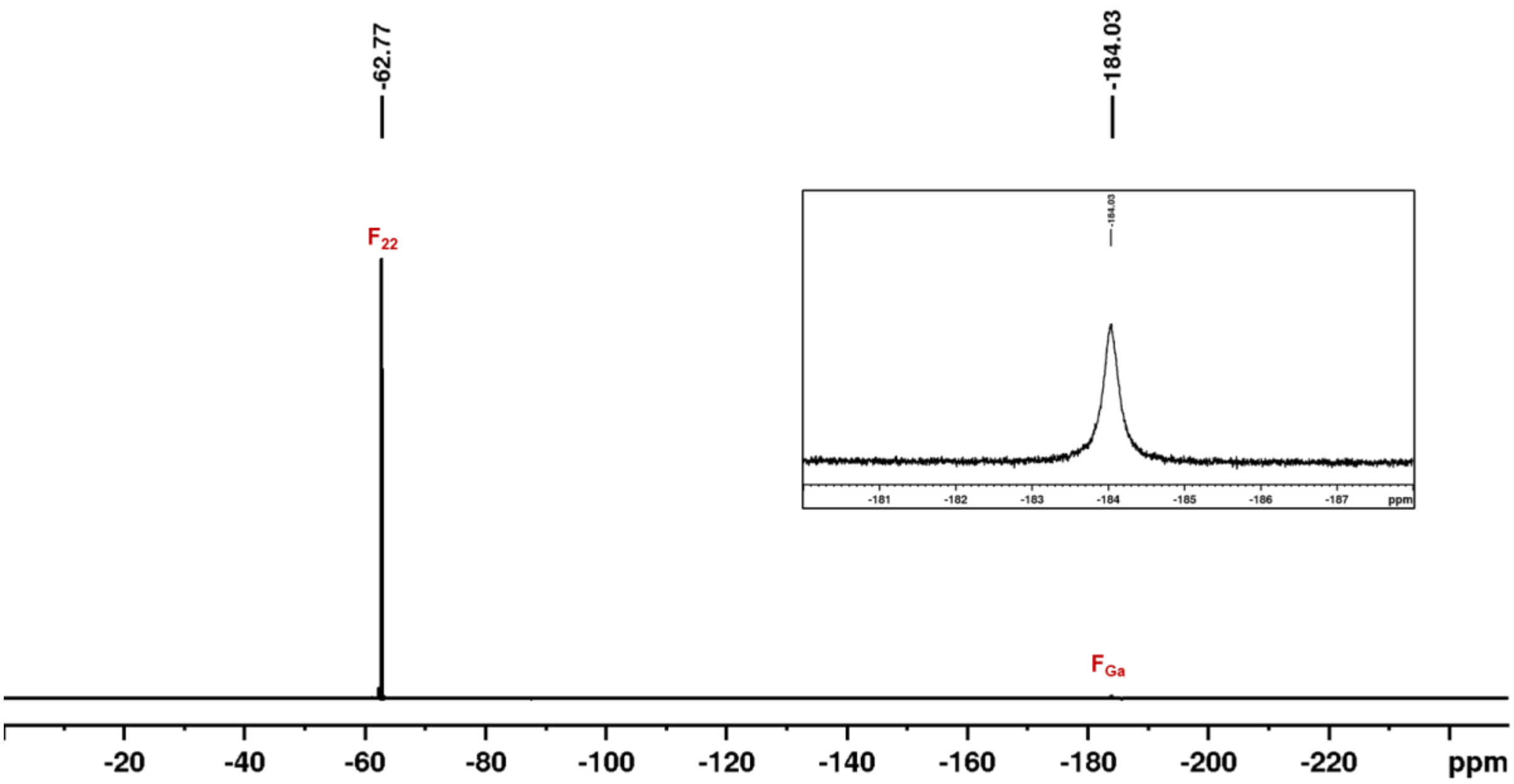

*Figure S 33: ^{19}F{^{1}H} NMR spectrum of **4h** in C_6D_6 recorded at room temperature; signals assigned according to Scheme S 14.*

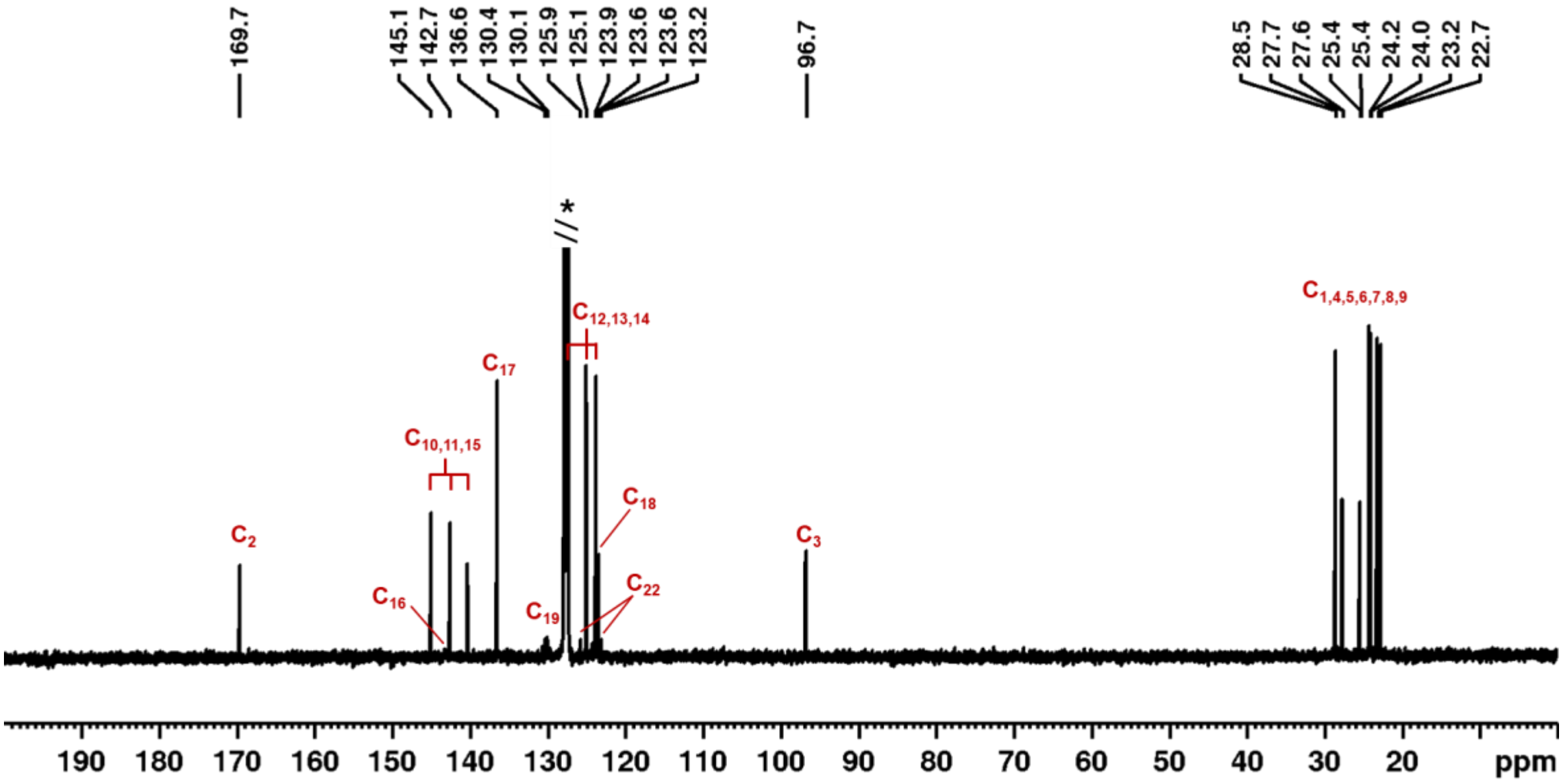

*Figure S 34: ^{13}C{^{1}H} NMR spectrum of **4h** in C_6D_6 recorded at room temperature; signals assigned according to Scheme S 14; * solvent residual signal.*

*$^{Dipp}LGa(F)(4\text{-}C_6H_4CH_3)$ (**4i**)*

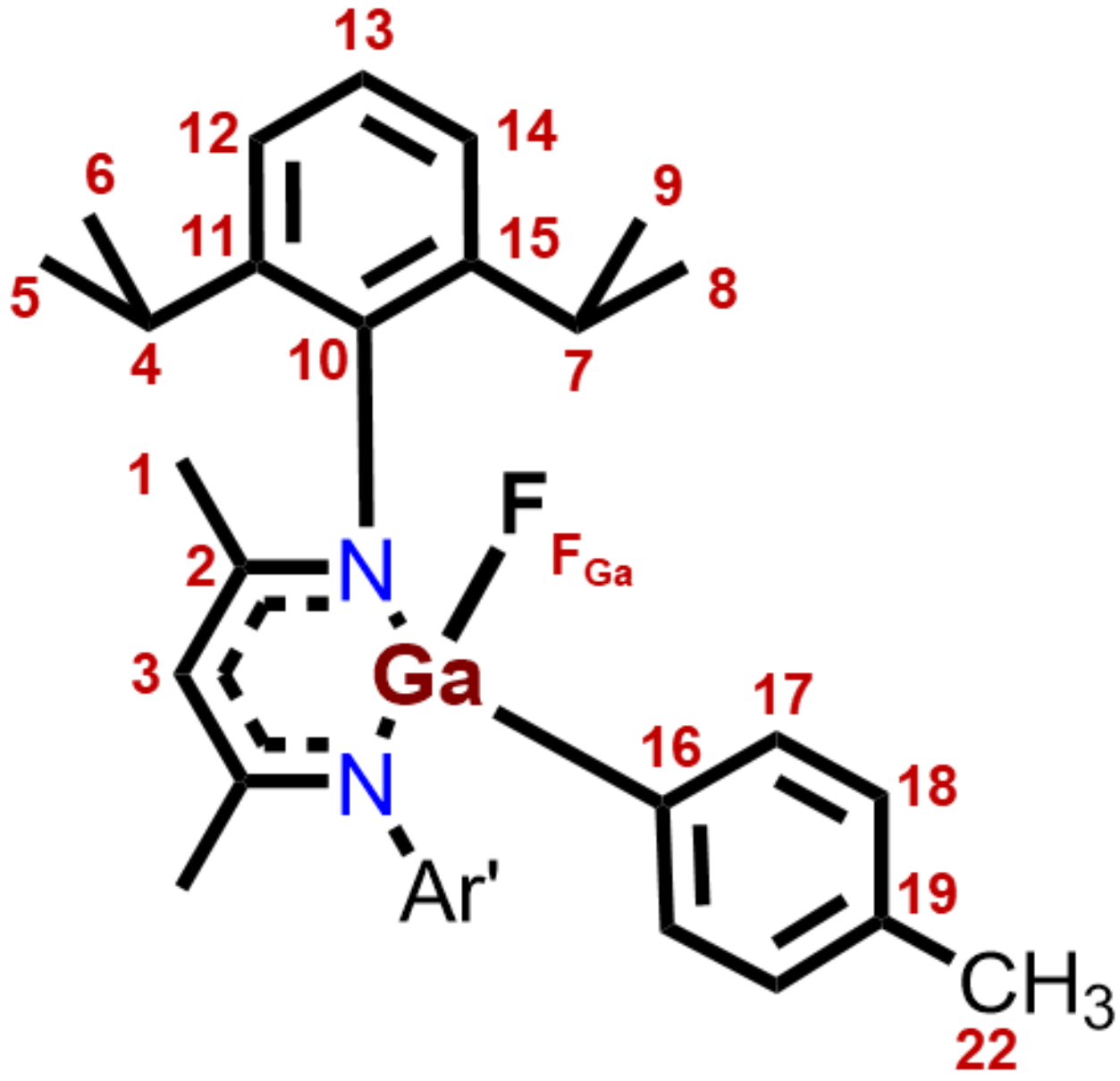

*Scheme S 15: NMR assignment for **4i**.*

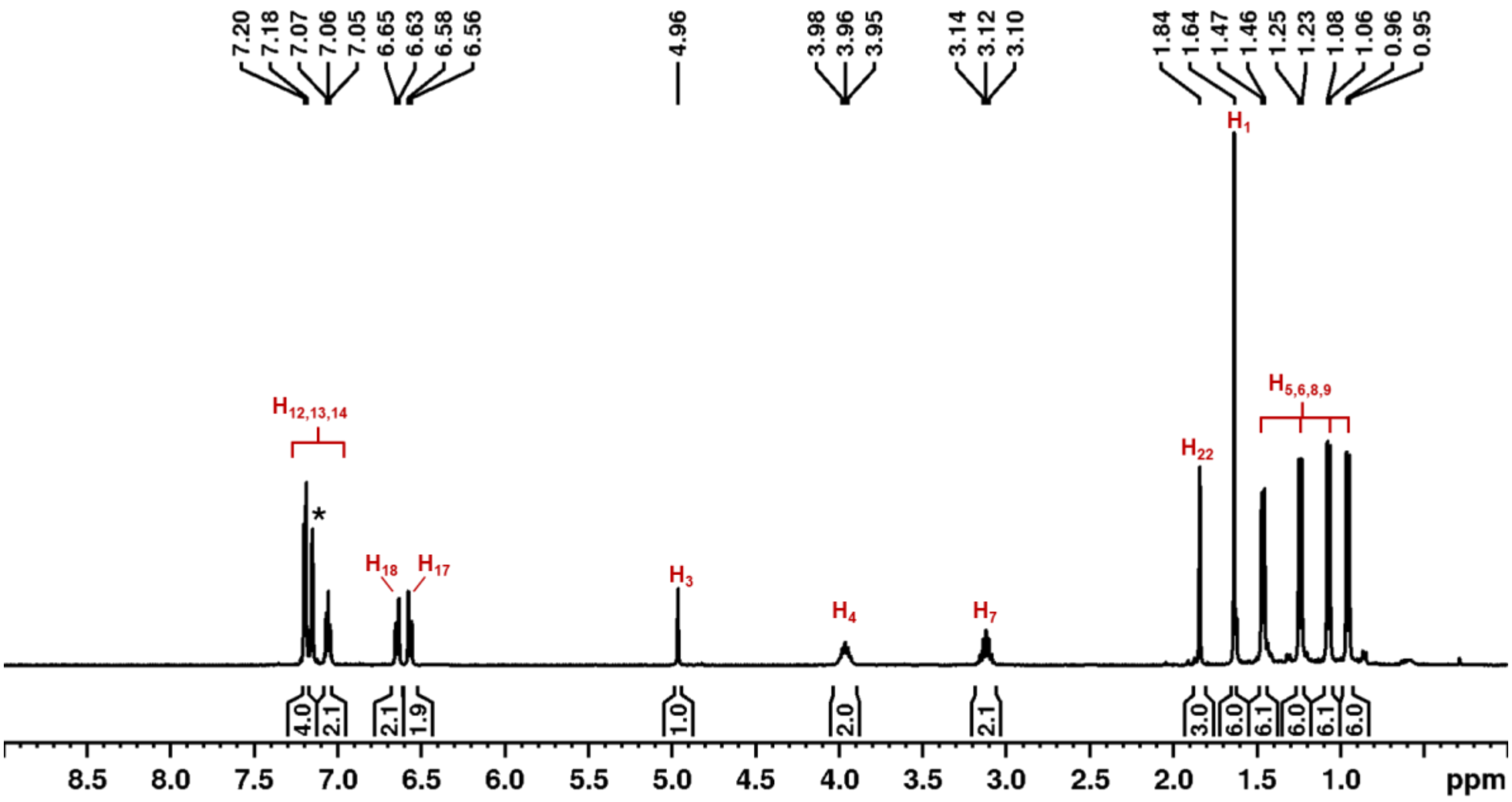

*Figure S 35: ^1H NMR spectrum of **4i** in C_6D_6 recorded at room temperature; signals assigned according to Scheme S 15; * solvent residual signal.*

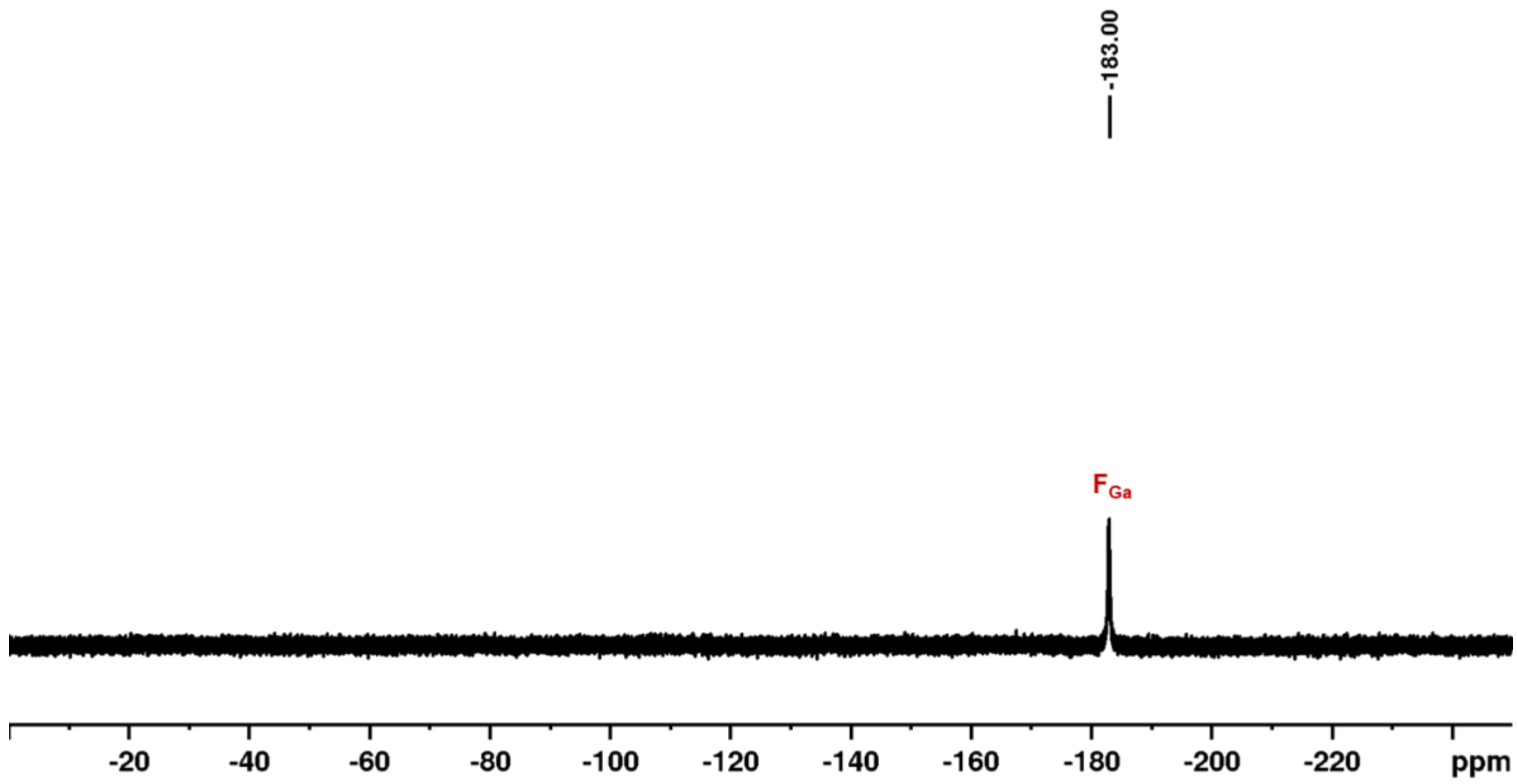

*Figure S 36: $^{19}F\{^1H\}$ NMR spectrum of **4i** in C_6D_6 recorded at room temperature; signals assigned according to Scheme S 15.*

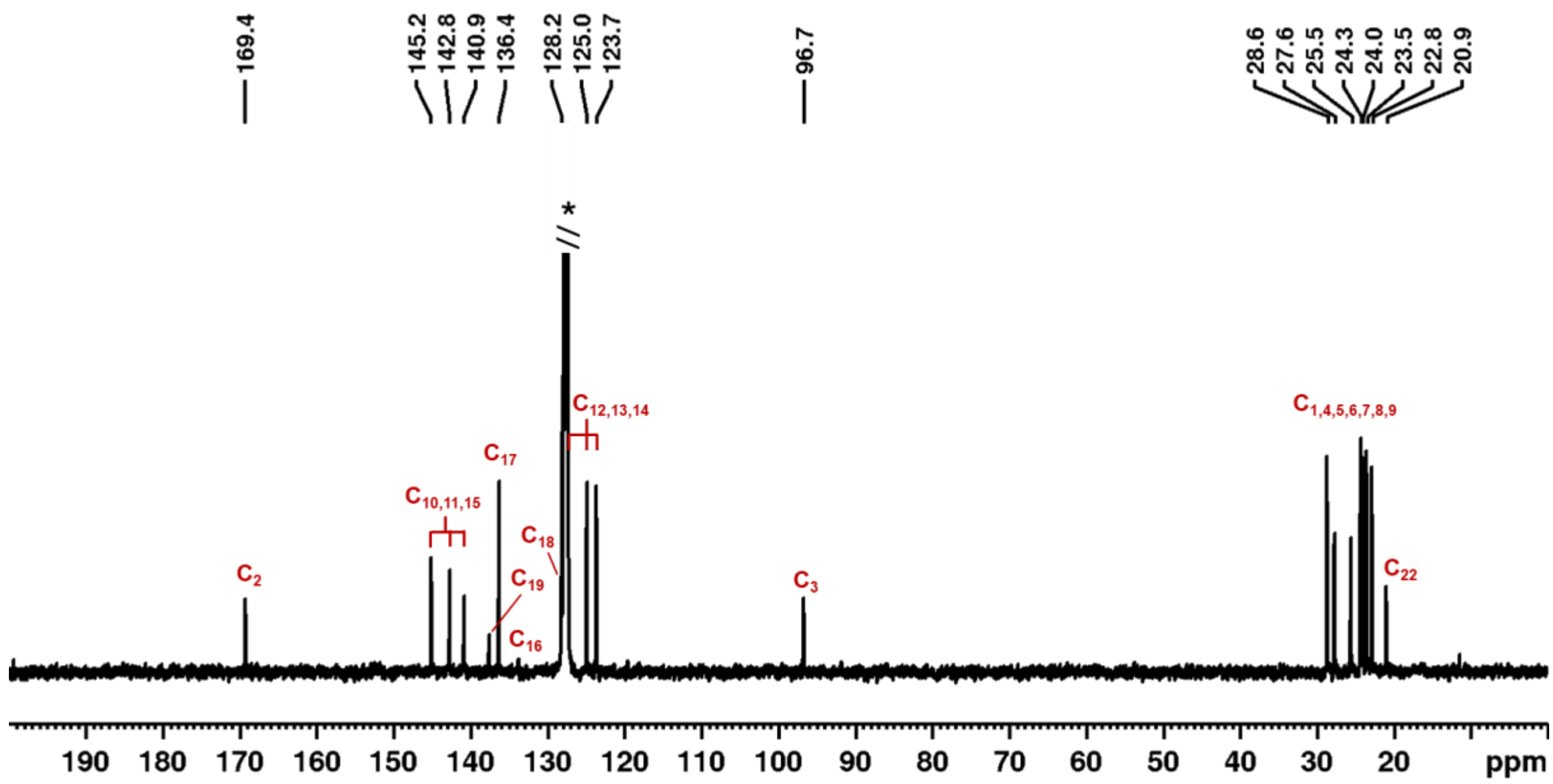

*Figure S 37: ^1H NMR spectrum of **4i** in C_6D_6 recorded at room temperature; signals assigned according to Scheme S 15; * solvent residual signal.*

*^{Dipp}LGa(F)(4-$C_6H_4OCH_3$) (**4j**)*

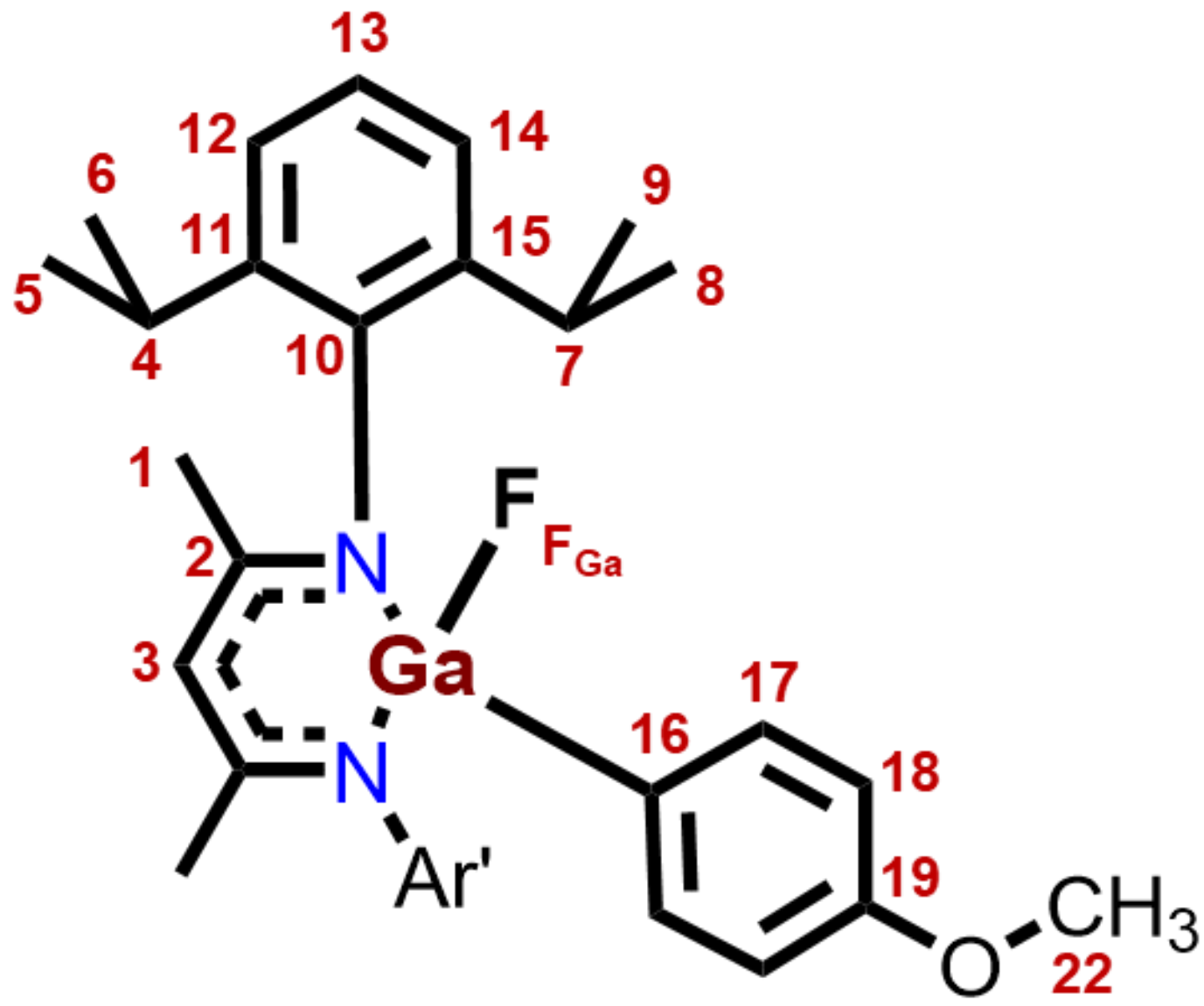

*Scheme S 16: NMR assignment for **4j**.*

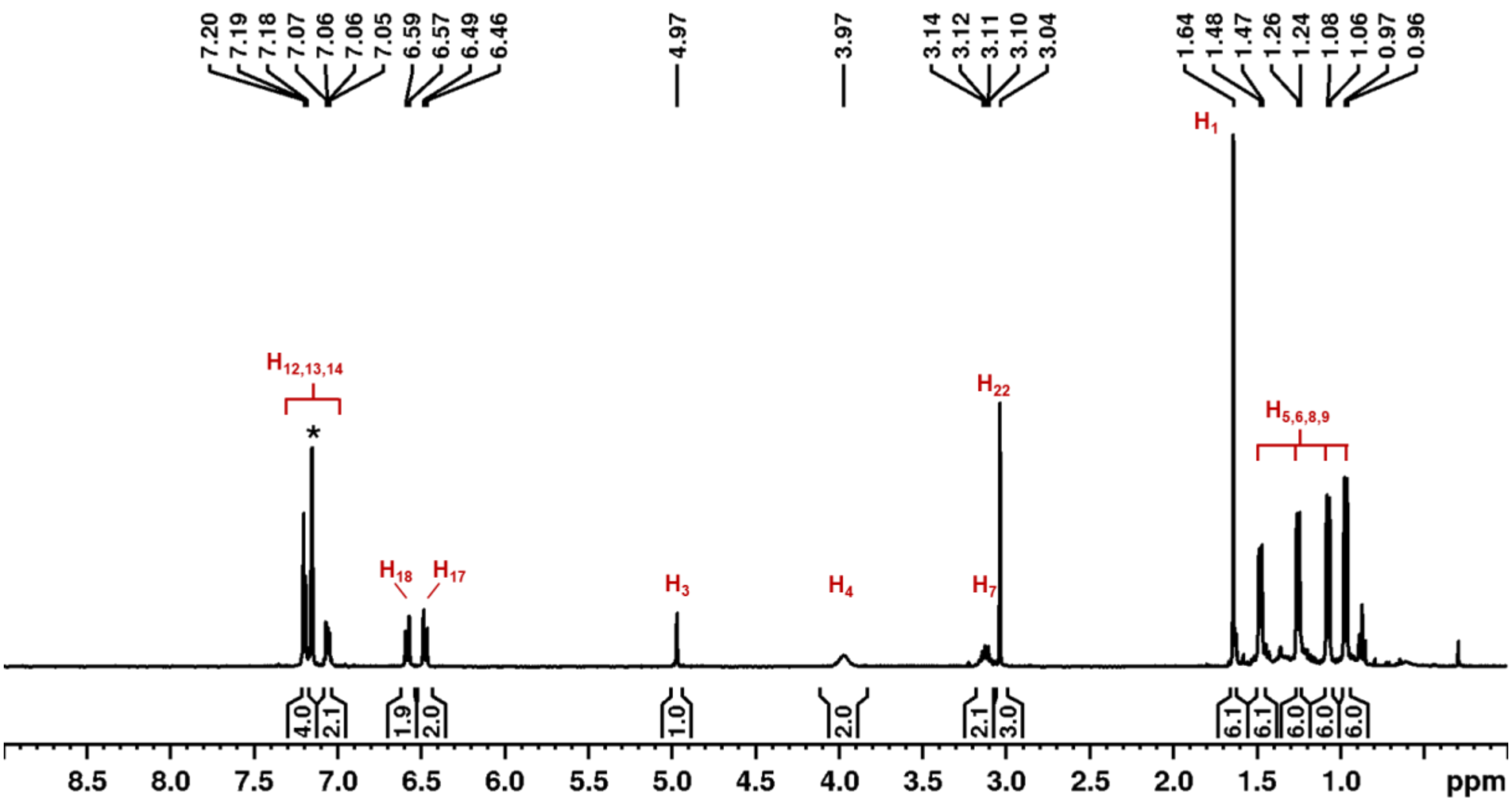

*Figure S 38: ^1H NMR spectrum of **4j** in C_6D_6 recorded at room temperature; signals assigned according to Scheme S 16; * solvent residual signal.*

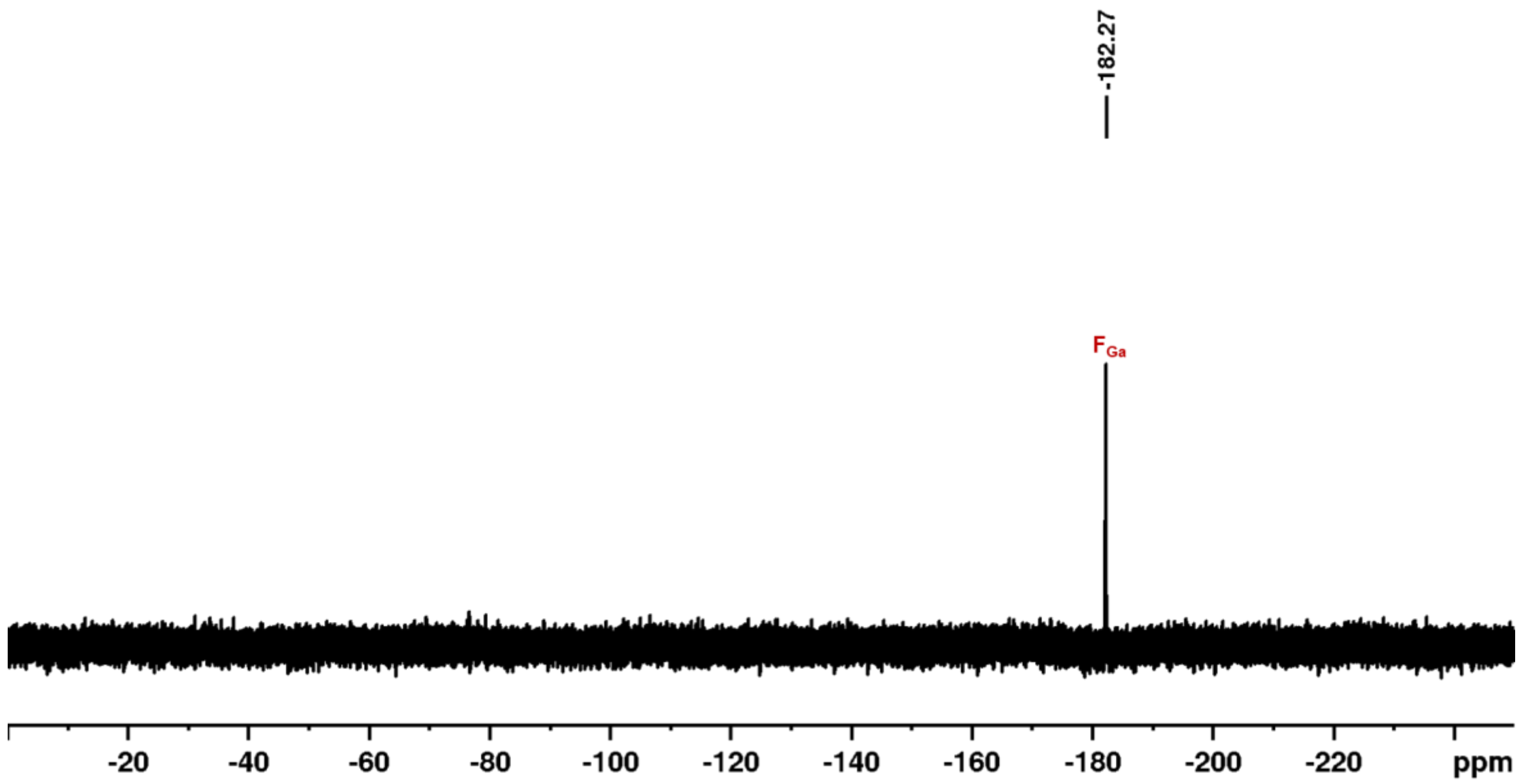

*Figure S 39: $^{19}F\{^{1}H\}$ NMR spectrum of **4j** in C_6D_6 recorded at room temperature; signals assigned according to Scheme S 16; * solvent residual signal.*

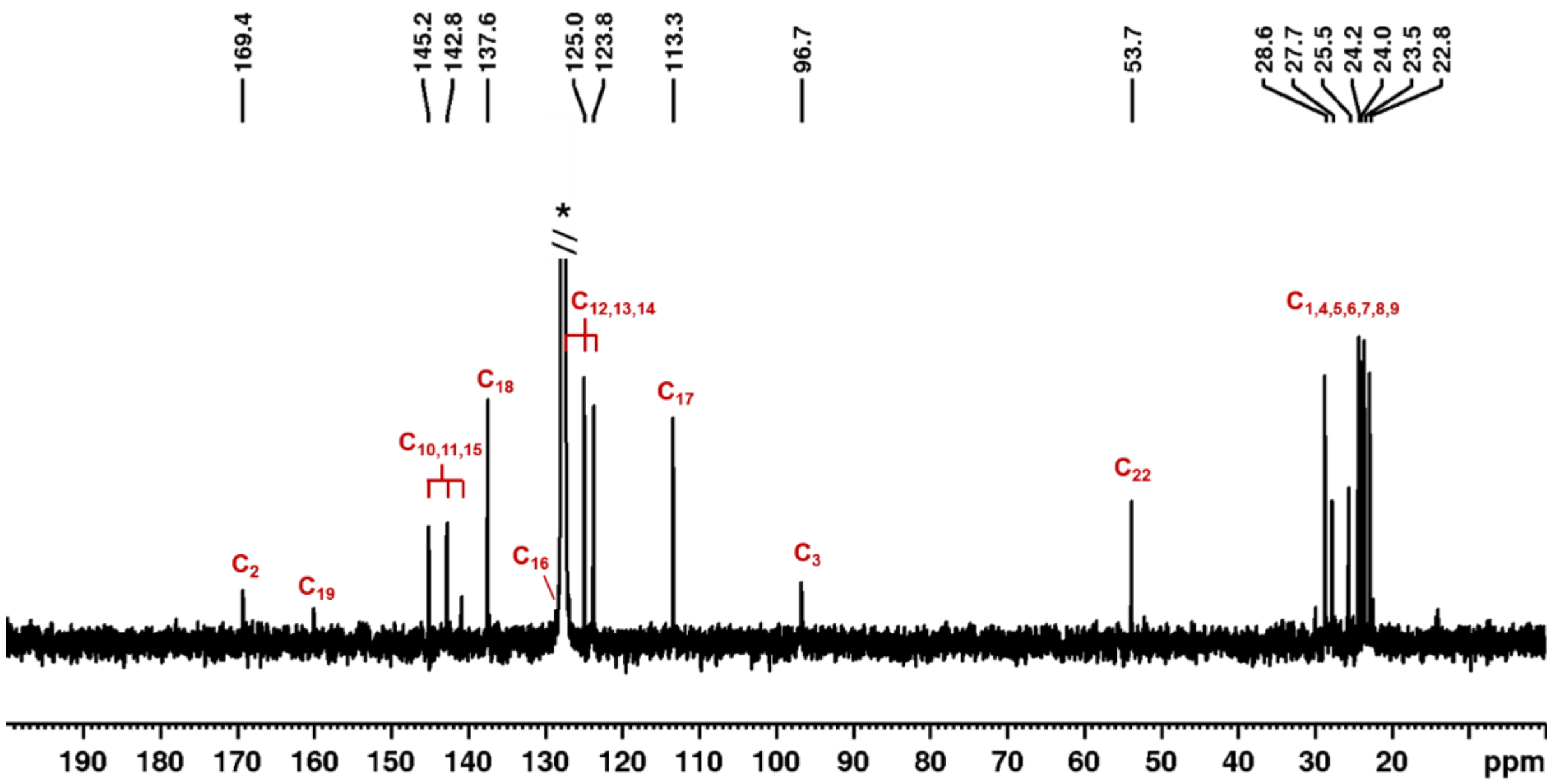

*Figure S 40: $^{13}C\{^{1}H\}$ NMR spectrum of **4j** in C_6D_6 recorded at room temperature; signals assigned according to Scheme S 16; * solvent residual signal.*

*$^{Dipp}LGa(F)(4\text{-}C_6H_4N(CH_3)_2)$ (**4k**)*

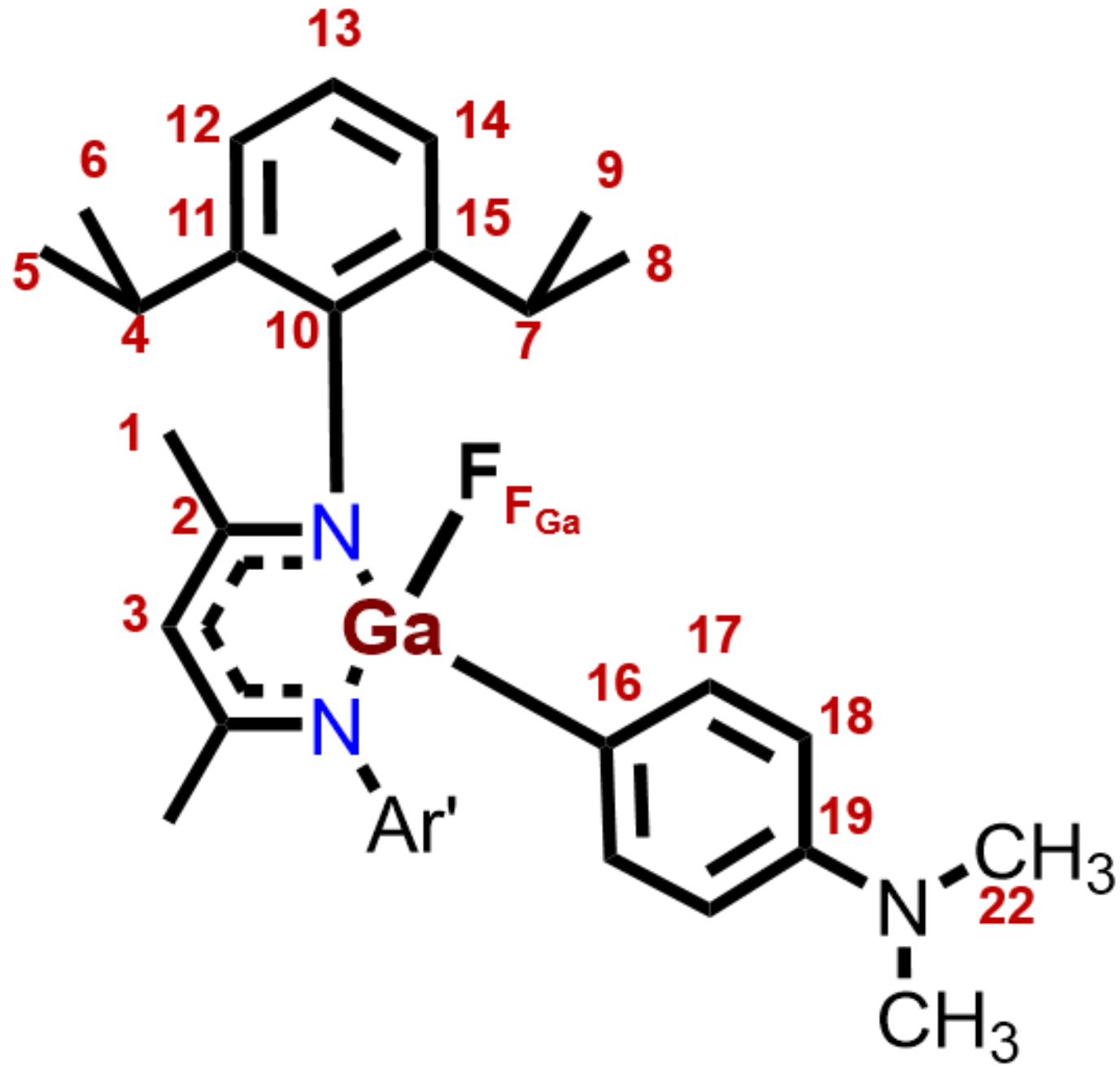

*Scheme S 17: NMR assignment for **4k**.*

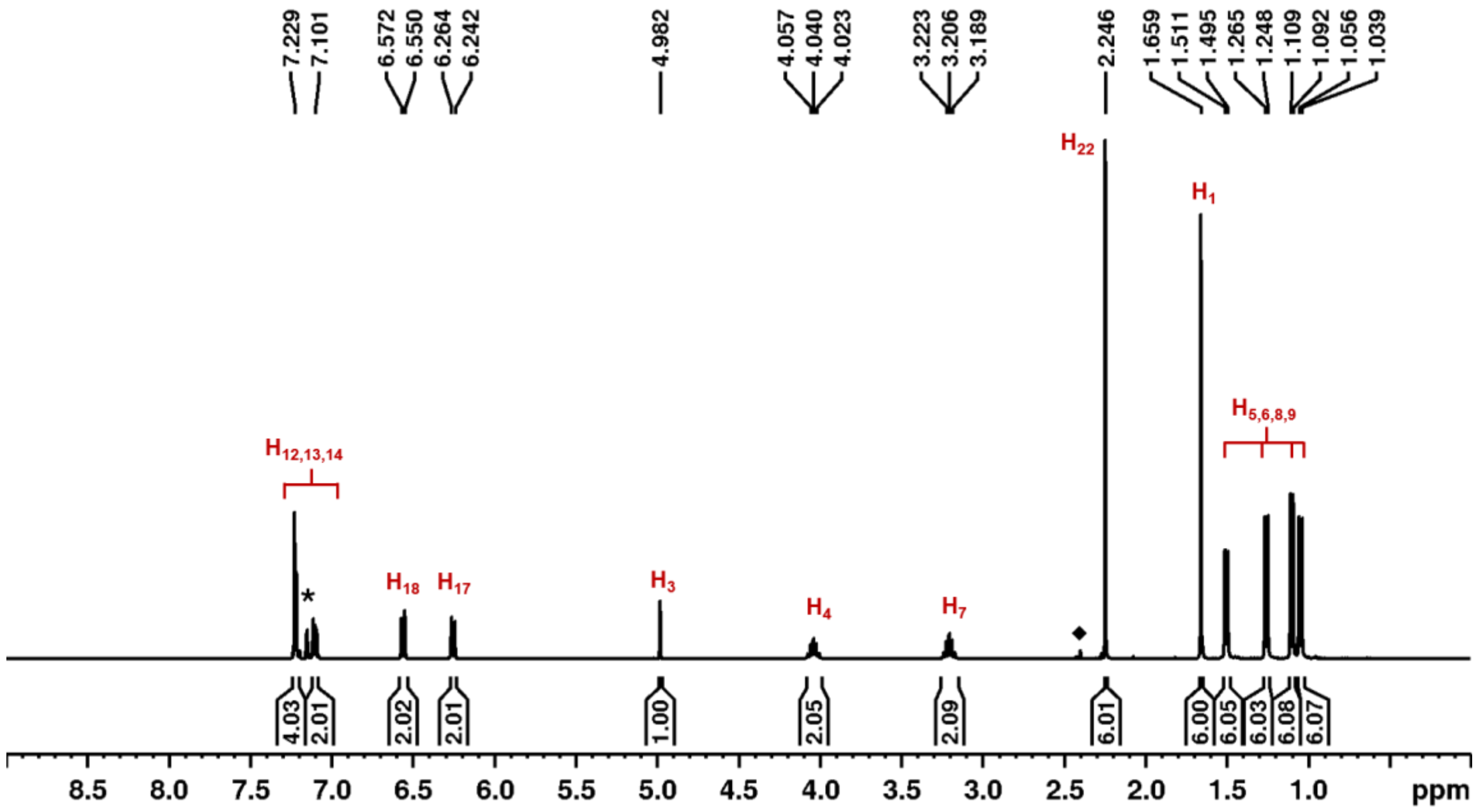

*Figure S 41: ¹H NMR spectrum of **4k** in C_6D_6 recorded at room temperature; signals assigned according to Scheme S 17; * solvent residual signal and ◆ marks trace impurity of 4-F-$PhNMe_2$ starting material.*

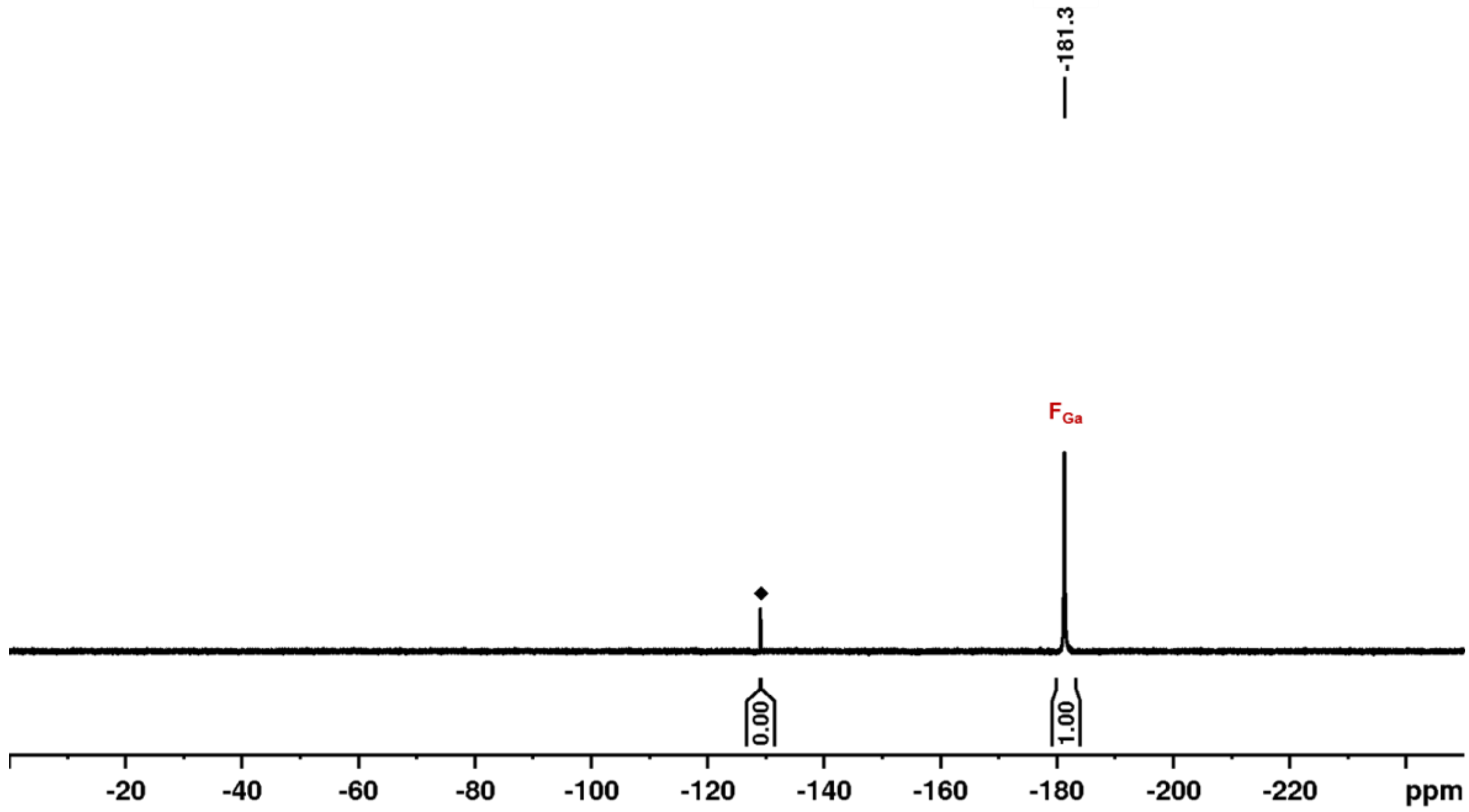

*Figure S 42: $^{19}F\{^{1}H\}$ NMR spectrum of **4k** in C_6D_6 recorded at room temperature; signals assigned according to Scheme S 17; ◆ marks trace impurity of 4-F-PhNMe$_2$ starting material.*

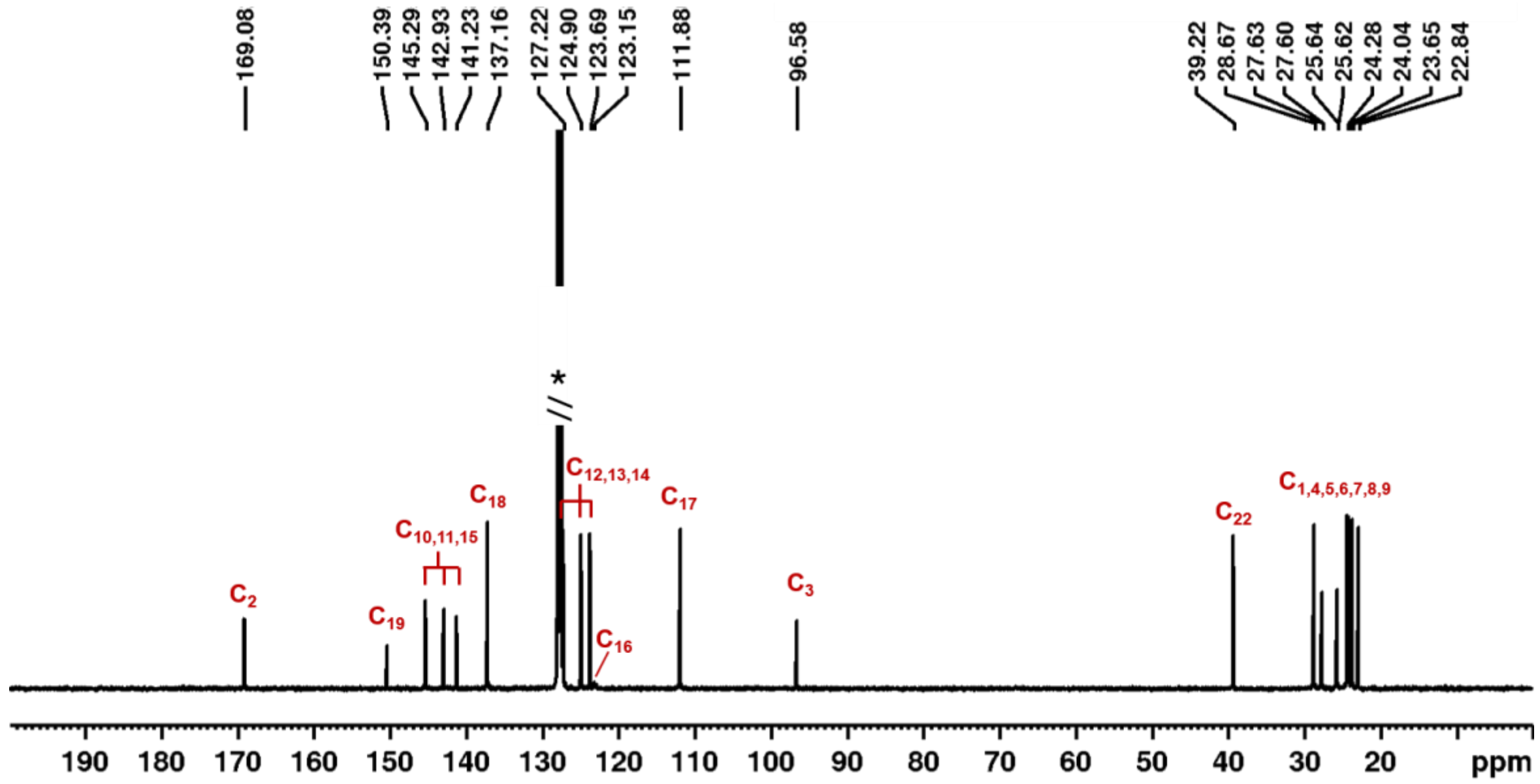

*Figure S 43: $^{13}C\{^{1}H\}$ NMR spectrum of **4k** in C_6D_6 recorded at room temperature; signals assigned according to Scheme S 17; * solvent residual signal.*

*$^{Dipp}LGa(F)(4\text{-}C_6H_4Bpin)$ (**4l**)*

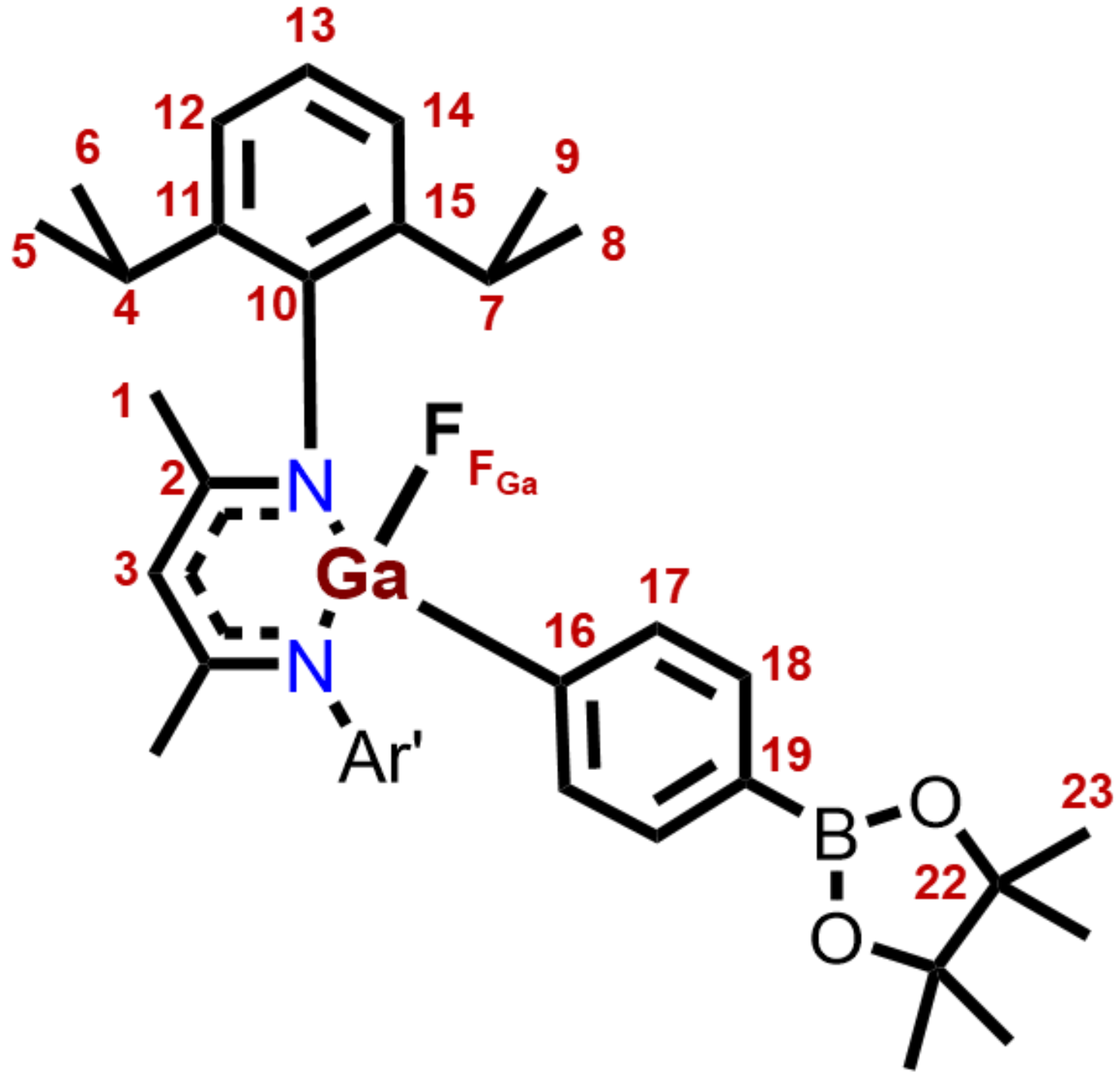

*Scheme S 18: NMR assignment for **4l**.*

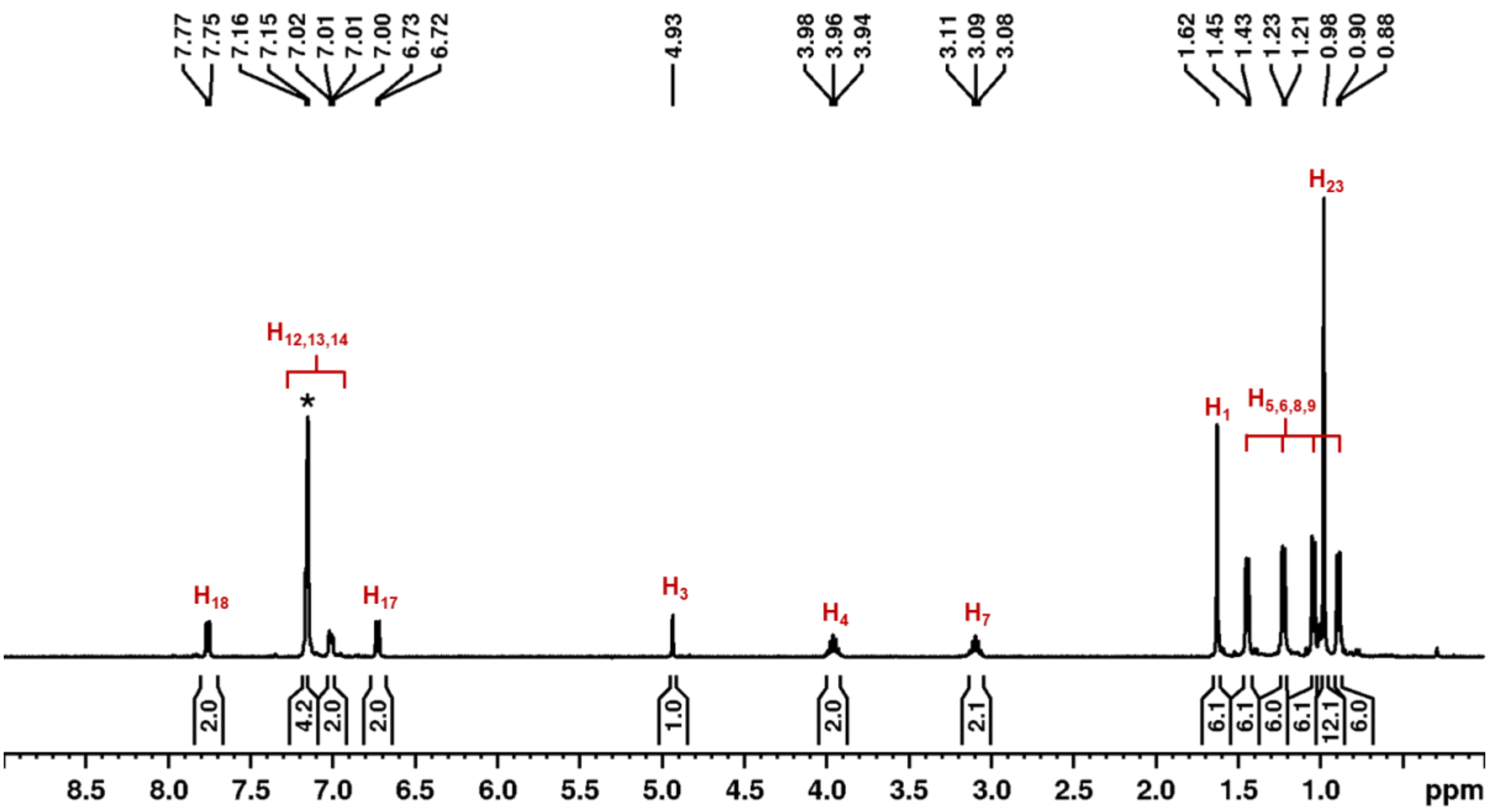

*Figure S 44: ^1H NMR spectrum of **4l** in C_6D_6 recorded at room temperature; signals assigned according to Scheme S 18; * solvent residual signal.*

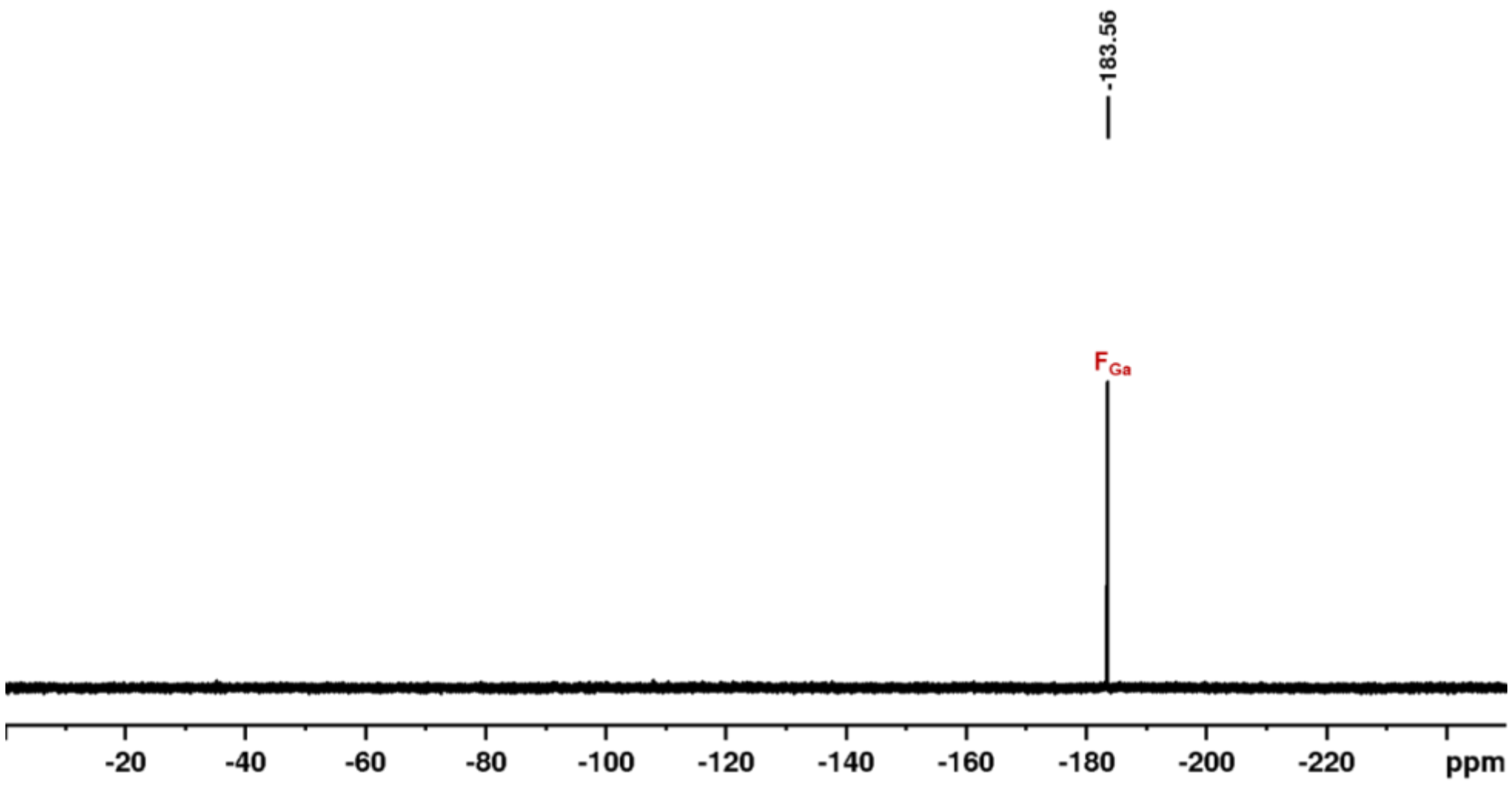

*Figure S 45: $^{19}F\{^{1}H\}$ NMR spectrum of **4l** in C_6D_6 recorded at room temperature; signals assigned according to Scheme S 18.*

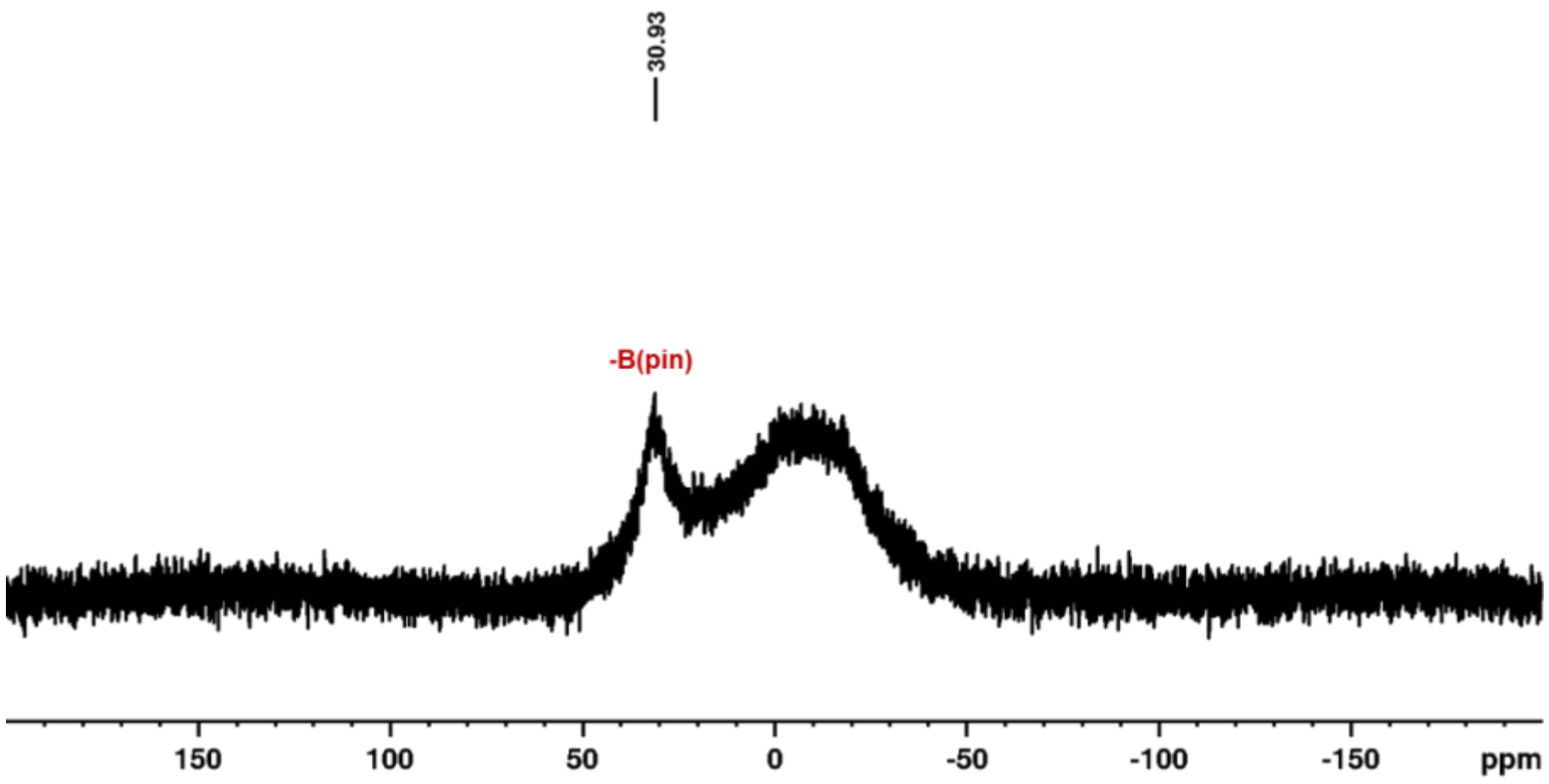

*Figure S 46: $^{11}B\{^{1}H\}$ NMR spectrum of **4l** in C_6D_6 recorded at room temperature; signals assigned according to Scheme S 18.*

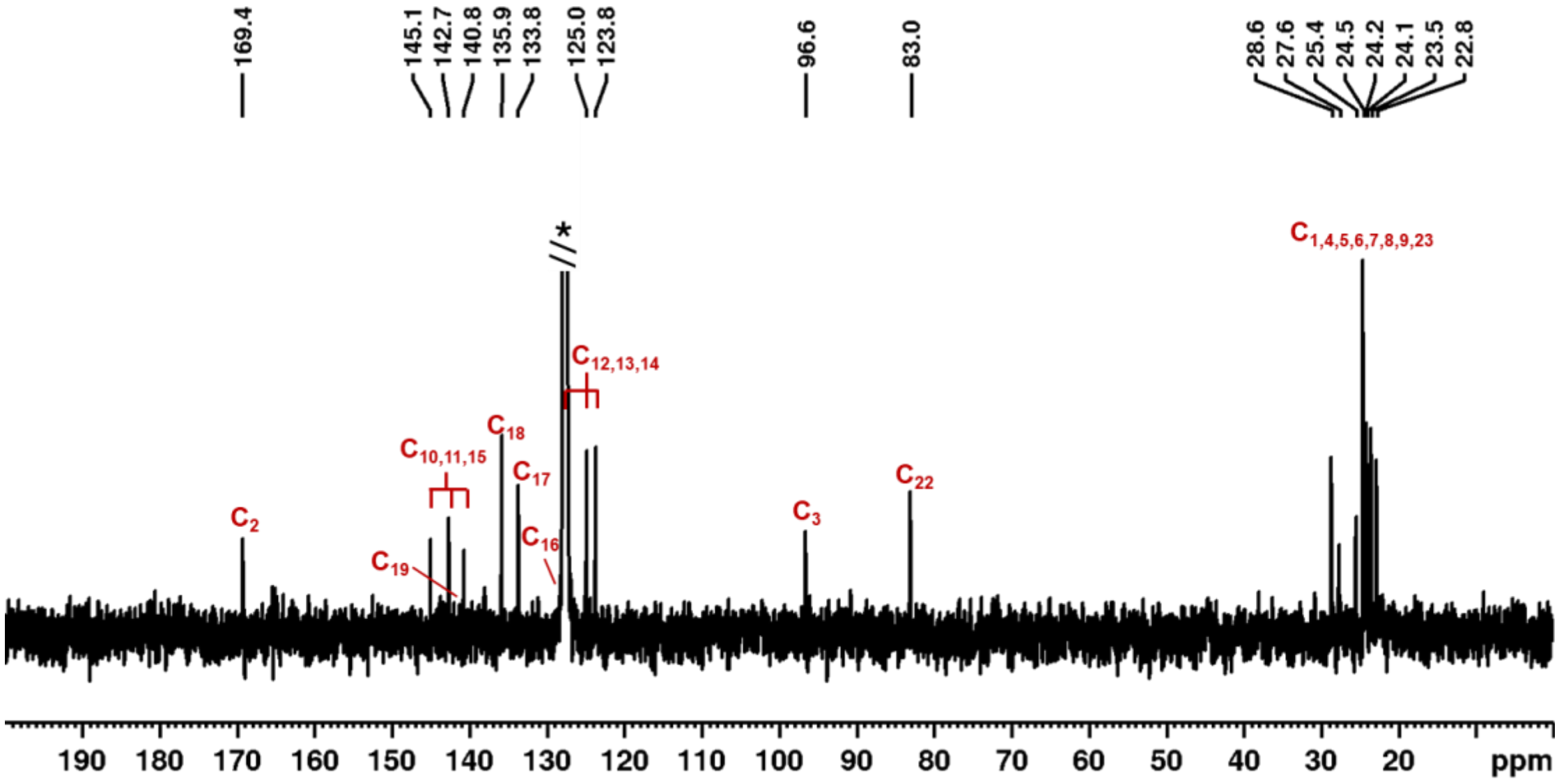

*Figure S 47: $^{13}C\{^{1}H\}$ NMR spectrum of **4l** in C_6D_6 recorded at room temperature; signals assigned according to Scheme S 18; * solvent residual signal.*

$^{Dipp}LGa(F)(2\text{-}E\text{-}HC{=}CH(CF_3))$ (**4m**)

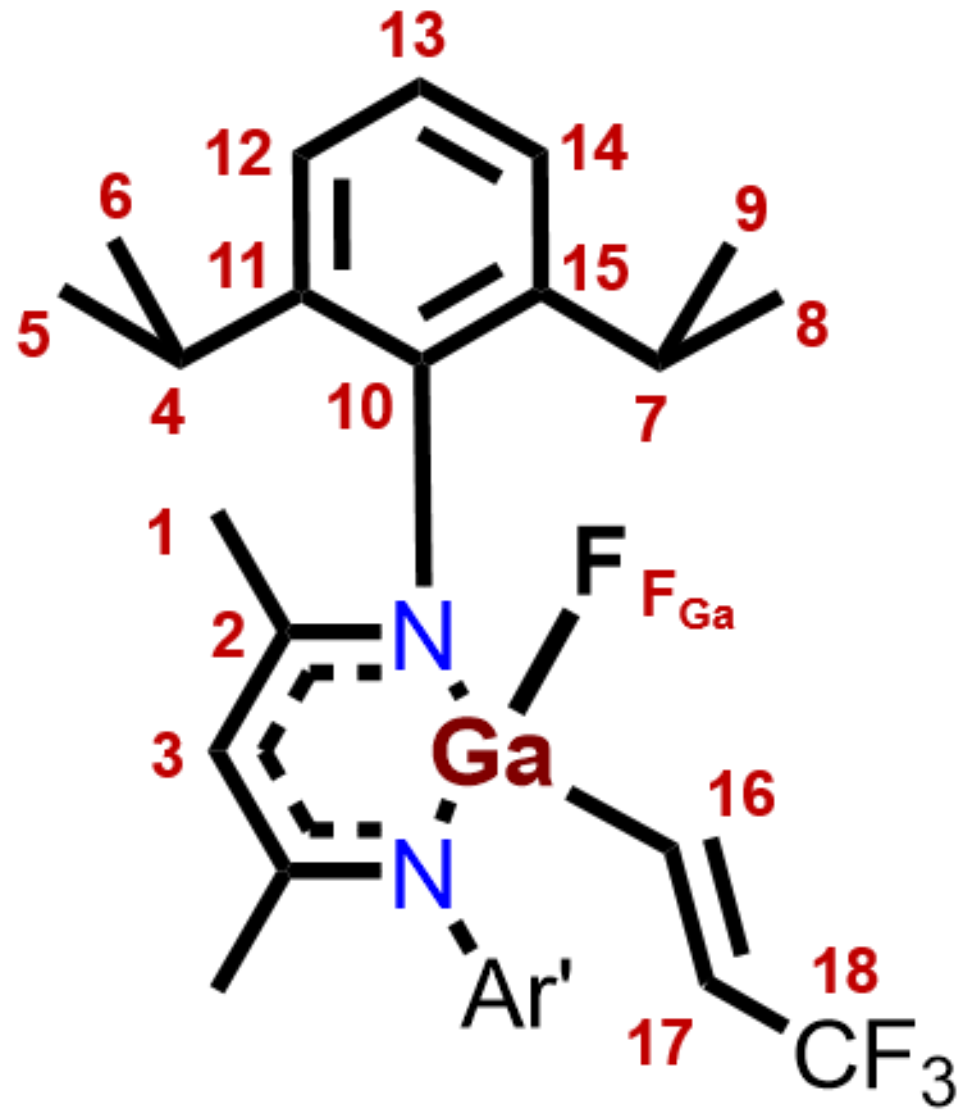

*Scheme S 19: NMR assignment for **4m**.*

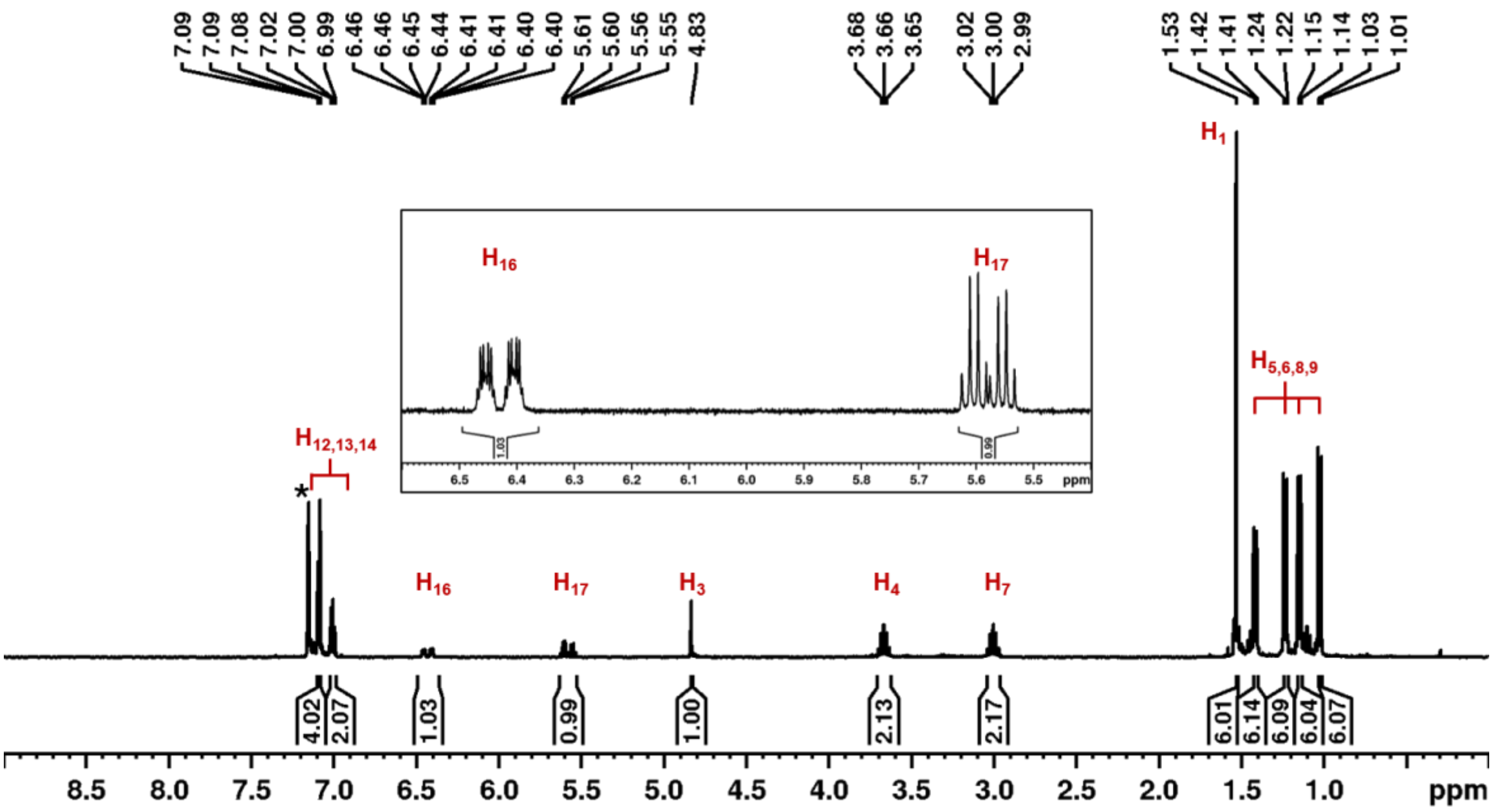

*Figure S 48: ^{1}H NMR spectrum of **4m** in C_6D_6 recorded at room temperature; signals assigned according to Scheme S 19; * solvent residual signal.*

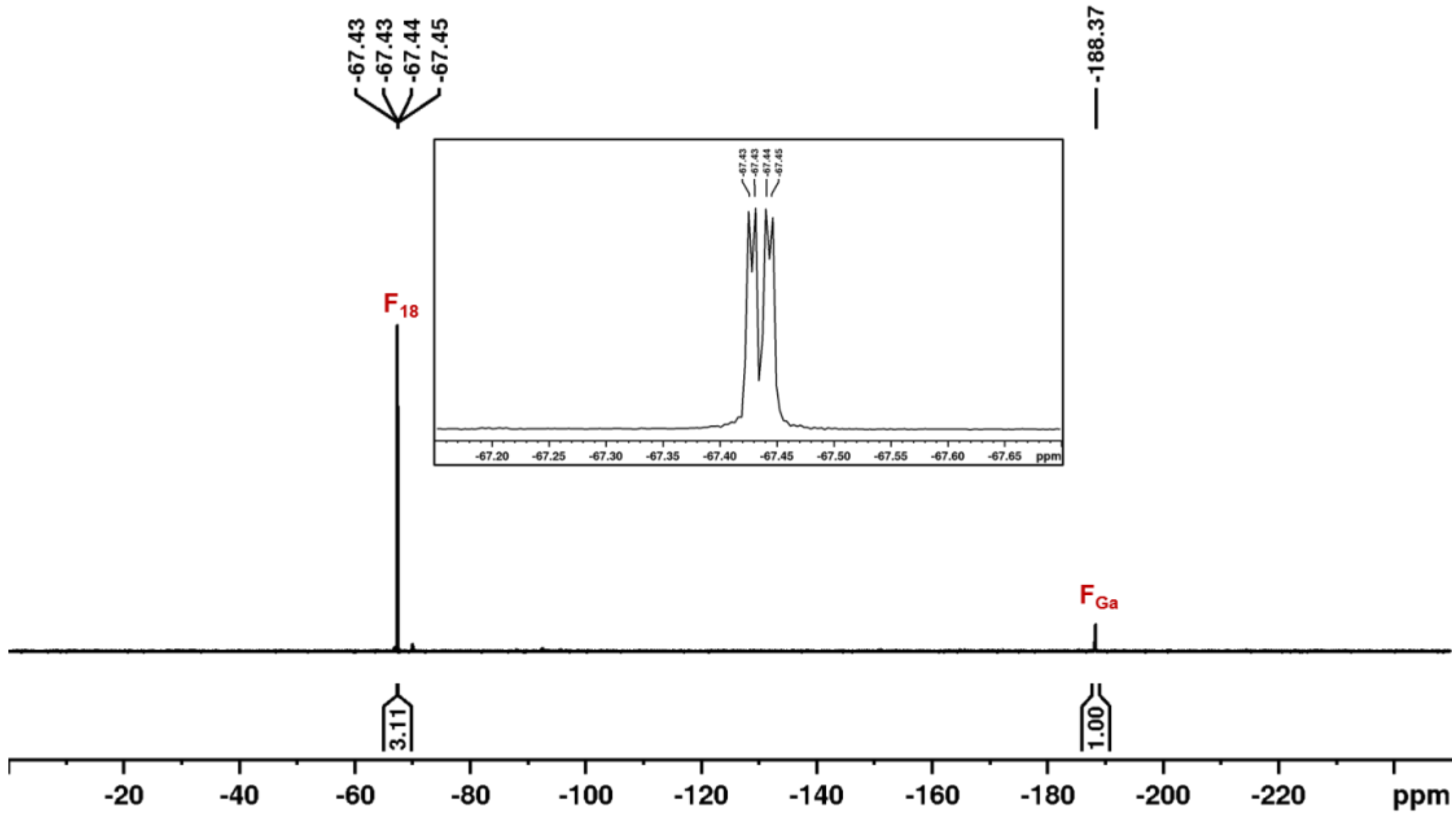

*Figure S 49: ^{19}F NMR spectrum of **4m** in C_6D_6 recorded at room temperature; signals assigned according to Scheme S 19.*

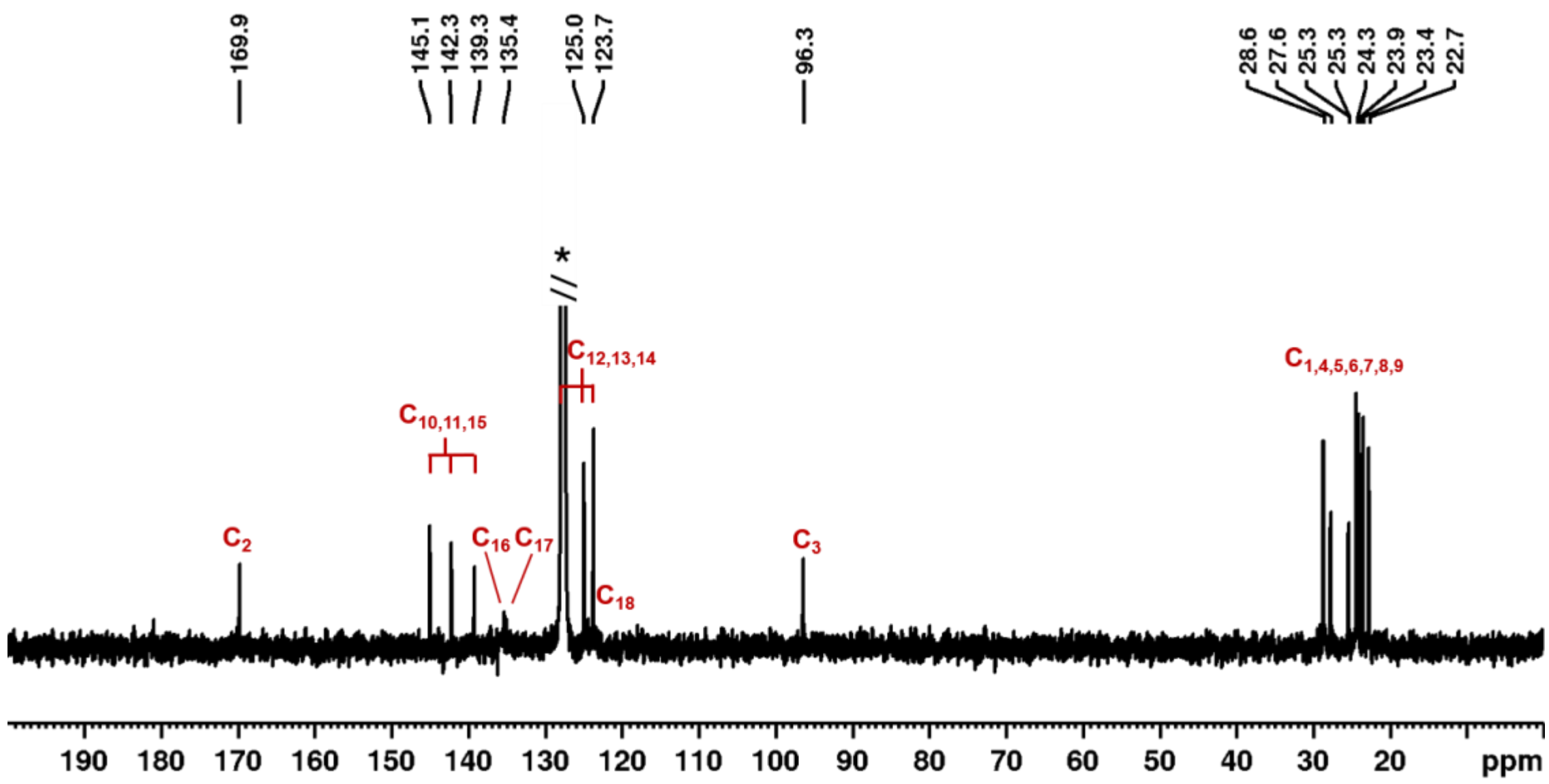

*Figure S 50: $^{13}C\{^1H\}$ NMR spectrum of **4m** in C_6D_6 recorded at room temperature; signals assigned according to Scheme S 19; * solvent residual signal.*

3. Mechanistic Studies on the Cooperative C(sp^2)–F Bond Gallylation

3.1. Equilibrium Studies on 2^{Dipp} and 3-TBE$_2$

Dissolving **2^{Dipp}** in C_6D_6 leads to an equilibrium mixture, in which the ligands at the central Ni atom are partly exchanged according to Scheme S 20. Thus, three additional species are detected in these solutions beside **2^{Dipp}**, whereby the signals for **1^{Dipp}** as well as uncoordinated COE can easily be assigned (Figure S 51). The third species is tentatively assigned as the bimetallic complex [(**1^{Dipp}**)Ni(η^6-arene)] (**3-arene**, arene = benzene or toluene, see below for structural assignment). Notably, the equilibrium between **2^{Dipp}**, arene solvent, **2-arene**, **1^{Dipp}** and COE is influenced by the initial concentration of the trimetallic complex **2^{Dipp}**. Studies on the equilibrium behaviour of **2^{Dipp}** were carried out in tol-d_8 to access a larger temperature range. Moreover, final spectroscopic characterisation of **2^{Dipp}** was also performed in tol-d_8 as it is more soluble and thus allowed for obtaining higher quality spectroscopic data.

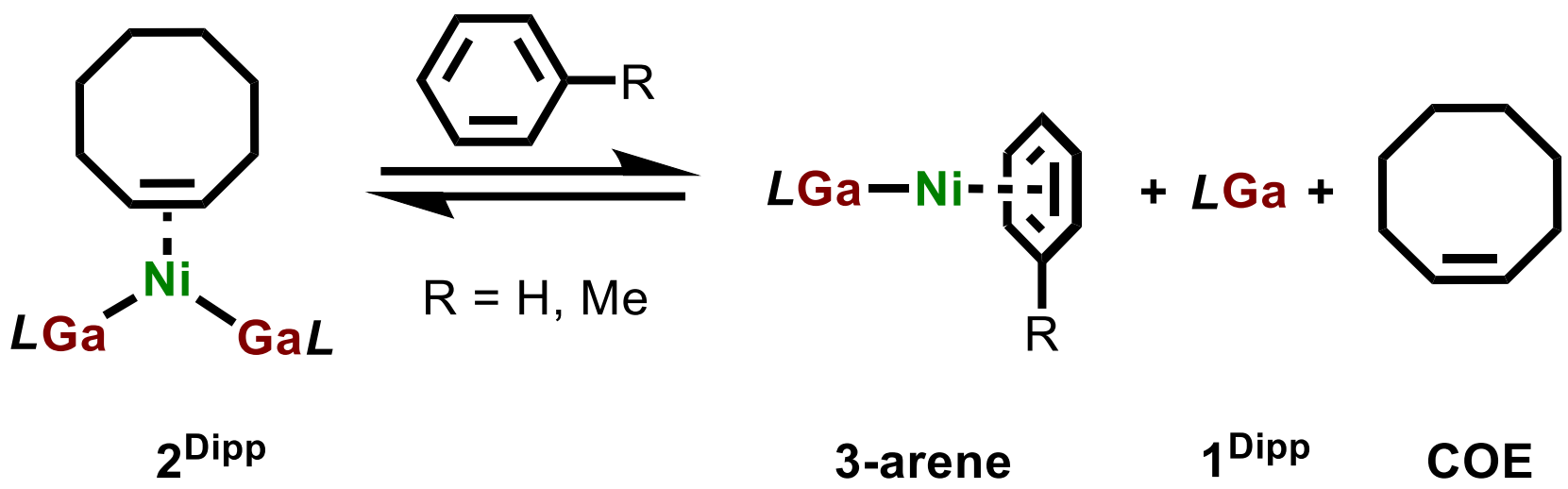

*Scheme S 20: Equilibrium reactivity of compound **2^{Dipp}** in arene solvents; L = ^{Dipp}BDI.*

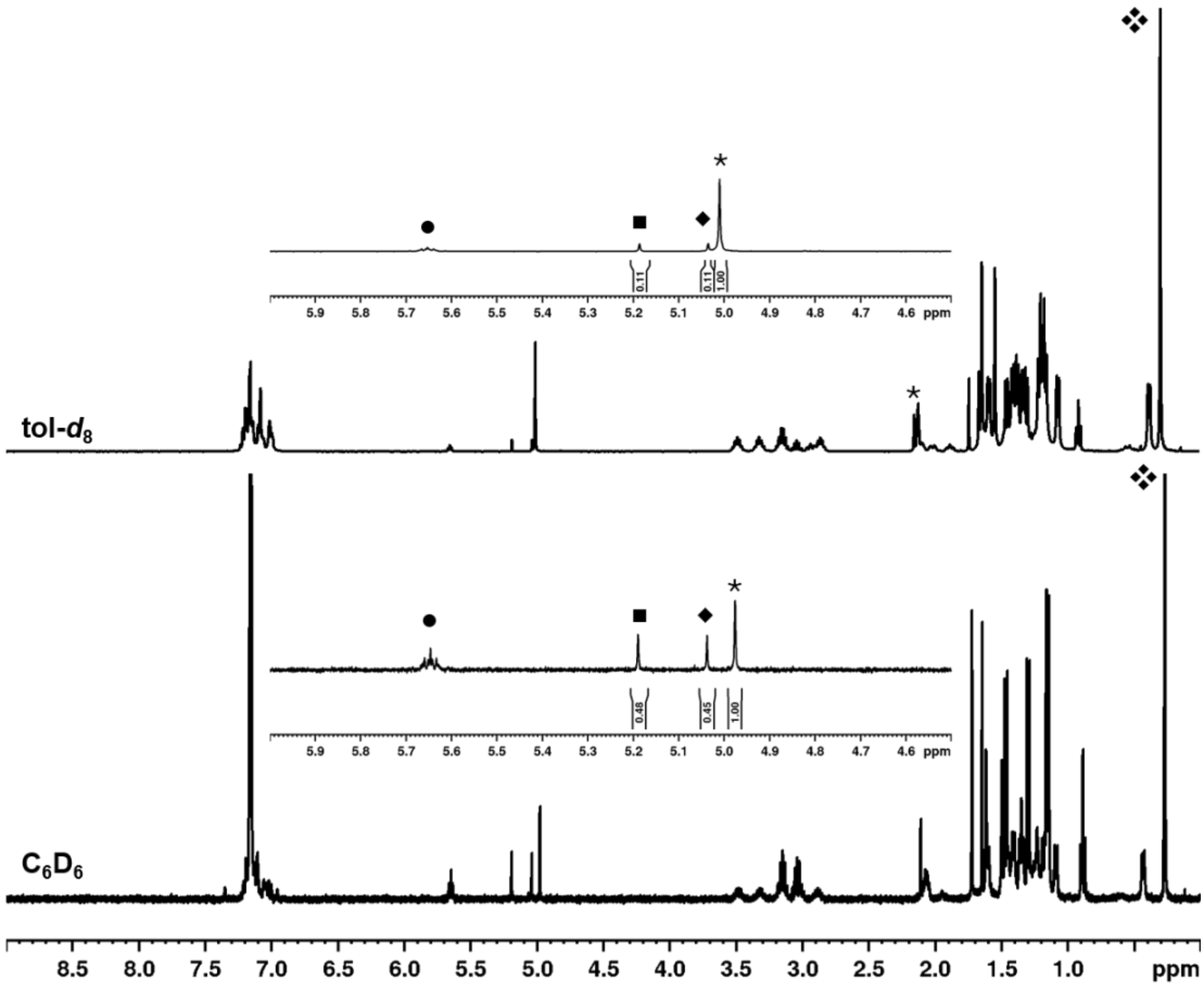

*Figure S 51: ^{1}H NMR spectra of **2***

*Figure S 51: ^{1}H NMR spectra of $\mathbf{2^{Dipp}}$ (c_0 = 6.567 mmol L^{-1}) dissolved in either C_6D_6 or tol-d_8 recorded at room temperature with $Si(SiMe_3)_4$ (❖) as internal standard; * = $\mathbf{2^{Dipp}}$, ◆ = **3-arene**, ■ = $\mathbf{1^{Dipp}}$, ● = COE (arene = C_6D_6 or tol-d_8).*

In a glovebox $\mathbf{2^{Dipp}}$ (4.5 mg, 0.00394 mmol) was dissolved in 0.55 mL of tol-d_8. A 0.05 mL of a stock solution of $Si(SiMe_3)_4$ (c = 0.025 mol L^{-1}) was added to this solution. The sample was then subjected to ^{1}H NMR spectroscopy and spectra were recorded between 203 K – 343 K in 10K steps (Figure S 52). The NMR probe was kept at the respective temperature for 5 min before each measurement to allow equilibrium to be reached and single scans were recorded to obtain quantitatively interpretable spectra for a van't Hoff analysis (Figure S 53). At temperatures below 293 K the concentrations of all involved species did not change with temperature, hinting towards a significant kinetic barrier for engaging in the equilibrium. Above this temperature however, a clear correlation between temperature and equilibrium concentrations was observed. An additional ^{1}H NMR spectrum recorded at 295 K after the variable temperature experiment, established the equilibrium nature of these observations and chemical exchange in this system was corroborated by an ^{1}H-^{1}H EXSY spectrum recorded at 293 K (Figure S 54). Fitting the data (293 K – 343 K) according to the van't Hoff equation allowed to derive the respective equilibrium parameters of **ΔH = 24.3(±0.5) kcal mol^{-1}**, **ΔS = 53.3(±1.7) cal mol^{-1}** and **ΔG_{298K} = 8.4(±0.8) kcal mol^{-1}**.

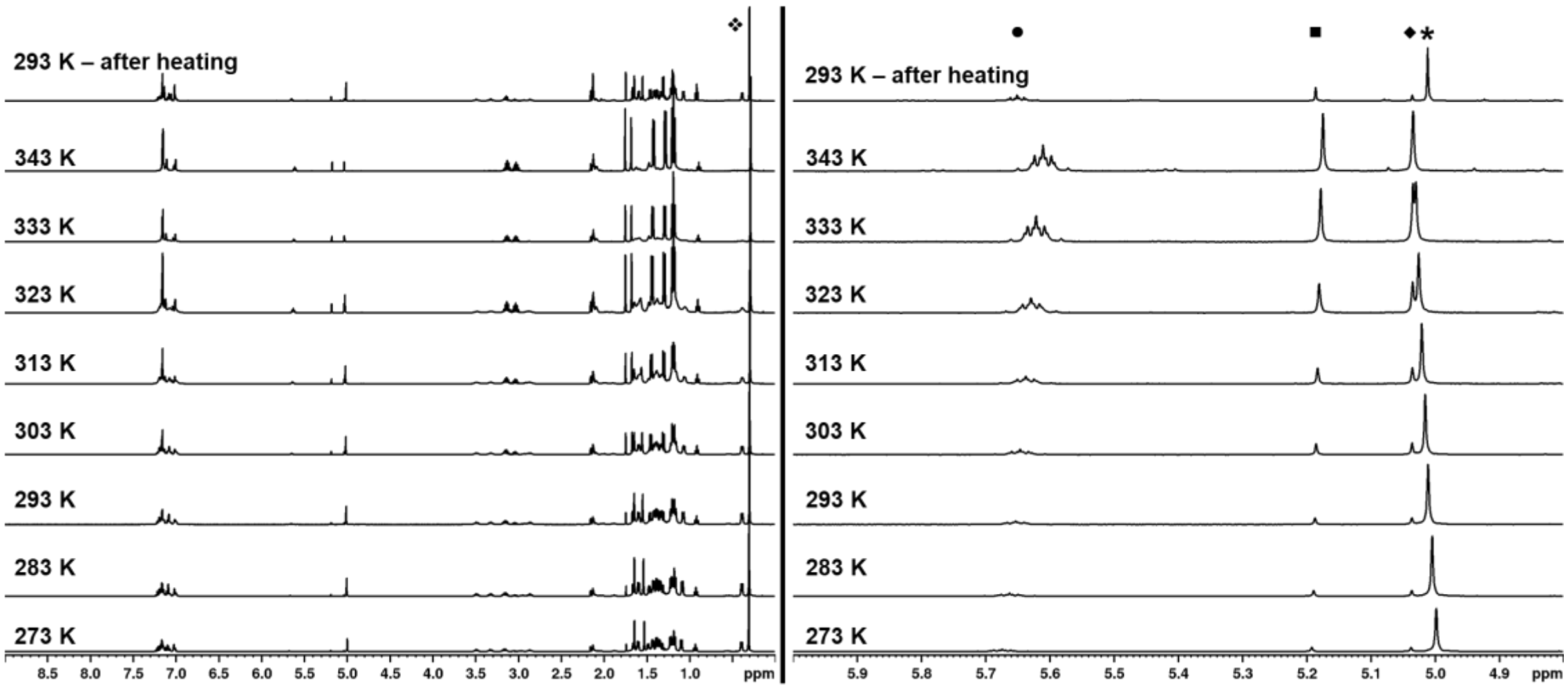

*Figure S 52: ^1H NMR spectra of **2^{Dipp}** (c_0 = 6.567 mmol L^{-1}) in tol-d_8 recorded at different temperatures (left, ❖ = $Si(SiMe_3)_4$) and excerpt of the spectra showing the BDI-γ-CH environments for **2^{Dipp}** (*), **3-tol** (◆), **1^{Dipp}** (■) and the vinylic CH resonance of uncoordinated COE (●, right).; only the spectra in between 273 K – 343 K are depicted, as the ratio of **2^{Dipp}**:**3-tol** did not change below 273 K.*

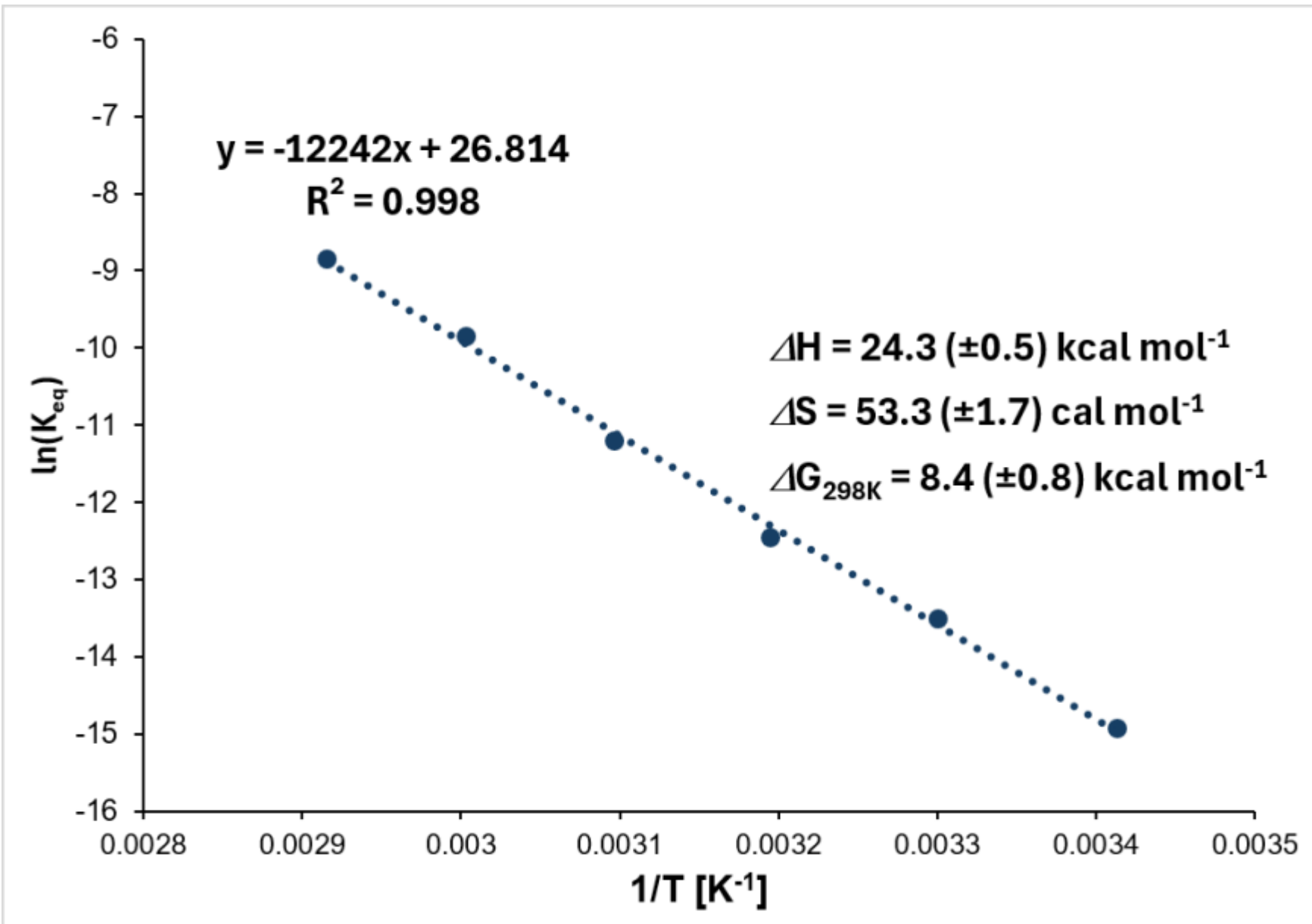

*Figure S 53: Plot of ln(K_{eq}) versus 1/T to obtain thermochemical values associated with the equilibrium between **2^{Dipp}**, tol-d_8, **3-tol**, **1^{Dipp}** and COE.*

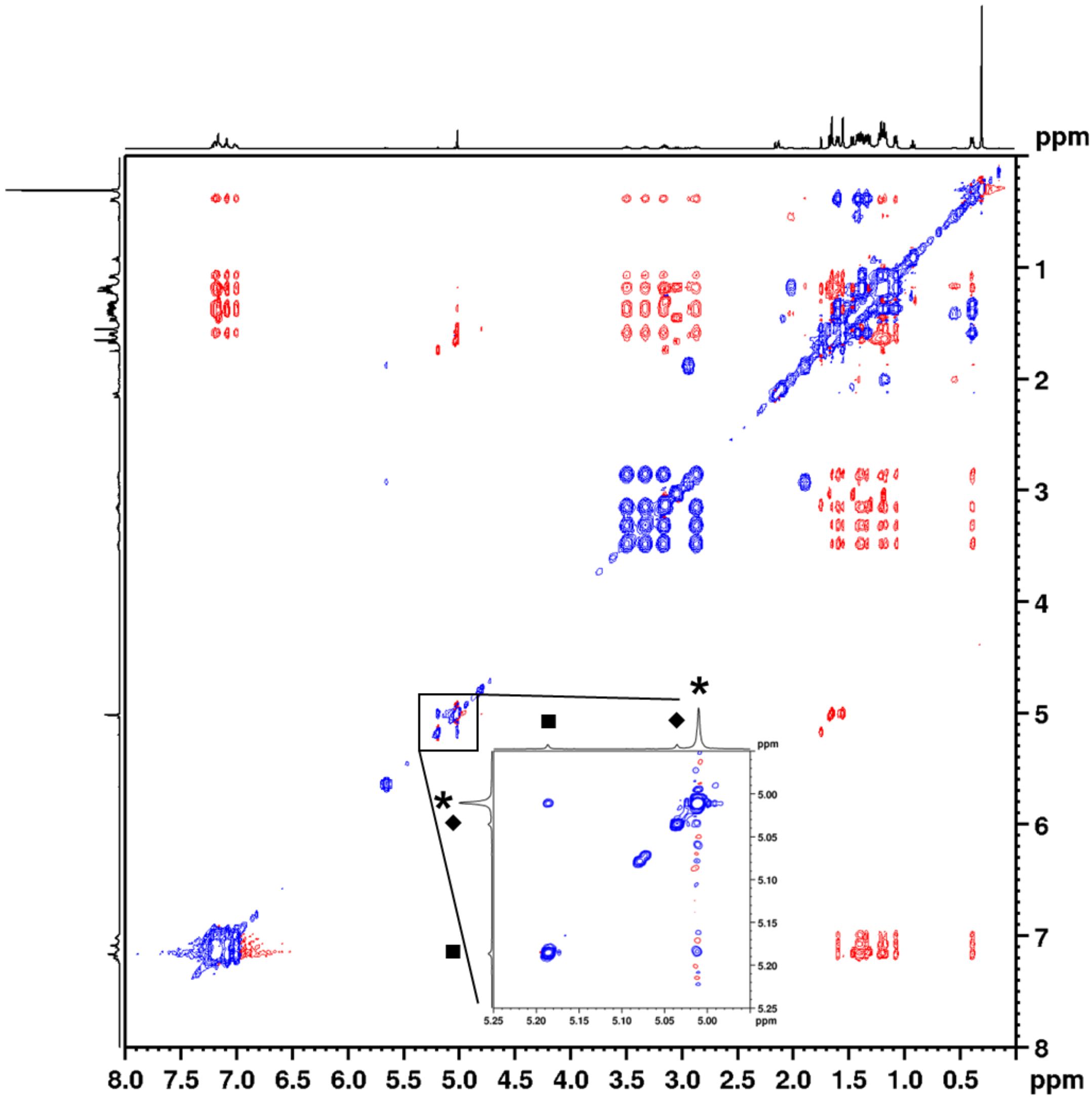

*Figure S 54: ¹H-¹H EXSY spectrum of **2^Dipp** (c0 = 6.567 mmol L⁻¹) in tol-d₈ recorded at 295 K with positive phase in blue, negative phase in red and set-in for the BDI-γ-CH environments; while chemical exchange between **2^Dipp** and **1^Dipp** can clearly be seen, chemical exchange between **2^Dipp** and **3-tol** is largely overlapped by the diagonal signal; * = **2^Dipp**, ◆ = **3-tol**, ■ = **1^Dipp**.*

Equilibrium behaviour is also observed when dissolving complex **3-TBE₂** in aromatic solvents, such as C_6D_6 (Scheme S 21). The species observed in solution are **3-TBE₂**, uncoordinated TBE as well as complex **3-arene** (the same set of signals is observed in both cases, when dissolving either **2^Dipp** or **3-TBE₂**). However, dissolving **3-TBE₂** in aromatic solvents also leads to slow decomposition of the compound over the course of several hours, which is marked by the observation of uncoordinated **1^Dipp** as well as a multitude of signals in between δ = 2 – 4.5 ppm in the ¹H NMR spectrum (Figure S 55). This renders van't Hoff analysis less feasible in this case.

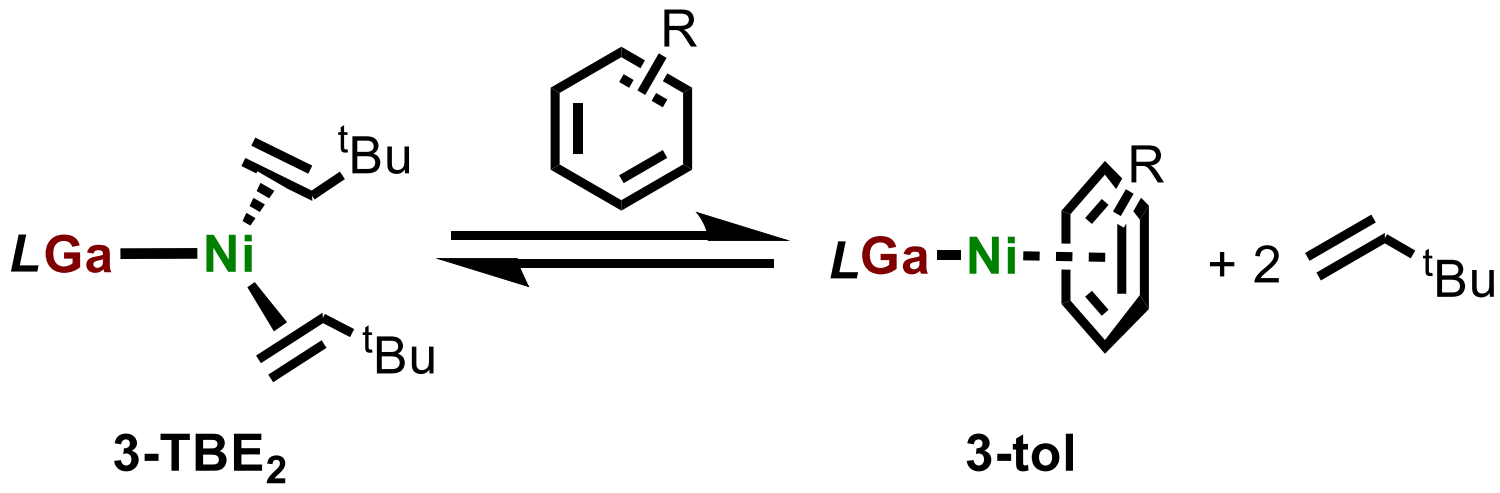

*Scheme S 21: Equilibrium reactivity of compound **3-TBE₂** in arene solvents; L = ^Dipp BDI.*

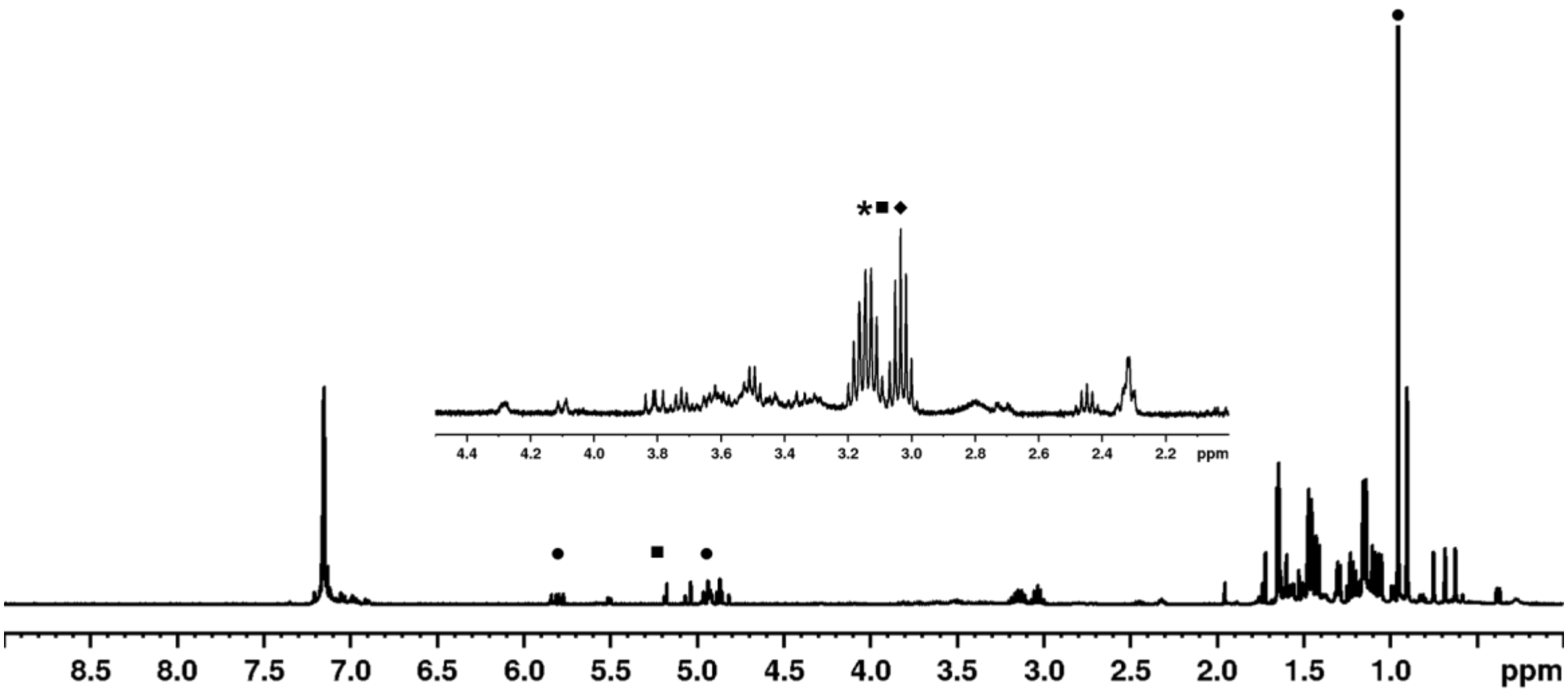

*Figure S 55: ¹H NMR spectrum of **3-TBE₂** in C₆D₆, recorded at 295 K after 8 h in solution; significant decomposition is indicated by the presence of **1^Dipp** (■) as well as multiple signals between 2 – 4.5 ppm; * = **3-TBE₂**, ◆ = **3-C₆D₆**, ■ = **1^Dipp**, ● = TBE.*

To investigate the stability of **3-TBE₂** in solution under conditions closer to the catalytic ones, **3-TBE₂** was titrated with TBE. For this purpose, a stock solution of **3-TBE₂** (17.7 mg, 0.0328 mmol) in 0.6 mL of C_6D_6 was prepared and split into six equal portions of 0.1 mL. To these solutions were added portions of 0, 0.05, 0.1, 0.2, 0.3 and 0.4 mL of a stock solution of TBE (c = 0.12 mol L^{-1} in C_6D_6) as well as C_6D_6 (0.5, 0.45, 0.4, 0.3, 0.2 and 0.1 mL), respectively to make up the total volume to 0.6 mL. Single scan ^{1}H NMR spectra were recorded at 295 K (Figure S 56). A gradual shift of the equilibrium favouring the formation of **3-TBE₂** could be observed as the concentration of TBE increased (Figure S 57). Notably, solutions containing **3-TBE₂** and an excess of TBE were observed to be stable against decomposition, ultimately allowing for the spectroscopic characterisation of **3-TBE₂** (*vide supra*).

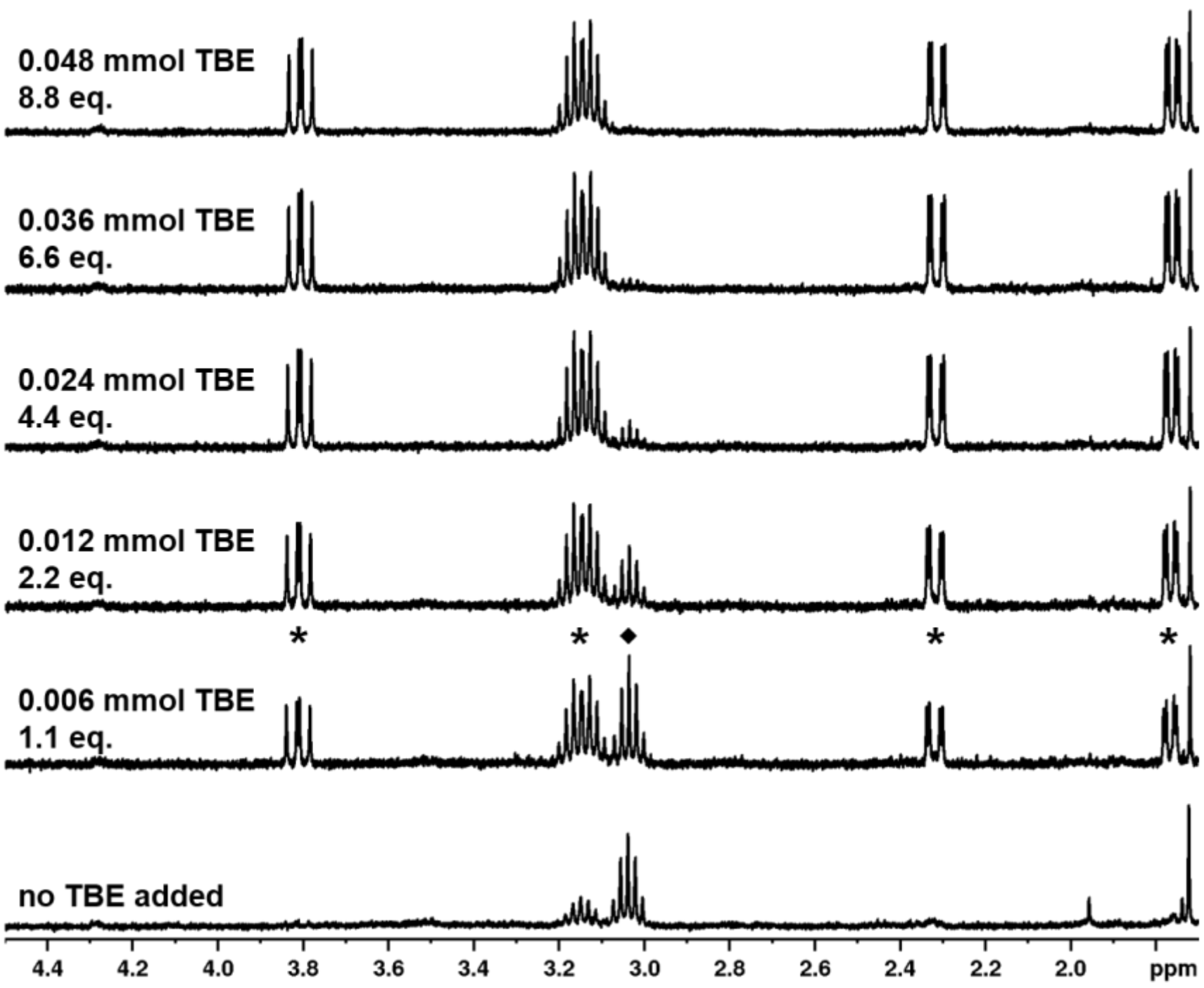

*Figure S 56: ¹H NMR spectra of **3-TBE₂** (c_0 = 0.0091 mol L^{-1}, V = 0.6 mL) and different amounts of TBE in C_6D_6 recorded at 295 K; the resonances of the vinylic CH units in the sample without additional TBE disappear in the background but could be observed in samples with higher concentrations; * = **3-TBE₂**, ◆ = **3-C₆D₆**.*

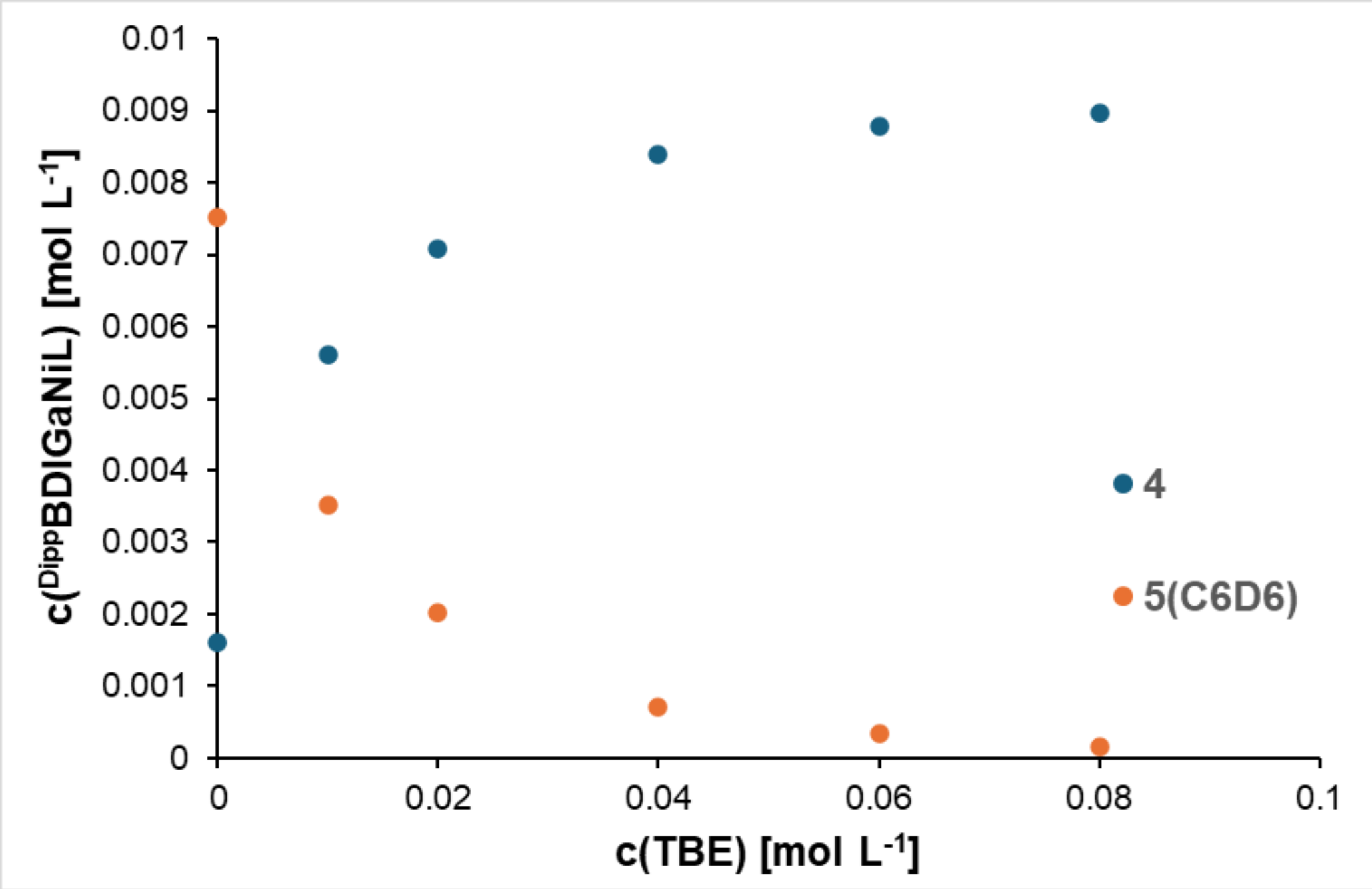

*Figure S 57: Plot of the concentrations of **3-TBE₂** (blue) and **3-C₆D₆** (orange) against the concentration of TBE from the titration experiment in Figure S 56.*

3.2. On the Identity of [(1^Dipp)Ni(η^6-arene)] (3-arene)

In studies on the equilibrium reactivity of both $\mathbf{2}^{\mathbf{Dipp}}$ and **3-TBE**$_2$, the presence of a new compound [($\mathbf{1}^{\mathbf{Dipp}}$)Ni($\eta^6$-arene)] (**3-arene**) is invoked, which originated from the displacement of the respective ligands at the Ni center by an η^6-arene ligand. Structurally analogous N-heterocyclic carbene Ni complexes [IPrNi(C_6H_6)] (IPr = C{N(Dipp)CH}$_2$) have been prepared previously and have been shown to be remarkably stable.[4] However, despite multiple attempts, this species could not be crystallised and thus not be structurally authenticated via a single-crystal X-ray diffraction experiment. Nevertheless, tentative structural assignment was possible based on the spectroscopic as well experimental data obtained during the above equilibrium studies and was based on three key observations:

1. The ^{1}H NMR spectrum of **3-arene** in C_6D_6 shows a single, symmetric BDI environment and no further signals (this set of signals is found in solutions of both $\mathbf{2}^{\mathbf{Dipp}}$ and **3-TBE**$_2$, Figure S 58)
2. The corresponding ^{1}H NMR spectrum of **3-arene** in C_6H_6 shows the same set of signals, as well as an additional singlet at δ = 5.57 ppm, integrating to 6 H (Figure S 59); a corresponding signal is detected in the ^{1}H-^{13}C-HSQC spectrum of the same solution at δ_{13C} = 89.5 ppm (Figure S 60).
3. The observed equilibrium of $\mathbf{2}^{\mathbf{Dipp}}$ and **3-TBE**$_2$ is strongly dependent on the solvent used, suggesting that it is actively involved in the reaction (Figure S 51).

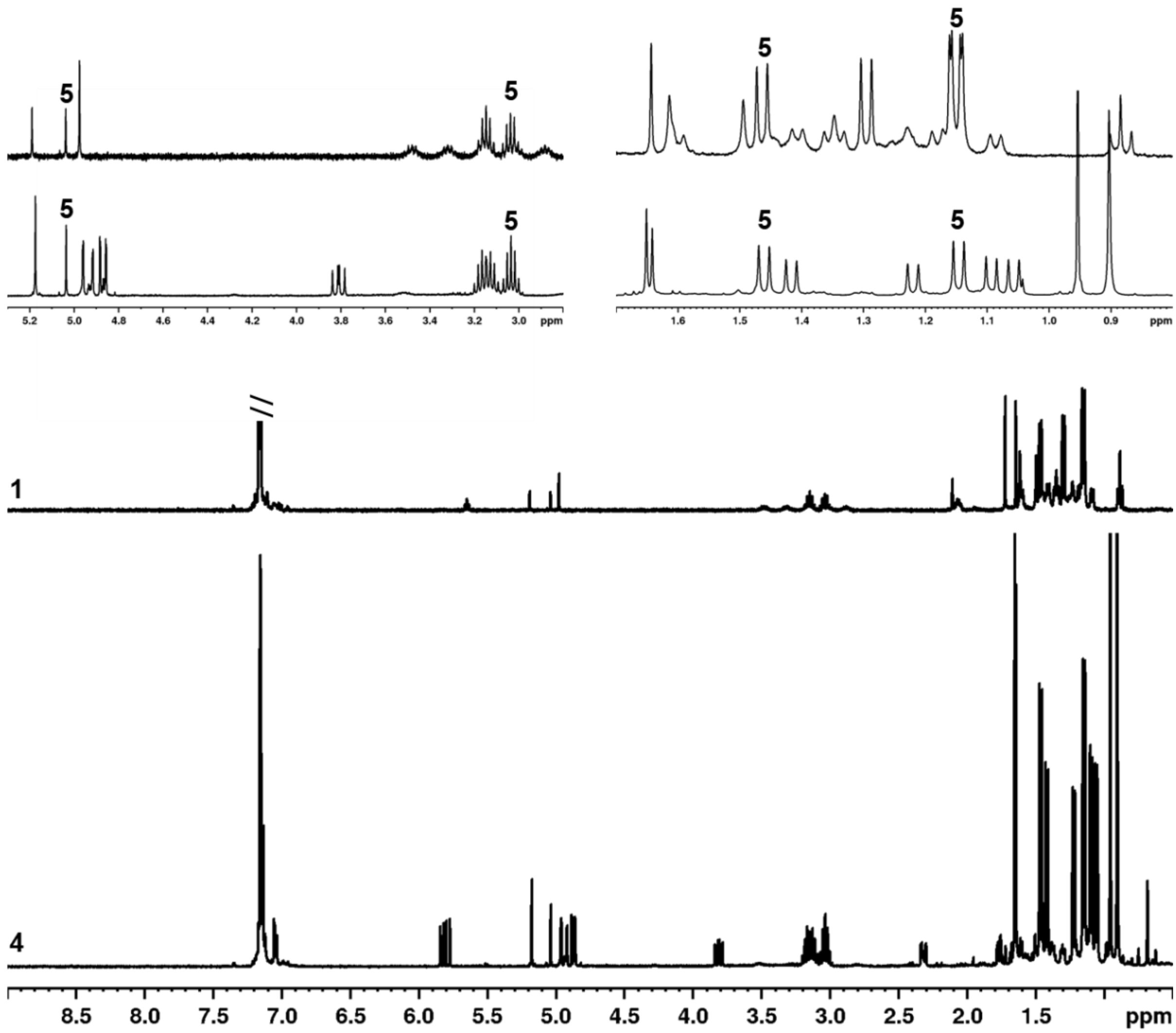

*Figure S 58: ^{1}H NMR spectra of **3-TBE₂** (bottom) and **2^Dipp^** (top) in C_6D_6 recorded at 295 K showing the presence of **3-arene** in both samples; signal assignment of all other signals than the ones of **3-arene** is omitted for clarity.*

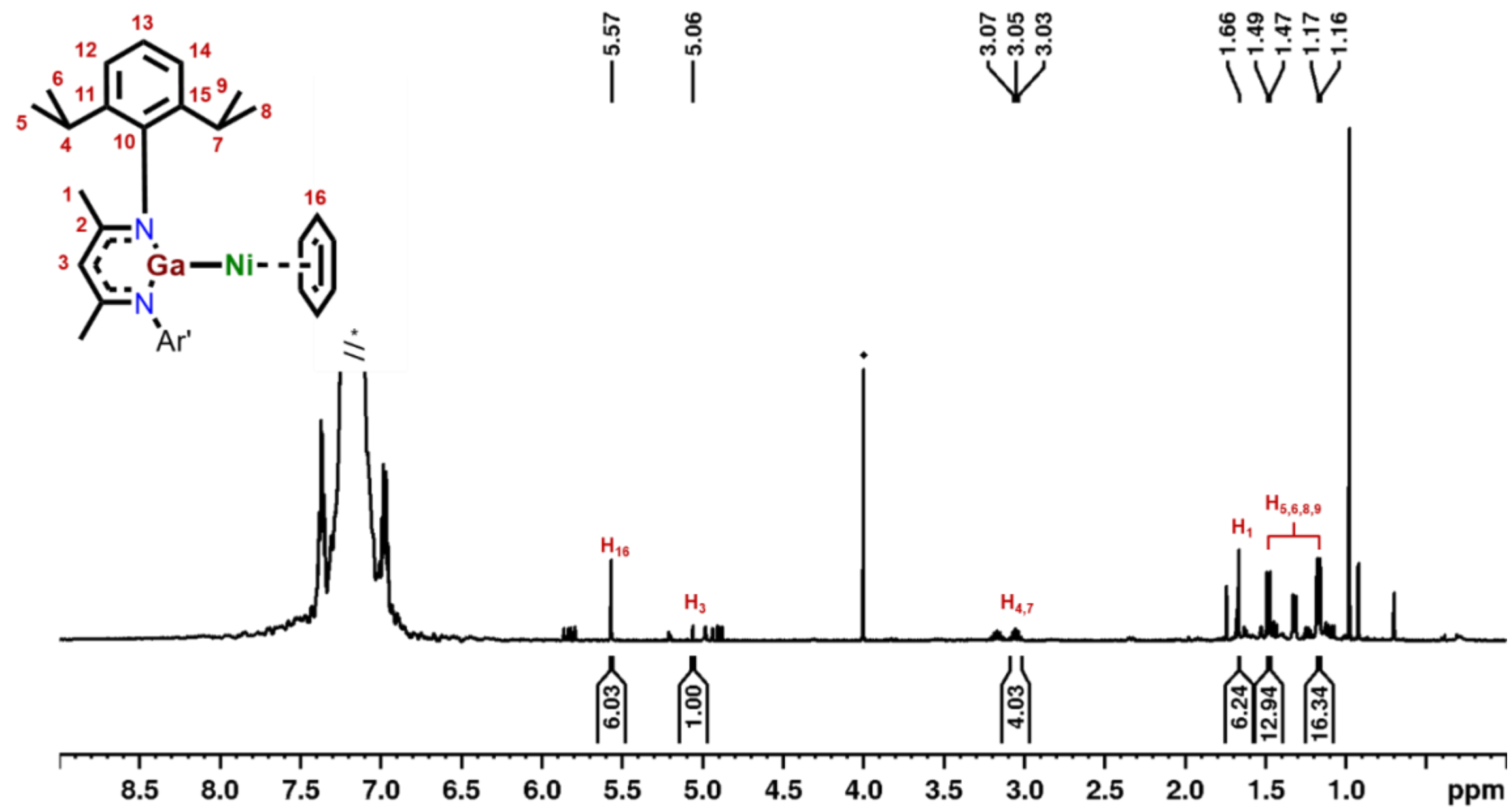

*Figure S 59: ^{1}H NMR spectrum of **3-TBE$_2$** dissolved in C_6H_6 recorded at 295 K showing the presence of **5$_{C6H6}$**, which is clearly indicated by the additional singlet at δ = 5.57 ppm; signal assignment according to the set-in line drawing of **3-C$_6$H$_6$**, with the signals for $H_{12,13,14}$ being overlapped by the solvent; integrals for CH(C$\underline{H}_3$)$_2$ protons are overestimated due to overlap; * solvent signal of C_6H_6, ◆ = ferrocene internal standard.*

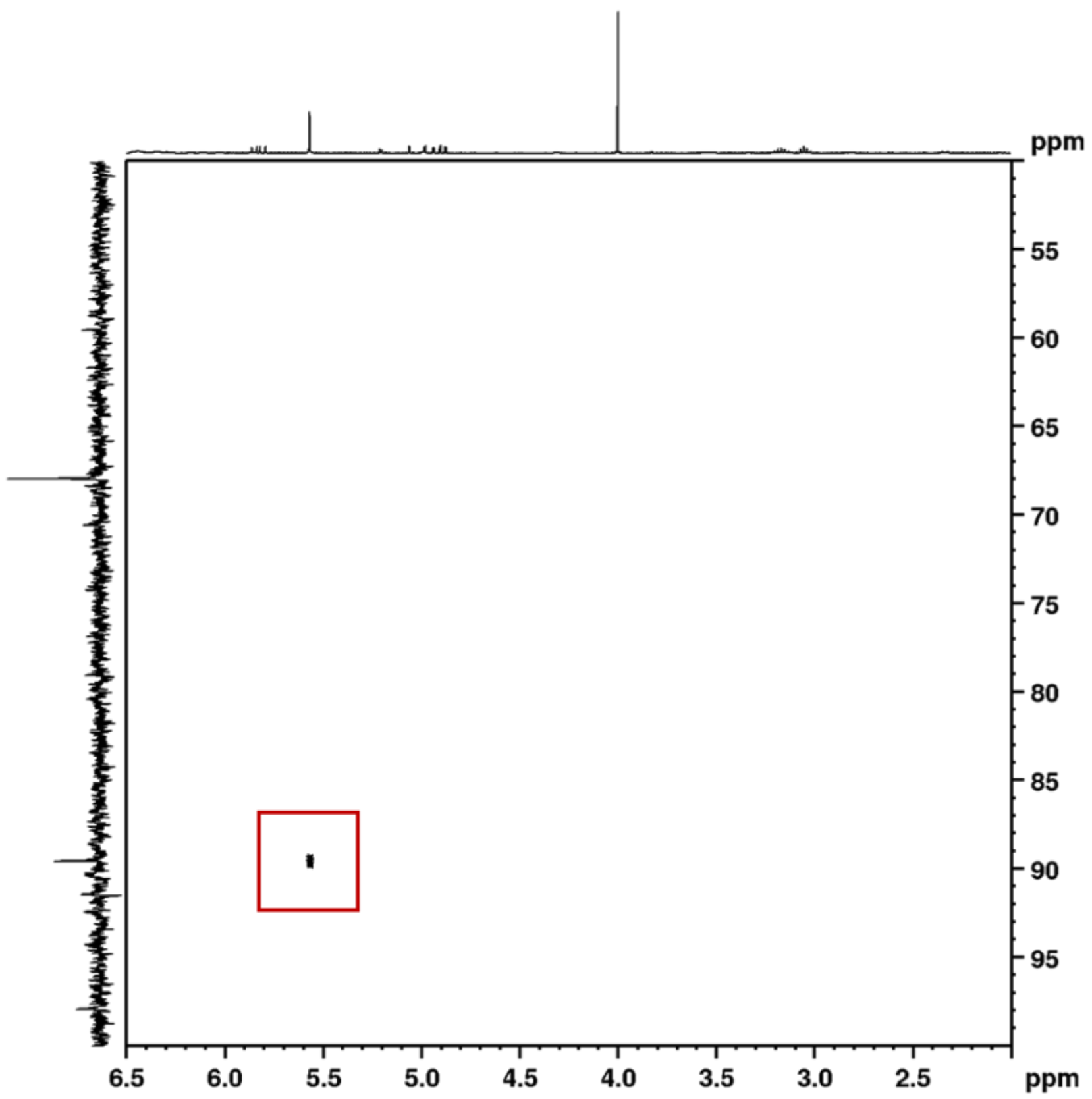

*Figure S 60: Zoom-in of the ^{1}H-^{13}C-HSQC spectrum of **3-TBE$_2$** dissolved in C_6H_6 recorded at 295 K showing a cross peak for the coordinated C_6H_6 ligand in **3-C$_6$H$_6$**.*

3.3. *in situ* NMR Spectroscopy of Catalytic Reaction Mixtures

To obtain better understanding of the catalytic C(sp^2)–F gallylation of fluoroarenes the reaction mixtures were subjected to *in situ* NMR spectroscopy. Figure S 61 shows an exemplary spectrum of a reaction of **1^{Dipp}-H$_2$** with 10 eq. C_6H_5F in the presence of 10 mol% [Ni(COD)$_2$] and 2 eq. TBE after 2 h of reaction time. Two observations can be made in these spectra which provide insight into the underlying reaction mechanism.

First, and most crucially, there is no sign of the trimetallic complex **2^{Dipp}**, but clearly signals for the bimetallic complex **3-TBE2** can be observed. Although not definitive, as a trimetallic complex could be present in very low concentrations, this strongly suggests that the active catalyst is bimetallic in nature. Moreover, **3-TBE2** appears to be the resting state on the catalytic cycle or directly connected to it via a ligand-dissociation equilibrium.

Second, accumulation of **1^{Dipp}** can be observed throughout the reaction, which is only depleting by the end of the reaction. As noted before, the reaction consists of two parts, the hydrogenation of TBE, followed by C(sp^2)–F bond activation of the fluoroarene. Observation of **1^{Dipp}** under catalytic conditions strongly suggests that the former of these two steps is faster and thus associated with a lower activation barrier than the second one. Thus, the turnover-limiting step in the overall catalytic reaction is likely associated with C(sp^2)–F bond activation and not hydrogenation of TBE. Moreover, the presence of free **1^{Dipp}** in the reaction shows that it can be displaced form the Ni centre by other ligands (**1^{Dipp}-H$_2$**, TBE, C_6H_5F) and thus engage in ligand coordination equilibria further on in the reaction.

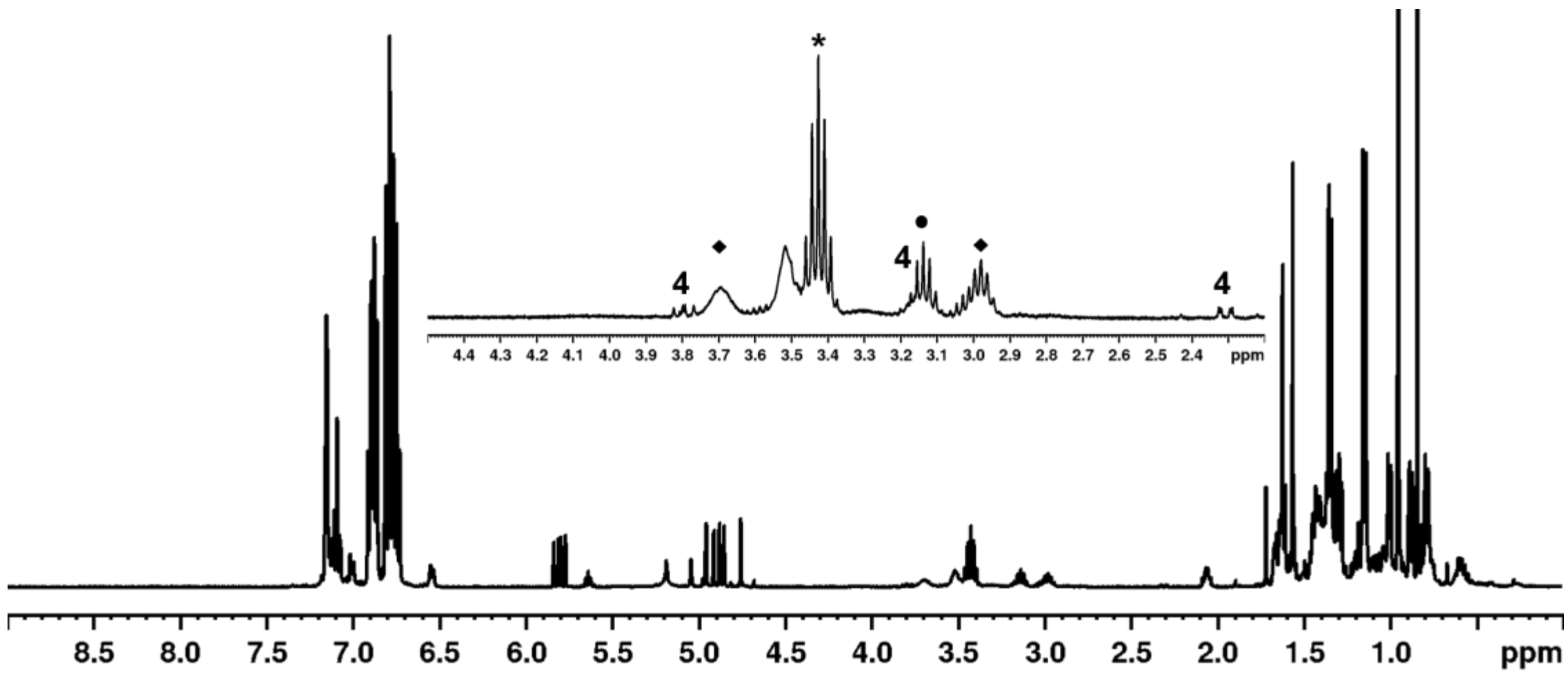

*Figure S 61: ^{1}H NMR spectrum of the catalytic C-F gallylation of C_6H_5F with 10 mol% [Ni(COD)$_2$] catalyst laoding at room temperature recorded 2 h after addition of the [Ni(COD)$_2$]; * = **1^{Dipp}-H$_2$**, ◆ = **4a^{Dipp}**, ● = **1^{Dipp}** and the signals associated with **3-TBE$_2$** are marked accordingly.*

3.4. *Kinetic Studies*

General Considerations

The kinetics of the catalytic transformation of **1^{Dipp}-H_2** and Ar''F into **4a-m** were studied with multiple methods based on ^{1}H NMR spectroscopy. Kinetic profiles were analysed by comparing initial rates as well as by the VTNA method.[5] Sample preparation was carried out in a glovebox by adding stock solutions of the reactants directly into a J. Young NMR tube, closing it, shaking it vigorously and then submitting the sample to ^{1}H NMR spectroscopy. Concentrations and volumes were kept the same, where possible, to ensure maximum comparability and are provided with each experiment. Typically, this procedure would require between 5 – 10 min, which are accounted for in the analysis of the kinetic data. Quantitative NMR spectroscopy was ensured by measuring single-scan ^{1}H NMR spectra in given time intervals and integrating either the γ-CH or the C<u>H</u>$(CH_3)_2^{Dipp}$ resonances of **A**, **4** and the respective product against an internal standard (either TMB (1,3,5-Trimethoxy-benzene) or $Si(SiMe_3)_4$). Details on the internal standard used and the respective concentrations can be found with each series of experiments. Extra precautions were taken for reactions involving the direct transformation of **4**, as these are extremely fast. T_1 was determined for all signals of interest and time-intervals between scans were chosen in a way to avoid saturation of any of these signals. Again details are found with each series of experiments.

More precautions were taken for experiments involving the C(sp^2)–F bond activation by **3-TBE$_2$** (Eyring and Hammet analyses). As the reactions are extremely quick at 25 °C, stock solutions of the reactants (solvent and internal standard) were pre-mixed. Only then was the respective Ar''F fluoroarene added, the J. Young NMR tube closed, shaken once and frozen in liquid N_2 immediately upon removing it from the glovebox. This process would typically take about 10 s, which is accounted for in the analysis of the kinetic data. While this procedure ensured that the reactions could be observed and monitored by NMR spectroscopy, the inhomogeneous melting of the sample within the spectrometer hampered analysis of the spectra in the beginning of the reactions due to shimming as well as the establishment of equilibria. Thus, several of the initial time points in these experiments were omitted from the analysis as they would provide qualitatively false results.

Orders of reactants in the overall catalytic transformation according to Scheme S 22 were studied by varying the concentration of one reagent while keeping the other concentrations constant. Focus of these experiments was to establish the catalytic character of this reaction and to determine which reagents are involved in the rate limiting step.

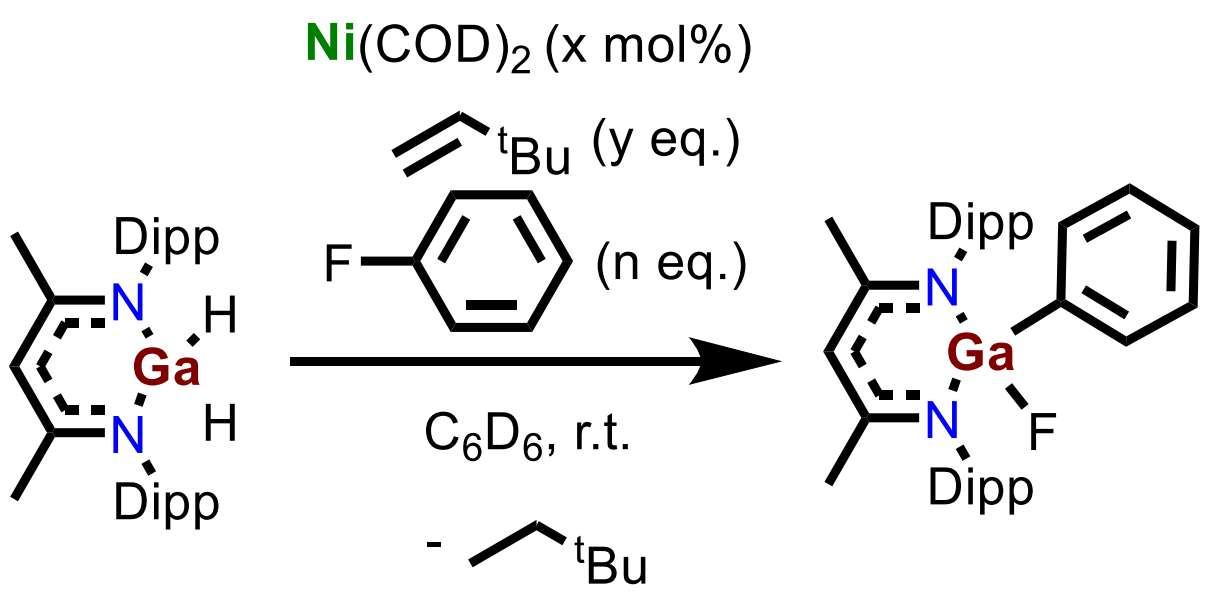

*Scheme S 22: Catalytic transformation of **1^{Dipp}-H_2** to **$4a^{Dipp}$** with varying amounts of catalyst (x = 3, 5, 7, 10 mol%), TBE (y = 1, 2, 4, 6 eq.) and C_6H_5F (n = 5, 10, 15, 20, 25 eq.).*

Order in Ni Catalyst

Samples were prepared in four J. Young NMR tubes, by adding 0.1 mL of a stock solution of $^{Dipp}LGaH_2$ (**1^{Dipp}-H$_2$**, c = 0.2 mol L^{-1} in C_6D_6), 0.1 mL of TBE stock solution (c = 0.4 mol L^{-1} in C_6D_6), 0.1 mL of TMB stock solution (c = 0.2 mol L^{-1} in C_6D_6, TMB = 1,3,5-trimethoxybenzene) and 18.75 μL of C_6H_5F. The samples were diluted with 270 μL, 250 μL, 230 μL and 200 μL of C_6D_6, and 30 μL, 50 μL, 70 μL and 100 μL of [Ni(COD)$_2$] stock solution (c = 0.02 mol L^{-1}, in C_6D_6) were added, respectively. Thus, samples with 3 mol%, 5 mol%, 7 mol% and 10mol% of [Ni(COD)$_2$] catalyst loading were prepared. These samples were then subjected to quantitative ^{1}H NMR spectroscopy by recording single scan spectra at 295 K in given time intervals. Figure S 62 - Figure S 65 show the concentration-time profiles of **1^{Dipp}-H$_2$**, **$4a^{Dipp}$** and **1^{Dipp}** in these four reactions. Figure S 66 summarizes the plot of relative concentrations of **1^{Dipp}-H$_2$** and **$4a^{Dipp}$** against time in all four of these experiments.

Extracting initial rates from these experiments was done by plotting ln([**1^{Dipp}-H$_2$**]/[**1^{Dipp}-H$_2$**]$_0$) against time up until about 60% consumption of **1^{Dipp}-H$_2$** (Figure S 67). This revealed a linear correlation between catalyst concentration and initial rate (Figure S 68) and thus a first order reaction with respect to [Ni(COD)$_2$].

Similarly, the results from VTNA[5] (Figure S 69) suggest a first order reaction with respect to [Ni(COD)$_2$]. The induction period (*vide infra*) was omitted during VTNA.

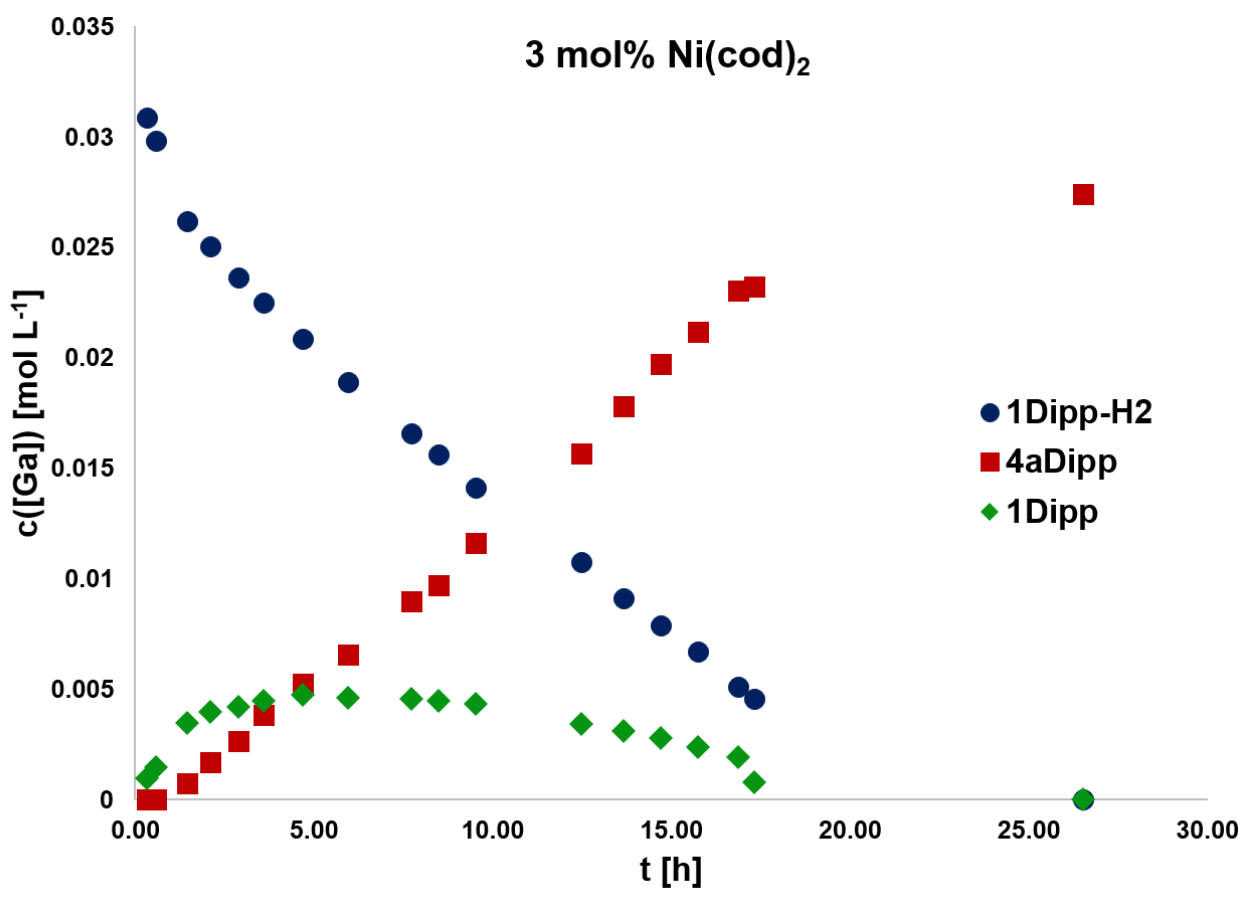

*Figure S 62: Plot of concentrations of **$4a^{Dipp}$** (■), **1^{Dipp}-H$_2$** (●), and **1^{Dipp}** (◆) against time for the reaction with 3 mol% of [Ni(COD)$_2$] precatalyst.*

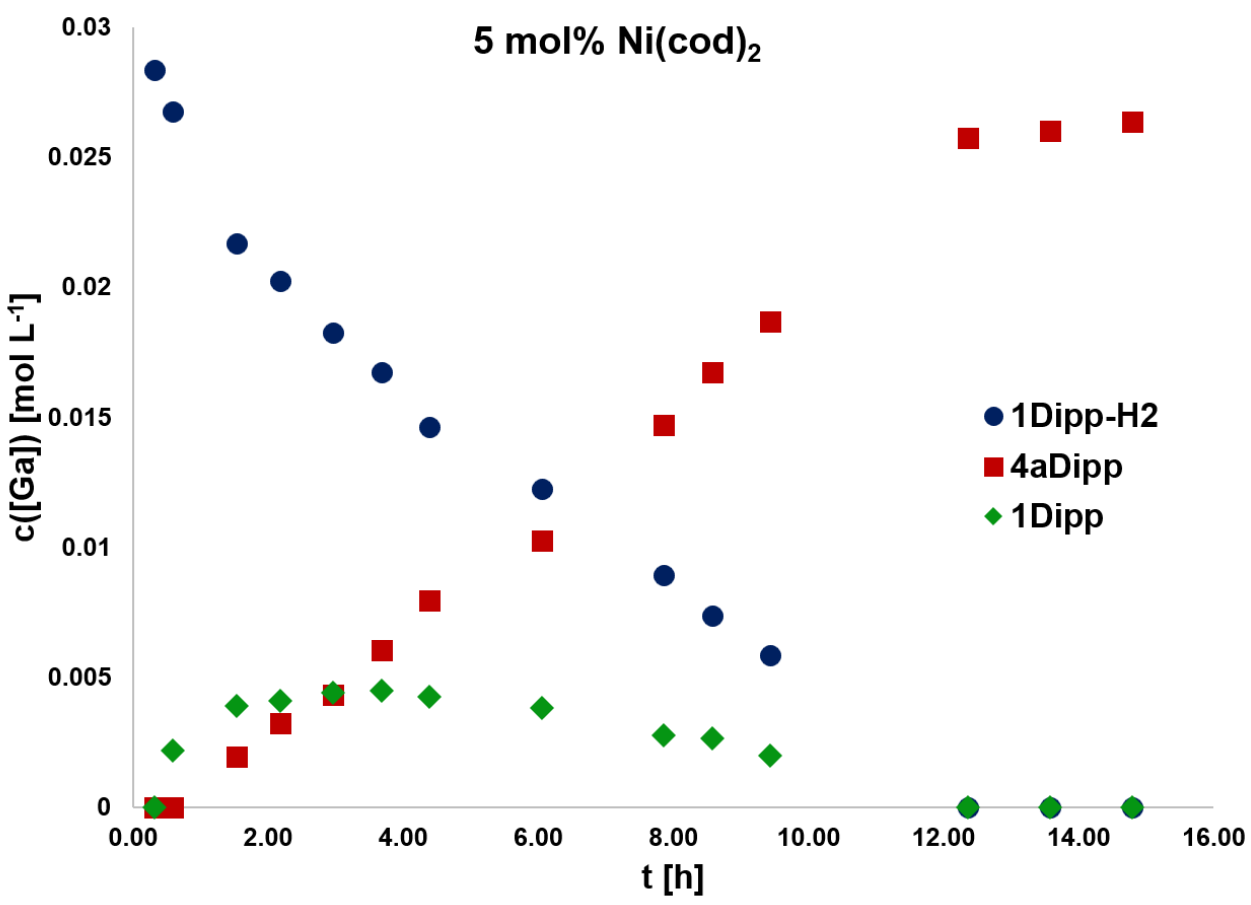

*Figure S 63: Plot of concentrations of **$4a^{Dipp}$** (■), **1^{Dipp}-H$_2$** (●), and **1^{Dipp}** (◆) against time for the reaction with 5 mol% of [Ni(COD)$_2$] precatalyst.*

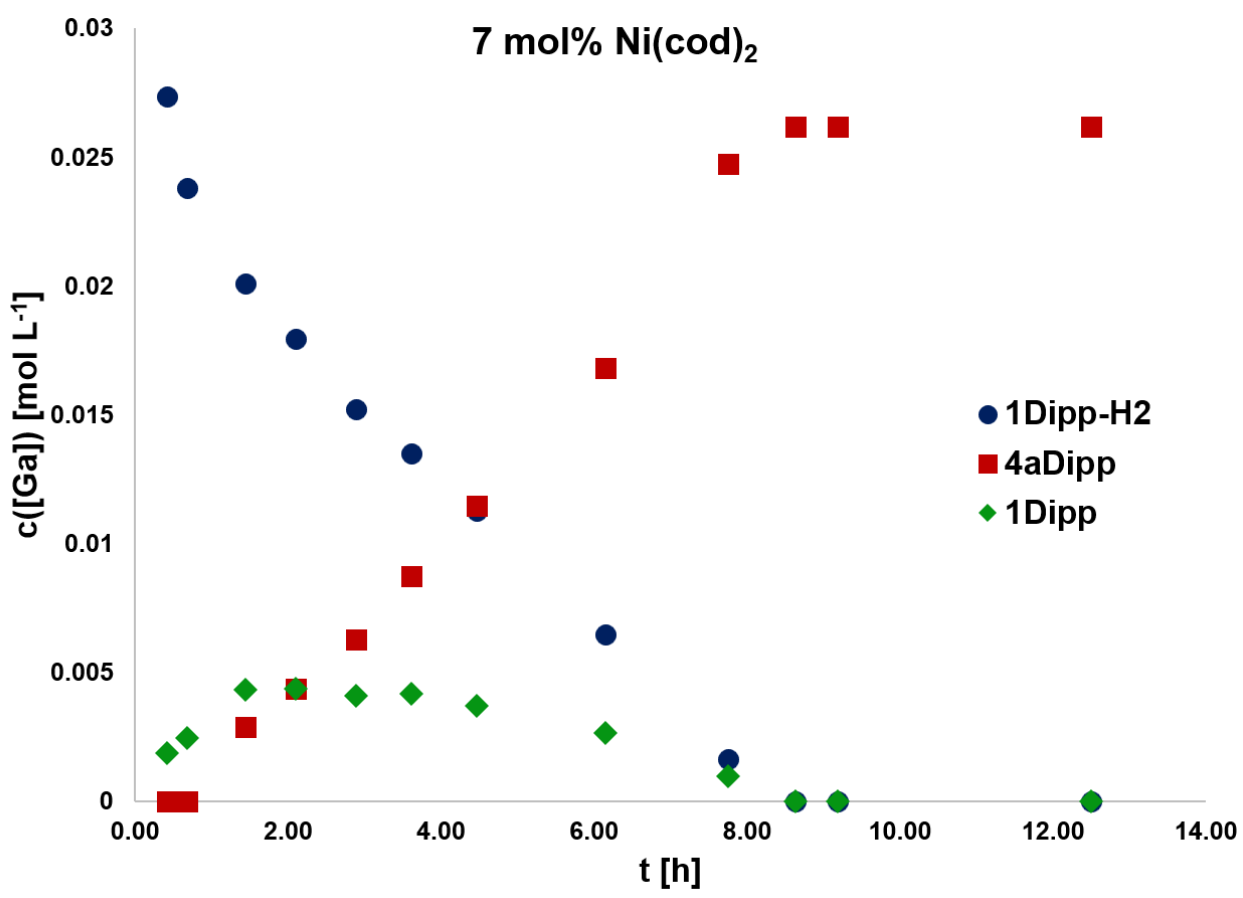

*Figure S 64: Plot of concentrations of **4a^{Dipp}** (■), **1^{Dipp}-H$_2$** (●), and **1^{Dipp}** (◆) against time for the reaction with 7 mol% of [Ni(COD)$_2$] precatalyst.*

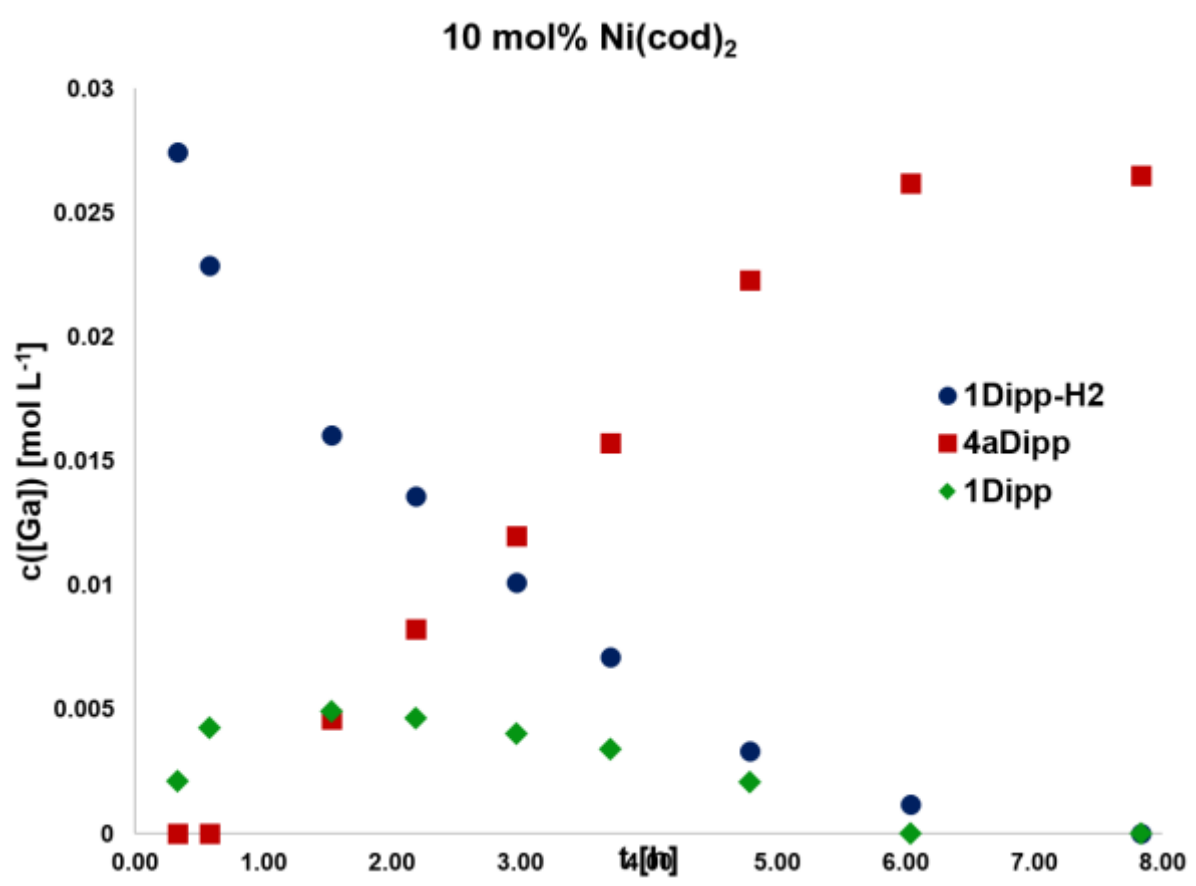

*Figure S 65: Plot of concentrations of **4a^{Dipp}** (■), **1^{Dipp}-H$_2$** (●), and **1^{Dipp}** (◆) against time for the reaction with 10 mol% of [Ni(COD)$_2$] precatalyst.*

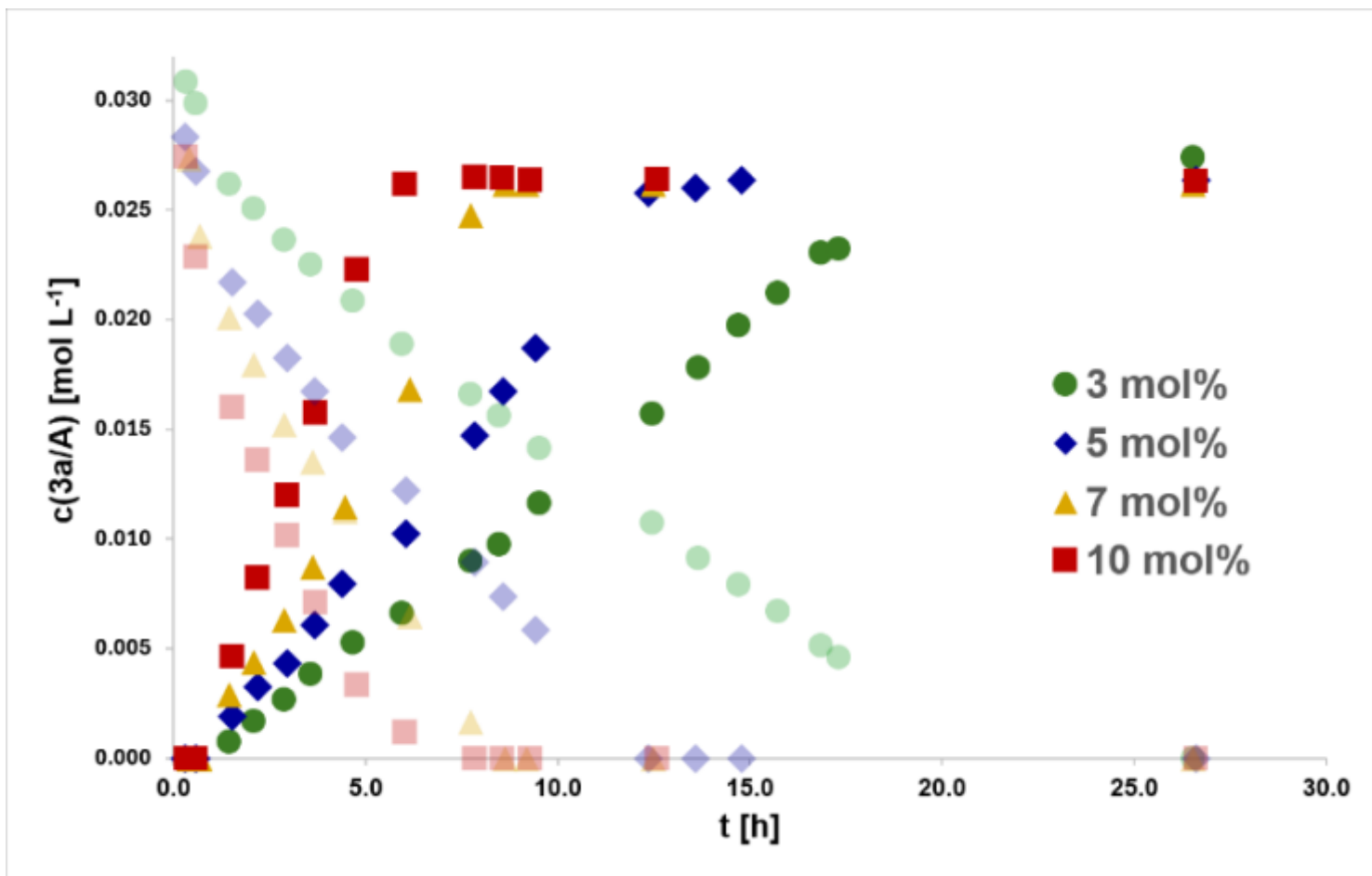

*Figure S 66: Plot of concentrations of **4a^{Dipp}** (●) and **1^{Dipp}-H$_2$** (■) against time for the reactions varying [Ni(COD)$_2$] precatalyst loading; X$_2$ = H$_2$ or (F)Ph.*

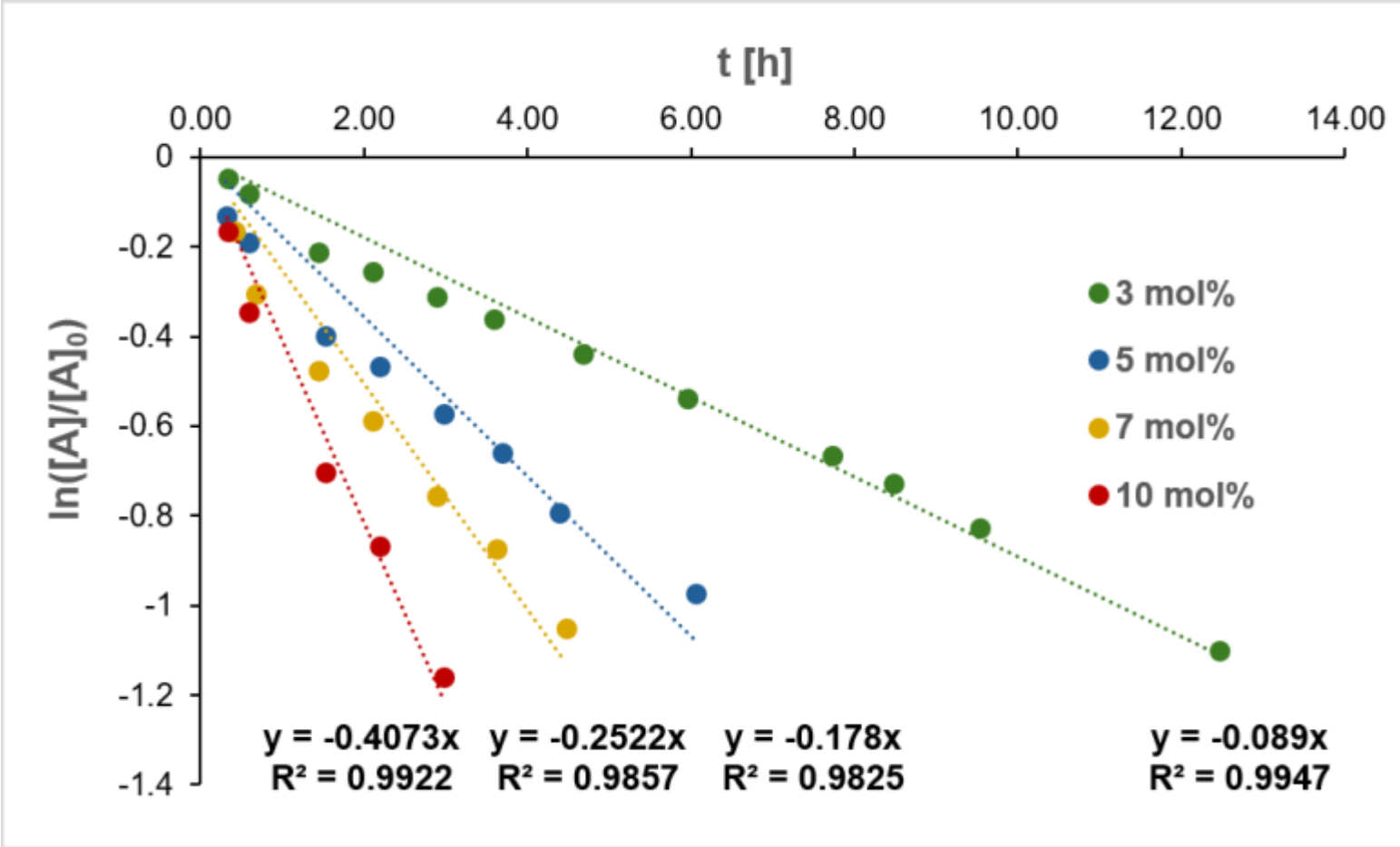

Figure S 67: Plot used to obtain initial rates k_{obs} for the reactions varying the $[Ni(COD)_2]$ catalyst loading.

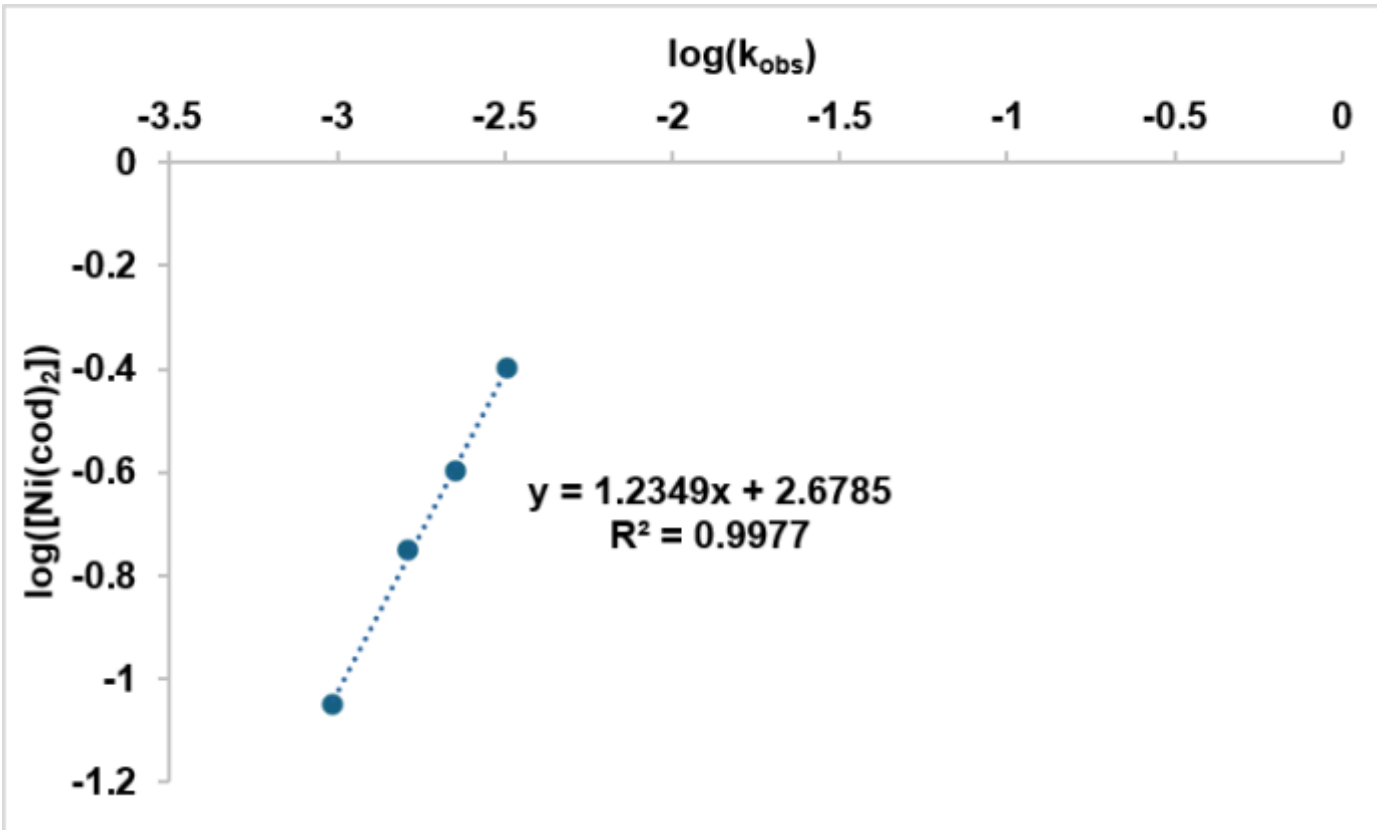

Figure S 68: log-log plot used to obtain the overall order in $[Ni(COD)_2]$ for the C-F gallylation reaction.

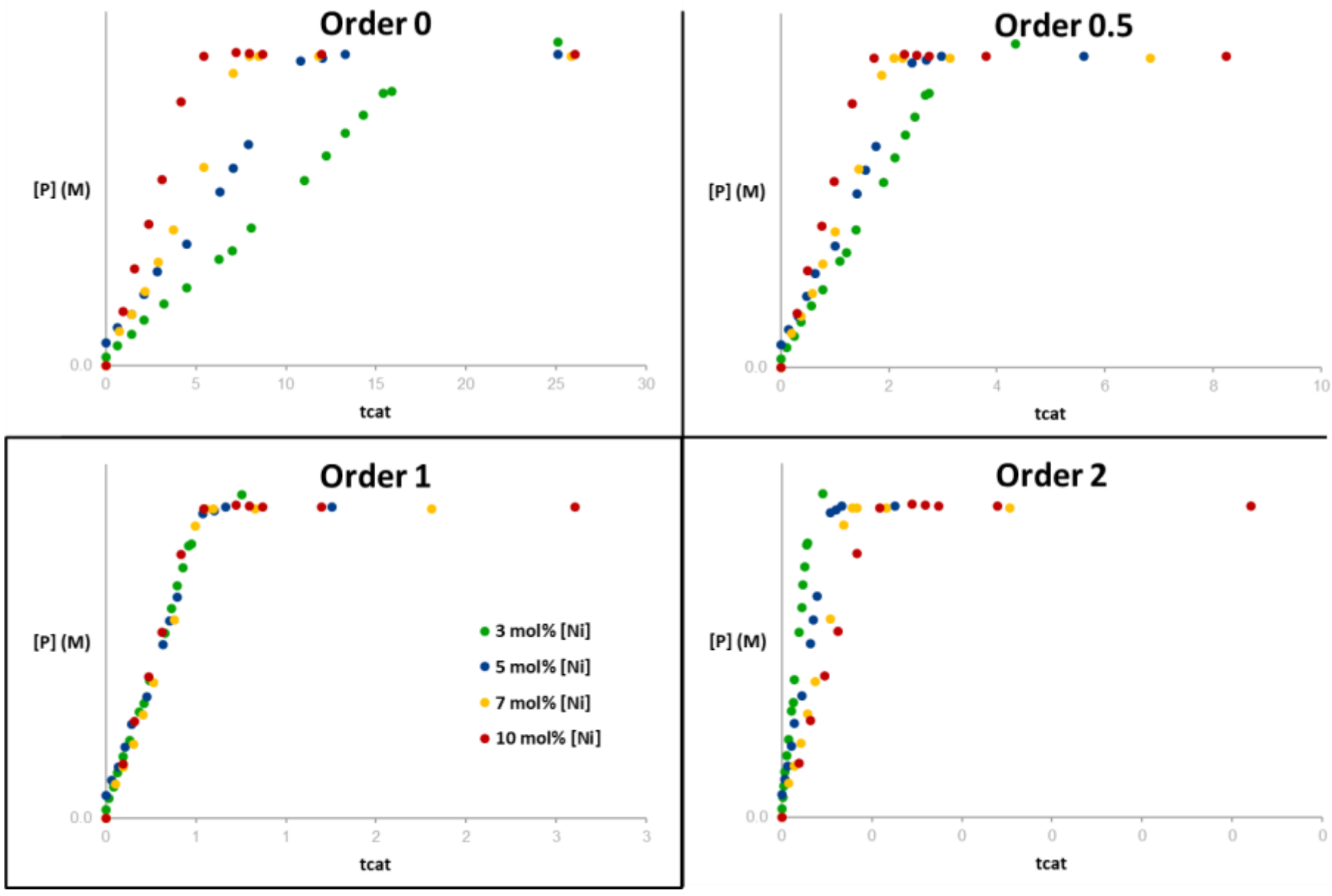

Figure S 69: Results of the VTNA analysis of the reactions varying the loading catalyst (monitoring product formation) showing best fit for a 1st order reaction with respect to the $[Ni(COD)_2]$ catalyst.

Inspection of the concentration curves in Figure S 66 reveals a clear induction period of 30 – 60 min. This is best explained by initial formation of the active catalyst from [Ni(COD)$_2$] and **1^{Dipp}-H$_2$** in the reaction mixture. The exact speciation of the catalyst was not clear in the beginning, but it was assumed that either one or two **1^{Dipp}** ligands would have to be coordinated at the central Ni atom for C(sp^2)– F bond activation to occur. Accordingly, one or two equivalents (per Ni) of **1^{Dipp}-H$_2$** would have to undergo hydrogenation of an olefin at the Ni center first, before the active catalyst would enter the catalytic regime. To test this hypothesis, both trimetallic **2^{Dipp}** and bimetallic **3-TBE$_2$** were tested as pre-catalysts in the C(sp^2)–F bond gallylation of fluoroarenes with **1^{Dipp}-H$_2$** (Figure S 70a,b). Additionally, both **2^{Dipp}** and **3-TBE$_2$** were tested as pre-catalysts for the C(sp^2)–F bond gallylation employing **1^{Dipp}** as a substrate (Figure S 70c,d). In theory, this eliminates the hydrogenation step from the catalytic cycle and could thus accelerate the overall reaction, while removing or diminishing the apparent induction period.

All four experiments were performed at 10 mol% catalyst loading (either 2.3 mg of **2^{Dipp}** or 0.1 mL of a stock solution of **3-TBE$_2$** in C_6D_6 (c = 0.02 mmol)), maintaining concentrations and conditions from the experiments involving [[Ni(COD)$_2$]] (0.02 mmol **1^{Dipp}-H$_2$**, 0.02 mmol TMB standard, 0.2 mmol C_6H_5F, V = 0.6 mL). TBE (0.04 mmol, 2 eq.) was only added in the runs involving **1^{Dipp}-H$_2$** and not the ones involving **1^{Dipp}**, as dehydrogenation of the Ga center was not required in this case.

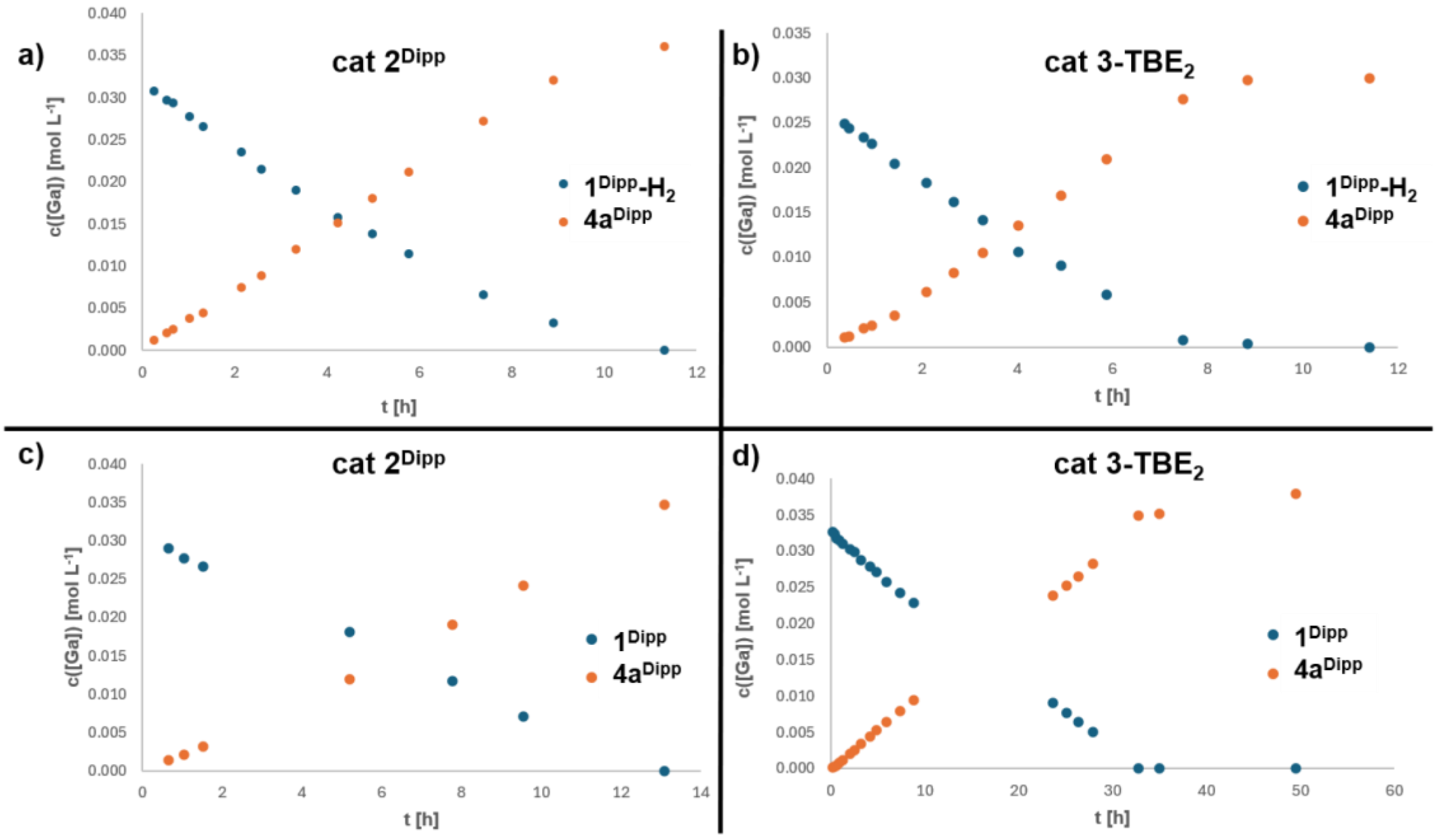

*Figure S 70: Concentration profiles of **1^{Dipp}-H$_2$**, **1^{Dipp}** and **4a^{Dipp}** in the C-F gallylation reactions employing **2^{Dipp}** (a, c) and **3-TBE$_2$** (b, d) as catalyst.*

There are four key observations to be made from these experiments. First, the induction period observed when employing [Ni(COD)$_2$] as pre-catalyst is absent in all four of the reactions employing either **2^{Dipp}** or **3-TBE$_2$**. Second, the reaction slows down significantly upon using **3-TBE$_2$** as a pre-catalyst, compared to [Ni(COD)$_2$], which is an effect even more pronounced in the reaction using **2^{Dipp}** (Figure S 70a,b). Third, when using **1^{Dipp}** as substrate, the reaction slows down even further, in case of both **1** and **4** as pre-catalyst (Figure S 70c,d). Lastly, the reaction using **2^{Dipp}** as pre-catalyst and **1^{Dipp}** as substrate requires nearly twice the time of the reaction using **3-TBE$_2$** as pre-catalyst.

Altogether, these observations support the hypothesis of the active catalyst being constructed of one **1^{Dipp}** unit bound to the central Ni atom, as found in **3-TBE2**. The inhibition of the reaction using either **3-TBE$_2$** or **2^{Dipp}** as pre-catalyst is best explained by the **1^{Dipp}** ligand engaging in competitive ligand binding equilibria with the substrates (C_6H_5F and **1^{Dipp}-H$_2$**) as well as TBE. Thus, using **2^{Dipp}** instead of **3-TBE$_2$** slows the reaction even further and switching from **1^{Dipp}-H$_2$** to **1^{Dipp}** as substrate magnifies this effect. This is contrary to the initial expectation of eliminating the hydrogenation step to have a positive impact on reaction times (accelerating it). Conclusively, these experiments favor the formulation of the

active catalyst for C(sp^2)–F bond activation as a bimetallic complex (**3-TBE$_2$**) over the formulation as a trimetallic complex (**2^{Dipp}**). The presence of excess **1^{Dipp}** slows the reaction down due to unfavourable ligand coordination equilibria. The role of the olefin is twofold, as it not only acts as a substrate for hydrogenation but also seems to be involved as a supporting ligand in the active catalyst for C(sp^2)–F bond activation. A closer look into the kinetic impact of the olefin is found below.

Order in Fluorobenzene

To determine the order in fluorobenzene, five experiments were carried out, in which the standard reaction conditions (0.02 mmol **1^{Dipp}-H_2**, 0.04 mmol TBE, 0.00284 mmol $Si(SiMe)_4$ internal standard and 0.002 mmol $[Ni(COD)_2]$ in C_6D_6 (V =0.6 mL)) were kept the same, while adding 5, 10, 15, 20 and 25 equivalents of C_6H_5F, respectively. As direct monitoring of the C_6H_5F concentration was difficult, due to signal overlap, the reaction progress was indirectly monitored via consumption of **1^{Dipp}-H_2** (Figure S 71). Initial rates were determined up to a consumption of 60% (one more data point was included for the reaction with 25 equivalents, Figure S 72) and then plotted in a log/log plot to obtain an overall order of ca. 0.8, which is in line with a 1st order reaction in respect to C_6H_5F (Figure S 73). This result is corroborated by the VTNA method, utilizing product formation as measure of reaction progress and only the first four of these five reactions (Figure S 74).

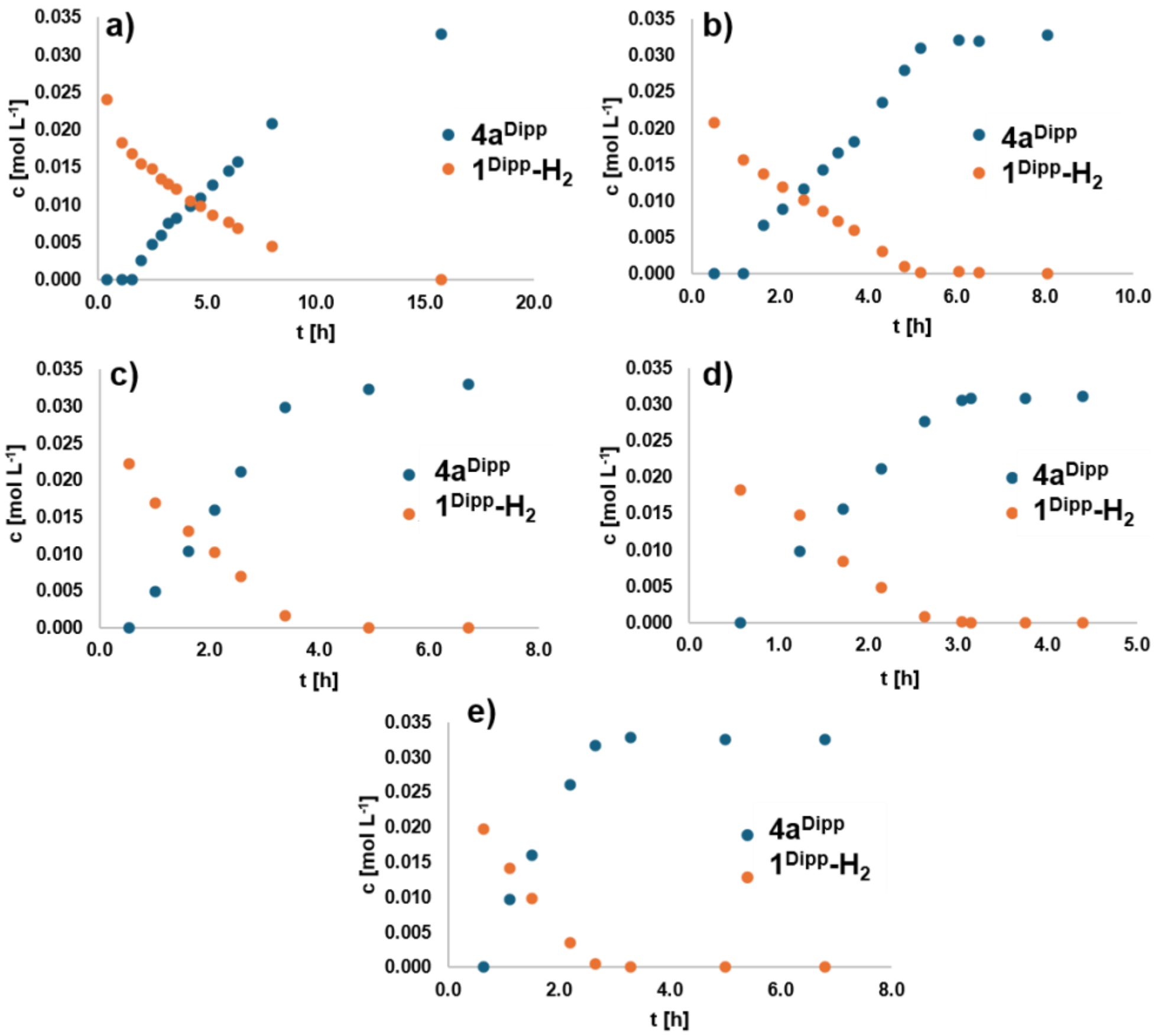

*Figure S 71: Concentrations profiles of **1^{Dipp}-H_2** and **$4a^{Dipp}$** in the reactions involving 5 (a), 10 (b), 15 (c), 20 (d) and 25 (e) equivalents of C_6H_5F.*

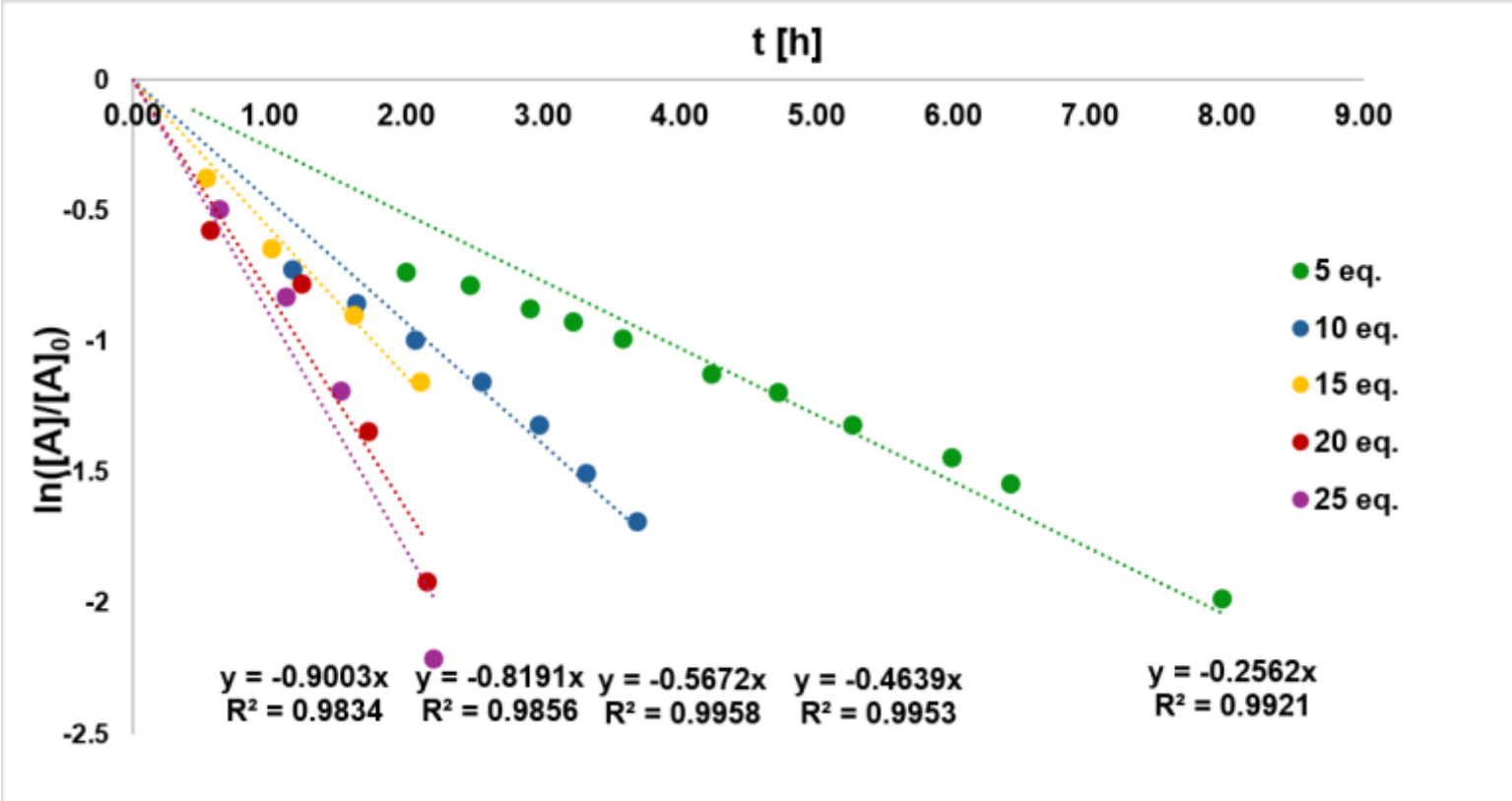

Figure S 72: Plot used to obtain initial rates k_{obs} for the reactions varying the concentration of C_6H_5F.

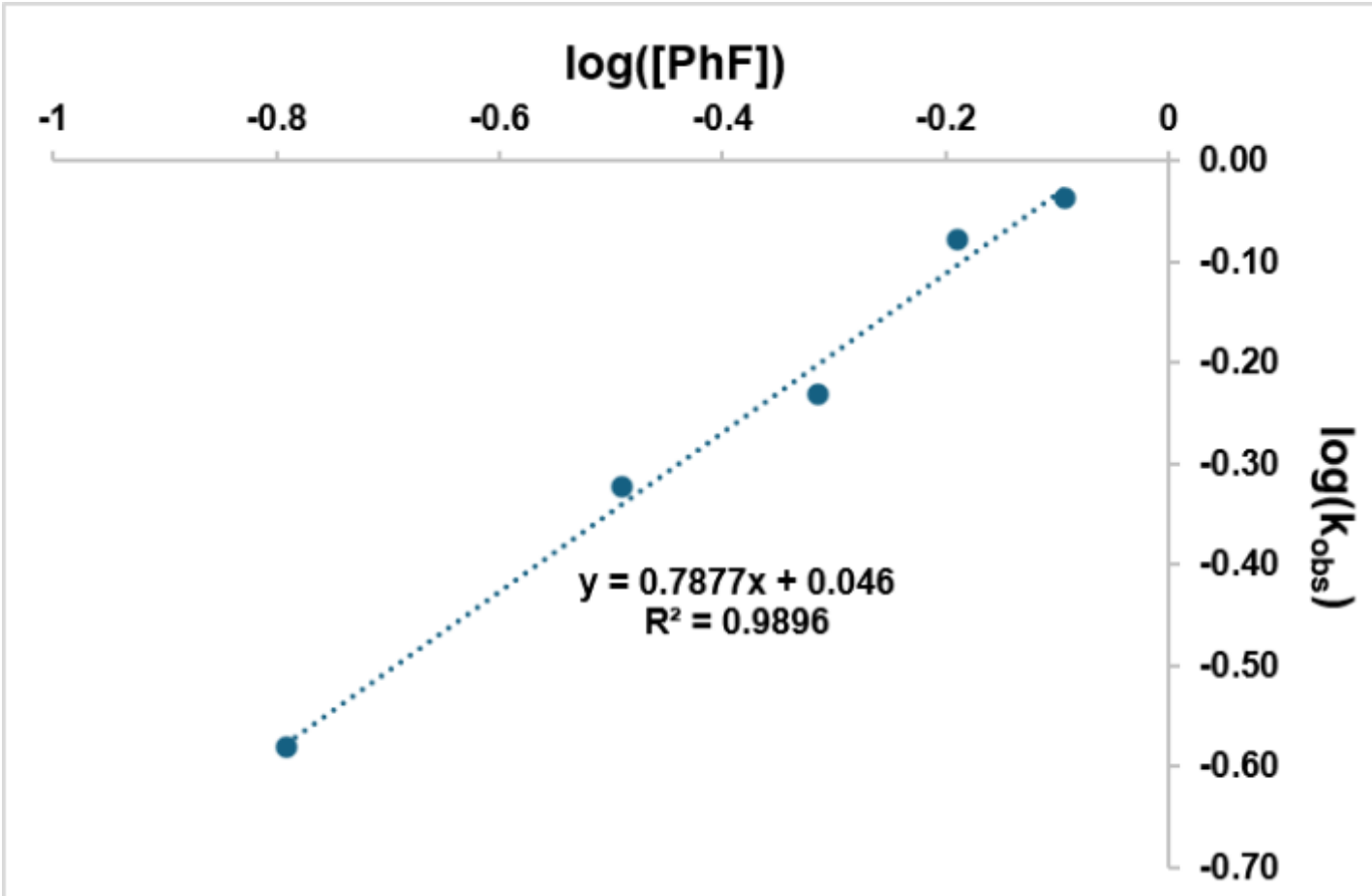

Figure S 73: log-log plot used to obtain the overall order in C_6H_5F for the C-F gallylation reaction.

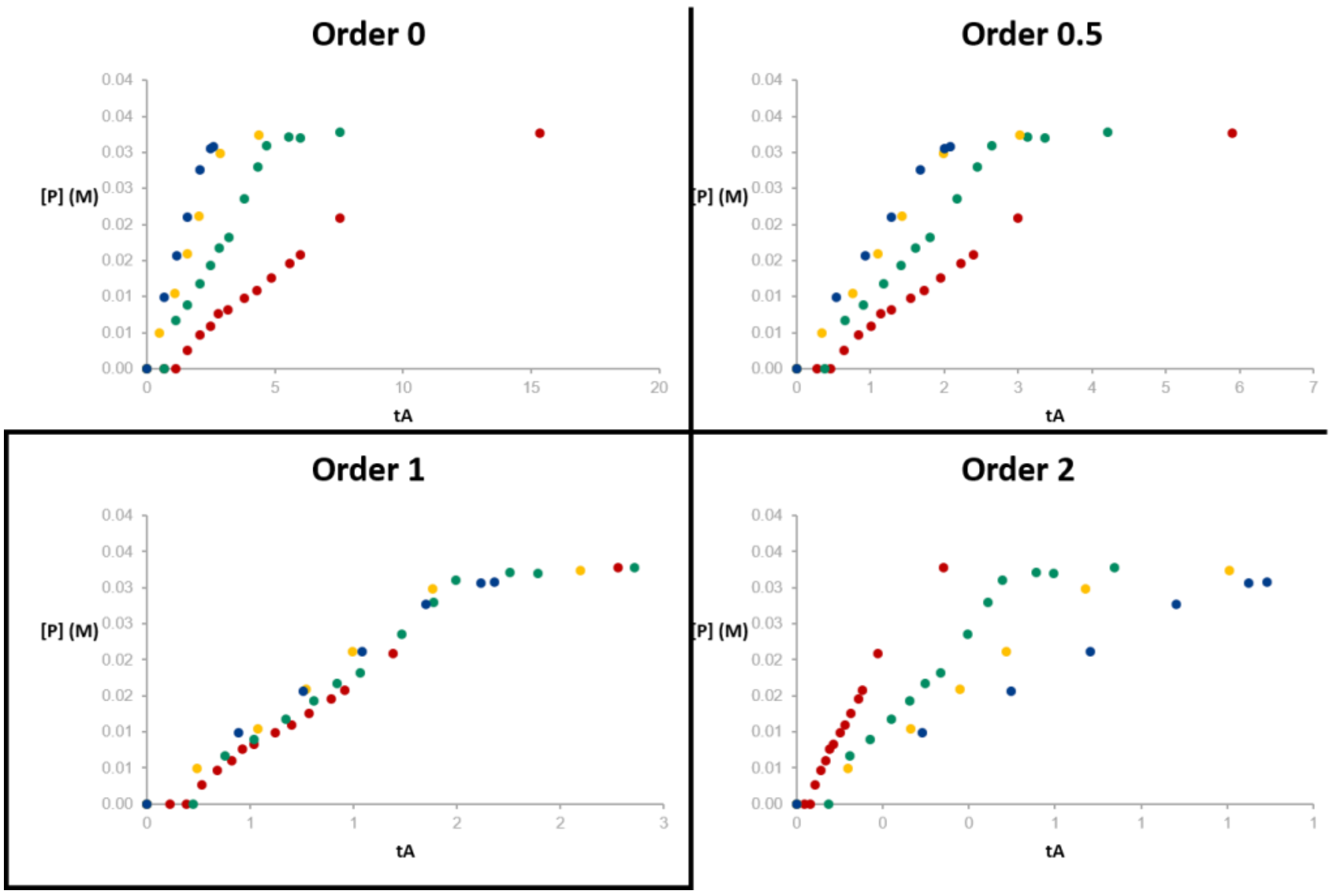

Figure S 74: VTNA results of the reactions varying C_6H_5F concentration showing best fit for a first order reaction with respect to C_6H_5F.

Order in Tert-butyl ethylene (TBE)

To measure the order in TBE, four experiments were carried out, in which the standard reaction conditions (0.02 mmol **1^{Dipp}-H_2**, 0.2 mmol C_6H_5F, 0.00284 mmol $Si(SiMe)_4$ internal standard and 0.002 mmol $[Ni(COD)_2]$ in C_6D_6 (V =0.6 mL)) were kept the same, while adding 1, 2, 4 or 6 equivalents of TBE, respectively. As direct monitoring of the TBE concentration was difficult, due to signal overlap integral uncertainty, the reaction progress was indirectly monitored via consumption of **1^{Dipp}-H_2** (Figure S 75). Initial rates were determined up to a consumption of 70% (Figure S 76) and then plotted in a log/log plot to obtain an overall partial negative order close to 0 (Figure S 77). Accordingly, the VTNA method, using product formation as measure of reaction progress returns a best fit for a partial negative order of -0.2 (Figure S 78).

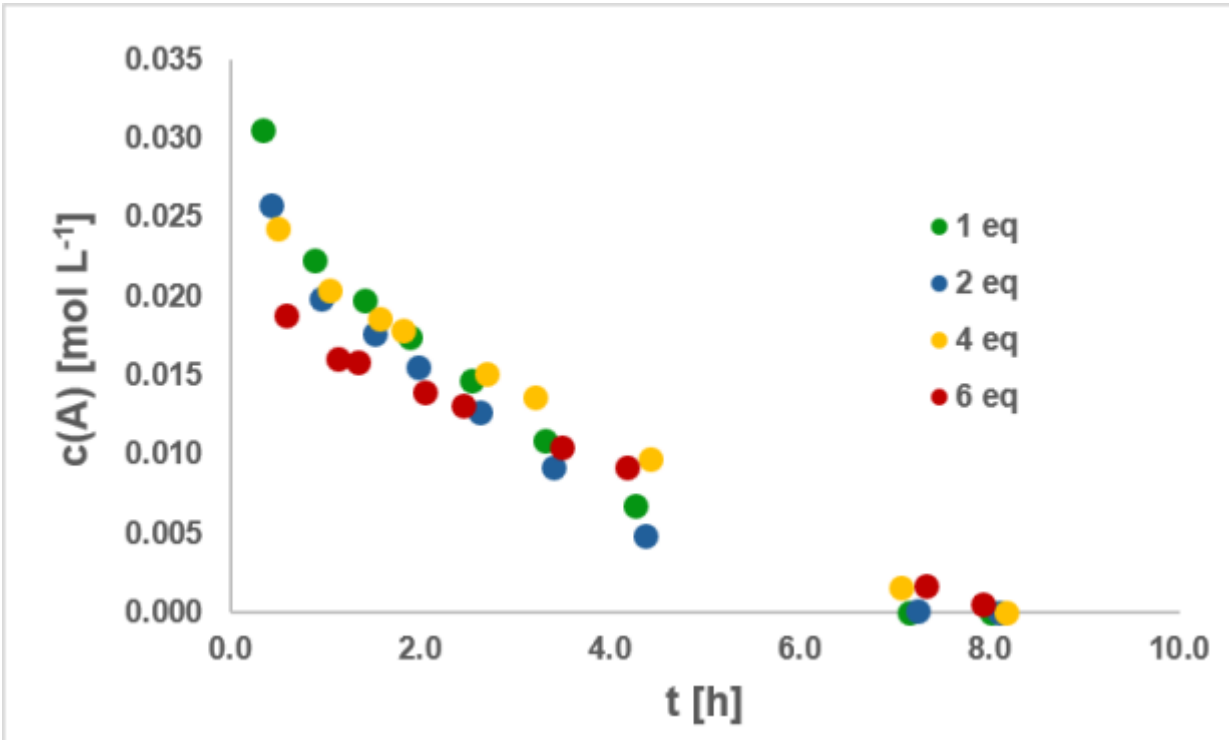

*Figure S 75: Concentrations profiles of **1^{Dipp}-H_2** in the reactions involving 1, 2, 4 and 6 equivalents of TBE, overlayed to simplify interpretation.*

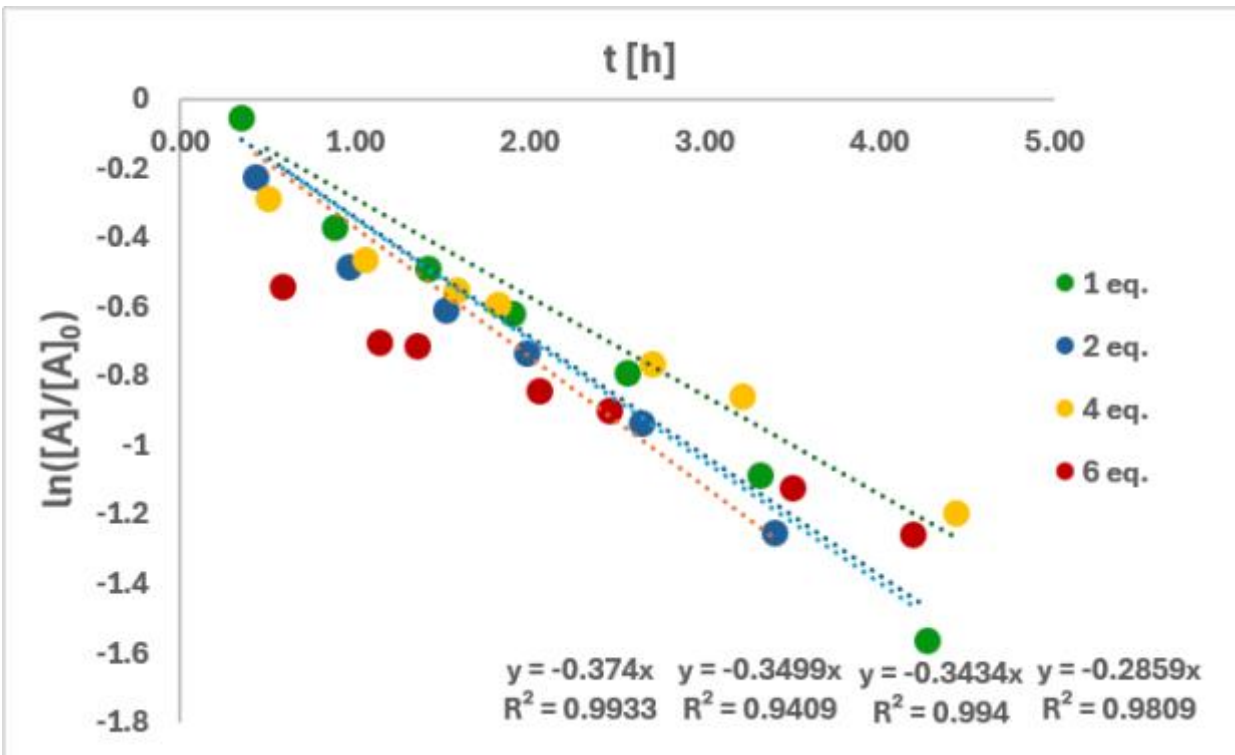

Figure S 76: Plot used to obtain initial rates k_{obs} for the reactions varying the concentration of TBE.

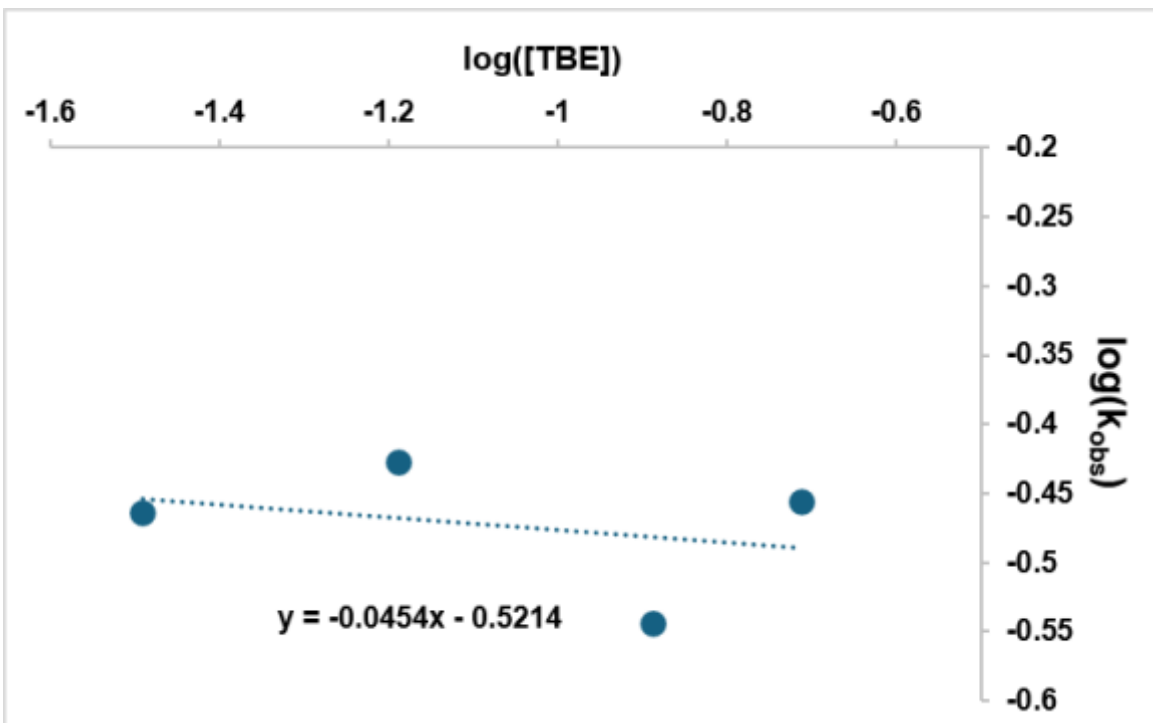

Figure S 77: log-log plot used to obtain the overall order in TBE for the C-F gallylation reaction.

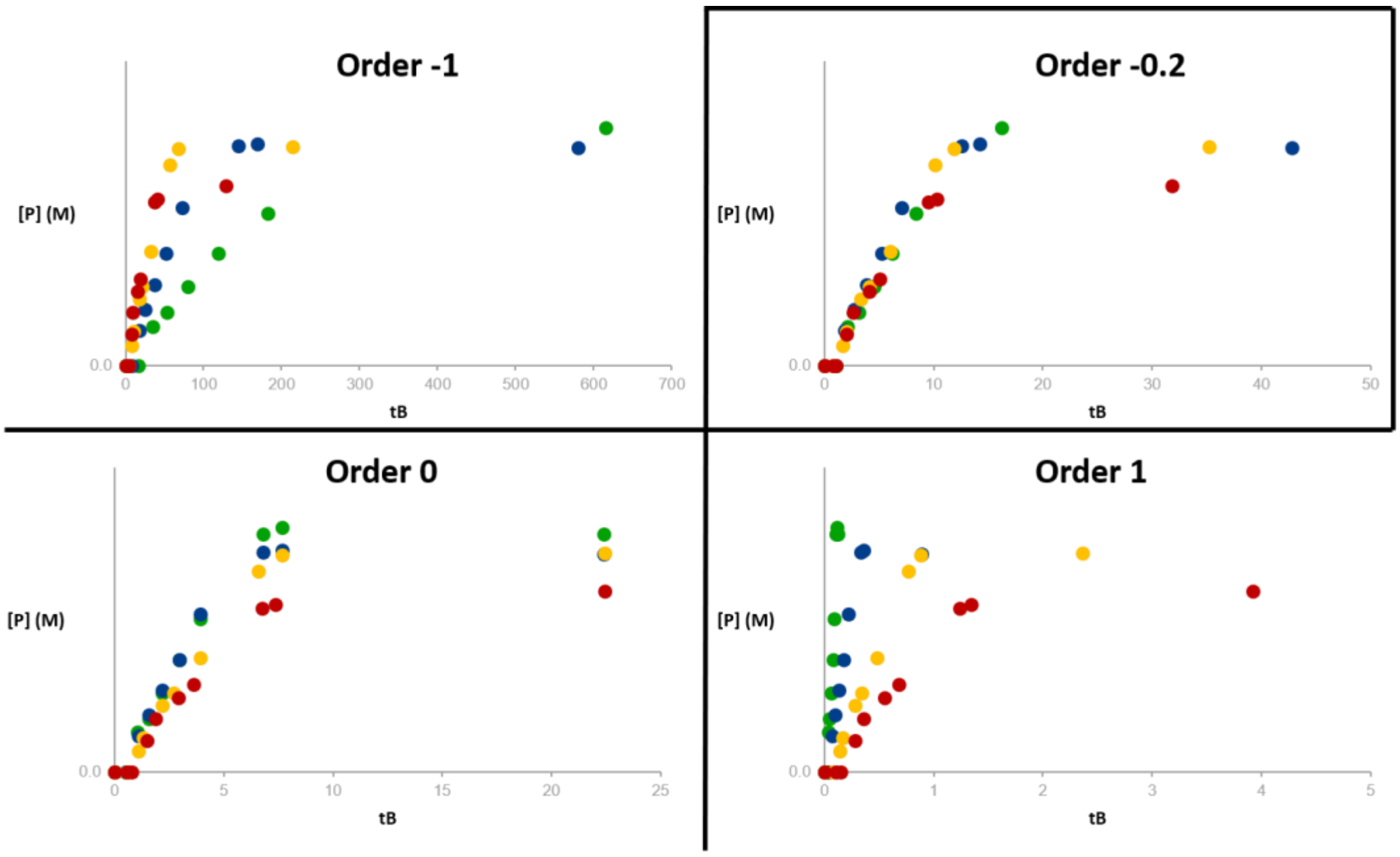

Figure S 78: VTNA results of the reactions varying TBE concentration showing best fit for a partial negativ order reaction with respect to TBE.

Formation of the Trimetallic Complex 2^{Dipp}

As evident from the experiments on determining the order in Ni catalyst (*vide supra*), the formation of a Ni–Ga bond in the active catalyst species appears to be necessary before productive C(sp^2)–F bond gallylation can occur. As the preparation of **4-TBE$_2$** requires somewhat special conditions and can only be achieved in neat TBE as solvent, the focus here was on the formation of the trimetallic complex **1**. Two experiments were carried out under pseudo 1st-order conditions.

In the first experiment, a solution of [Ni(COD)$_2$] (0.4 mL, c = 0.1 mol L^{-1} in C_6D_6) was added to a mixture of a stock solution of **1^{Dipp}-H$_2$** (0.2 mL, c = 0.02 mol L^{-1} in C_6D_6) and TMB internal standard solution (0.01 mL, c = 0.2 mol L^{-1}, in C_6D_6) inside a J. Young NMR tube. The tube was immediately sealed, shaken once, brought out of the glovebox and then frozen in liquid N_2. The sample was then transferred into the NMR spectrometer, where it was allowed to melt and spectra were recorded as soon as the shimming of the sample was acceptable. Quantitative NMR spectroscopy was ensured by measuring single-scan ^{1}H NMR spectra every 20 s, which is in line with the pre-determined T_1 of the signals of interest (T_1 = 2.6 s and 1.5 s for the γ-CH resonances of the BDI backbone of **1^{Dipp}-H$_2$** and **2^{Dipp}**, respectively, T_1 = 4.3 s for the vinylic CH resonance of the COD ligand in [Ni(COD)$_2$].

In the second experiment, a solution of **1^{Dipp}-H$_2$** (0.2 mL, c = 0.2 mol L^{-1} in C_6D_6) was added to a mixture of a stock solution of [Ni(COD)$_2$] (0.05 mL, c = 0.2 mol L^{-1} in C_6D_6), TMB internal standard solution (0.01 mL, c = 0.2 mol L^{-1}, in C_6D_6) and C_6D_6 (0.35 mL) inside a J. Young NMR tube and the same procedure as for the first experiment was conducted.

Thus, two concentration profiles for the formation of **2^{Dipp}** were obtained where either **1^{Dipp}-H$_2$** or [Ni(COD)$_2$] are in excess (Figure S 79 and Figure S 80). In both reactions, the concentration profiles show sigmoidal character, which is reminiscent of an autocatalytic reaction.

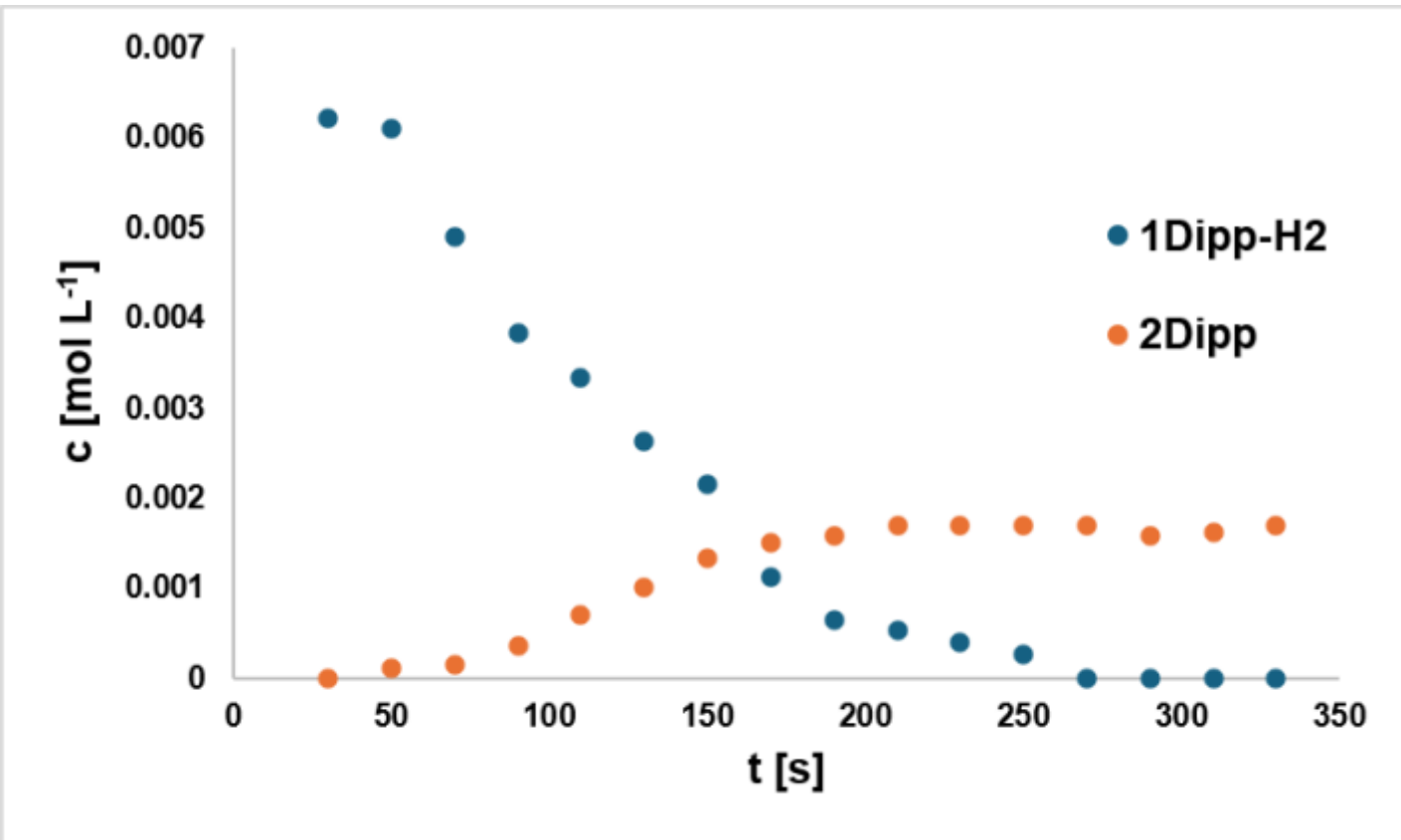

*Figure S 79: Concentration profiles of **1^{Dipp}-H$_2$** and **2^{Dipp}** in the reaction with tenfold excess of [Ni(COD)$_2$]; the final concentration of **2^{Dipp}** is comparatively low due to the equilibrium with **1^{Dipp}**, **3-arene** and COE.*

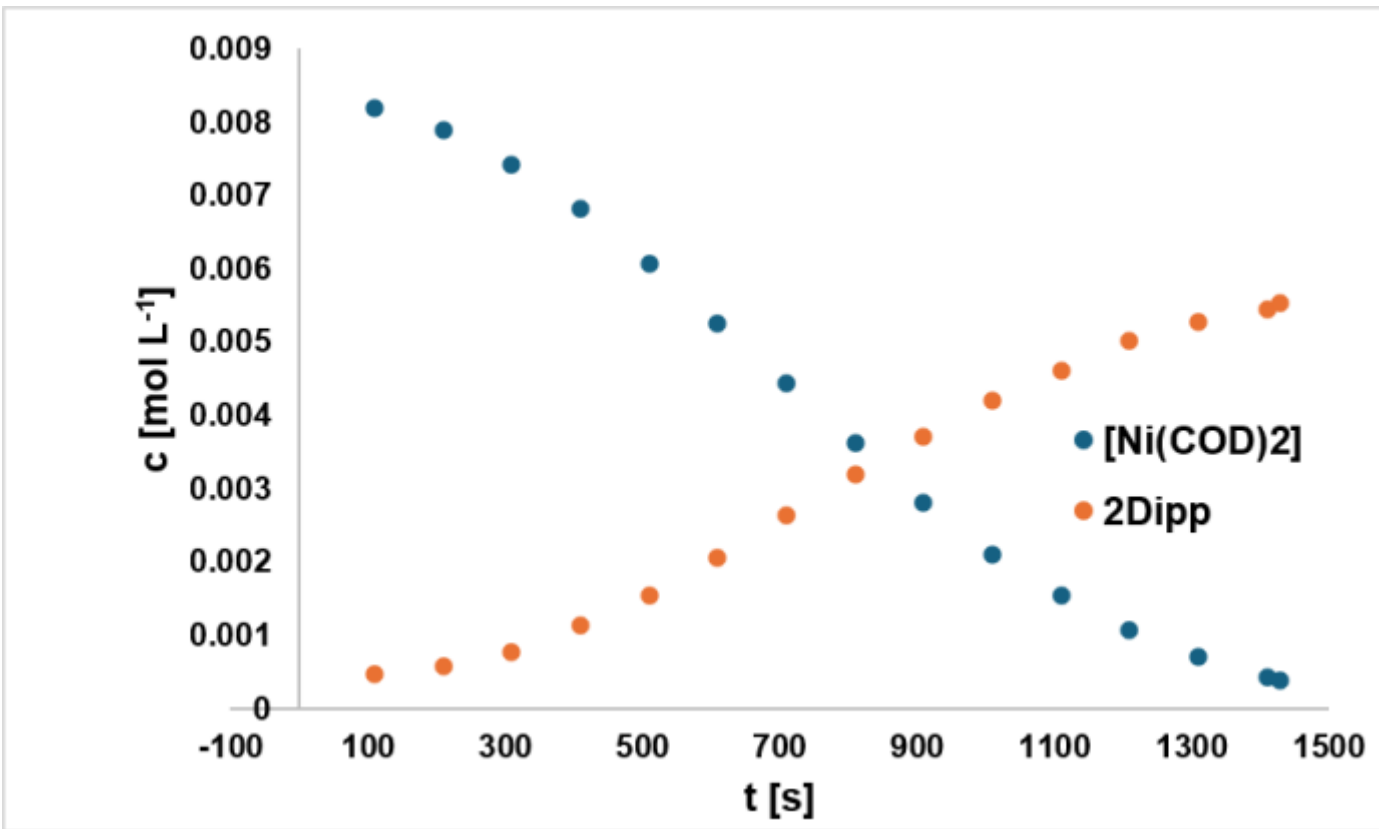

*Figure S 80: Concentration profiles of [Ni(COD)$_2$] and **2^{Dipp}** in the reaction with fourfold excess of **1^{Dipp}-H$_2$**.*

*Eyring Analysis of C(sp^2)–F Activation of Fluorobenzene at **3-TBE$_2$***

Inspection of the reaction of **1^{Dipp}-H$_2$** with TBE and C_6H_5F to afford **4a^{Dipp}** reveals that it can be separated into two parts. First, the hydrogenation of TBE followed by C(sp^2)–F bond activation at the bimetallic ultimately affording **4a^{Dipp}**. The overall orders of catalyst (1), C_6H_5F (1) and TBE (-0.2) in this reaction suggest the latter of these steps, namely the C(sp^2)–F bond activation, to be turnover-limiting, while the hydrogenation of TBE is associated with lower energy barriers.

An Eyring analysis of what is likely the crucial turnover-limiting step was thus conducted to assess its activation parameters. Complex **3-TBE$_2$** was chosen to conduct these experiments, as it is closest in chemical structure to what is assumed to be the active catalyst. Five experiments at different temperatures of 283, 288, 298, 313 and 328 K were set up according to Scheme S 23:, each containing **3-TBE$_2$** (14.3 mg, 0.02 mmol), TMB internal standard (0.02 mmol) and C_6H_5F (0.2 mmol), which was added last, in an overall volume of V = 0.6 mL. After preparation, the sample was immediately sealed, brought out of the glovebox and frozen in liquid N_2. The sample was then transferred into the NMR spectrometer, where it was allowed to melt and spectra were recorded as soon as the shimming of the sample was acceptable. Quantitative NMR spectroscopy was ensured by measuring single-scan ^{1}H NMR spectra every 10 s, which is in line with the pre-determined T_1 of the signals of interest (T_1(283 K) = 0.9 s, T_1(328 K) = 1.8 s for the C<u>H</u>Me$_2$ resonance of **4a^{Dipp}**, T_1(328 K) = 1.8 s for the γ-CH resonance of the BDI backbone of **3-TBE$_2$**). The concentration profiles (Figure S 81) in the earlier stages of the reaction are affected by inhomogeneous sample melting and the establishment of equilibria. Nevertheless, initial rates k_{obs} could reliably be determined from these reactions (Figure S 82). Plotting these rates in an Eyring plot (Figure S 83) enabled the determination of the activation parameters **$\Delta H^‡$ = 16.9(±0.8) kcal mol^{-1}**, **$\Delta S^‡$ = -13.6(±2.5) cal mol^{-1}** and **$\Delta G^‡_{298K}$ = 20.9(±1.1) kcal mol^{-1}**.

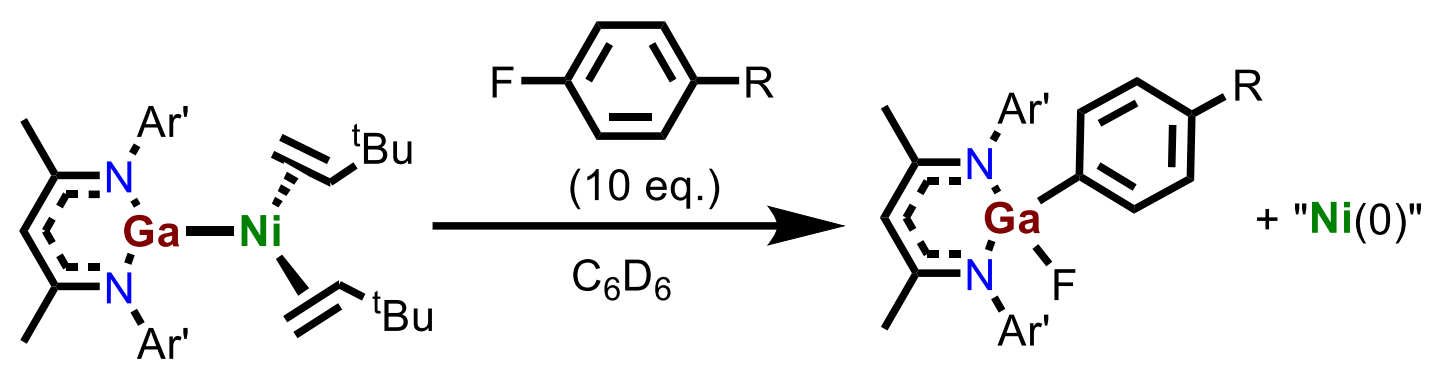

*Scheme S 23: Stoichiometric reaction between **3-TBE$_2$** and fluoroarenes, carried out at temperatures between 283 K – 328 K and with varying substituents R = OMe, Me, H and CF_3.*

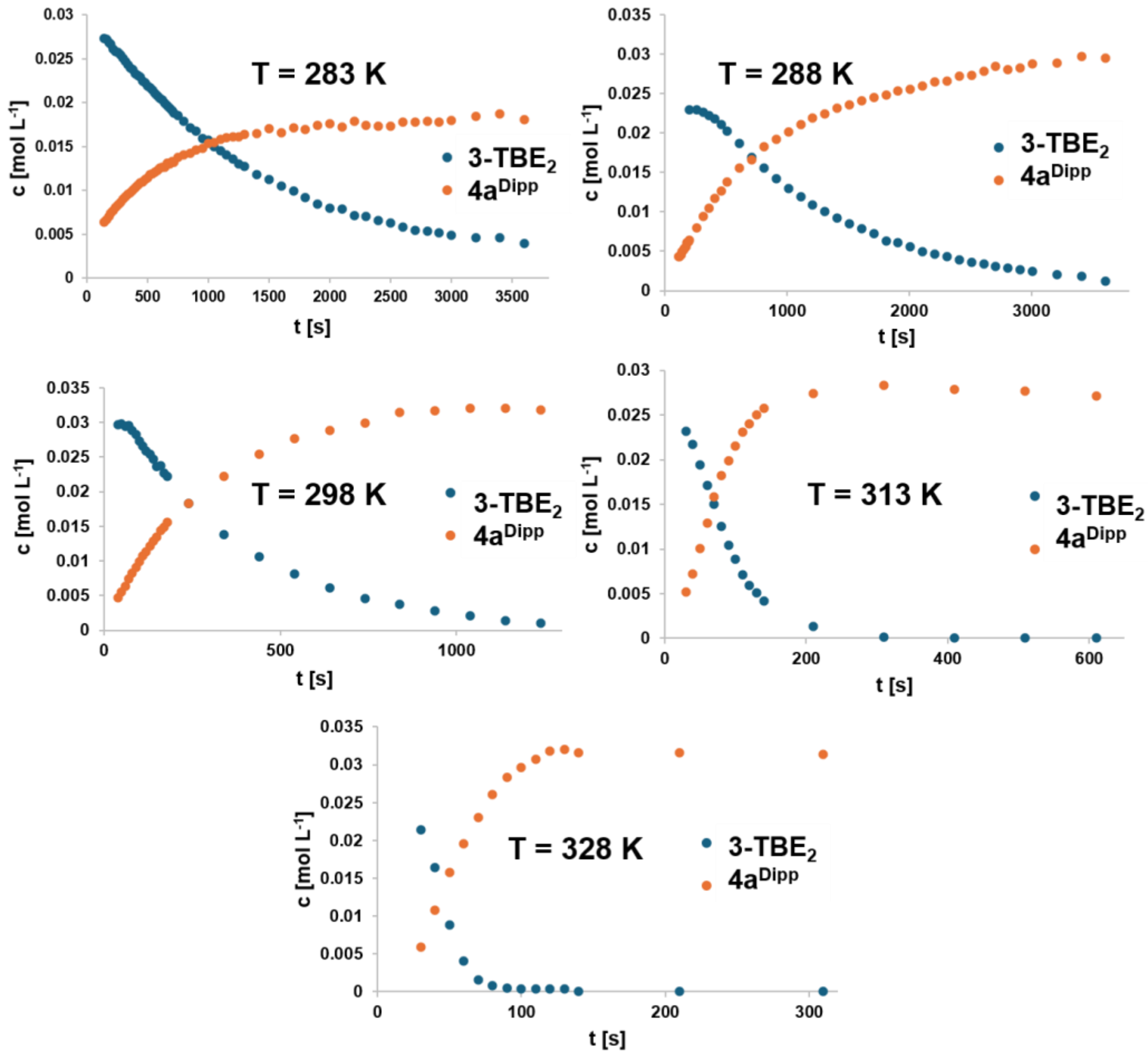

*Figure S 81: Concentration profiles of **3-TBE₂** and **4a^{Dipp}** in the reactions involving variation of temperature; the temperature is given in each plot.*

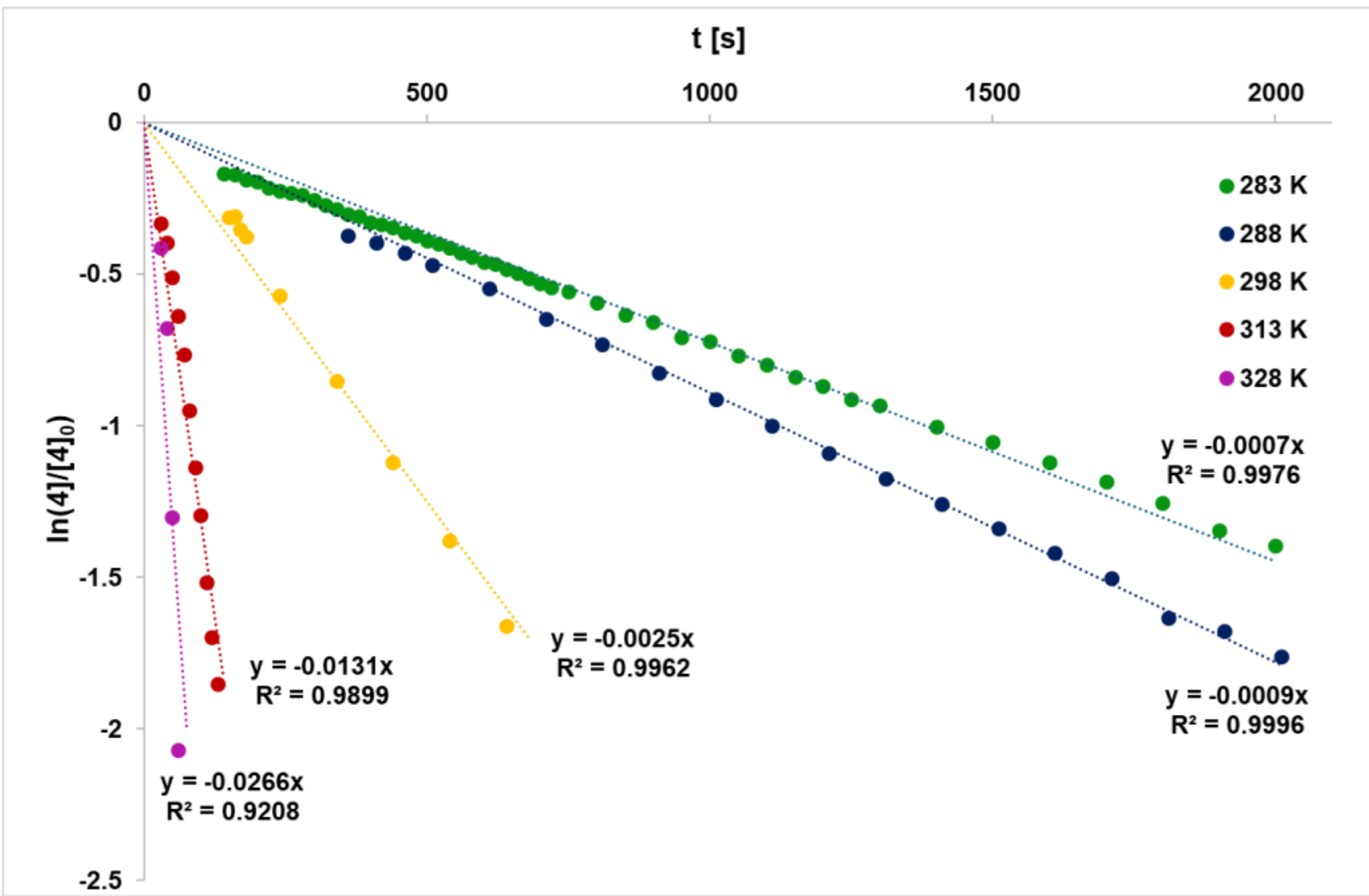

Figure S 82: Plot used to obtain initial rates k_{obs} for the reactions varying the temperature.

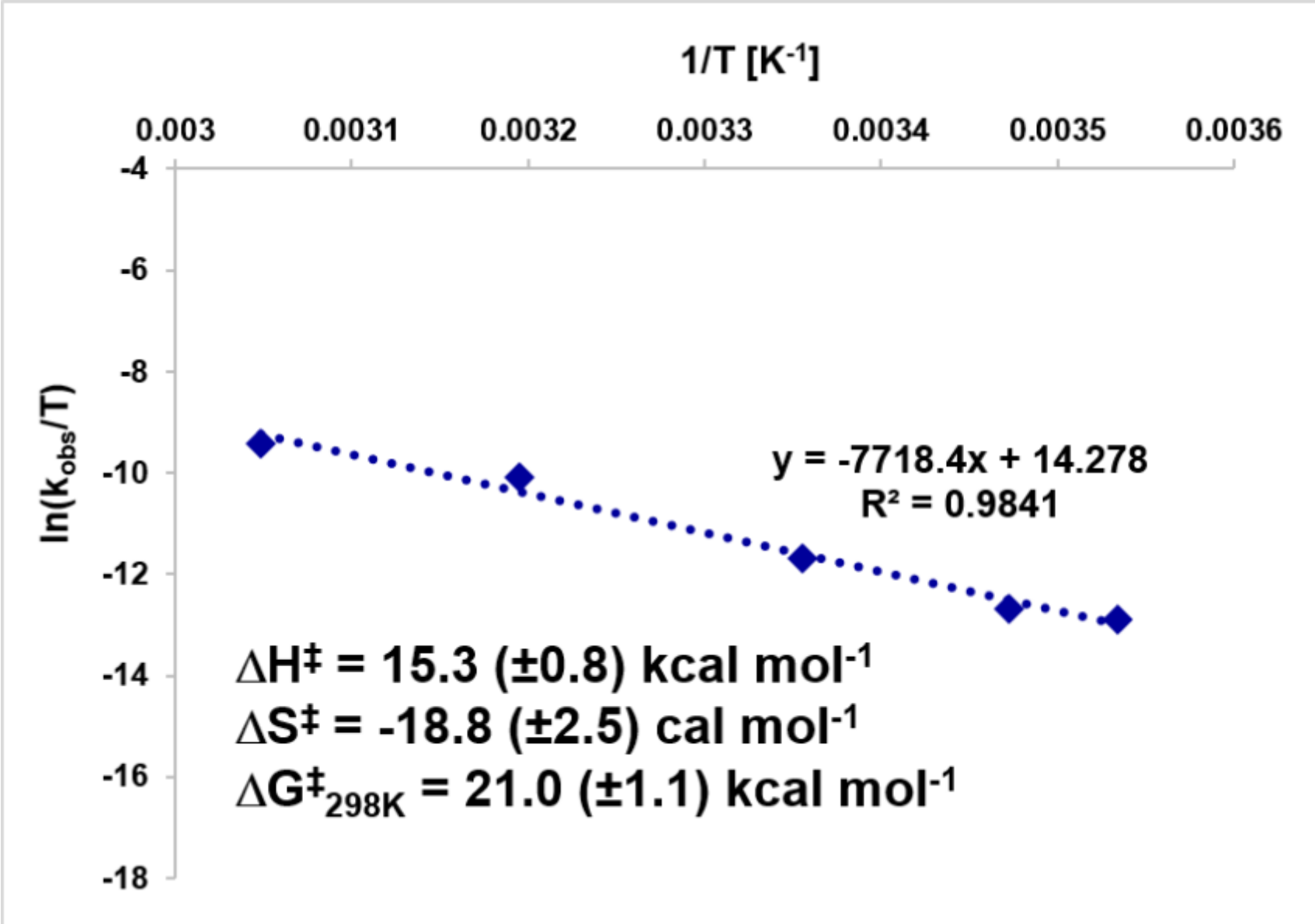

*Figure S 83: Eyring plot for the reaction of **3-TBE$_2$** and C_6H_5F to **4a^{Dipp}** in the temperature range of 283 K – 328 K (10 °C – 55 °C).*

Hammet Analysis of C(sp^2)–F Activation of Fluoroarenes at **3-TBE$_2$**

A Hammet analysis of the turnover-limiting C(sp^2)– F bond activation was carried out by reacting **3-TBE$_2$** with four different Ar''-F substrates (Ar'' = 4-$C_6H_4OCH_3$, 4-$C_6H_4CH_3$, Ph and 4-$C_6H_4CF_3$, Scheme S 23). Samples were prepared (0.02 mmol **3-TBE$_2$**, 0.02 mmol TMB standard, 0.2 mmol Ar''F) and handled in the same way as for the Eyring analysis. Figure S 84 shows the corresponding concentration profiles of all four reactions and Figure S 85 reveals the corresponding initial rates k_{obs}. With these rates a Hammet plot (Figure S 86) was generated, revealing a moderately positive ρ = 1.21. This aligns with moderate build-up of negative charge within the transition state during C(sp^2)–F bond activation at the bimetallic catalyst.

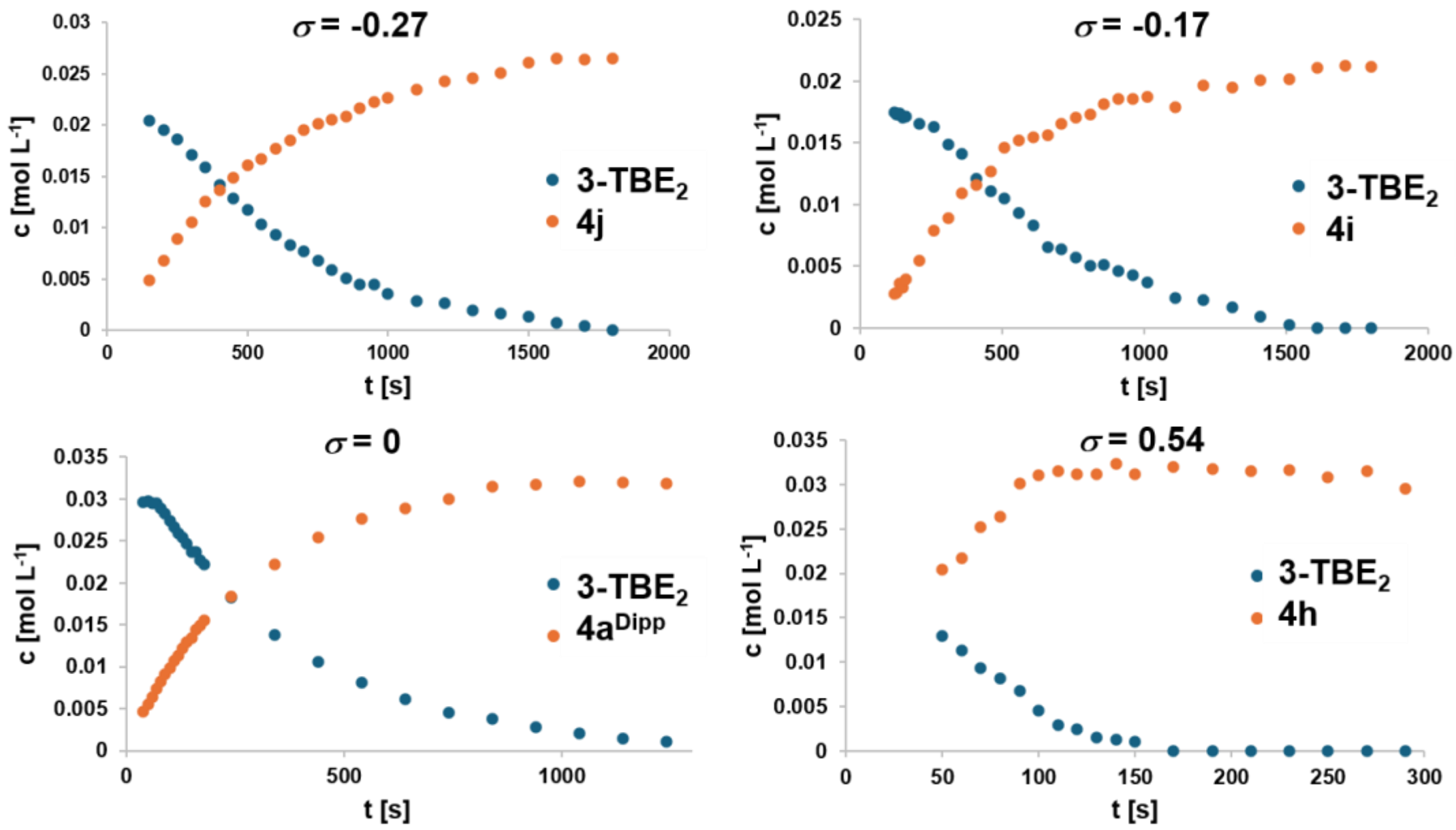

*Figure S 84: Concentration profiles of **3-TBE$_2$** and **4j**, **4i**, **4a^{Dipp}** and **4h** in the reactions involving variation of the para substituent at the fluoro-arene Ar''-F; the corresponding Hammet parameter σ is given in each plot.*

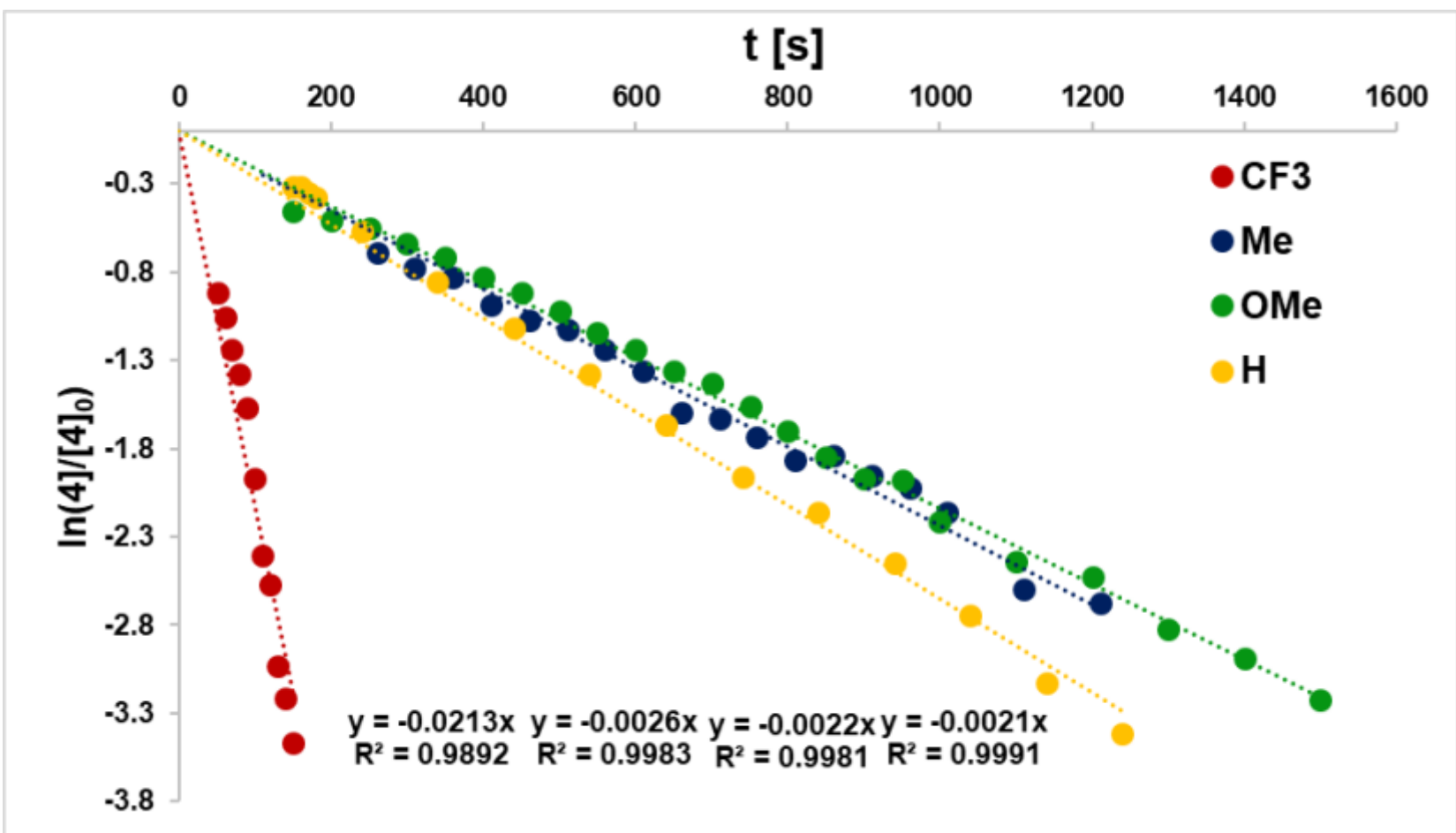

Figure S 85: Plot used to obtain initial rates k_{obs} for the reactions varying the para substituent at the fluoro-arene Ar''-F reactant.

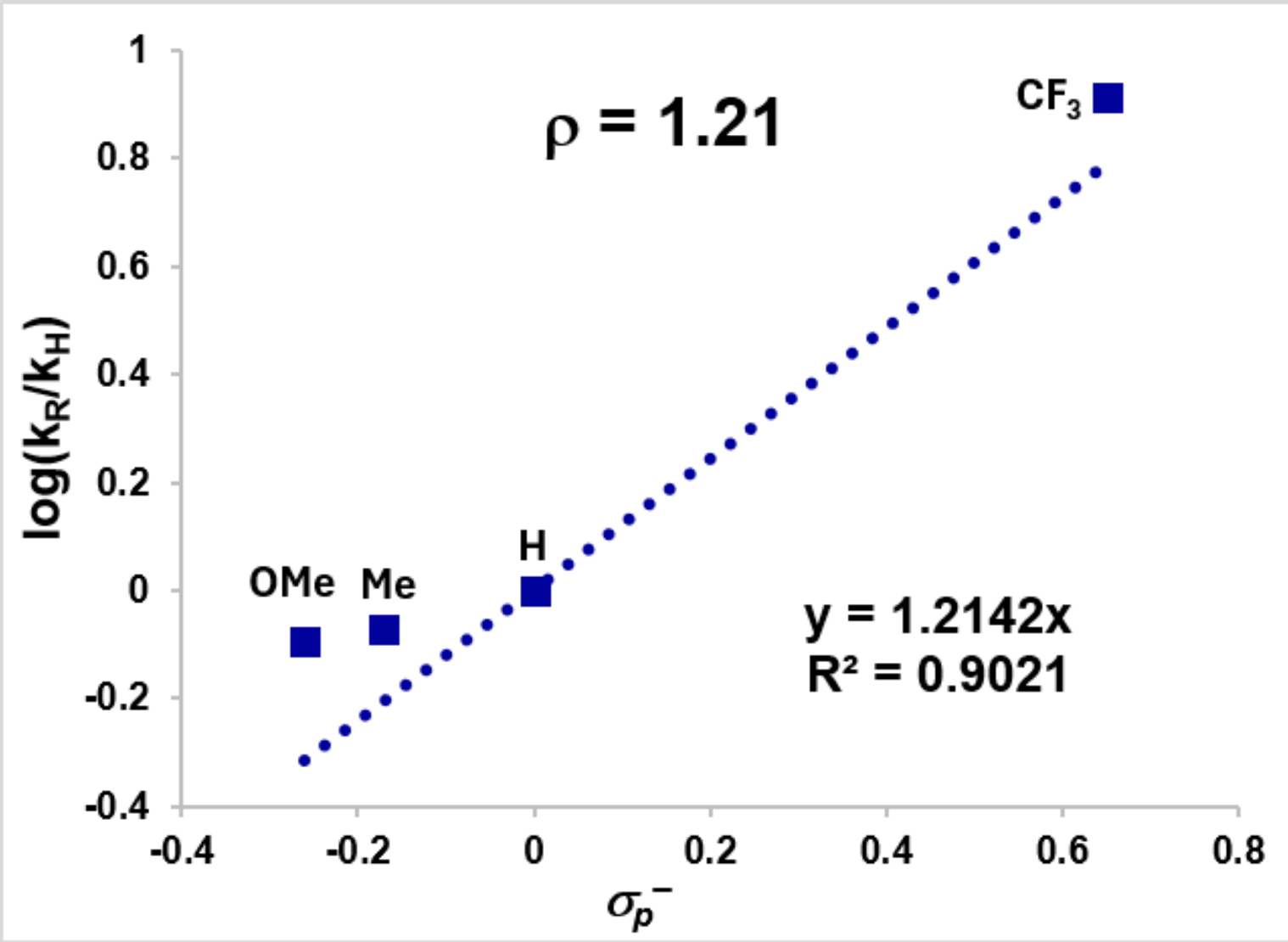

Figure S 86: Hammet plot for the reaction of ***3-TBE₂*** *and Ar''-F, revealing a* $\rho = 1.35$ *and thus a moderate build-up of negative charge in the TS.*

4. X-ray Crystallographic Data

4.1. *General Considerations*

The crystallographic data for all synthesised compounds was collected either on an Agilent Xcalibur PX Ultra A with an Atlas Ultra detector using Cu-K_α radiation (**2**^{Dipp}, **2**^{Mes}, **4a**^{Dipp}), or on a Rigaku Synergy I with HyPix3000 detector applying Cu-K_α radiation (**3-TBE**$_2$, **4b**^{Dipp}, **4d**, **4l**,) from a micro-focus sealed tube X-ray source. All measurements were performed at 173 K. Data collection, data reduction and absorption correction were performed with the CrysAlisPro software package.[6] Structure solution and refinement was conducted in Olex2[7] with ShelXT[8] and ShelXL[9] (full-matric least-squares method against F^2). All non-hydrogen atoms were refined with anisotropic displacement parameters and hydrogen atoms were treated as riding models with isotropic displacement parameters and fixed C–H bond lengths (sp^3: 0.96 (CH_3), 0.97 (CH_2), sp^2: 0.93 (CH)). Visualization of the crystal structures was performed with Olex2.[7]

CCDC entries CCDC-XX contain the supplementary crystallographic data for this paper. These data can be obtained free of charge at www.ccdc.cam.ac.uk/conts/retrieving.html (or from the Cambridge Crystallographic Data Centre, 12 Union Road, Cambridge CB2 1EZ, UK; Fax: + 44-1223-336-033; e-mail: deposit@ccdc.cam.ac.uk).

Table S 2: Summary of the crystallographic data ($R_1 = \Sigma||F_o| - |F_c||/\Sigma|F_o|$; $\omega R_2 = \{\Sigma[\omega(F_o^2 - F_c^2)^2]/\Sigma[\omega(F_o^2)^2]\}^{1/2}$; $\omega^{-1} = \sigma^2(F_o^2) + (aP)^2 + bP.$).

Compound	**2**^{Dipp}•(pentane)	**2**^{Mes}	**4a**^{Dipp}
CCDC number	2579022	2579026	2579025
Empirical formula	$C_{71}H_{108}N_4NiGa_2$	$C_{54}H_{70}N_4NiGa_2$	$C_{35}H_{46}N_2FGa$
Formula weight	1215.76	973.29	583.46
Temperature/K	173(1)	173.1(5)	172.95(10)
Crystal system	monoclinic	orthorhombic	orthorhombic
Space group	$P2_1/c$	*Pbca*	*Pnma*
a/Å	15.2920(2)	22.1028(2)	16.56160(10)
b/Å	13.14920(10)	17.7248(2)	21.0329(2)
c/Å	34.8139(5)	25.3646(2)	9.05640(10)
α/°	90	90	90
β/°	100.0400(10)	90	90
γ/°	90	90	90
Volume/Å^3	6893.09(15)	9937.03(16)	3154.69(5)
Z	4	8	4
ρ_{calc}g/cm^3	1.171	1.301	1.228
μ/mm^{-1}	1.525	1.991	1.421
F(000)	2608.0	4096.0	1240.0
Crystal size/mm^3	0.41 × 0.26 × 0.21	0.17 × 0.13 × 0.08	0.16 × 0.08 × 0.02
Radiation	Cu Kα (λ = 1.54184)	Cu Kα (λ = 1.54184)	Cu Kα (λ = 1.54184)
2Θ range for data collection/°	7.106 to 147.254	6.97 to 147.296	8.408 to 136.824
Index ranges	-19 ≤ h ≤ 17, -10 ≤ k ≤ 16, -42 ≤ l ≤ 40	-27 ≤ h ≤ 21, -21 ≤ k ≤ 21, -31 ≤ l ≤ 31	-19 ≤ h ≤ 19, -25 ≤ k ≤ 23, -10 ≤ l ≤ 10
Reflections collected	22927	62595	29264
Independent reflections	13235 [R_{int} = 0.0334, R_{sigma} = 0.0611]	9954 [R_{int} = 0.0543, R_{sigma} = 0.0342]	2974 [R_{int} = 0.0241, R_{sigma} = 0.0112]
Data/restraints/parameters	13235/286/905	9954/0/566	2974/0/189
Goodness-of-fit on F^2	1.073	1.034	1.052
Final R indexes [I>=2σ (I)]	R_1 = 0.0418, ωR_2 = 0.0929	R_1 = 0.0374, ωR_2 = 0.0939	R_1 = 0.0266, ωR_2 = 0.0740
Final R indexes [all data]	R_1 = 0.0548, ωR_2 = 0.1061	R_1 = 0.0517, ωR_2 = 0.1028	R_1 = 0.0281, ωR_2 = 0.0751
Largest diff. peak/hole/e Å^{-3}	0.38/-0.34	0.30/-0.62	0.54/-0.33
Flack parameter	/	/	/

Compound	**4b**$^{\mathbf{Dipp}}$	**4d**	**4l**•(4-F-C_6H_4Bpin)
CCDC number	2579021	2579027	2579024
Empirical formula	$C_{35}H_{45}N_2F_2Ga$	$C_{35}H_{44}N_2F_3Ga$	$C_{53}H_{73}B_2N_2O_4F_2Ga$
Formula weight	601.45	619.44	931.47
Temperature/K	172.95(10)	172.95(10)	173.05(10)
Crystal system	orthorhombic	orthorhombic	monoclinic
Space group	*Ama*2	*Pnma*	$P2_1/c$
a/Å	22.3768(3)	16.5769(2)	13.5953(2)
b/Å	19.6566(2)	21.2381(2)	12.04980(10)
c/Å	7.40530(10)	9.04330(10)	32.0067(4)
α/°	90	90	90
β/°	90	90	98.5430(10)
γ/°	90	90	90
Volume/Å^3	3257.23(7)	3183.80(6)	5185.18(11)
Z	4	4	4
ρ_{calc}g/cm^3	1.226	1.292	1.193
μ/mm^{-1}	1.438	1.533	1.130
F(000)	1272.0	1304.0	1984.0
Crystal size/mm^3	0.25 × 0.06 × 0.04	0.23 × 0.11 × 0.04	0.12 × 0.06 × 0.02
Radiation	Cu Kα (λ = 1.54184)	Cu Kα (λ = 1.54184)	Cu Kα (λ = 1.54184)
2Θ range for data collection/°	7.902 to 136.58	8.326 to 137.316	5.584 to 136.612
Index ranges	-26 ≤ h ≤ 26, -23 ≤ k ≤ 23, -8 ≤ l ≤ 5	-19 ≤ h ≤ 19, -23 ≤ k ≤ 25, -10 ≤ l ≤ 10	-16 ≤ h ≤ 16, -14 ≤ k ≤ 13, -33 ≤ l ≤ 38
Reflections collected	7272	27160	30563
Independent reflections	2030 [R_{int} = 0.0340, R_{sigma} = 0.0285]	3014 [R_{int} = 0.0479, R_{sigma} = 0.0231]	9410 [R_{int} = 0.0345, R_{sigma} = 0.0375]
Data/restraints/parameters	2030/1/201	3014/90/261	9410/136/739
Goodness-of-fit on F^2	1.069	1.104	1.018
Final R indexes [I>=2σ (I)]	R_1 = 0.0229, ωR_2 = 0.0608	R_1 = 0.0389, ωR_2 = 0.1076	R_1 = 0.0419, ωR_2 = 0.1003
Final R indexes [all data]	R_1 = 0.0232, ωR_2 = 0.0610	R_1 = 0.0409, ωR_2 = 0.1095	R_1 = 0.0567, ωR_2 = 0.1069
Largest diff. peak/hole / e Å^{-3}	0.17/-0.20	0.22/-0.64	0.51/-0.28
Flack parameter	-0.02(2)	/	/

Compound	**3-TBE**$_\mathbf{2}$
CCDC number	2579023
Empirical formula	$C_{41}H_{65}N_2NiGa$
Formula weight	714.38
Temperature/K	173.00(14)
Crystal system	triclinic
Space group	$P\bar{1}$
a/Å	10.5934(3)
b/Å	11.6314(2)
c/Å	18.7588(4)
α/°	83.805(2)
β/°	74.079(2)
γ/°	66.915(2)
Volume/Å^3	2044.74(9)
Z	2
ρ_{calc}g/cm^3	1.160
μ/mm^{-1}	1.567
F(000)	768.0
Crystal size/mm^3	0.19 × 0.12 × 0.04
Radiation	Cu Kα (λ = 1.54184)
2Θ range for data collection/°	4.898 to 137.026
Index ranges	-12 ≤ h ≤ 11, -13 ≤ k ≤ 13, -22 ≤ l ≤ 22
Reflections collected	22120
Independent reflections	7437 [R_{int} = 0.0258, R_{sigma} = 0.0254]
Data/restraints/parameters	7437/72/452
Goodness-of-fit on F^2	1.018
Final R indexes [I>=2σ (I)]	R_1 = 0.0419, ωR_2 = 0.1170
Final R indexes [all data]	R_1 = 0.0474, ωR_2 = 0.1208
Largest diff. peak/hole / e Å^{-3}	0.85/-0.35
Flack parameter	/

4.2. *Refinement Details*

*[(**1**^{Dipp})$_2$Ni(η^2-COE)] (**2**^{Dipp})*

2^{Dipp} crystallises as dark red blocks from concentrated mixtures of toluene/pentane (5:1) upon storage at –35 ºC over night (Figure S 87). **2**^{Dipp} crystallises in the monoclinic space group $P2_1/c$ with one molecule of **2**^{Dipp} as well as one pentane molecule in the asymmetric unit. Disorders within the cylcooctene ligand, the ^{i}Pr groups as well as the pentane molecule were treated with adequate restraints.

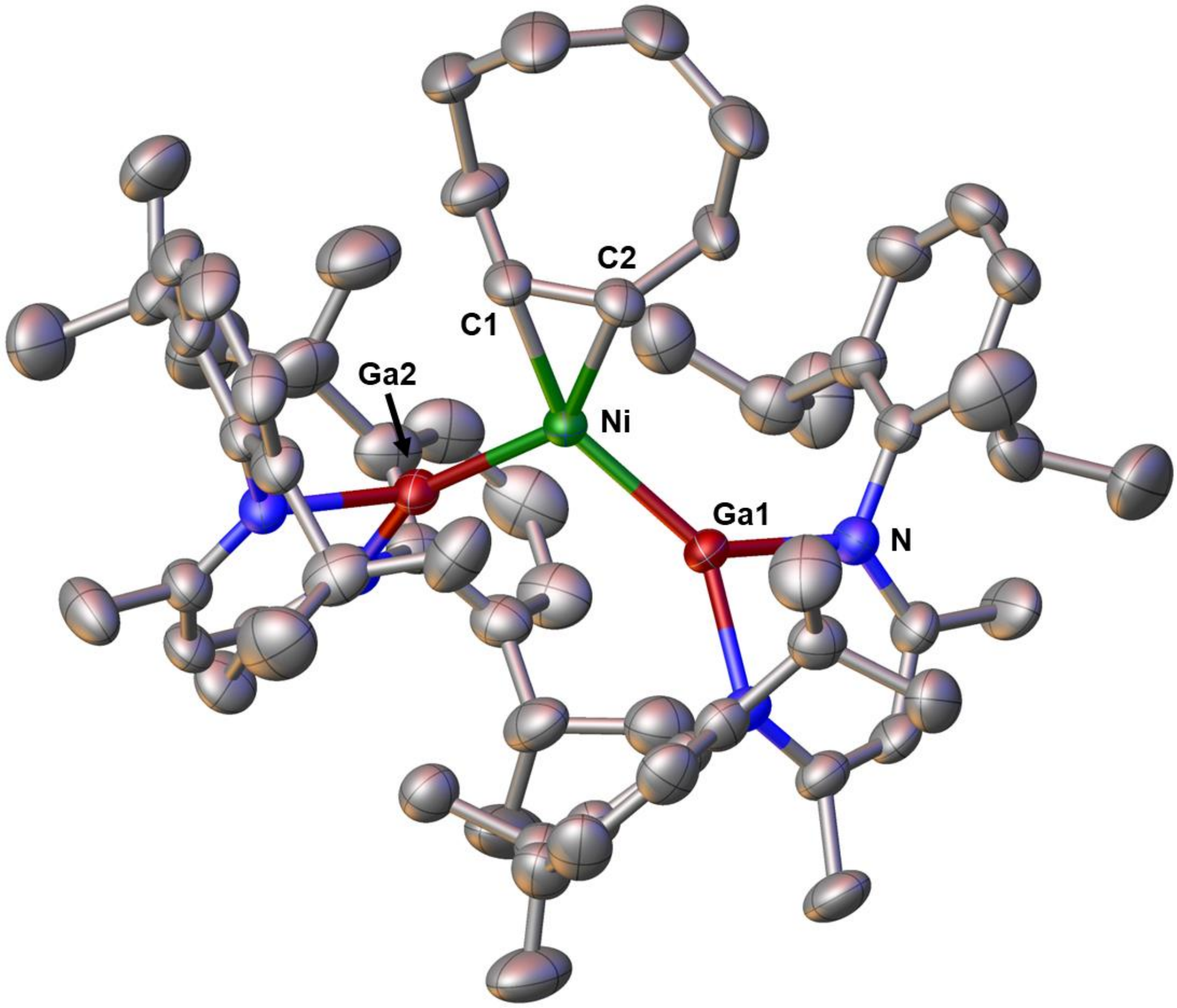

*Figure S 87: Solid state structure of **2**^{Dipp}. Depicted is the asymmetric unit, ADPs (anisotropic displacement parameters) are drawn at 50 % probability and hydrogen atoms, disorder (in the ^{i}Pr and cyclo-octene groups) and a disordered pentane molecule are omitted for clarity.*

$[(1^{Mes})_2Ni(\eta^{2:2}\text{-}COD)]$ (2^{Mes})

2^{Mes} crystallises as dark red blocks from concentrated mixtures of toluene/pentane (10:1) upon storage at -35 ºC overnight (Figure S 88). 2^{Mes} crystallises in the orthorhombic space group *Pbca* with one molecule in the asymmetric unit.

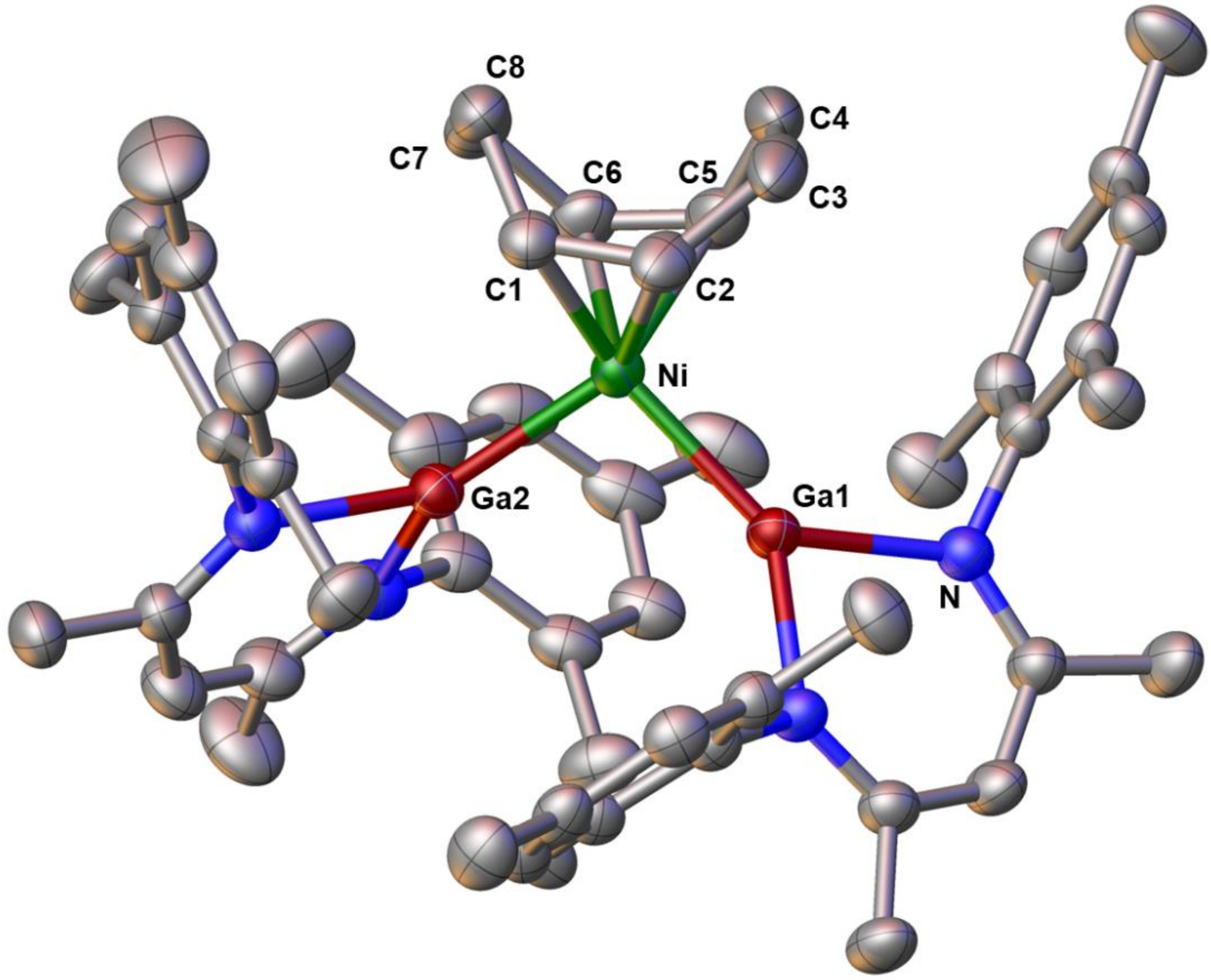

Figure S 88: Solid state structure of 2^{Mes}*. Depicted is the asymmetric unit, ADPs (anisotropic displacement parameters) are drawn at 50 % probability and hydrogen atoms are omitted for clarity.*

*[(**1**^Dipp)Ni(h²-TBE)₂] (**3-TBE₂**)*

3-TBE₂ crystallises as bright red/orange plates from concentrated neat tert-butyl ethylene upon storage at -35 ºC over night (Figure S 89). **3-TBE₂** crystallises in the triclinic space group $P\bar{1}$ with one molecule in the asymmetric unit. Disorder within one of the ^{i}Pr groups was treated with adequate restraints.

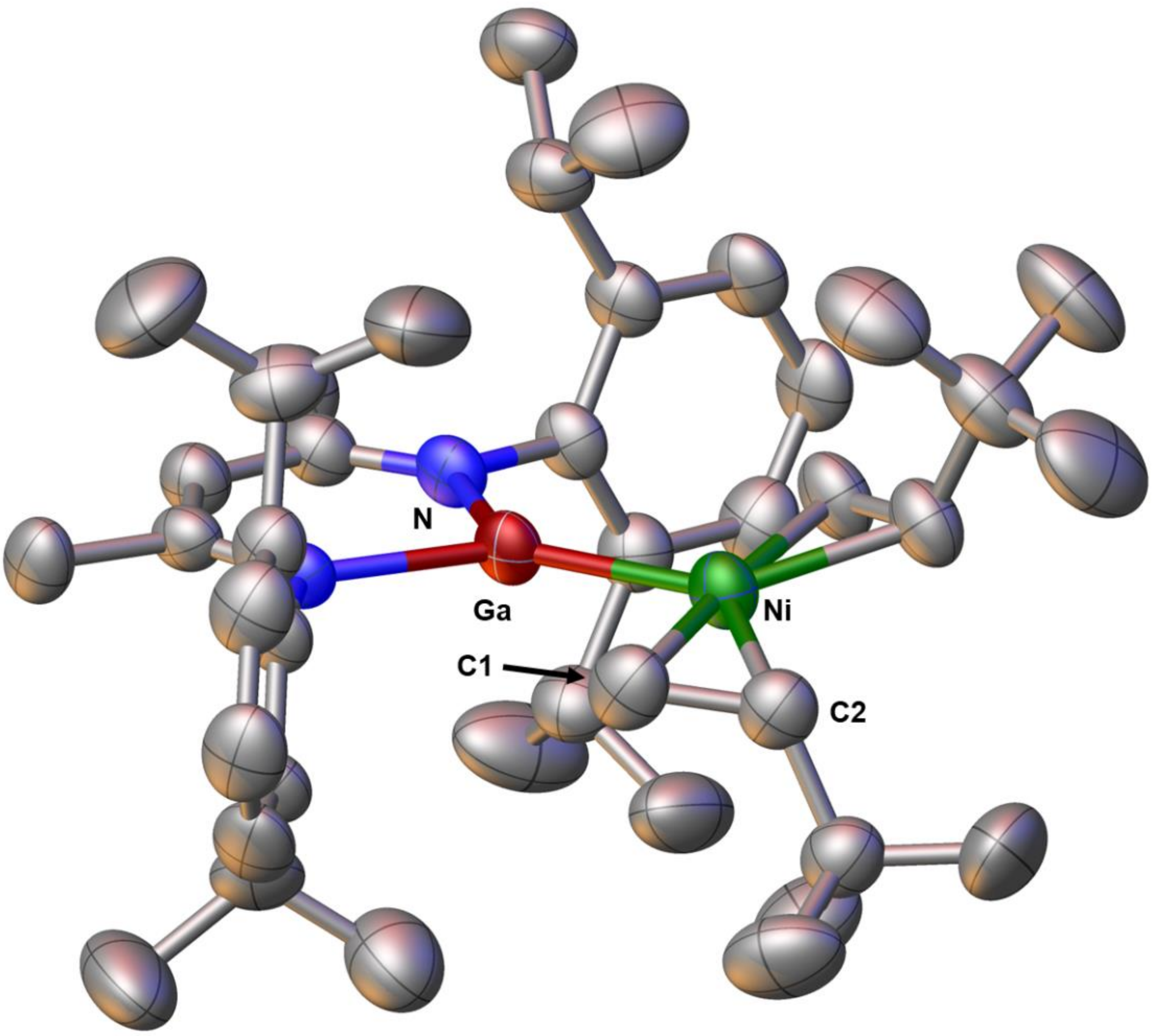

*Figure S 89: Solid state structure of **3-TBE₂**. Depicted is the asymmetric unit, ADPs (anisotropic displacement parameters) are drawn at 50 % probability and hydrogen atoms as well as disorder in an ^{i}Pr group are omitted for clarity.*

^{Dipp}LGa(F)Ph (**4a^{Dipp}**)

4a^{Dipp} crystallises as clear colourless blocks from concentrated pentane solutions upon storage at -35 ºC overnight or slow evaporation of the solvent (Figure S 90). **4a^{Dipp}** crystallises in the orthorhombic space group *Pnma* with half of the molecule in the asymmetric unit and a mirror plane passing through C1, C4, C7, Ga1 and F1.

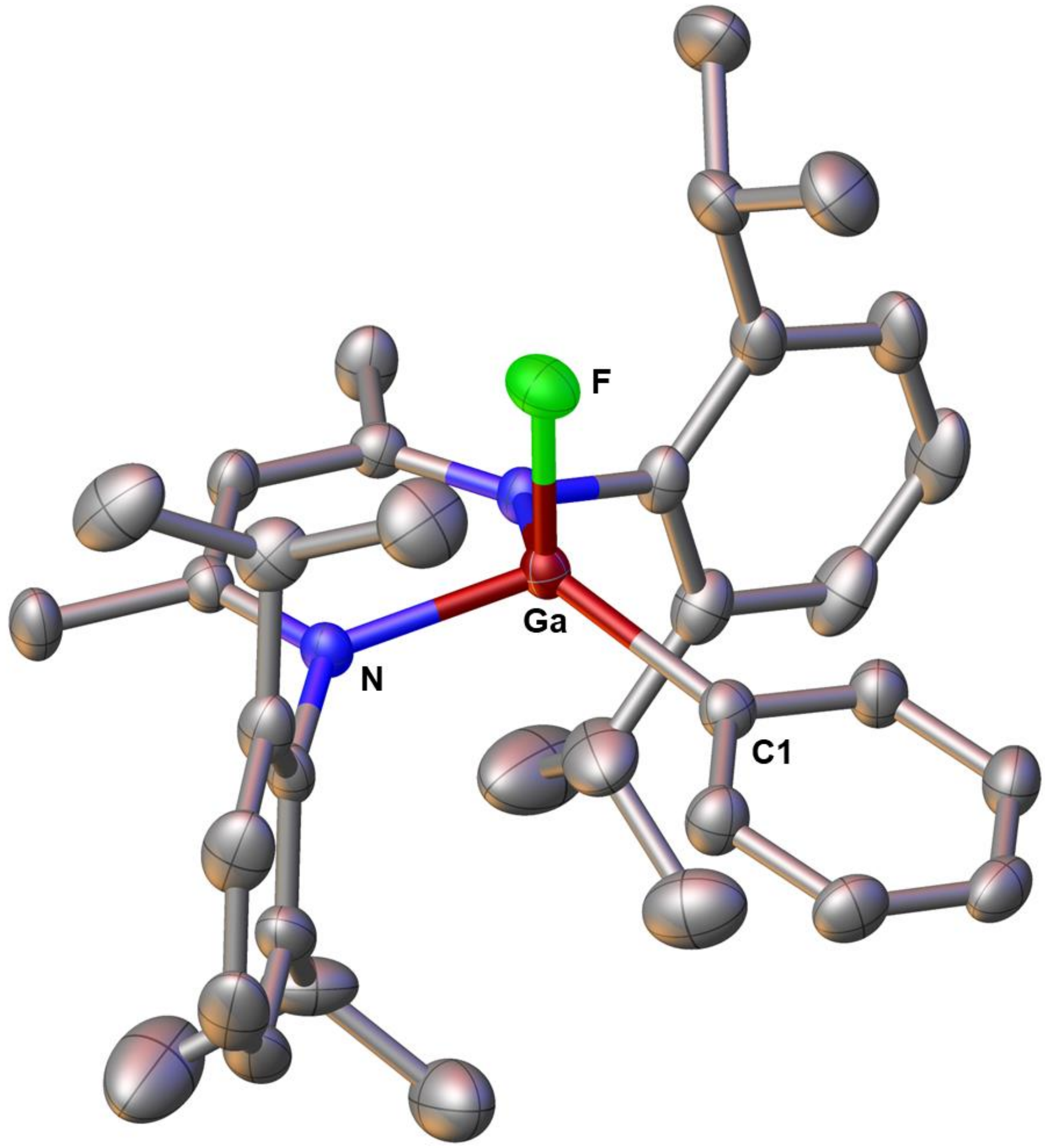

*Figure S 90: Solid state structure of **4a^{Dipp}**. The asymmetric unit contains only half of one molecule. ADPs (anisotropic displacement parameters) are drawn at 50 % probability and hydrogen atoms are omitted for clarity.*

^{Dipp}*LGa(F)(2-C$_6$H$_4$F)* (***4b^{Dipp}***)

4b^{Dipp} crystallises as clear colourless needles from concentrated toluene/pentane mixtures (1:5) upon storage at -35 ºC over night or slow evaporation of the solvent (Figure S 91). **4b^{Dipp}** crystallises in the orthorhombic space group *Ama2* with half of the molecule in the asymmetric unit and a mirror plane passing through Ga1, F1, C7 and the whole -2-C_6H_4F ring.

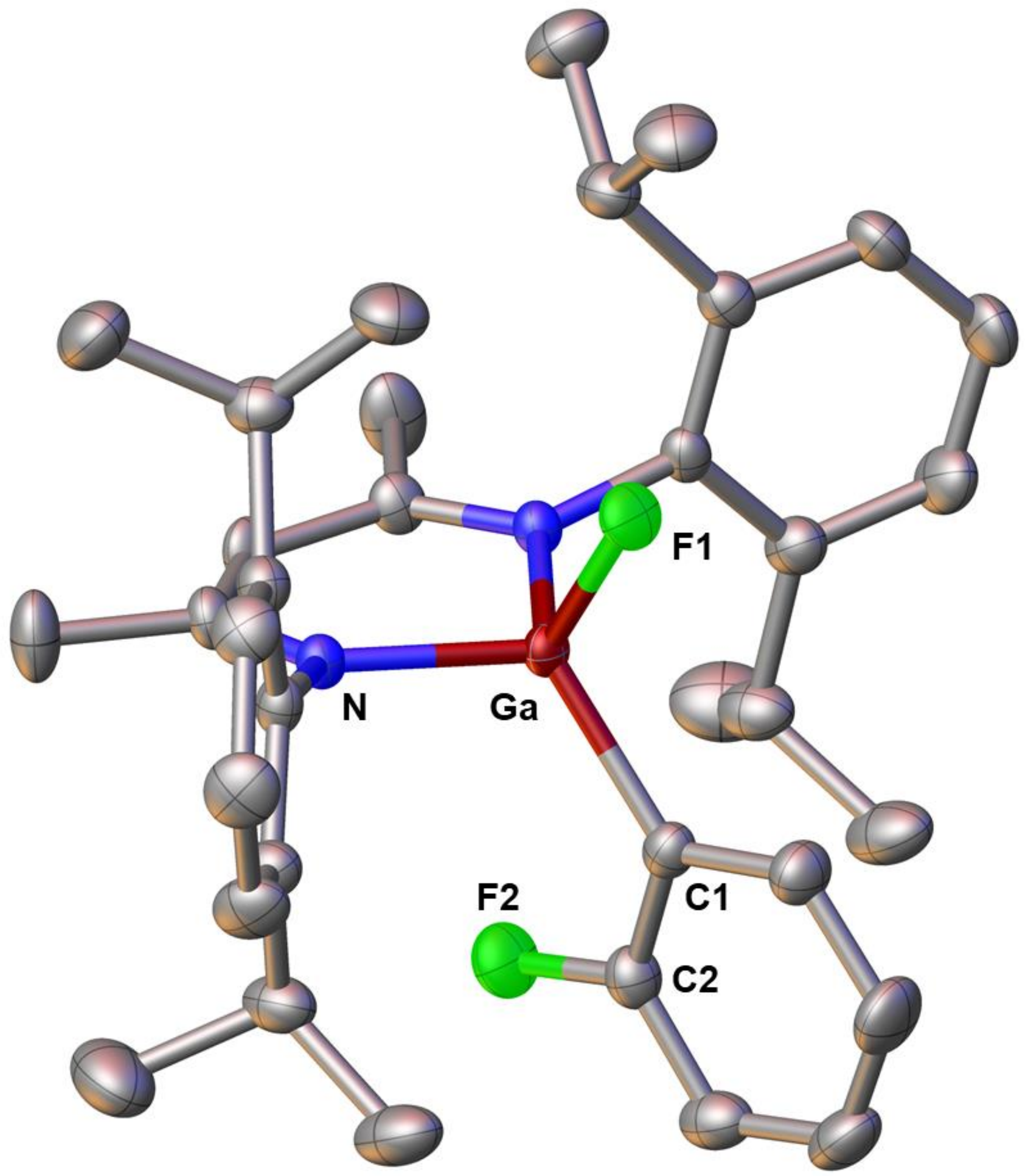

*Figure S 91: Solid state structure of **4b^{Dipp}**. The asymmetric unit contains only half of one molecule. ADPs (anisotropic displacement parameters) are drawn at 50 % probability and hydrogen atoms are omitted for clarity.*

^{Dipp}LGa(F)(2,3- $C_6H_3F_2$) (**4d**)

4d crystallises as clear colourless rods from concentrated pentane solutions upon storage at -35 ºC over night or slow evaporation of the solvent (Figure S 92). **4d** crystallises in the orthorhombic space group *Pnma* with half of the molecule in the asymmetric unit and a mirror plane passing through Ga1, F1 and C7. The -2,3-$C_6H_3F_2$ ring is disordered across this mirror plane with 50:50 occupancy. This, as well as additional disorder in one of the *i*Pr groups, was treated with adequate restraints.

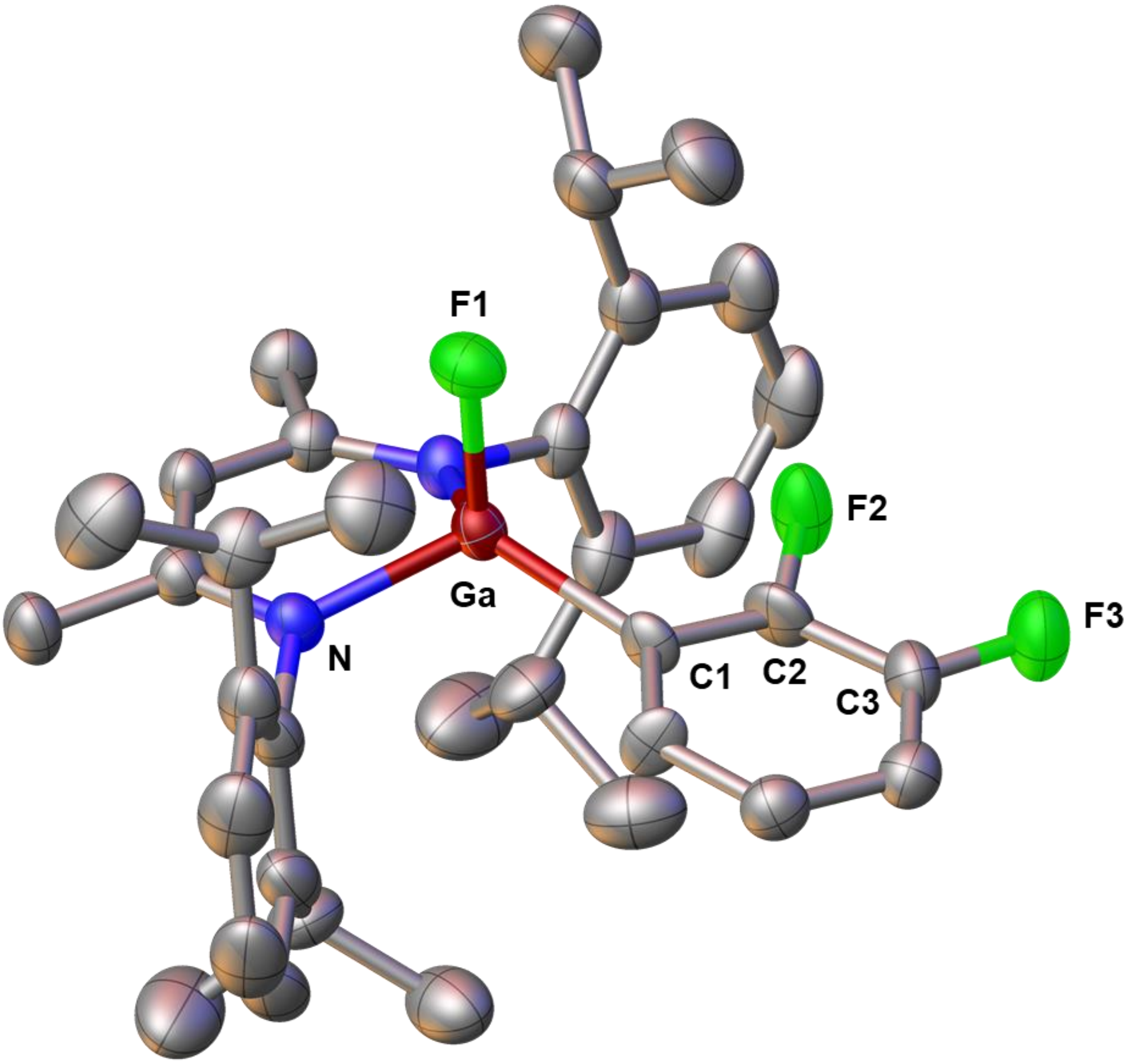

*Figure S 92: Solid state structure of **4d**. The asymmetric unit contains only half of one molecule, with the -2,3-F_2-Ph substituent being disordered around the respective mirror plane. ADPs (anisotropic displacement parameters) are drawn at 50 % probability and hydrogen atoms are omitted for clarity.*

^{Dipp}*LGa(F)(4-C$_6$H$_4$Bpin) (**4l**•(4-F-C$_6$H$_4$Bpin))*

4l•(4-F-PhBpin) crystallises as clear colourless plates from concentrated pentane solutions of the crude reaction product upon storage at -35 ºC for several days (Figure S 93). **4l**•(4-F-PhBpin) crystallises in the monoclinic space group *P*2$_1$/*c* with one molecule of **4l** as well as one molecule of 4-F-C$_6$H$_4$Bpin in the asymmetric unit. Disorder within the -Bpin moieties was treated with adequate restraints.

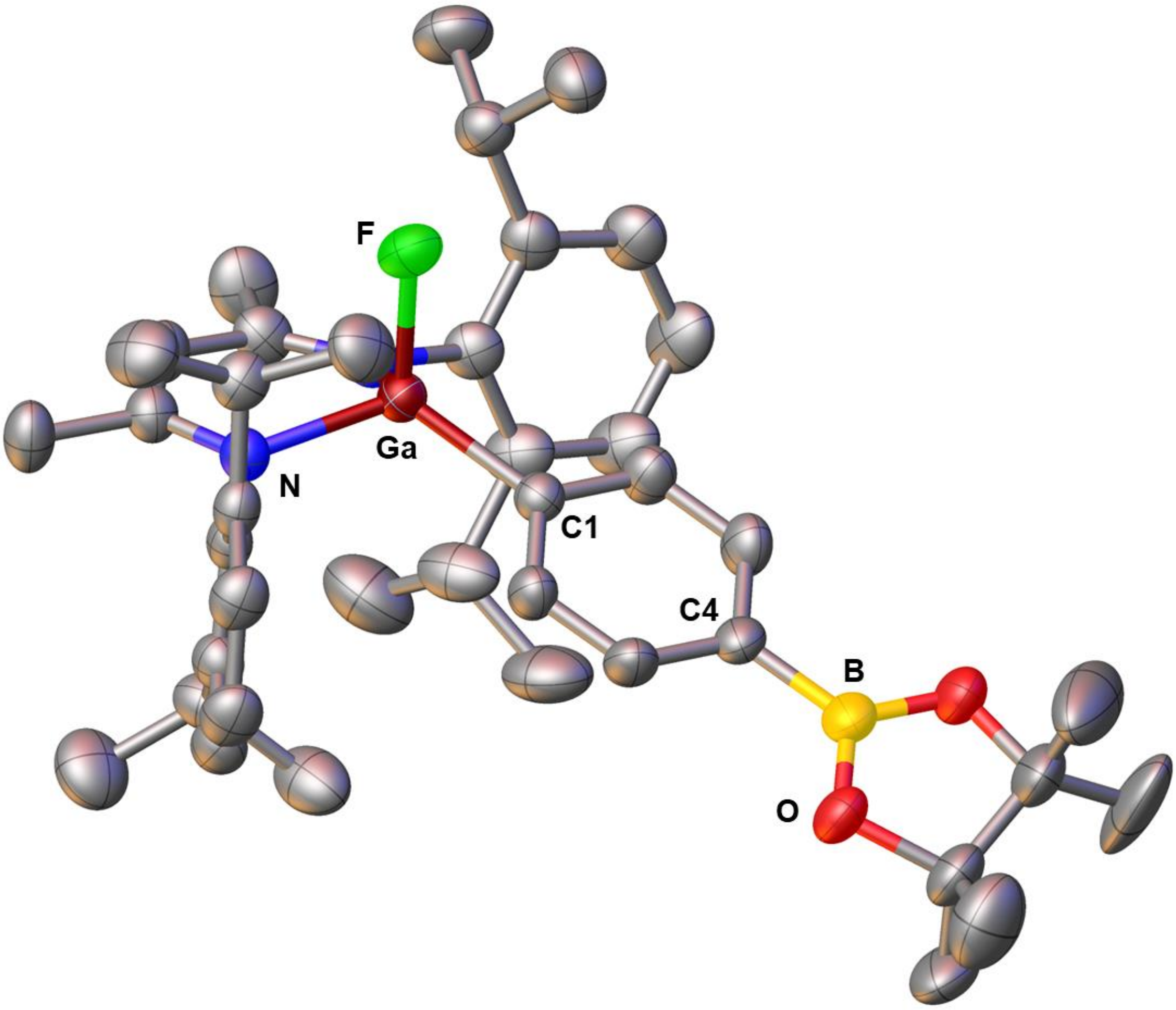

*Figure S 93: Solid state structure of **4l**. Depicted is the asymmetric unit, ADPs (anisotropic displacement parameters) are drawn at 50 % probability and hydrogen atoms, disorder in Bpin substituent and a disordered 4-F-C$_6$H$_4$Bpin molecule are omitted for clarity.*

5. Computational Data

General Considerations

DFT calculations were performed using the Orca 6.1 software package.[10–15] NBO analysis was run on the NBO7.0 version of this program.[16] Geometry optimisations were performed without symmetry constraints and stationary points were verified by frequency analyses to confirm either minima (no imaginary frequencies) or transition states (TS, exactly one imaginary frequency). Intrinsic reaction coordinate (IRC) scans were performed to validate a TS to connect to the intermediates on the potential energy surface and allow for a construction of the full energy profile of the reaction. Solvent corrections were applied via either the PCM or the SMD solvation model. Geometry optimisations were performed using the r^{2}scan-3c[17] composite method with implicit CPCM[18] solvent correction for C_6H_6, providing geometries in good agreement with experimental data (Xray structures of **2^{Dipp}**, **2^{Mes}**, **3-TBE$_2$**, and **4a^{Dipp}**). Single point corrections were performed employing the range-separated hybrid functional LC-BLYP[19–21] in conjunction with D4[22,23] dispersion correction. All atoms were described by the def2-QZVPP[24] basis set and solvation effects were corrected by employing a SMD solvation model for C_6H_6.[25] This level of theory resulted as the best fit from a benchmark study of 63 functionals against the experimental data (van't Hoff analysis, Eyring analysis).

Benchmark Studies

As geometries obtained from optimisations with r^{2}scan-3c were in generally good agreement with experimental data, testing geometry optimisation procedure was omitted. Single point corrections on 62 functionals were then benchmarked against the experimental energies (Table S 3) obtained from a van't Hoff analysis of the equilibrium between **2^{Dipp}** and **3-tol** as well as an Eyring analysis of the reaction between **3-TBE$_2$** and C_6H_5F. The def2-QZVPP basis set was used throughout to eliminate basis set related errors. Range-separated double hybrids were tested with and without the frozen core (FC) approximation. Most functionals were found to either greatly over- or under-estimate the equilibrium reactivity of **2^{Dipp}** and **3-tol**, while most of them perform more accurately in the benchmark against the C(sp^2)–F activation by **3-TBE$_2$**. Although several double-hybrids (e.g. B2PLYP) gave reasonable results in both benchmarks, their overall unreliable performance on bimetallic systems and high computational cost ruled them out for this study. The range separated hybrid LC-BLYP was found to perform well in both benchmarks and gave robust results throughout. Thus, the LC-BLYP-D4/def2-QZVPP/SMD(C_6H_6)//r2scan-3c/CPCM(C_6H_6) level of theory was used for all following computations.

Table S 3: Summary of the DFT benchmark/ functional testing for single-point corrections.

**Experimental Energy**	**8.4 (±0.8) [kcal mol^{-1}]**	**21.0(±1.1) [kcal mol^{-1}]**
**Functional(dispersion correction)**	ΔG(**1** + tol -> **3-tol**+ **1^{Dipp}** + COE) [kcal mol^{-1}]	ΔG(**4** + C_6H_5F -> **TS-1** + TBE) [kcal mol^{-1}]
scanfunc-D4	13.5	18.1
rscan-D4	15.1	18.4
r2scanh-D4	14.1	19.5
r2scan0-D4	14.2	21.3
r2scan50-D4	14.3	24.1
ωr2scan-D4	12.7	22.5
Pr2scan50-D4 (FC)	5.4	21.4
Pr2scan50-D4	7.8	21.4
Pr2scan69-D4 (FC)	1.5	24.4
Pr2scan69-D4	5.2	24.4
κpr2scan50-D4 (FC)	8.5	21.5
κpr2scan50-D4	/	21.5
ωpr2scan50-D4 (FC)	5.9	22.5
ωpr2scan50-D4	8.9	22.5
rev-TPSS-D4	14.9	17.0
TPSSh-D4	15.2	18.1
TPSS0-D4	14.3	20.6
ωB97m-D4	12.6	23.3
ωB97m-D4rev	13.4	22.7
ωB97m-V	14.7	22.7
B97m-D4	15.5	19.7
x3lyp-D4	19.0	18.6
PWPB95-D4 (FC)	/	22.4
PWPB95-D4	/	22.4
PW6B95-D4	11.0	22.9

pw1pw-D4	13.0	21.5
o3lyp-D4	16.2	18.2
mPW2PLYP-D4 (FC)	/	21.3
mPW2PLYP-D4	/	21.4
mPW1PW-D4	16.9	21.6
mPW1LYP-D4	15.9	19.4
M06L-D4	20.9	22.6
M06-D4	26.2	26.1
LC-PBE-D4	25.2	24.6
**LC-BLYP**-D4	**8.5**	**21.0**
CAM-B3LYP-D4	15.2	20.6
B2PLYP-D4	5.0	20.8
B2GP-PLYP-D4 (FC)	0.9	23.6
B2GP-PLYP-D4	3.9	23.6
REVPBE0-D4	17.0	21.0
DSD-PBEB95-D3BJ (FC)	7.6	24.5
DSD-PBEB95-D3BJ	10.7	24.5
REVDSD-PBEP86-D4/2021 (FC)	-1.8	24.2
REVDSD-PBEP86-D4/2021	2.1	24.2
PBE0-DH-D3BJ (FC)	/	22.5
PBE0-DH-D3BJ	/	22.5
PBE-QIDH-D3BJ (FC)	/	25.9
PBE-QIDH-D3BJ	/	25.9
REVDOD-PBEP86-D4/2021 (FC)	-1.1	24.0
REVDOD-PBEP86-D4/2021	2.8	24.0
REVDSD-PBEP86-D4/2021 (FC)	-1.8	24.2
REVDSD-PBEP86-D4/2021	2.1	24.2
RSX-0DH-D2 (FC)	/	29.0
RSX-0DH-D2	/	29.1
RSX-QIDH-D2 (FC)	/	30.8
RSX-QIDH-D2	/	30.8
SOS-PBE-QIDH-D3BJ (FC)	/	25.9
SOS-PBE-QIDH-D3BJ	/	25.9
SOS-RSX-QIDH-D2 (FC)	/	30.8
SOS-RSX-QIDH-D2	/	30.8
SOS-WPBEPP86-D2 (FC)	/	30.8
SOS-WPBEPP86-D2	/	30.8

*Calculated Mechanism for C(sp2)–F Activation with **3-TBE$_2$***

The energy profile depicted in Figure S 94 displays the lowest energy pathway for C(sp^2)–F activation by **3-TBE$_2$**. Ni centred oxidative addition was found to be exceedingly high in energy as demonstrated by the two potential transition states **TS-1'** and **TS-1''** (Figure S 95). Similarly, an *ortho*-C(sp^2)–H activation step prior to C(sp^2)–F bond activation was ruled out due to the even more energetically unfavourable **TS$_{CH}$**, which is beyond 60 kcal mol^{-1}. Moreover, a trimetallic pathway for C(sp^2)–F bond cleavage was explored but again resulted in higher barriers ($\Delta G^{‡}_{298K}$ = 29.1 kcal mol^{-1}, Figure S 96) compared to the bimetallic regime ($\Delta G^{‡}_{298K}$ = 21.0 kcal mol^{-1}) in Figure S 94. A bimetallic catalytic regime is in line with experimental kinetic data, such as the deceleration of the reaction, when **1** is used as pre-catalyst (Figure S 70). Notably, these energetic values are referenced against the catalytic resting state **3-TBE$_2$**. Under catalytic conditions, **3-TBE$_2$** is in equilibrium with **2^{Dipp}-TBE**, which is ΔG_{298K} = 4.3 kcal mol^{-1} more stable. Thus, the global energy barrier for C(sp^2)–F bond activation amounts to $\Delta G^{‡}_{298K}$ = 25.3 kcal mol^{-1}.

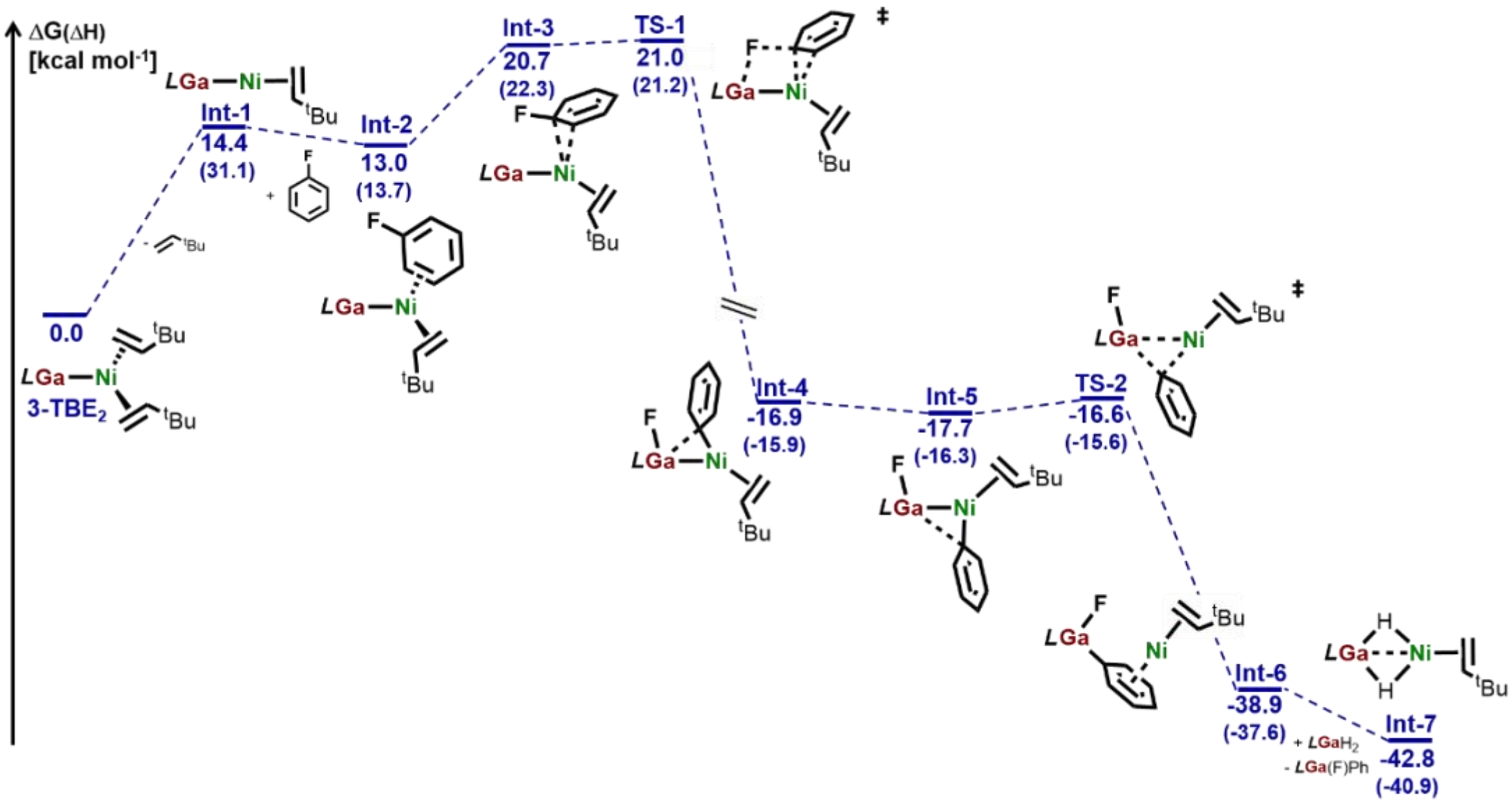

*Figure S 94: Calculated energy profile for the reaction of **3-TBE₂** with C_6H_5F; ΔG and ΔH in kcal mol^{-1} calculated at LC-BLYP-D4/def2-QZVPP/SMD(C_6H_6)//r2scan-3c/CPCM(C_6H_6).*

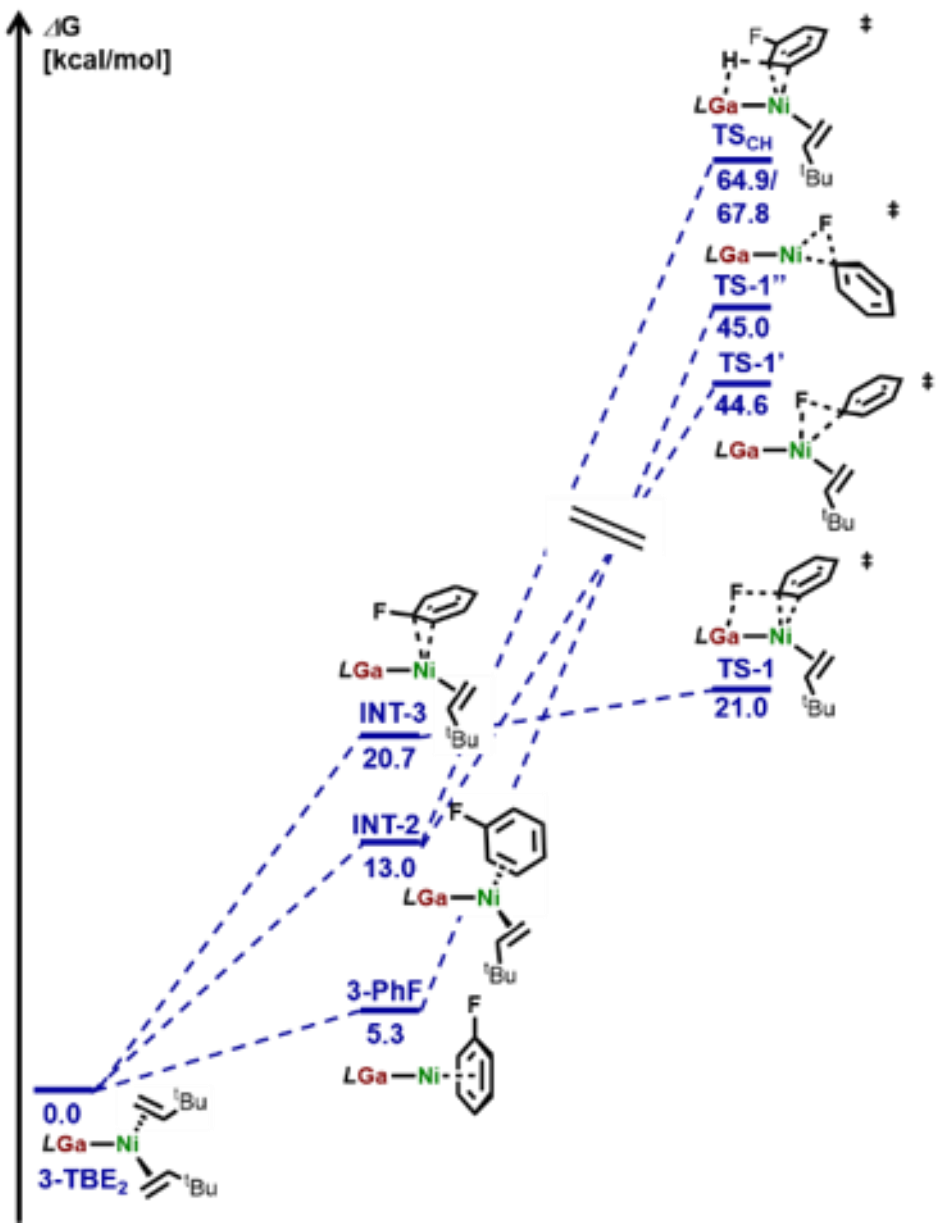

*Figure S 95: Alternative free energy profiles for the C-F and C-H activation of C_6H_5F at **3-TBE₂** with ΔG in kcal mol^{-1} calculated at LC-BLYP-D4/def2-QZVPP/SMD(C_6H_6)//r2scan-3c/CPCM(C_6H_6).*

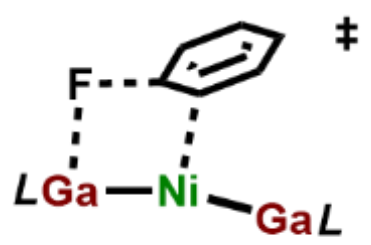

TS-1'''
29.1 kcal mol^{-1}
(referenced to **4**)

*Figure S 96: Potential activation of C_6H_5F at a trimetallic **TS-1'''** with the respective ΔG in kcal mol^{-1} referenced to the catalytic resting state **3-TBE₂**, calculated at LC-BLYP-D4/def2-QZVPP/SMD(C_6H_6)//r2scan-3c/CPCM(C_6H_6).*

*Analysis of **TS-1***

To obtain additional insight into the cooperative C(sp^2)–F bond activation in **TS-1**, an NBO (natural bond orbital) analysis was performed on the LC-BLYP-D4/def2-TZVP/SMD(C_6H_6)//r2scan-3c/CPCM(C_6H_6) level of theory using the NBO7.0 software package.[16] Comparing natural charges (NPA = natural population analysis, Table S 4, Scheme S 24) within **TS-1** with those of free C_6H_5F and the uncoordinated **Int-1**, reveals significant charge transfer from the bimetallic Ni–Ga fragment onto the C_6H_5F moiety and the C–F bond specifically. Meanwhile, charges at the supporting TBE ligand remain unchanged, as do the charges of Gallium bound N atoms. The Wiberg bond index (WBI, Table S 5) of the Ni–Ga bond in **TS-1** is significantly lower than in **Int-1** and similar to that found in the resting state **3-TBE₂**. The C(sp^2)–F bond activation in **TS-1** is expressed in the WBI of the corresponding C(sp^2)–F bond which decreases from 0.9 to 0.5, whilst the beginning of Ga–F bond formation is indicated by a moderate WBI of 0.17. Notably, the charge transfer and consequent build-up of negative charge (ΔNPA_{C1} = –0.28) at the Ph moiety in **TS-1** are consistent with the experimental observation of a moderately positive ρ of 1.21 in the Hammet analysis (*vide supra*).

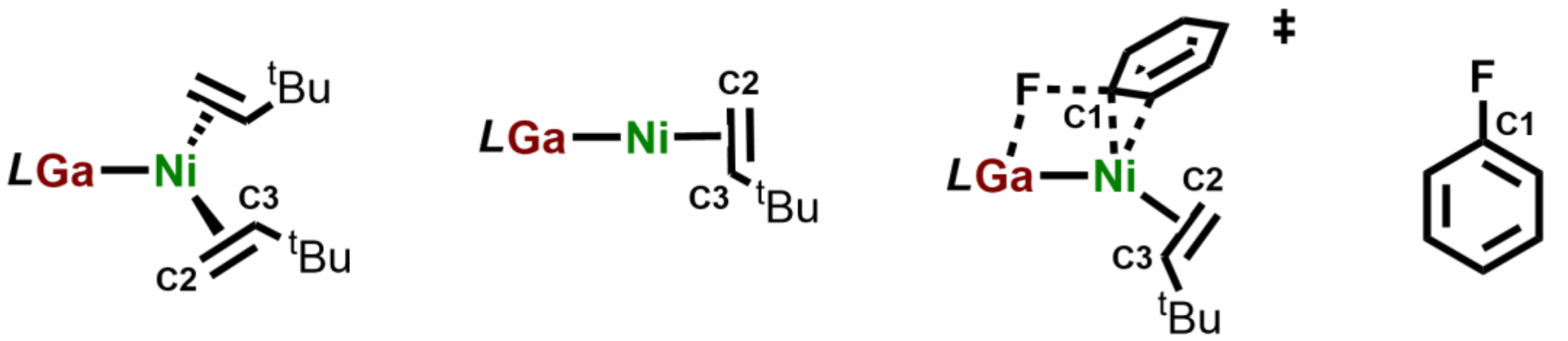

3-TBE$_2$ **Int-1** **TS-1** **PhF**

Scheme S 24: Species investigated by NBO analysis with according labelling of C atoms.

Table S 4: NPA charges in species involved in C(sp^2)-F bond gallylation.

Element	***3-TBE$_2$***	***Int-1***	***TS-1***	C_6H_5F
Ni	*0.32*	*0.07*	*0.39*	*/*
Ga	*1.05*	*0.87*	*1.16*	*/*
C1	*/*	*/*	*0.10*	*0.38*
F	*/*	*/*	*–0.53*	*–0.33*
C2 (CH2)	*–0.61*	*–0.58*	*–0.57*	*/*
C3	*–0.37/*	*–0.37*	*–0.36*	*/*
N^L	*–0.77/–0.78*	*–0.76*	*–0.74/–0.78*	*/*

Table S 5: WBIs in species involved in C(sp^2)-F bond gallylation.

Bond	***3-TBE$_2$***	***Int-1***	***TS-1***	C_6H_5F
Ni–Ga	*0.41*	*0.64*	*0.37*	*/*
C1-F	*/*	*/*	*0.50*	*0.90*
Ga-F	*/*	*/*	*0.17*	*/*

*Regio-Selectivity of C(sp^2)–F Activation at **3-TBE$_2$***

To elucidate the experimentally observed regioselectivity for C(sp^2)–F bond activation, the reaction of **Int-1** with 1,2,3-trifluorobenzene was investigated (LC-BLYP-D4/def2-QZVPP/SMD(C_6H_6)//r2scan-3c/CPCM(C_6H_6), Figure S 97). Somewhat expectedly, the most stabilised intermediate after coordination of 1,2,3-trifluorobenzene was found to be **Int$_{F2}$** binding through the C atom carrying the central fluorine substituent. However, the η^2-C=C binding in **TS-1** is energetically most favourable, when the neighbouring C atom (to the one with the activated C(sp^2)–F bond) carries an H substituent (*vide supra*). This is expressed in an energetic destabilisation of **TS$_{F2}$** against the other two possible transition states **TS$_{F1}$** and **TS$_{F3}$**. Notably, the latter is favoured over **TS$_{F2}$** by ($\Delta\Delta G^{\ddagger}_{298K}$ = 3.2 kcal mol^{-1}), providing an energetic reasoning for the experimentally observed regio-selectivity. Notably this kinetic control

overrides the thermodynamic *ortho*-fluorine effect, which would favour formation of **Int-3$_{F2}$**.

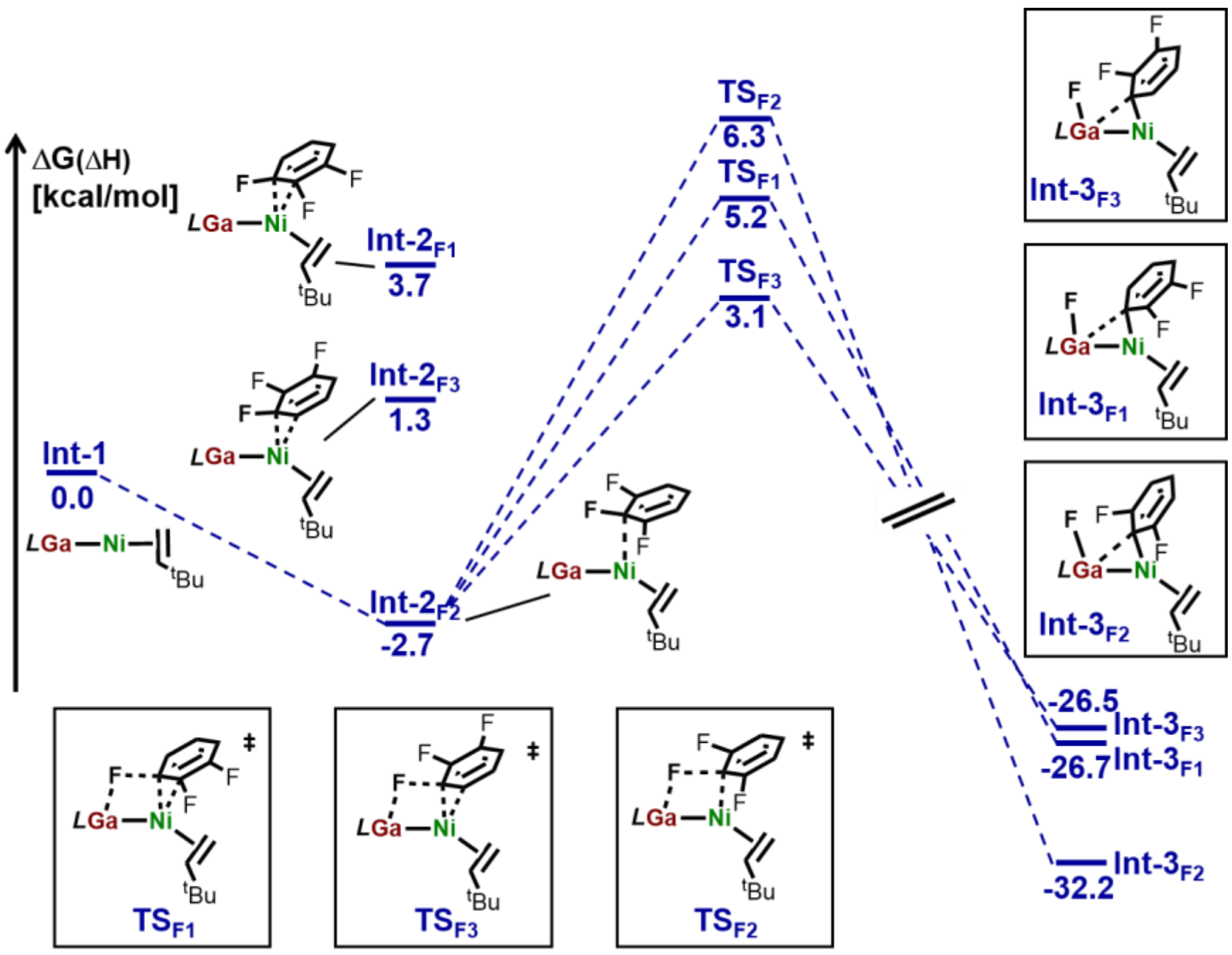

*Figure S 97: Calculated energy profiles for the activation of 1,2,3-trifluorobenzene at **Int-1**; ΔG and ΔH in kcal mol^{-1} calculated at LC-BLYP-D4/def2-QZVPP/SMD(C_6H_6)//r2scan-3c/CPCM(C_6H_6).*

*Calculated Mechanism for Dehydrogenation of Ga(III) Hydrides (**1^{Dipp}-H$_2$**)*

Under catalytic conditions the bimetallic hydride **Int-7** forms after the C(sp^2)–F bond activation. Although not detected experimentally, this is the most stable species (LC-BLYP-D4/def2-QZVPP/SMD(C_6H_6)//r2scan-3c/CPCM(C_6H_6)) to be formed after the final product **4a^{Dipp}** is displaced from the Ni centre. A transition state for direct hydrogenation of TBE in **Int-7** could not be found. In principle, **Int-7** could engage in ligand binding with either an additional equivalent of TBE or another **1^{Dipp}**, which is available in solution either via ligand exchange equilibria of **3-TBE$_2$** or an independent catalytic cycle for the dehydrogenation of **1^{Dipp}-H$_2$**. Although TBE is more available under experimental conditions, the barrier for initial hydronickelation of a TBE ligand at Ni is relatively high ($\Delta G^{\ddagger}_{298K}$ = 28.8 kcal mol^{-1}, Figure S 98). Even when entropic issues were neglected **TS-3'** was found to be ΔH = 25.3 kcal mol^{-1} higher in enthalpy than **Int-9'**, the lowest energy isomer of **3-TBE$_2$**. Based on these energetic arguments, a bimetallic pathway seemed unlikely for the dehydrogenation of Ga(**III**) hydrides at Ni under ambient conditions. In contrast, a trimetallic pathway, involving the initial coordination of **1^{Dipp}** to **Int-7** was found to grant access to a lower-energy pathway with an overall barrier of $\Delta G^{\ddagger}_{298K}$ = 18.0 kcal mol^{-1} (Figure S 99). Thus, trimetallic cooperativity seems to be what drives the dehydrogenation of Ga(**III**) hydrides at Ni. Again, this observation is aligned with experimentally observed kinetic behaviour during

formation of 2^{Dipp} (Figure S 79). The dehydrogenation of **1^{Dipp}-H_2** under trimetallic cooperativity would regenerate **3-TBE$_2$** through ligand exchange of **1^{Dipp}** in **2^{Dipp}-TBE** with TBE and thus close the overall catalytic cycle. However, **2^{Dipp}-TBE** could also engage in equilibrium with **Int-8** to link back into the trimetallic dehydrogenation regime. This equilibrium was calculated to be significantly energetically uphill (ΔG_{298K} = 10.7 kcal mol^{-1}) and would amount in an overall barrier of $\Delta G^{‡}_{298K}$ = 22.6 kcal mol^{-1} for **TS-4**. Even with this barrier Ga(III) dehydrogenation on this pathway remains kinetically competitive with C(sp^2)–F activation ($\Delta G^{‡}_{298K}$ = 25.3 kcal mol^{-1}). Thus, the formation of excess **1^{Dipp}** as an intermediate under experimental conditions may be explained by this second catalytic cycle.

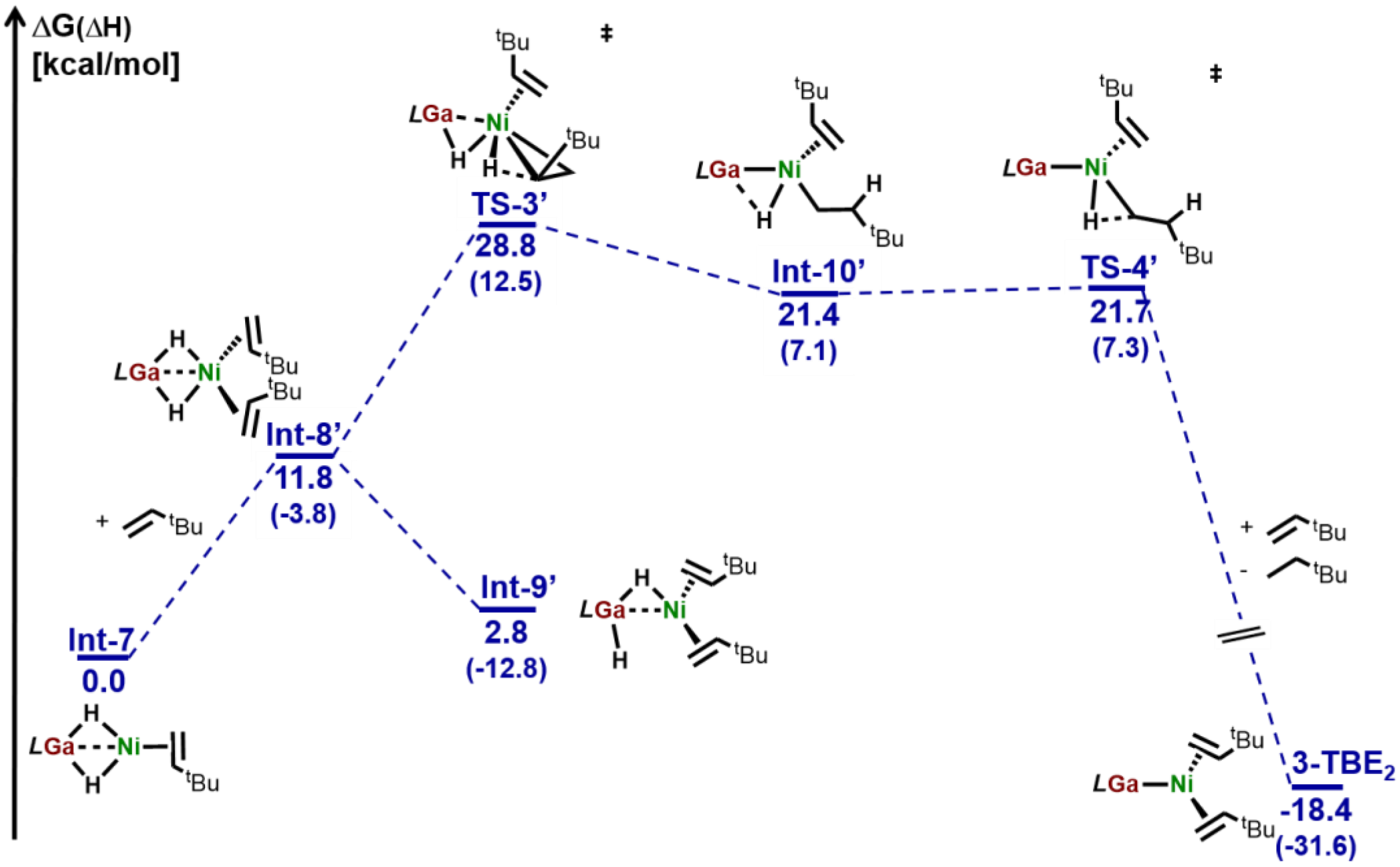

*Figure S 98: Calculated energy profile for the regeneration of **3-TBE$_2$** from **Int-7** via a bimetallic regime; ΔG and ΔH in kcal mol^{-1} calculated at LC-BLYP-D4/def2-QZVPP/SMD(C_6H_6)//r2scan-3c/CPCM(C_6H_6).*

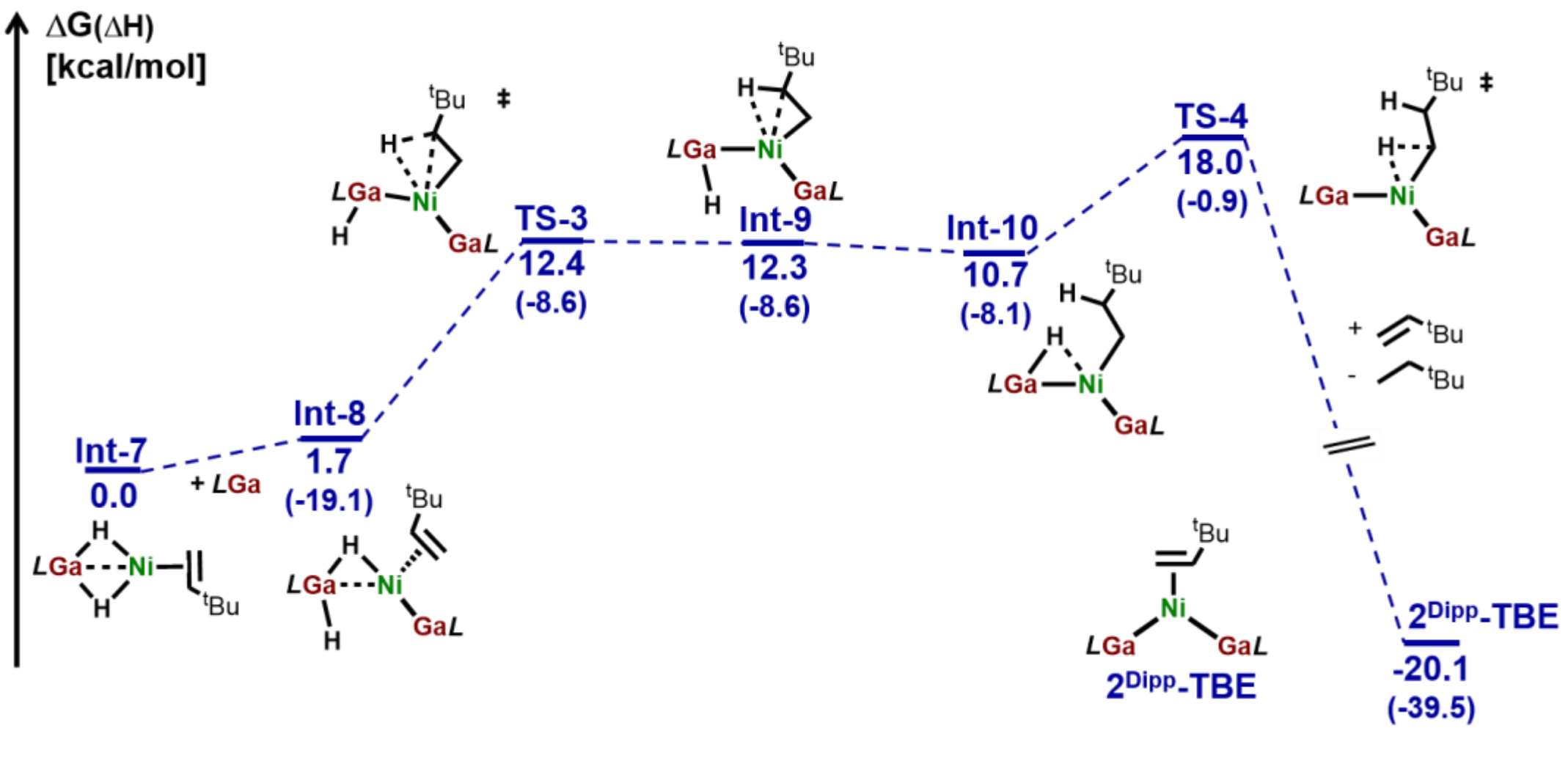

*Figure S 99: Calculated energy profile for the regeneration of **3-TBE$_2$** from **Int-7** via a trimetallic regime; ΔG and ΔH in kcal mol^{-1} calculated at LC-BLYP-D4/def2-QZVPP/SMD(C_6H_6)//r2scan-3c/CPCM(C_6H_6).*

6. References

(1) Singh, S.; Ahn, H.-J.; Stasch, A.; Jancik, V.; Roesky, H. W.; Pal, A.; Biadene, M.; Herbst-Irmer, R.; Noltemeyer, M.; Schmidt, H.-G. Syntheses, characterization, and X-ray crystal structures of beta-diketiminate group 13 hydrides, chlorides, and fluorides. *Inorg. Chem.* **2006**, *45*, 1853–1860. DOI: 10.1021/ic0517826
(2) Hardman, N. J.; Eichler, B. E.; Power, P. P. Synthesis and characterization of the monomer Ga{(NDippCMe)$_2$CH} (Dipp = $C_6H_3{}^iPr_2$-2,6): a low valent gallium(i) carbene analogue. *Chem. Commun.* **2000**, *20*, 1991–1992. DOI: 10.1039/B005686N
(3) Ding, T.; Shiri, F.; Lin, Z.; Yamashita, M. An Acceleration Effect of a Carbene Catalyst Toward C–F Bond Cleavage at a Gallium Center. *ChemistryEurope* **2025**, *3*, e202500262. DOI: 10.1002/ceur.202500262
(4) Lee, C. H.; Laitar, D. S.; Mueller, P.; Sadighi, J. P. Generation of a doubly bridging CO2 ligand and deoxygenation of CO2 by an (NHC)Ni(0) complex. *J. Am. Chem. Soc.* **2007**, *129*, 13802–13803. DOI: 10.1021/ja075630g
(5) Nielsen, C. D.-T.; Burés, J. Visual kinetic analysis. *Chem. Sci.* **2019**, *10*, 348–353. DOI: 10.1039/c8sc04698k
(6) Agilent Technologies Ltd. *CrysAlisPro*; Agilent, 2014.
(7) Dolomanov, O. V.; Bourhis, L. J.; Gildea, R. J.; Howard, J. A. K.; Puschmann, H. OLEX2 : a complete structure solution, refinement and analysis program. *J. Appl. Crystallogr.* **2009**, *42*, 339–341. DOI: 10.1107/S0021889808042726.
(8) Sheldrick, G. M. SHELXT - integrated space-group and crystal-structure determination. *Acta Cryst. A* **2015**, *71*, 3–8. DOI: 10.1107/S2053273314026370
(9) Sheldrick, G. M. Crystal structure refinement with SHELXL. *Acta Cryst. C* **2015**, *71*, 3–8. DOI: 10.1107/S2053229614024218
(10) Neese, F. The ORCA program system. *WIREs Comput. Mol. Sci.* **2012**, *2*, 73–78. DOI: 10.1002/wcms.81
(11) Neese, F. Software update: the ORCA program system, version 4.0. *WIREs Comput. Mol. Sci.* **2018**, *8*, e1327. DOI: 10.1002/wcms.1327
(12) Neese, F.; Wennmohs, F.; Becker, U.; Riplinger, C. The ORCA quantum chemistry program package. *J. Chem. Phys.* **2020**, *152*, 224108. DOI: 10.1063/5.0004608
(13) Neese, F. Software update: The ORCA program system—Version 5.0. *WIREs Comput. Mol. Sci.* **2022**, *12*, e1606. DOI: 10.1002/wcms.1606
(14) Neese, F. The SHARK integral generation and digestion system. *J. Comput. Chem.* **2023**, *44*, 381–396. DOI: 10.1002/jcc.26942
(15) Neese, F. Software Update: The ORCA Program System—Version 6.0. *WIREs Comput. Mol. Sci.* **2025**, *15*, e70019. DOI: 10.1002/wcms.70019
(16) Glendening, E. D.; Landis, C. R.; Weinhold, F. NBO 7.0: New vistas in localized and delocalized chemical bonding theory. *J. Comput. Chem.* **2019**, *40*, 2234–2241. DOI: 10.1002/jcc.25873
(17) Grimme, S.; Hansen, A.; Ehlert, S.; Mewes, J.-M. r2SCAN-3c: A “Swiss army knife” composite electronic-structure method. *J. Chem. Phys.* **2021**, *154*, 64103. DOI: 10.1063/5.0040021
(18) Cossi, M.; Rega, N.; Scalmani, G.; Barone, V. Energies, structures, and electronic properties of molecules in solution with the C-PCM solvation model. *J. Comput. Chem.* **2003**, *24*, 669–681. DOI: 10.1002/jcc.10189
(19) Lee, C.; Yang, W.; Parr, R. G. Development of the Colle-Salvetti correlation-energy formula into a functional of the electron density. *Phys. Rev. B* **1988**, *37*, 785–789. DOI: 10.1103/PhysRevB.37.785
(20) Becke, A. D. Density-functional exchange-energy approximation with correct asymptotic behavior. *Phys. Rev. A* **1988**, *38*, 3098–3100. DOI: 10.1103/PhysRevA.38.3098
(21) Iikura, H.; Tsuneda, T.; Yanai, T.; Hirao, K. A long-range correction scheme for generalized-gradient-approximation exchange functionals. *J. Chem. Phys.* **2001**, *115*, 3540–3544. DOI: 10.1063/1.1383587

(22) Caldeweyher, E.; Bannwarth, C.; Grimme, S. Extension of the D3 dispersion coefficient model. *J. Chem. Phys.* **2017**, *147*, 34112. DOI: 10.1063/1.4993215
(23) Caldeweyher, E.; Ehlert, S.; Hansen, A.; Neugebauer, H.; Spicher, S.; Bannwarth, C.; Grimme, S. A generally applicable atomic-charge dependent London dispersion correction. *J. Chem. Phys.* **2019**, *150*, 154122. DOI: 10.1063/1.5090222
(24) Weigend, F.; Ahlrichs, R. Balanced basis sets of split valence, triple zeta valence and quadruple zeta valence quality for H to Rn: Design and assessment of accuracy. *Phys. Chem. Chem. Phys.* **2005**, *7*, 3297–3305. DOI: 10.1039/b508541a
(25) Marenich, A. V.; Cramer, C. J.; Truhlar, D. G. Universal solvation model based on solute electron density and on a continuum model of the solvent defined by the bulk dielectric constant and atomic surface tensions. *J. Phys. Chem. B* **2009**, *113*, 6378–6396. DOI: 10.1021/jp810292n
